# Machine Learning-derived Multi-omics Prognostic Signature of Pyroptosis-related lncRNA with Regard to ZKSCAN2-DT and Tumor Immune Infiltration in Colorectal Cancer

**DOI:** 10.2174/1386207326666230823104952

**Published:** 2023-09-14

**Authors:** Jiamin Chen, Dan Jin, Liming Shao, Lingling Wang, Liuzhi Zhou, Jianting Cai

**Affiliations:** 1 Department of Gastroenterology, The Second Affiliated Hospital, Zhejiang University School of Medicine, Hangzhou 310009, Zhejiang Province, China;; 2 Institute of Immunology, Zhejiang University School of Medicine, Hangzhou 310009, China;; 3 Department of Surgical Oncology, Sir Run Run Shaw Hospital, Zhejiang University, Hangzhou 310000, Zhejiang Province, China

**Keywords:** Colon adenocarcinoma, pyroptosis-related lncRNA, prognosis, biomarkers, immune infiltration, colorectal cancer (CRC)

## Abstract

**Background:**

Colorectal cancer (CRC) has become the most prevalent gastrointestinal malignant tumor, ranking third (10.2%) in incidence and second (9.2%) in death among all malignancies globally. The most common histological subtype of CRC is colon adenocarcinoma (COAD), although the cause of CRC remains unknown, as there are no valid biomarkers.

**Methods:**

A thorough investigation was used to build a credible biomolecular risk model based on the pyroptosis-associated lncRNAs discovered for COAD prediction. Furthermore, Cibersort and Tumor Immune Dysfunction and Exclusion (TIDE), the methods of exploring tumor immune infiltration, were adopted in our paper to detect the effects of differential lncRNAs on the tumor microenvironment. Finally, quantitative real-time polymerase chain reaction (qPCR), as the approach of exploring expressions, was utilized on four different cell lines.

**Results:**

Seven pyroptosis-related lncRNAs have been identified as COAD predictive risk factors. Cox analysis, both univariate and multivariate, revealed that the established signature might serve as a novel independent factor with prognostic meaning in COAD patients. ZKSCAN2-DT was shown to be considerably overexpressed in the COAD cell line when compared to normal human colonic epithelial cells. Furthermore, ssGSEA analysis results revealed that the immune infiltration percentage of most immune cells dropped considerably as ZKSCAN2-DT expression increased, implying that ZKSCAN2-DT may play an important role in COAD immunotherapy.

**Conclusion:**

Our research is the first to identify pyroptosis-related lncRNAs connected with COAD patient prognosis and to construct a predictive prognosis signature, directing COAD patient prognosis in therapeutic interventions.

## 1 INTRODUCTION

In many categories of malignancies globally, colorectal cancer (CRC) ranks second (9.2%) for death and third (10.2%) for incidence [1]. It is now widely accepted that CRC tends to strike younger people [2]. CRC has been found to have multifactorial etiological factors, including genetic mutations, unhealthy lifestyle choices, such as smoking, excessive alcohol consumption, and a diet high in red meat and processed foods. Other risk factors may include obesity, chronic inflammation, and excessive exposure to industrial chemicals or environmental pollutants [3]. The etiological factors of CRC still deserve further in-depth research, and their factors may contradict our understanding. For example, a study has reported that pks+ E. coli may not be associated with the development of CRC in Filipinos [4]. Besides, the prognosis for CRC patients is still inadequate even after advanced comprehensive treatment strategies. For those patients diagnosed with distant metastasis, their survival rate is extremely low, just at 14% [2]. The most frequent histological subtype of CRC, colon adenocarcinoma (COAD), makes up over 90% of cases and has been widely studied [5].

Gasdermins (GSDMs) mediate the pathological type of programmed cell death known as pyroptosis. As a unique category of cell death, pyroptosis consists of the process of cell swelling, lysis, as well as the eventual release of inflammatory cytokines [6]. With GSDMs and cancer types in mind, pyroptosis plays a variety of multidimensional and context-specific functions in carcinogenesis, cancer proliferation, and anti-tumor immunity [7]. GSDMs is a family of proteins that contains six members, namely GSDMA, GSDMB, GSDMC, GSDMD, GSDME, and DFNA5 [8]. According to reports, GSDMC has an oncogenic role that might enhance tumor cell proliferation during CRC carcinogenesis [9]. GSDME is regarded as a tumor suppressor gene in CRC, and its expression is markedly reduced in colon cancer tissues [10]. The development of colitis-associated CRC may, however, be aided by GSDME-mediated pyroptosis, according to a recent study [11]. According to studies, the tumor-suppressing effects of GSDME depended on immunological surveillance, which could improve anti-tumor immunity by causing tumors to undergo inflammatory pyroptosis rather than non-inflammatory cell death, which is mediated by NK and CD8^+^ T killer cells [12]. Although pyroptosis still possesses an underlying association with anti-cancer immunity, growing numbers of evidence suggest that pyroptosis-mediated tumor eradication was accomplished by stimulating the immune system [13]. Intriguingly, some chemotherapeutic drugs can enhance their effectiveness *via* inducing pyroptosis through caspase-3 cleavage of GSDME and activating anti-tumor immunity [14, 15]. Patients with advanced CRC may benefit from immunotherapy, a potent therapeutic approach that relies on anti-tumor immunity [16]. In recent years, the investigations involving pyroptosis and cancers have provided novel research insights for the prevention and intervention of cancers. The relationship between genes related to pyroptosis and the prognosis and anti-tumor immunity of COAD patients still exists, nevertheless.

Although it lacks a coding function, long non-coding RNA (lncRNA), whose RNA transcript is longer than 200 nucleotides, is an essential regulator of gene transcription and post-transcriptional modification [17]. LncRNA has significant potential for diagnostic and prognostic applications. For example, Zhang *et al*. developed a prognostic lncRNA profile linked to cuproptosis that can forecast immunotherapy response in hepatocellular carcinoma patients [18]. Besides, lncRNA also has excellent application prospects in the diagnosis prediction of Epstein-Barr Virus-associated cancers [19]. As a major factor in controlling tumor immunity, lncRNAs have been shown to control inflammation and take part in the production of immune genes, which has an impact on the tumor immune microenvironment [20]. At the same time, the crucial role that lncRNAs can successively mediate cell pyrolysis has also been verified [21]. Pyroptosis-associated lncRNAs have been linked to tumor immunity in COAD, although their prognostic values and biomarker functions have not yet been documented.

Our study is the first to identify pyroptosis-related lncRNAs associated with the prognosis of patients with COAD and investigate their effects on tumor immune infiltration. We also developed an engineering predictive prognosis signature, providing valuable insights for future therapeutic interventions. Additionally, our experimental data confirmed the expression levels of differential pyroptosis-related lncRNAs in COAD.

## 2 MATERIALS AND METHODS

### 2.1 Data Processing and Pyroptosis-related lncRNA

The TCGA database yielded a total of 473 cases of colon adenocarcinoma. Patients with COAD who lacked complete clinical or survival data (n=21) were removed. Also, the following study contained 452 samples altogether (Table **1**). According to their Ensembl IDs, genes were briefly divided into protein-coding and non-coding genes [22]. From GENCODE, the GRCH37 long non-coding RNA annotation file was retrieved. From previously published literature, 52 genes associated with pyroptosis were found [23]. To investigate the relationship between 52 pyroptosis-related genes and 14086 lncRNAs, we used Pearson correlation analysis. In the end, 1730 lncRNAs associated with pyroptosis were verified with correlation coefficients |Cor| > 0.4 and *P*-0.01.

### 2.2 Identification of Prognostic Pyroptosis-related Hub lncRNA

We analyzed the 1730 pyroptosis-related lncRNAs in order to investigate their predictive potential. The first step was to identify 277 differential pyroptosis-related lncRNAs. The pyroptosis-related lncRNA was discovered using univariate Cox analysis, along with the LASSO regression. Additionally, the multivariate Cox analysis was also applied. Finally, according to the regression coefficient of multivariate Cox analysis and lncRNA expression, a novel predictive pyroptosis-associated lncRNA risk signature was developed using a total of 7 pyroptosis-related lncRNAs. Risk score = β1 × Expression_lncRNA1_+β2 × Expression_lncRNA2_+…+ βn × Expression_lncRNAn_ [24].

### 2.3 Confirmation of Pyroptosis-related lncRNA Signature

Patients' clinical data were gathered and integrated based on the appropriate risk score. Furthermore, patients were allocated into two groups, namely, high-risk subpopulation, and low-risk subpopulation, according to the optimal risk score. Next, survival analysis comparing two subpopulation was applied *via* Kaplan-Meier curve. Additionally, to evaluate the predictive ability of the model, the 1-, 3-, and 5-year ROC curves were presented [25]. Furthermore, using Cox regression analysis, both univariate and multivariate, we combined gender, and age, along with stage, TNM, and risk scores, to assess their prognostic markers' accuracy with forest plots.

### 2.4 Development of ROC and Nomogram

To evaluate the prognostic ability of risk score and other clinical features (including age, gender, stage and TNM), the ROC curves of 1/3/5-year were constructed and plotted to evaluate the AUC value [25]. Additionally, we developed a nomogram including risk scores, age, gender, stage, and TNM to statistically assess the effectiveness of our signature's 1/3/5-year prognostic capability. Furthermore, the capability of the nomogram was detected by plotting a 1/3/5-year calibration curve.

### 2.5 Role of Pyroptosis-related lncRNA Signature in Clinicopathological Features

We compared the risk score in several clinical feature stratifications based on the clinical characteristics of the patients (*i.e.,* age, gender, pathologic stage, AJCC-TNM.). In order to examine if our signature played a significant influence in the onset and development of COAD, a survival curve was applied to assess the overall survival status of various stratifications.

### 2.6 Tumor Immune Infiltration and Immune Checkpoint Inhibitors

Based on previous research, Cibersort was utilized to calculate the number of immune cells in each sample [26]. Besides, we conducted ssGSEA analysis to evaluate the function of infiltrating immune cells [27]. Furthermore, TIDE was aimed at assessing the immune responses of each patient [28]. From previously published literature, immune checkpoint genes were found that may be relevant to immune checkpoint therapy [29-32]. In order to investigate the possible use of our model in COAD immune checkpoint therapy, we examined whether our model is associated with 47 immune checkpoint genes.

### 2.7 Evaluation of Pyroptosis-related lncRNA Expression in Immune Cells

Our study utilized the Tumor Immune Single Cell Hub Database (TISCH) [33], a comprehensive scRNA-seq atlas that was constructed by collecting data from both the Gene Expression Omnibus and ArrayExpress databases. From TISCH, we conducted research to evaluate the expression levels of pyroptosis-related lncRNAs, specifically within immune cells.

### 2.8 Cell Culture and Q-PCR

NCM460 (human colonic epithelial cell) and three human COAD cell lines (SW480, RKO, and HCT116 cells) were cultured in DMEM medium containing 10% fetal bovine serum (FBS). Quantitative real-time polymerase chain reaction was performed on four different cell lines. TRIzol reagent extracted total RNA according to the instruction manual. Next, total RNA was transferred into cDNA using RNA reverse transcription kit. qRT-PCR was performed on cDNA in the applied Biosystems 7500 Fast Real-Time PCR System to determine the ZKSCAN2-DT level. All samples were made in triplicate, and β-actin functioned as a control. Additionally, primer sequences used in PCR were listed as follows: ZKSCAN2-DT, forward TCCAGCTTGGTTGACAAAGTGAGAC and reverse, CCTCCTCGCC TTGCTCTTAATGC; β-actin, Forward, ATCGTGCGT GACATTAAGGAGAAG and Reverse, AGGAAGGAAGG CTGGAAGAGTG.

### 2.9 Statistical Analysis

Using R software and the Perl language, all results were examined. To determine the statistical significance of two variables, the Student's t-test was used. Statistical significance was considered as *P<0.05, **P<0.01, ***P<0.001.

## 3 RESULTS

### 3.1 Seven Prognostic Pyroptosis-related Hub lncRNA were Identified

In addition, 52 genes relevant to pyroptosis were compiled from published literature (Table **S1**). To identify the pyroptosis-associated lncRNAs, we used Person correlation analysis. Finally, 1730 lncRNAs associated with pyroptosis were discovered (Table **S2**).

**Fig. (1):**
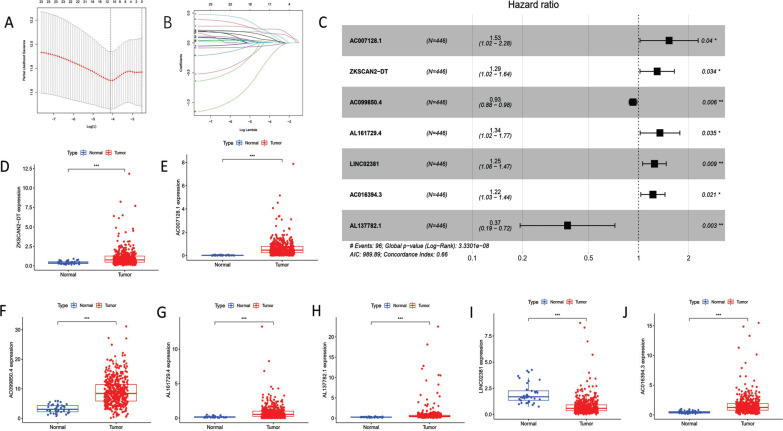
The results of 7 pyroptosis-related lncRNAs based in the multivariate Cox regression analysis. (**A**) The validation *via* LASSO regression. (**B**) LASSO regression coefficient of 7 pyroptosis-related lncRNAs. (**C**) The forest plot shown 7 independent prognostic pyroptosis-related lncRNAs in TCGA-COAD. (**D**) ZKSCAN2-DT expression in TCGA-COAD (*P*<0.001). (**E**) AC007128.1 expression in TCGA-COAD (*P*<0.001). (**F**) AC099850.4 expression in TCGA-COAD (*P*<0.001). (**G**) AL161729.4 expression in TCGA-COAD (*P*<0.001). (**H**) AL137782.1 expression in TCGA-COAD (*P*<0.001) (**I**) LINC02381 expression in TCGA-COAD (*P*<0.001). (**J**) AC016394.3 expression in TCGA-COAD (*P*<0.001).

Then, based on the “limma” package, 277 distinct pyroptosis-related lncRNAs were discovered (Table **S3**). On the 277 variables, we performed LASSO regression analysis, along with Cox analysis, both univariate and multivariate (Fig. **1A** and **B**). Ultimately, it was determined that seven pyroptosis-related lncRNAs were biological prognostic markers in COAD patients. All 7 of the pyroptosis-related lncRNAs were shown in Fig. (**1C**) and Table **2** to be risk factors for the prognosis of COAD. Additionally, TCGA-COAD transcription data revealed that these pyroptosis-related lncRNAs were significantly expressed differently in COAD patients (Fig. **1D**-**1J**).

### 3.2 Confirmation of a Pyroptosis-related lncRNA Signature

**Fig. (2):**
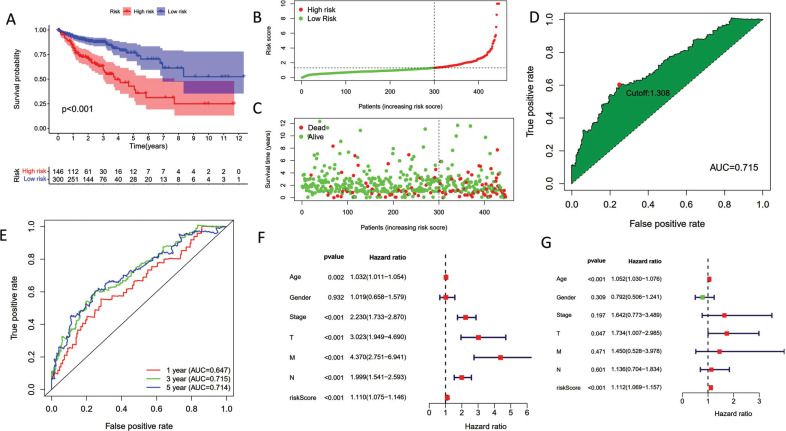
Confirmation of the pyroptosis-related lncRNA signature. (**A**) Survival analysis indicated that subgroup with high-risk score subpopulation possessed a miserable OS. (**B**) The scatter plot of risk scores. (**C**) The scatter plot of survival status. (**D**) The optimal cutoff value as the threshold in ROC curve analysis. (**E**) ROC analysis for predicting 1/3/5-year OS was performed using risk scores. (**F**) Univariate Cox analysis combined with other clinical features. (**G**) Multivariate Cox analysis combined with other clinical features.

According to Fig. (**2A**), the high-risk subpopulation had a more miserable prognosis outcome than the low-risk group. COAD patients in the low-risk category possessed a greater prognosis result and decreased death, according to the distribution of risk scores (Fig. **2B** and **2C**). We split the group of COAD patients into high-risk subpopulation and low-risk subpopulation according to the optimal risk score value for each COAD patient (Fig. **2D**). The ROC curves were examined with the aim of assessing the diagnostic functions of the risk score (Fig. **2E**, AUC1-year=0.647, AUC3-year=0.715, AUC5-year=0.714). Additionally, univariate Cox analysis showed that risk score might be a standalone prognostic factor in COAD patients when paired with other clinical parameters (Fig. **2F**, HR=1.110, 95% CI: 1.069-1.157). Similarly, the HR value of multivariate Cox regression analysis was 1.112 (Fig. **2G**, HR=1.112, 95% CI: 1.069-1.157).

### 3.3 Predictive Analysis of the Pyroptosis-related lncRNA Signature with ROC Curve and Nomogram

**Fig. (3):**
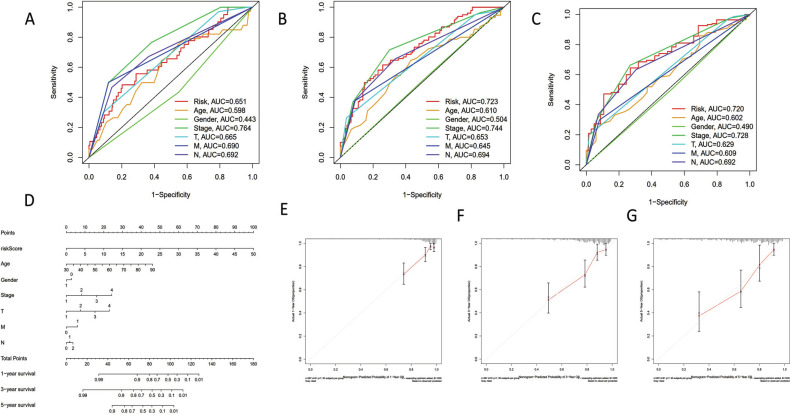
Predictive analysis of the pyroptosis-related lncRNA signature with ROC curve and nomogram. (**A**) ROC curve for 1-year OS with different clinicopathological features. (**B**) ROC curve for 3-year OS with different clinicopathological features. (**C**) ROC curve for 5-year OS with different clinicopathological features. (**D**) Nomogram quantitatively shown the predictive accuracy of our model among age, gender, stage, and TNM. (**E**) Calibration curve for 1-year OS in nomogram. (**F**) Calibration curve for 3-year OS in nomogram. (**G**) Calibration curve for 5-year OS in nomogram.

Gender, age, tumor stage, and TNM were gathered as potential prognostic markers in order to confirm the predictive performance of our signature in comparison to other clinical parameters. When we compared our risk signature to other parameters, we discovered that it had a strong and reliable capacity for predicting the overall survival status of COAD atients (Fig. **3A**-**3C**). Additionally, we statistically use the nomogram to demonstrate the model's ability to predict changes in age, gender, stage, and TNM (Fig. **3D**). Our risk model displayed independent prognostic prediction potential for 1/3/5-year OS in COAD patients, as demonstrated by the calibration curve (Fig. **3E**-**3G**). All things considered, the risk signature we developed had a strong and precise predictive capacity to direct COAD prediction.

### 3.4 Association with other Clinicopathological Features

**Fig. (4):**
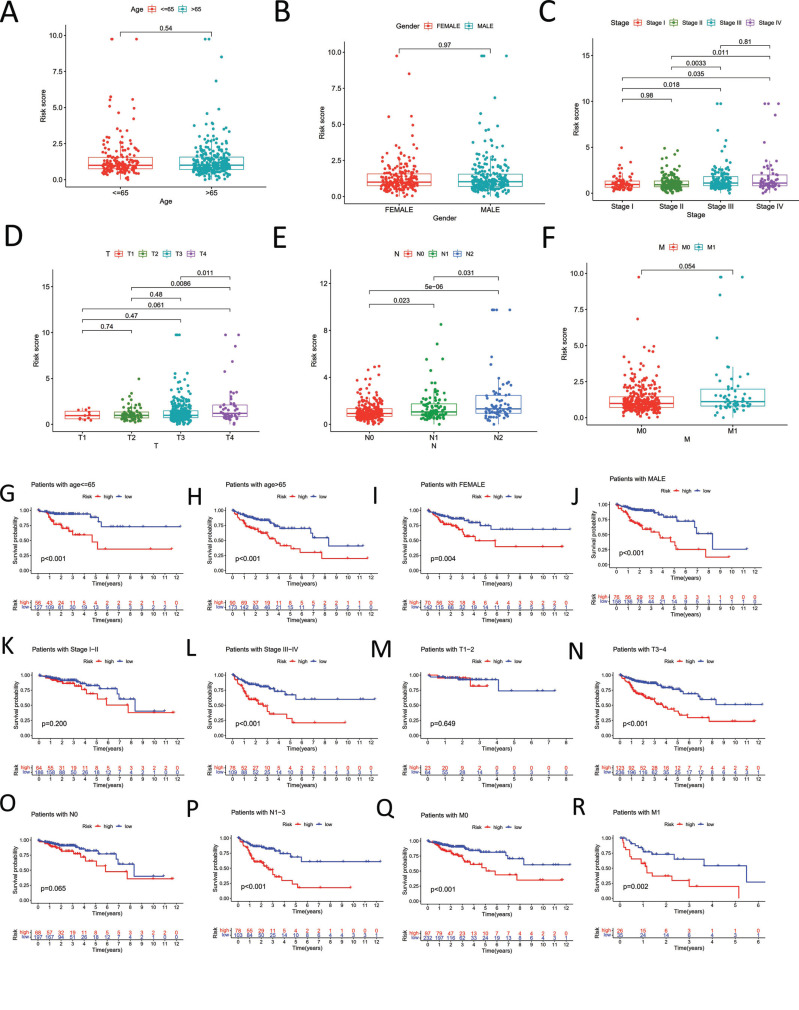
Association with other clinicopathological features. (**A**) Association between risk score and age. (**B**) Association between risk score and gender. (**C**) Association between risk score and pathological stage. (**D-F**) Association between risk score and AJCC-TNM. (**G-R**) Kaplan–Meier survival analysis of COAD patients with high-risk score in various subgroups.

Age and gender, however, were not yet statistically significant (Fig. **4A** and **4B**). In addition, we sought to look at how our signature related to other clinicopathological traits in TCGA-COAD. Student's t-test results showed that stage and TNM significantly differed in how the risk score was displayed. In other words, the risk score steadily grew as the stage, or TNM advanced (Fig. **4C**-**F**). Additionally, we conducted a stratified analysis to see if a particular discovered lncRNA may predict OS in different COAD subgroups. The COAD patient with a poor prognosis in various categories (*e.g*., age65, age>65, female, stage, TNM, *etc*.) is shown in Fig. (**4G**-**4R**).

### 3.5 TME Features and Immune Infiltration in TCGA-COAD

**Fig. (5):**
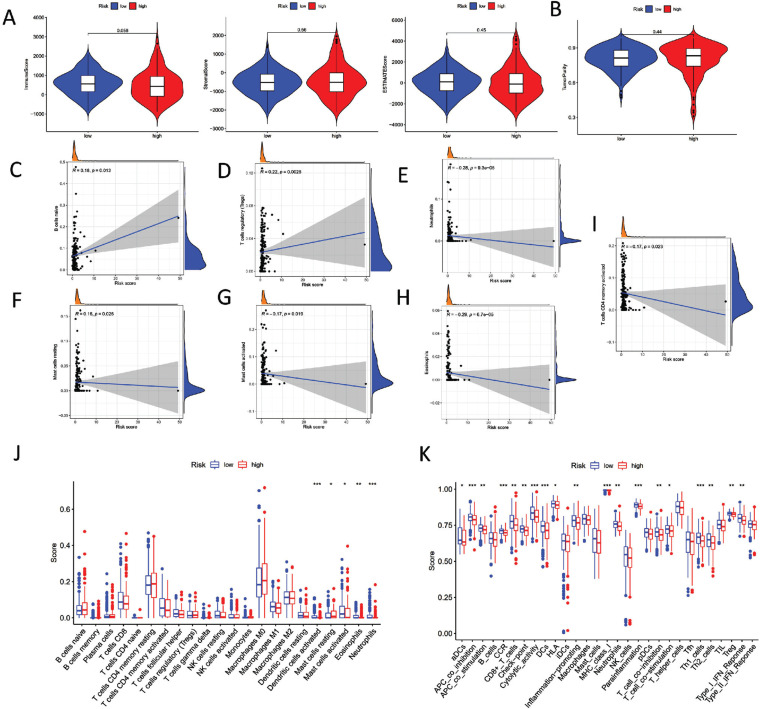
The TME features and immune infiltration in TCGA-COAD. (**A**) Association between risk score and Immune score, Stromal score and ESTIMATE score. (**B**) Association between risk score and Tumor purity. (**C**) Association between risk score, and B cells naïve. (**D**) Association between risk score and T cells regulatory. (**E**) Association between risk score and Neutrophils. (**F**) Association between risk score and Mast cells resting. (**G**) Association between risk score and Mast cells activated. (**H**) Association between risk score and Eosinophils. (**I**) Association between risk score and T cells CD4 memory activated. (**J**) Cibersort shown the tumor immune infiltration fraction of multiple immune cells between high-risk score subgroup and low-risk score subgroup. (**K**) ssGSEA exhibited differential significance of 29 immune cell functions in two groups.

We further explored whether the risk signature we established was associated with tumor microenvironment characteristics and immune infiltration in TCGA-COAD. The ESTIMATE algorithm exhibited that the high-risk group of COAD patients was significantly linked to a lower immune score and elevated tumor purity (Fig. **5A**-**5C**). On the contrary, it was significantly positively associated with B cells naïve and Tregs (Fig. **5C** and **5D**). Besides, we found that the signature as constructed was a notably negative correlation with neutrophils, mast cells resting, mast cells activated, eosinophils, and T cells CD4 activated (Fig. **5E**-**5I**). Cibersort showed the tumor immune infiltration statutes between high- and low-groups (Fig. **5J**). Our ssGSEA results exhibited the differential distribution of 29 immune cell functions in two subgroups (Fig. **5K**). The outcomes provided strong evidence that the signature, as established, was significantly associated with TME features and immune infiltration.

### 3.6 Correlation between the Pyroptosis-related lncRNA Model and all Crucial Immune Checkpoint Inhibitors

**Fig. (6):**
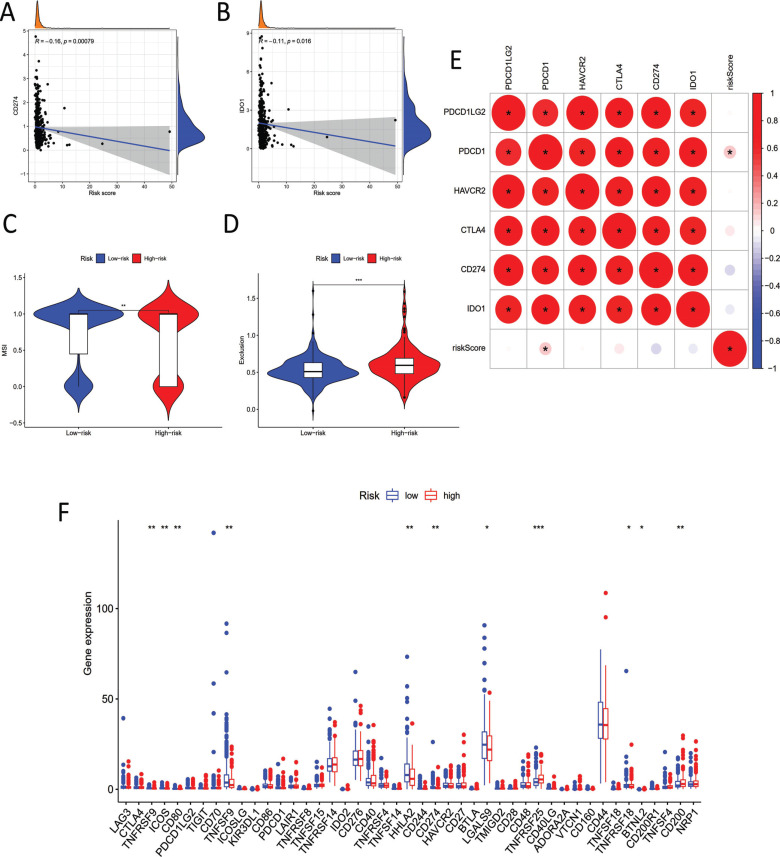
Association between the pyroptosis-related lncRNA signature and all crucial immune checkpoint inhibitors. (**A**) Association between CD274 and risk score. (**B**) Association between IDO1 and risk score. (**C**) Association between MSI and risk score. (**D**) Association between TIDE and risk score. (**E**) Correlation Diagram between risk score and ICI related genes: PDCD1LG2, PDCD1, HAVCR2, CTLA4, CD274 and IDO1. (**F**) Comparison of all crucial immune checkpoint inhibitors related genes in two groups.

We subsequently explored the following six hub immune checkpoint inhibitors related genes: PDCD1LG2, PDCD1, HAVCR2, CTLA4, CD274, and IDO1 [25-27]. The results expressed that the signature we constructed was significantly negatively correlated with the levels of CD274 and IDO1, implying that our model served as a vital function in asses of ICI treatment response in COAD (Fig. **6A** and **6B**). In addition, TIDE showed that COAD patients with high-risk scores had lower microsatellite instability (MSI) and stronger immune exclusion, further indicating that our risk model could perfectly predict the responsiveness to ICI therapy (Fig. **6C** and **6D**). We analyzed the correlation between our pyroptosis-related lncRNA model and immune checkpoint inhibitors (ICI) related targets to identify the potential functions of our model on the immunotherapy of COAD patients (Fig. **6E**). By and last, we explored and analyzed all crucial immune checkpoint inhibitors-related genes in high- and low-risk groups, and some of them (*i.e*., CTLA4, TNFRSF9, ICOS, *etc*.) were markedly differentially expressed (Fig. **6F**).

### 3.7 The Clinical Significance of ZKSCN2-DT *in vitro* and COAD Study

**Fig. (7):**
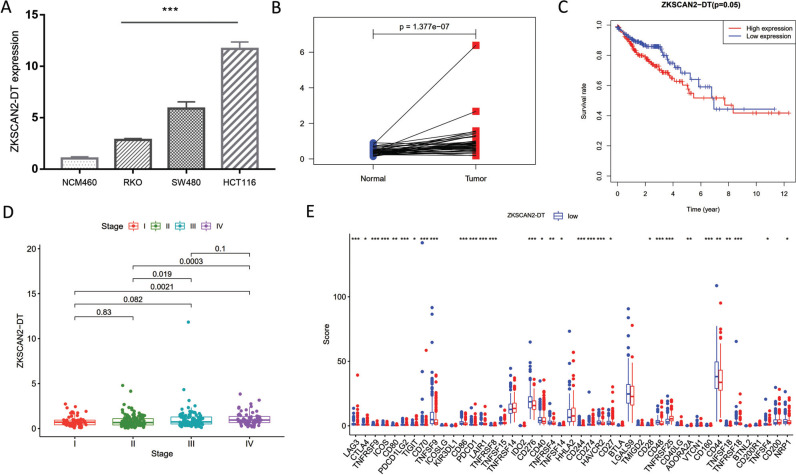
The clinical significance of ZKSCN2-DT *in vitro* and COAD study. (**A**) ZKSCAN2-DT was overexpressed in three COAD cell lines and (**B**) TGCA-COAD cohort. (**C**) Higher ZKSCAN2-DT expression possessed poor prognosis. (**D**) The ZKSCAN2-DT expression was significantly different in different stage. (**E**) The correlation between ZKSCAN2-DT and 49 ICI-related genes.

ZKSCAN2-DT level was one of the most overexpressed of seven prognostic pyroptosis-related lncRNAs in COAD. Herein, we further explored the ZKSCAN2-DT expression in COAD using Q-PCR. We analyzed the ZKSCAN2-DT expression in three COAD cell lines (SW480, RKO, and HCT116 cells) and normal human colonic epithelial cells (NCM460). Fig. (**7A**) presented that ZKSCAN2-DT was significantly overexpressed in the COAD cell line compared with normal human colonic epithelial cells. Furthermore, paired analysis was employed, and the results showed that ZKSCAN2-DT expression was significantly upregulated in COAD tissues (Fig. **7B**). To evaluate the latent role of ZKSCAN2-DT in COAD, the expression matrix of TCGA-COAD was employed for analysis. As shown in Fig. (**7C**), patients with higher ZKSCAN2-DT had a worse survival prognosis. Combined with the pathological stage, we could find that ZKSCAN2-DT expression was markedly differentially expressed in different pathological stages. That was, the worse the pathological stage, the higher the ZKSCAN2-DT expression (Fig. **7D**). Finally, the correlation between ZKSCAN2-DT and 49 ICI-related genes was analyzed. The finding presented that the higher ZKSCAN2-DT expression possessed a stronger level of ICI-related genes, suggesting that ZKSCAN2-DT might play an important role in COAD immunotherapy (Fig. **7E**).

### 3.8 Role of ZKSCN2-DT in Immune Status and TME

**Fig. (8):**
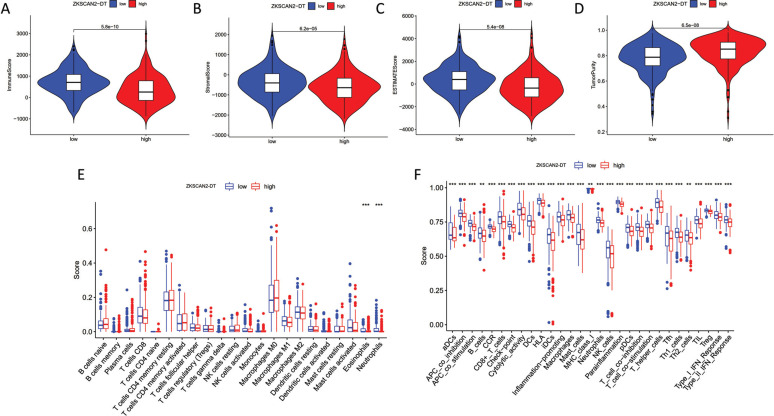
Role of ZKSCAN2-DT in immune status and TME. Association between ZKSCAN2-DT expression and immune status of Immune score (**A**). Association between ZKSCAN2-DT expression and immune status of Stromal score (**B**). Association between ZKSCAN2-DT expression and immune status of ESTIMATE score (**C**). Association between ZKSCAN2-DT expression and immune status of Tumor purity (**D**). CIBERSORT results revealed that some immune cells showed a significantly higher level in low-ZKSCAN2-DT subtype, including T cells, B cells, as well as M1 macrophages (**E**). ssGSEA analysis results shown that the immune infiltration fraction of most immune cells decreased significantly with the increase of ZKSCAN2-DT expression in TCGA-COAD (**F**).

To clarify the relationship between ZKSCAN2-DT and TME characteristics in COAD, patients were further divided into the high ZKSCAN2-DT subgroup and the low ZKSCAN2-DT subgroup based on the median expression of ZKSCAN2-DT. ESTIMATE results showed that the subgroup with lower ZKSCAN2-DT expression had significantly higher stromal score, immune score, along with ESTIMATE score compared with the high-ZKSCAN2-DT group, indicating more immune cells and stroma cells in the low expression subgroup (Fig. **8A**-**8D**). CIBERSORT results revealed that B cells, T cells, and M1 macrophages showed a significantly elevated level in the low-ZKSCAN2-DT subtype (Fig. **8E**). ssGSEA analysis results showed that the immune infiltration fraction of most immune cells (*i.e*., APC co-inhibition, B cells, CD^8+^Tcells, iDCs, Th1, Type-1 IFN Response, *etc*.) decreased significantly with the increase of ZKSCAN2-DT expression in TCGA-COAD (Fig. **8F**). We used GSE166555 of the TISCH database to evaluate pyroptosis-related lncRNA expression in tumor immune cells. As shown in Fig. (**9**), the relatively higher expression level of AL161729.4 was observed in malignant cells, suggesting high AL161729.4 expression in malignant colorectal cancer cells. LINC02381 showed higher expression levels in fibroblasts, and AC007128.1 showed less expression in malignant colorectal cancer cells.

## 4 DISCUSSION

Colorectal cancer is a genetically heterogeneous disease involving many different molecular pathways and mechanisms [34]. Previous studies have indicated that the effective population of colorectal cancer immunotherapy only accounted for about 5% [16]. Therefore, the immunotherapy of colon cancer still needs more investigations to benefit more patients. Pyroptosis and various components involved in pyroptosis are closely associated with tumor development and anti-tumor immunity [35]. With augmenting research, lncRNA can serve as a biomarker, predicting cancer diagnosis and survival status. Besides, pyroptosis-related lncRNAs and their regulatory mechanisms involving the pyroptosis pathway are increasingly clarified [21]. However, comprehensive studies about pyroptosis-associated lncRNA models with the ability of predicting COAD prognosis are still lacking. Therefore, it is urgent and essential to construct a pyroptosis-related lncRNA signature of COAD patients according to the bioinformatics and large-scale databases.

Our signature, which was constructed from seven pyroptosis-related lncRNAs, could considerably predict how COAD patients will fare. According to earlier studies, the low-risk group had superior OS than the others. The areas under the ROC curve for survival at one year, three years, and five years were 0.647, 0.715, and 0.714, respectively. This evidence showed that our risk signature had some promise for predicting COAD survival status. The results of the ROC curve, C-index, and Calibration curve, as well as univariate and multivariate Cox analysis, further confirmed that our model might function as a possible independent prognostic indicator.

In our research, lncRNAs associated with pyroptosis were found by creating a network of genes and lncRNAs that co-express each other. In addition, Lasso regression along with multivariate Cox regression were applied to identify predictive values of the following seven pyroptosis-related lncRNAs: ZKSCAN2-DT, AC007128.1, AL161729.4, LINC02381, AC016394.3, and AL137782.1. The seven pyroptosis-related lncRNAs could serve as therapeutic targets for COAD patients and prognostic molecular markers of prognosis. Three lncRNAs related to pyroptosis (AC007128.1, AC099850.4, and LINC02381) have been linked to malignancies. It is the first study to confirm the predictive usefulness of the remaining four pyroptosis-related lncRNAs (ZKSCAN2-DT, AL161729.4, AC016394.3, and AL137782.1). Therefore, additional research is required to understand the precise processes of these lncRNAs in COAD.

Zinc finger and SCAN domain-containing (ZSCAN) transcription factors demonstrated promotive or inhibitory effects on tumor growth as a subfamily of zinc finger transcription factors [36]. Circular ZKSCAN1, for instance, was described as a tumor suppressor that significantly influenced the proliferative, migratory, and invading capacities of bladder cancer cells [37]. It has not been documented how ZKSCAN2-DT and colon cancer are related.

Several limitations still existed in our study. First, our study is a large-sample retrospective study based on public databases that may have some inherent biases. Besides, there is a deficiency of several potentially important parameters related to prognosis, including the family history of colon cancer, the surgical margin status and the treatment situation. The left-sided and right-sided colon cancer differ greatly in their carcinogenic mechanisms, clinical manifestations, and response to treatment due to their different embryonic origins and different anatomical physiology [38]. Moreover, the location of the primary cancer has been recognized as an important prognostic and predictive factor. Due to database limitations, these factors are not included in the prognostic model. Third, our nomogram was internally and externally validated based on the TCGA database, and it is important to evaluate it by external validation using other satisfactory populations.

Regarding future research directions, we have outlined a plan to investigate the functional relevance of the pyroptosis-related lncRNAs we have identified with respect to colorectal cancer progression. Our goal is to gain a deeper understanding of their molecular mechanisms and explore their potential as therapeutic targets. Furthermore, we plan to conduct large-scale clinical studies in order to assess the prognostic significance of the established profiles and explore how they may be applied in personalized medicine approaches. We believe that these avenues of inquiry will lead to valuable insights in the field of colorectal cancer research.

## CONCLUSION

Collectively, our study developed a prognostic prediction model and first discovered the pyroptosis-related lncRNAs linked to the prognosis of COAD. Additionally, this study discovered that the pyroptosis-related risk score, which is made up of seven pyroptosis-related lncRNAs, is associated to TILs and the expression of immune checkpoint molecules in addition to being utilized to distinguish patients at different risk levels. Therefore, promoting pyroptosis may be a possible treatment to increase the efficacy of immunotherapy in COAD, and the seven pyroptosis-associated lncRNAs may have the potential to be molecular biomarkers for patients with colon adenocarcinoma.

## Figures and Tables

**Fig. (9):**
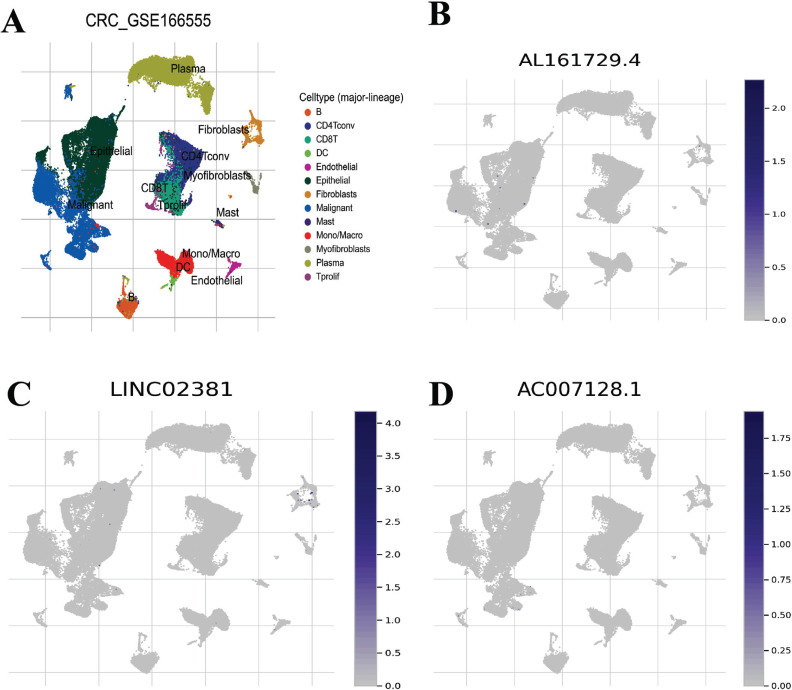
The cell type distribution of pyroptosis-related lncRNA using scRNA seq database. The cell types and their distribution in GSE166555 (**A**). Distribution of AL161729.4 in different cells in GSE166555 (**B**). Distribution of LINC02381 in different cells in GSE166555 (**C**). Distribution of AC007128.1 in different cells in GSE166555 (**D**).

**Table 1:** Basic clinical information of all 452 COAD patients from TCGA cohort.

**Variables**	**TCGA Cohorts** **(n=452)**
Age	67.09±13.00
**Gender**	
Female Male	214 (47.3%) 238 (52.7%)
**Pathologic Stage**	
I & II III & IV Unknow	254 (56.2%) 187 (41.4%) 11 (2.4%)
**AJCC-T**	
T1 T2 T3 T4	11 (2.4%) 77 (17.0%) 308 (68.1%) 56 (12.4%)
**AJCC-N**	
N0 N1-N3	372 (82.3%) 80 (17.7%)
**AJCC-M**	
M0 M1 Unknow	334 (73.9%) 62 (13.7%) 56 (12.4%)

**Table 2:** The results of 7 pyroptosis-related lncRNAs in the multivariate Cox regression analysis.

**LncRNAs**	**HR (95% CI)**	***P*-value**	**Coef**
AC007128.1	1.535 (1.019-2.283)	0.039	0.422
ZKSCAN2-DT	1.293 (1.019-1.641)	0.033	0.257
AC099850.4	0.925 (0.876-0.977)	0.005	-0.077
AL161729.4	1.344 (1.021-1.769)	0.034	0.295
LINC02381	1.246 (1.057-1.468)	0.008	0.220
AC016394.3	1.219 (1.031-1.442)	0.020	0.198
AL137782.1	0.373 (0.193-0.720)	0.003	-0.983

**Note:** HR = hazard ratio; Coef = regression coefficient; 95% CI = 95% confidence interval.

## Data Availability

All the data and supporting information is provided within the article.

## LIST OF ABBREVIATIONS

CRC: Colorectal Cancer
COAD: Colon Adenocarcinoma
TIDE: Tumor Immune Dysfunction and Exclusion
qPCR: Quantitative Real-time Polymerase Chain Reaction
TISCH: Tumor Immune Single Cell Hub Database

## References

[1] Bray F., Ferlay J., Soerjomataram I., Siegel R.L., Torre L.A., Jemal A. (2018). Global cancer statistics 2018: GLOBOCAN estimates of incidence and mortality worldwide for 36 cancers in 185 countries.. CA Cancer J. Clin..

[2] Siegel R.L., Miller K.D., Goding Sauer A., Fedewa S.A., Butterly L.F., Anderson J.C., Cercek A., Smith R.A., Jemal A. (2020). Colorectal cancer statistics, 2020.. CA Cancer J. Clin..

[3] Baidoun F., Elshiwy K., Elkeraie Y., Merjaneh Z., Khoudari G., Sarmini M.T., Gad M., Al-Husseini M., Saad A. (2021). Colorectal cancer epidemiology: Recent trends and impact on outcomes.. Curr. Drug Targets.

[4] Villariba-Tolentino C., Cariño A.M., Notarte K.I., Macaranas I., Fellizar A., Tomas R.C., Angeles L.M., Abanilla L., Lim A., Aguilar M.K.C., Albano P.M. (2021). pks+ Escherichia coli more prevalent in benign than malignant colorectal tumors.. Mol. Biol. Rep..

[5] Wang J., Li S., Liu Y., Zhang C., Li H., Lai B. (2020). Metastatic patterns and survival outcomes in patients with stage IV colon cancer: A population‐based analysis.. Cancer Med..

[6] Shi J., Gao W., Shao F. (2017). Pyroptosis: Gasdermin-mediated programmed necrotic cell death.. Trends Biochem. Sci..

[7] Liu X., Xia S., Zhang Z., Wu H., Lieberman J. (2021). Channelling inflammation: Gasdermins in physiology and disease.. Nat. Rev. Drug Discov..

[8] Broz P., Pelegrín P., Shao F. (2020). The gasdermins, a protein family executing cell death and inflammation.. Nat. Rev. Immunol..

[9] Miguchi M., Hinoi T., Shimomura M., Adachi T., Saito Y., Niitsu H., Kochi M., Sada H., Sotomaru Y., Ikenoue T., Shigeyasu K., Tanakaya K., Kitadai Y., Sentani K., Oue N., Yasui W., Ohdan H., Gasdermin C. (2016). Gasdermin C is upregulated by inactivation of transforming growth factor β receptor type II in the presence of mutated Apc, promoting colorectal cancer proliferation.. PLoS One.

[10] Kim M.S., Chang X., Yamashita K., Nagpal J.K., Baek J.H., Wu G., Trink B., Ratovitski E.A., Mori M., Sidransky D. (2008). Aberrant promoter methylation and tumor suppressive activity of the DFNA5 gene in colorectal carcinoma.. Oncogene.

[11] Tan G., Huang C., Chen J., Zhi F. (2020). HMGB1 released from GSDME-mediated pyroptotic epithelial cells participates in the] tumorigenesis of colitis-associated colorectal cancer through the ERK1/2 pathway.. J. Hematol. Oncol..

[12] Zhang Z., Zhang Y., Xia S., Kong Q., Li S., Liu X., Junqueira C., Meza-Sosa K.F., Mok T.M.Y., Ansara J., Sengupta S., Yao Y., Wu H., Lieberman J. (2020). Gasdermin E suppresses tumour growth by activating anti-tumour immunity.. Nature.

[13] Hou J., Hsu J.M., Hung M.C. (2021). Molecular mechanisms and functions of pyroptosis in inflammation and antitumor immunity.. Mol. Cell.

[14] Wang Y., Gao W., Shi X., Ding J., Liu W., He H., Wang K., Shao F. (2017). Chemotherapy drugs induce pyroptosis through caspase-3 cleavage of a gasdermin.. Nature.

[15] Zhang X., Zhang H. (2018). Chemotherapy drugs induce pyroptosis through caspase-3-dependent cleavage of GSDME.. Sci. China Life Sci..

[16] Ganesh K., Stadler Z.K., Cercek A., Mendelsohn R.B., Shia J., Segal N.H., Diaz L.A. (2019). Immunotherapy in colorectal cancer: Rationale, challenges and potential.. Nat. Rev. Gastroenterol. Hepatol..

[17] Mercer T.R., Dinger M.E., Mattick J.S. (2009). Long non-coding RNAs: Insights into functions.. Nat. Rev. Genet..

[18] Zhang G., Sun J., Zhang X. (2022). A novel Cuproptosis-related LncRNA signature to predict prognosis in hepatocellular carcinoma.. Sci. Rep..

[19] Notarte K.I., Senanayake S., Macaranas I., Albano P.M., Mundo L., Fennell E., Leoncini L., Murray P. (2021). MicroRNA and other non-coding RNAs in epstein–barr virus-associated cancers.. Cancers..

[20] Denaro N., Merlano M.C., Lo Nigro C. (2019). Long noncoding RNA s as regulators of cancer immunity.. Mol. Oncol..

[21] He D., Zheng J., Hu J., Chen J., Wei X. (2020). Long non-coding RNAs and pyroptosis.. Clin. Chim. Acta.

[22] Zhang X., Sun S., Pu J.K.S., Tsang A.C.O., Lee D., Man V.O.Y., Lui W.M., Wong S.T.S., Leung G.K.K. (2012). Long non-coding RNA expression profiles predict clinical phenotypes in glioma.. Neurobiol. Dis..

[23] Wu J., Zhu Y., Luo M., Li L. (2021). Comprehensive analysis of pyroptosis-related genes and tumor microenvironment infiltration characterization in breast cancer.. Front. Immunol..

[24] Lossos I.S., Czerwinski D.K., Alizadeh A.A., Wechser M.A., Tibshirani R., Botstein D., Levy R. (2004). Prediction of survival in diffuse large-B-cell lymphoma based on the expression of six genes.. N. Engl. J. Med..

[25] Blanche P., Dartigues J.F., Jacqmin-Gadda H. (2013). Estimating and comparing time-dependent areas under receiver operating characteristic curves for censored event times with competing risks.. Stat. Med..

[26] Newman A.M., Liu C.L., Green M.R., Gentles A.J., Feng W., Xu Y., Hoang C.D., Diehn M., Alizadeh A.A. (2015). Robust enumeration of cell subsets from tissue expression profiles.. Nat. Methods.

[27] Xiao B., Liu L., Li A., Xiang C., Wang P., Li H., Xiao T. (2020). Identification and verification of immune-related gene prognostic signature based on ssGSEA for osteosarcoma.. Front. Oncol..

[28] Cao R., Yuan L., Ma B., Wang G., Tian Y. (2021). Tumour microenvironment (TME) characterization identified prognosis and immunotherapy response in muscle-invasive bladder cancer (MIBC).. Cancer Immunol. Immunother..

[29] Goodman A., Patel S.P., Kurzrock R. (2017). PD-1–PD-L1 immune-checkpoint blockade in B-cell lymphomas.. Nat. Rev. Clin. Oncol..

[30] Nishino M., Ramaiya N.H., Hatabu H., Hodi F.S. (2017). Monitoring immune-checkpoint blockade: Response evaluation and biomarker development.. Nat. Rev. Clin. Oncol..

[31] Zhai L., Ladomersky E., Lenzen A., Nguyen B., Patel R., Lauing K.L., Wu M., Wainwright D.A. (2018). IDO1 in cancer: A Gemini of immune checkpoints.. Cell. Mol. Immunol..

[32] Kim J.E., Patel M.A., Mangraviti A., Kim E.S., Theodros D., Velarde E., Liu A., Sankey E.W., Tam A., Xu H., Mathios D., Jackson C.M., Harris-Bookman S., Garzon-Muvdi T., Sheu M., Martin A.M., Tyler B.M., Tran P.T., Ye X., Olivi A., Taube J.M., Burger P.C., Drake C.G., Brem H., Pardoll D.M., Lim M. (2017). Combination therapy with Anti-PD-1, Anti-TIM-3, and focal radiation results in regression of murine gliomas.. Clin. Cancer Res..

[33] Sun D., Wang J., Han Y., Dong X., Ge J., Zheng R., Shi X., Wang B., Li Z., Ren P., Sun L., Yan Y., Zhang P., Zhang F., Li T., Wang C. (2021). TISCH: A comprehensive web resource enabling interactive single-cell transcriptome visualization of tumor microenvironment.. Nucleic Acids Res..

[34] Raskov H., Søby J.H., Troelsen J., Bojesen R.D., Gögenur I. (2020). Driver gene mutations and epigenetics in colorectal cancer.. Ann. Surg..

[35] Tang R., Xu J., Zhang B., Liu J., Liang C., Hua J., Meng Q., Yu X., Shi S. (2020). Ferroptosis, necroptosis, and pyroptosis in anticancer immunity.. J. Hematol. Oncol..

[36] Huang M., Chen Y., Han D., Lei Z., Chu X. (2019). Role of the zinc finger and SCAN domain-containing transcription factors in cancer.. Am. J. Cancer Res..

[37] Bi J., Liu H., Dong W., Xie W., He Q., Cai Z., Huang J., Lin T. (2020). Correction to: Circular RNA circ-ZKSCAN1 inhibits bladder cancer progression through miR-1178-3p/p21 axis and acts as a prognostic factor of recurrence.. Mol. Cancer.

[38] Benedix F., Kube R., Meyer F., Schmidt U., Gastinger I., Lippert H. (2010). Colon/rectum carcinomas study, comparison of 17,641 patients with right- and left-sided colon cancer: Differences in epidemiology, perioperative course, histology, and survival.. Dis. Colon Rectum.

