# Supplementary material for: Machine Learning-derived Multi-omics Prognostic Signature of Pyroptosis-related lncRNA with Regard to ZKSCAN2-DT and Tumor Immune Infiltration in Colorectal Cancer
Source: Comb Chem High Throughput Screen. 2023 Sep 14;27(8):1161–74. doi: 10.2174/1386207326666230823104952 (PMC11327744; doi:10.2174/1386207326666230823104952)
Supplement: Supplementary file 1 [file CCHTS-27-1161_SD1.pdf]

Supplementary Material

Machine Learning-derived Multi-omics Prognostic Signature of Pyroptosis-related lncRNA with Regard to ZKSCAN2-DT and Tumor Immune Infiltration in Colorectal Cancer

Jiamin Chen[1,#], Dan Jin[1,#], Liming Shao[1,#], Lingling Wang[2], Liuzhi Zhou[3,*] and Jianting Cai[1,*]

[1]Department of Gastroenterology, The Second Affiliated Hospital, Zhejiang University School of Medicine, Hangzhou 310009, Zhejiang Province, China; [2]Institute of Immunology, Zhejiang University School of Medicine, Hangzhou 310009, China; [3]Department of Surgical Oncology, Sir Run Run Shaw Hospital, Zhejiang University, Hangzhou 310000, Zhejiang Province, China

Table S1.

BAK1
BAX
CASP1
CASP3
CASP4
CASP5
CHMP2A
CHMP2B
CHMP3
CHMP4A
CHMP4B
CHMP4C
CHMP6
CHMP7
CYCS
ELANE
GSDMD
GSDME
GZMB
HMGB1
IL18
IL1A
IL1B
IRF1

IRF2
TP53
TP63
AIM2
CASP6
CASP8
CASP9
GPX4
GSDMA
GSDMB
GSDMC
IL6
NLRC4
NLRP1
NLRP2
NLRP3
NLRP6
NLRP7
NOD1
NOD2
PJVK
PLCG1
PRKACA
PYCARD
SCAF11
TIRAP
TNF
GZMA

Table S2.

Pyroptosis-related Genes	lncRNA	cor	pvalue	Regulation
GSDME	AC245060.5	0.402754986	7.14E-20	postive
PJVK	AC245060.5	0.474296677	6.64E-28	postive
SCAF11	AC245060.5	0.572354755	1.64E-42	postive
GSDME	LINC02156	0.472215268	1.21E-27	postive
NLRC4	LINC02156	0.420525299	1.08E-21	postive
PJVK	LINC02156	0.470250243	2.13E-27	postive
SCAF11	LINC02156	0.659834906	1.92E-60	postive

Pyroptosis-related Genes	lncRNA	cor	pvalue	Regulation
CHMP2A	AL731566.2	-0.491457094	3.95E-30	negative
CHMP6	AL731566.2	-0.429350015	1.23E-22	negative
GSDME	AL731566.2	0.436136991	2.20E-23	postive
NLRC4	AL731566.2	0.403587386	5.90E-20	postive
NOD1	AL731566.2	0.417442382	2.28E-21	postive
PJVK	AL731566.2	0.40185659	8.77E-20	postive
SCAF11	AL731566.2	0.702663828	1.25E-71	postive
SCAF11	LINC01290	0.413863528	5.35E-21	postive
GSDME	AC023510.2	0.458453583	5.87E-26	postive
NLRC4	AC023510.2	0.460246102	3.58E-26	postive
PJVK	AC023510.2	0.420319008	1.14E-21	postive
SCAF11	AC023510.2	0.616655954	6.89E-51	postive
NLRC4	C9orf139	0.412084438	8.16E-21	postive
NLRP1	C9orf139	0.424036307	4.58E-22	postive
PLCG1	LINC00174	0.400387027	1.22E-19	postive
PJVK	AL158835.2	0.481593416	7.78E-29	postive
SCAF11	AGAP1-IT1	0.445157091	2.11E-24	postive
GSDME	AL138921.1	0.426010254	2.82E-22	postive
PJVK	AL138921.1	0.542620999	1.44E-37	postive
SCAF11	AL138921.1	0.549813128	1.02E-38	postive
GSDME	AL157392.4	0.523832174	1.09E-34	postive
NLRC4	AL157392.4	0.469232903	2.85E-27	postive
PJVK	AL157392.4	0.452720541	2.81E-25	postive
SCAF11	AL157392.4	0.640143334	6.77E-56	postive
CHMP6	AC107027.3	-0.410213002	1.27E-20	negative
CASP8	AC107027.3	0.440040941	8.05E-24	postive
PJVK	AC107027.3	0.452272912	3.17E-25	postive
SCAF11	AC107027.3	0.680045062	1.75E-65	postive
GSDME	AL031651.2	0.57454243	6.75E-43	postive
IL1B	AL031651.2	0.446923802	1.32E-24	postive
NLRC4	AL031651.2	0.555735843	1.09E-39	postive
SCAF11	AL031651.2	0.521618377	2.32E-34	postive
PJVK	AL021707.2	0.553829608	2.25E-39	postive
SCAF11	AL021707.2	0.480272409	1.15E-28	postive
GSDME	LUCAT1	0.569690617	4.76E-42	postive
IL1A	LUCAT1	0.624227296	1.86E-52	postive
IL1B	LUCAT1	0.80405669	2.01E-108	postive
NLRC4	LUCAT1	0.499153711	3.60E-31	postive

Pyroptosis-related Genes	lncRNA	cor	pvalue	Regulation
NLRP3	LUCAT1	0.606450062	7.67E-49	postive
SCAF11	LUCAT1	0.452158364	3.27E-25	postive
PJVK	CYP4A22-AS1	0.557308814	6.00E-40	postive
PJVK	CEP83-DT	0.508531129	1.79E-32	postive
PLCG1	STAG3L5P-PVRIG2P-PILRB	0.47677843	3.22E-28	postive
PJVK	AC026367.2	0.485073061	2.75E-29	postive
NOD1	AC008543.1	0.432436896	5.64E-23	postive
SCAF11	AC008543.1	0.644026544	9.13E-57	postive
SCAF11	LINC00513	0.501054952	1.97E-31	postive
BAK1	NORAD	-0.411835774	8.65E-21	negative
CHMP3	NORAD	0.424890665	3.71E-22	postive
NOD1	NORAD	0.480815313	9.81E-29	postive
NOD2	NORAD	0.407068241	2.64E-20	postive
PJVK	NORAD	0.435645059	2.50E-23	postive
PLCG1	NORAD	0.519566843	4.64E-34	postive
SCAF11	NORAD	0.504765443	6.04E-32	postive
PJVK	AC079907.1	0.513687255	3.30E-33	postive
SCAF11	AC079907.1	0.603992828	2.33E-48	postive
GSDME	AC004584.1	0.431810349	6.61E-23	postive
PJVK	AC004584.1	0.459933996	3.90E-26	postive
SCAF11	AC004584.1	0.553347103	2.70E-39	postive
CHMP4A	AC009065.9	0.408933088	1.71E-20	postive
GSDME	AC010542.6	0.464074067	1.23E-26	postive
IL1B	AC010542.6	0.404562536	4.71E-20	postive
NLRC4	AC010542.6	0.445814045	1.78E-24	postive
SCAF11	AC010542.6	0.592602422	3.52E-46	postive
GSDME	KIF26B-AS1	0.550155737	8.96E-39	postive
IL1A	KIF26B-AS1	0.465318116	8.66E-27	postive
IL1B	KIF26B-AS1	0.499595664	3.13E-31	postive
NLRC4	KIF26B-AS1	0.435472159	2.61E-23	postive
NLRP3	KIF26B-AS1	0.444903562	2.26E-24	postive
SCAF11	KIF26B-AS1	0.531826956	6.83E-36	postive
ELANE	AL161785.1	0.433192005	4.66E-23	postive
GSDME	AL161785.1	0.535145101	2.12E-36	postive
NLRC4	AL161785.1	0.623436044	2.73E-52	postive
NLRP1	AL161785.1	0.416957446	2.56E-21	postive
NLRP3	AL161785.1	0.45540377	1.35E-25	postive
IRF1	AF124730.2	0.430138853	1.01E-22	postive

Pyroptosis-related Genes	lncRNA	cor	pvalue	Regulation
AIM2	AF124730.2	0.407350469	2.47E-20	postive
GSDME	WDFY3-AS2	0.470295161	2.11E-27	postive
PJVK	WDFY3-AS2	0.46914368	2.93E-27	postive
SCAF11	WDFY3-AS2	0.490864638	4.74E-30	postive
GSDME	Z99289.2	0.578156389	1.54E-43	postive
IL1A	Z99289.2	0.470973752	1.73E-27	postive
IL1B	Z99289.2	0.4989199	3.88E-31	postive
NLRC4	Z99289.2	0.472876795	1.00E-27	postive
NLRP3	Z99289.2	0.408882129	1.73E-20	postive
PJVK	Z99289.2	0.404989992	4.27E-20	postive
SCAF11	Z99289.2	0.612650324	4.47E-50	postive
PJVK	ZNF674-AS1	0.409309256	1.57E-20	postive
CHMP2A	C1RL-AS1	-0.416790752	2.66E-21	negative
GSDME	C1RL-AS1	0.45265526	2.86E-25	postive
NLRC4	C1RL-AS1	0.439083441	1.03E-23	postive
NOD1	C1RL-AS1	0.492818757	2.60E-30	postive
SCAF11	C1RL-AS1	0.661882196	6.17E-61	postive
CHMP2A	AC244093.5	-0.408208616	2.03E-20	negative
GSDME	AC244093.5	0.49104057	4.49E-30	postive
NLRC4	AC244093.5	0.437885834	1.41E-23	postive
PJVK	AC244093.5	0.411204966	1.00E-20	postive
SCAF11	AC244093.5	0.641284308	3.77E-56	postive
PLCG1	AL118506.1	0.556967051	6.84E-40	postive
CHMP2A	THAP9-AS1	-0.436920862	1.80E-23	negative
CHMP6	THAP9-AS1	-0.428794722	1.41E-22	negative
CASP8	THAP9-AS1	0.462738777	1.79E-26	postive
PJVK	THAP9-AS1	0.416638412	2.76E-21	postive
SCAF11	THAP9-AS1	0.544987429	6.07E-38	postive
GSDME	AL158071.1	0.533641237	3.61E-36	postive
NLRC4	AL158071.1	0.425662018	3.07E-22	postive
PJVK	AL158071.1	0.470980969	1.73E-27	postive
SCAF11	AL158071.1	0.622803009	3.71E-52	postive
GSDME	CFAP44-AS1	0.567456883	1.16E-41	postive
NLRC4	CFAP44-AS1	0.507051855	2.89E-32	postive
PJVK	CFAP44-AS1	0.409627151	1.45E-20	postive
SCAF11	CFAP44-AS1	0.671243818	3.07E-63	postive
CHMP6	AC074032.1	-0.407309597	2.50E-20	negative
GSDME	AC074032.1	0.471105527	1.67E-27	postive

Pyroptosis-related Genes	lncRNA	cor	pvalue	Regulation
NLRC4	AC074032.1	0.406794937	2.81E-20	postive
SCAF11	AC074032.1	0.676862452	1.16E-64	postive
GSDME	MESTIT1	0.44423267	2.70E-24	postive
PJVK	MESTIT1	0.438014696	1.36E-23	postive
SCAF11	MESTIT1	0.665533632	7.97E-62	postive
CHMP2A	AC125257.1	-0.428119171	1.67E-22	negative
CHMP2B	AC125257.1	0.428336327	1.58E-22	postive
CASP8	AC125257.1	0.458333952	6.06E-26	postive
PJVK	AC125257.1	0.485651162	2.31E-29	postive
SCAF11	AC125257.1	0.657923679	5.49E-60	postive
PJVK	AC135050.3	0.462706517	1.80E-26	postive
GSDME	Z82243.1	0.43697924	1.77E-23	postive
NOD1	Z82243.1	0.417270953	2.37E-21	postive
SCAF11	Z82243.1	0.589278137	1.47E-45	postive
CHMP2A	AL132989.2	-0.439751528	8.68E-24	negative
CHMP6	AL132989.2	-0.411679935	8.98E-21	negative
GSDME	AL132989.2	0.577918786	1.70E-43	postive
IL1A	AL132989.2	0.476287003	3.72E-28	postive
IL1B	AL132989.2	0.509694734	1.23E-32	postive
NLRC4	AL132989.2	0.522407251	1.77E-34	postive
NLRP3	AL132989.2	0.443978508	2.88E-24	postive
SCAF11	AL132989.2	0.752154434	2.39E-87	postive
SCAF11	AC106791.1	0.463938071	1.28E-26	postive
CHMP4A	VASH1-AS1	0.42578088	2.98E-22	postive
GSDME	LINC02649	0.559603114	2.49E-40	postive
IL1A	LINC02649	0.512237289	5.33E-33	postive
IL1B	LINC02649	0.641761031	2.95E-56	postive
NLRC4	LINC02649	0.474004505	7.23E-28	postive
NLRP3	LINC02649	0.546213386	3.87E-38	postive
SCAF11	LINC02649	0.494157511	1.72E-30	postive
GPX4	SNHG9	0.459247156	4.71E-26	postive
PYCARD	SNHG9	0.47834919	2.03E-28	postive
SCAF11	AC080188.1	0.469295636	2.80E-27	postive
GSDME	AL110115.1	0.55271832	3.43E-39	postive
NLRC4	AL110115.1	0.466338392	6.49E-27	postive
NOD1	AL110115.1	0.430553853	9.07E-23	postive
SCAF11	AL110115.1	0.665484524	8.20E-62	postive
GSDME	AC010999.2	0.486839314	1.61E-29	postive

Pyroptosis-related Genes	lncRNA	cor	pvalue	Regulation
IL1A	AC010999.2	0.446924967	1.32E-24	postive
IL1B	AC010999.2	0.532921813	4.65E-36	postive
NLRC4	AC010999.2	0.401215124	1.01E-19	postive
SCAF11	AC010999.2	0.454421385	1.77E-25	postive
PJVK	AC125494.2	0.401592314	9.31E-20	postive
GSDME	AC068790.2	0.423413215	5.34E-22	postive
PJVK	AC068790.2	0.431588517	6.99E-23	postive
SCAF11	AC068790.2	0.666891205	3.70E-62	postive
SCAF11	AC103858.1	0.493216458	2.30E-30	postive
CHMP2A	PLCG1-AS1	-0.405374912	3.91E-20	negative
GSDME	PLCG1-AS1	0.585539149	7.16E-45	postive
IL1A	PLCG1-AS1	0.453815001	2.09E-25	postive
IL1B	PLCG1-AS1	0.507511198	2.49E-32	postive
NLRC4	PLCG1-AS1	0.520603036	3.27E-34	postive
NLRP3	PLCG1-AS1	0.406142661	3.27E-20	postive
PJVK	PLCG1-AS1	0.417808691	2.08E-21	postive
SCAF11	PLCG1-AS1	0.719329798	1.53E-76	postive
GSDME	IRF1-AS1	0.418959662	1.58E-21	postive
SCAF11	IRF1-AS1	0.50956854	1.28E-32	postive
CHMP4A	AL137779.2	0.406104222	3.30E-20	postive
GSDME	AL137779.2	0.538175841	7.18E-37	postive
IL1A	AL137779.2	0.425918456	2.88E-22	postive
IL1B	AL137779.2	0.481852525	7.21E-29	postive
NLRC4	AL137779.2	0.430270029	9.74E-23	postive
NLRP3	AL137779.2	0.506593385	3.36E-32	postive
SCAF11	AL137779.2	0.495166704	1.25E-30	postive
GSDME	AC006460.1	0.580186997	6.69E-44	postive
IL1A	AC006460.1	0.47217125	1.23E-27	postive
IL1B	AC006460.1	0.540690556	2.90E-37	postive
NLRC4	AC006460.1	0.5276175	2.97E-35	postive
SCAF11	AC006460.1	0.646632137	2.34E-57	postive
NOD1	PAN3-AS1	0.43373313	4.06E-23	postive
PJVK	PAN3-AS1	0.40129096	9.97E-20	postive
PJVK	AC024361.1	0.412132587	8.07E-21	postive
SCAF11	AC024361.1	0.468820457	3.21E-27	postive
SCAF11	C3orf35	0.536407235	1.35E-36	postive
CHMP4B	LINC02747	0.414093786	5.07E-21	postive
CHMP2A	AC109587.1	-0.461260709	2.70E-26	negative

Pyroptosis-related Genes	lncRNA	cor	pvalue	Regulation
CHMP6	AC109587.1	-0.433088016	4.79E-23	negative
GSDME	AC109587.1	0.575693195	4.23E-43	postive
IL1A	AC109587.1	0.48493033	2.87E-29	postive
IL1B	AC109587.1	0.5059595	4.12E-32	postive
NLRC4	AC109587.1	0.503613047	8.74E-32	postive
NLRP3	AC109587.1	0.469196336	2.88E-27	postive
PJVK	AC109587.1	0.45475229	1.62E-25	postive
SCAF11	AC109587.1	0.768769382	1.73E-93	postive
CHMP4A	U73166.1	0.447393579	1.17E-24	postive
PJVK	U73166.1	0.483680096	4.18E-29	postive
CHMP6	AC018809.1	-0.414750228	4.34E-21	negative
SCAF11	AC018809.1	0.562694618	7.50E-41	postive
CHMP2A	AC008937.3	-0.434357438	3.47E-23	negative
CHMP6	AC008937.3	-0.41082879	1.10E-20	negative
GSDME	AC008937.3	0.43567314	2.48E-23	postive
NLRC4	AC008937.3	0.411917064	8.49E-21	postive
PJVK	AC008937.3	0.455129572	1.46E-25	postive
SCAF11	AC008937.3	0.729734409	8.55E-80	postive
GSDME	AL645568.1	0.627529883	3.74E-53	postive
IL1A	AL645568.1	0.487850352	1.19E-29	postive
IL1B	AL645568.1	0.563965053	4.57E-41	postive
NLRC4	AL645568.1	0.562332806	8.64E-41	postive
NLRP3	AL645568.1	0.522875523	1.51E-34	postive
SCAF11	AL645568.1	0.595910929	8.36E-47	postive
CHMP2A	LINC00641	-0.460510691	3.32E-26	negative
CHMP6	LINC00641	-0.434877765	3.04E-23	negative
GSDME	LINC00641	0.548402826	1.72E-38	postive
IL1A	LINC00641	0.401622945	9.25E-20	postive
IL1B	LINC00641	0.44487673	2.28E-24	postive
NLRC4	LINC00641	0.495302665	1.20E-30	postive
NLRP3	LINC00641	0.407639075	2.31E-20	postive
SCAF11	LINC00641	0.702478037	1.42E-71	postive
SCAF11	PRKAR1B-AS2	0.406127981	3.28E-20	postive
GSDME	AC112503.2	0.543727638	9.62E-38	postive
NLRC4	AC112503.2	0.437549391	1.53E-23	postive
NOD1	AC112503.2	0.416397904	2.92E-21	postive
PJVK	AC112503.2	0.448321418	9.14E-25	postive
SCAF11	AC112503.2	0.622991763	3.38E-52	postive

Pyroptosis-related Genes	lncRNA	cor	pvalue	Regulation
GSDME	AC018926.2	0.561617476	1.14E-40	postive
IL1A	AC018926.2	0.488779295	8.95E-30	postive
IL1B	AC018926.2	0.584557241	1.08E-44	postive
NLRC4	AC018926.2	0.50434183	6.92E-32	postive
NLRP3	AC018926.2	0.505204673	5.25E-32	postive
SCAF11	AC018926.2	0.607587006	4.58E-49	postive
GSDME	AC068189.1	0.412348239	7.67E-21	postive
SCAF11	AC068189.1	0.573784807	9.18E-43	postive
GSDME	AC122129.1	0.52931009	1.65E-35	postive
NLRC4	AC122129.1	0.436924367	1.80E-23	postive
NLRP3	AC122129.1	0.416455036	2.88E-21	postive
NOD1	AC122129.1	0.491420777	3.99E-30	postive
SCAF11	AC122129.1	0.567674105	1.06E-41	postive
NOD1	AC005674.2	0.432827718	5.11E-23	postive
PJVK	AC005674.2	0.587543012	3.07E-45	postive
SCAF11	AC005674.2	0.557400552	5.79E-40	postive
CHMP2A	AC066613.1	-0.455842589	1.20E-25	negative
GSDME	AC066613.1	0.570447906	3.51E-42	postive
IL1A	AC066613.1	0.441598112	5.37E-24	postive
IL1B	AC066613.1	0.492045416	3.29E-30	postive
NLRC4	AC066613.1	0.524517136	8.63E-35	postive
NLRP3	AC066613.1	0.48754573	1.30E-29	postive
SCAF11	AC066613.1	0.697395872	3.81E-70	postive
PJVK	LINC00662	0.482639688	5.70E-29	postive
SCAF11	LINC00662	0.475348108	4.89E-28	postive
IL1B	LINC00923	0.456769802	9.32E-26	postive
SCAF11	LINC00923	0.415616263	3.53E-21	postive
NOD1	AC138028.4	0.424928434	3.68E-22	postive
PLCG1	AC138028.4	0.417154819	2.44E-21	postive
SCAF11	AC007342.5	0.546213129	3.87E-38	postive
CHMP2A	ITGA6-AS1	-0.432037152	6.24E-23	negative
CHMP6	ITGA6-AS1	-0.414613532	4.48E-21	negative
GSDME	ITGA6-AS1	0.460719433	3.14E-26	postive
SCAF11	ITGA6-AS1	0.674809074	3.87E-64	postive
GSDME	CSNK1G2-AS1	0.494185228	1.70E-30	postive
NLRP3	CSNK1G2-AS1	0.416251003	3.03E-21	postive
NOD1	CSNK1G2-AS1	0.461793233	2.33E-26	postive
SCAF11	CSNK1G2-AS1	0.605654317	1.10E-48	postive

Pyroptosis-related Genes	lncRNA	cor	pvalue	Regulation
PJVK	AC100791.3	0.596209997	7.34E-47	postive
SCAF11	AC100791.3	0.475043957	5.34E-28	postive
CHMP4B	LINC01315	0.45166938	3.73E-25	postive
GSDME	AC018755.4	0.639577617	9.05E-56	postive
IL1A	AC018755.4	0.56534984	2.65E-41	postive
IL1B	AC018755.4	0.734459129	2.54E-81	postive
GSDMC	AC018755.4	0.417999004	1.99E-21	postive
NLRC4	AC018755.4	0.652670268	9.48E-59	postive
NLRP3	AC018755.4	0.667555335	2.54E-62	postive
SCAF11	AC018755.4	0.432578475	5.44E-23	postive
TNF	AC018755.4	0.404942375	4.32E-20	postive
PJVK	LINC01160	0.440103395	7.93E-24	postive
PJVK	AL121895.1	0.521334108	2.55E-34	postive
SCAF11	AL121895.1	0.598358543	2.86E-47	postive
CHMP2A	SCAT2	-0.441383456	5.68E-24	negative
CHMP6	SCAT2	-0.433935901	3.86E-23	negative
GSDME	SCAT2	0.465537646	8.14E-27	postive
IL1A	SCAT2	0.402343118	7.84E-20	postive
IL1B	SCAT2	0.416175185	3.08E-21	postive
NLRC4	SCAT2	0.42897923	1.35E-22	postive
PJVK	SCAT2	0.456970989	8.82E-26	postive
SCAF11	SCAT2	0.730214473	6.00E-80	postive
NOD1	AC120053.1	0.471327846	1.57E-27	postive
PJVK	AC120053.1	0.548362101	1.75E-38	postive
SCAF11	AC120053.1	0.493506299	2.10E-30	postive
GSDME	ZMIZ1-AS1	0.538074513	7.45E-37	postive
IL1A	ZMIZ1-AS1	0.416107666	3.14E-21	postive
IL1B	ZMIZ1-AS1	0.536342519	1.38E-36	postive
NLRC4	ZMIZ1-AS1	0.443587487	3.19E-24	postive
NLRP3	ZMIZ1-AS1	0.517256992	1.01E-33	postive
SCAF11	ZMIZ1-AS1	0.412752563	6.97E-21	postive
CHMP2A	PPP3CB-AS1	-0.440858628	6.51E-24	negative
GSDME	PPP3CB-AS1	0.502406062	1.29E-31	postive
NLRC4	PPP3CB-AS1	0.432918588	5.00E-23	postive
PJVK	PPP3CB-AS1	0.471920615	1.32E-27	postive
SCAF11	PPP3CB-AS1	0.660589731	1.26E-60	postive
GSDME	UBOX5-AS1	0.426050821	2.79E-22	postive
NOD1	UBOX5-AS1	0.427516653	1.94E-22	postive

Pyroptosis-related Genes	lncRNA	cor	pvalue	Regulation
PJVK	UBOX5-AS1	0.481755812	7.42E-29	postive
SCAF11	UBOX5-AS1	0.576092999	3.59E-43	postive
GSDME	AC084824.6	0.415148376	3.94E-21	postive
PJVK	AC084824.6	0.485708702	2.27E-29	postive
SCAF11	AC084824.6	0.672388093	1.58E-63	postive
SCAF11	AC020658.4	0.420662785	1.04E-21	postive
PJVK	AC012676.4	0.442586356	4.15E-24	postive
SCAF11	AC012676.4	0.544249794	7.95E-38	postive
GSDME	LINC02035	0.489590153	6.99E-30	postive
NLRC4	LINC02035	0.404681645	4.59E-20	postive
PJVK	LINC02035	0.42798548	1.72E-22	postive
SCAF11	LINC02035	0.563863306	4.75E-41	postive
PJVK	AC009107.2	0.452851803	2.71E-25	postive
GSDME	AC007038.1	0.455861405	1.19E-25	postive
NLRC4	AC007038.1	0.411141252	1.02E-20	postive
NOD1	AC007038.1	0.420502743	1.09E-21	postive
PJVK	AC007038.1	0.575729999	4.17E-43	postive
SCAF11	AC007038.1	0.648979403	6.79E-58	postive
GSDME	AC108463.2	0.651532727	1.74E-58	postive
IL1A	AC108463.2	0.434193208	3.61E-23	postive
IL1B	AC108463.2	0.518096853	7.61E-34	postive
NLRC4	AC108463.2	0.568406658	7.93E-42	postive
NLRP3	AC108463.2	0.546862548	3.04E-38	postive
SCAF11	AC108463.2	0.621513261	6.88E-52	postive
SCAF11	AL031716.1	0.489445966	7.31E-30	postive
CHMP2A	AC078778.1	-0.420859133	9.96E-22	negative
GSDME	AC078778.1	0.454607198	1.68E-25	postive
NLRC4	AC078778.1	0.40688586	2.76E-20	postive
PJVK	AC078778.1	0.404181305	5.15E-20	postive
SCAF11	AC078778.1	0.71530852	2.53E-75	postive
GSDME	AC092171.1	0.547905273	2.07E-38	postive
NLRC4	AC092171.1	0.437523611	1.54E-23	postive
NOD1	AC092171.1	0.458187132	6.31E-26	postive
PJVK	AC092171.1	0.424126984	4.48E-22	postive
SCAF11	AC092171.1	0.640642448	5.24E-56	postive
SCAF11	AF131215.5	0.45002056	5.80E-25	postive
GSDME	AC068790.6	0.515169775	2.02E-33	postive
NLRC4	AC068790.6	0.439185791	1.01E-23	postive

Pyroptosis-related Genes	lncRNA	cor	pvalue	Regulation
PJVK	AC068790.6	0.470037568	2.27E-27	postive
SCAF11	AC068790.6	0.668464613	1.51E-62	postive
CHMP4B	LINC02487	0.409047402	1.67E-20	postive
PLCG1	LINC02487	0.422877523	6.09E-22	postive
PJVK	AC012313.2	0.462675378	1.82E-26	postive
PJVK	AL035416.1	0.515438806	1.85E-33	postive
SCAF11	AL035416.1	0.460886212	2.99E-26	postive
CHMP2A	LINC01572	-0.417492544	2.25E-21	negative
GSDME	LINC01572	0.402602303	7.39E-20	postive
CASP8	LINC01572	0.414158086	4.99E-21	postive
NLRC4	LINC01572	0.409066266	1.66E-20	postive
NOD1	LINC01572	0.447613948	1.10E-24	postive
PJVK	LINC01572	0.50429595	7.03E-32	postive
SCAF11	LINC01572	0.679738173	2.10E-65	postive
PLCG1	OGFR-AS1	0.530262103	1.18E-35	postive
CHMP2A	AC068025.2	-0.433145059	4.72E-23	negative
GSDME	AC068025.2	0.513770147	3.22E-33	postive
NLRC4	AC068025.2	0.440169851	7.79E-24	postive
SCAF11	AC068025.2	0.710258638	7.99E-74	postive
CHMP2A	AC025569.1	-0.403931444	5.45E-20	negative
GSDME	AC025569.1	0.565027924	3.01E-41	postive
IL1A	AC025569.1	0.513183982	3.90E-33	postive
IL1B	AC025569.1	0.529723341	1.43E-35	postive
NLRC4	AC025569.1	0.546329598	3.70E-38	postive
NLRP3	AC025569.1	0.415875862	3.31E-21	postive
SCAF11	AC025569.1	0.668404683	1.56E-62	postive
CHMP2A	DHDDS-AS1	-0.420793826	1.01E-21	negative
GSDME	DHDDS-AS1	0.548664338	1.56E-38	postive
IL1A	DHDDS-AS1	0.428063612	1.69E-22	postive
IL1B	DHDDS-AS1	0.434906291	3.01E-23	postive
NLRC4	DHDDS-AS1	0.454306676	1.83E-25	postive
NLRP3	DHDDS-AS1	0.445435628	1.96E-24	postive
SCAF11	DHDDS-AS1	0.683992935	1.62E-66	postive
GSDME	AF230666.2	0.600082239	1.33E-47	postive
IL1A	AF230666.2	0.494615384	1.49E-30	postive
IL1B	AF230666.2	0.545712429	4.65E-38	postive
NLRC4	AF230666.2	0.531901209	6.66E-36	postive
NLRP3	AF230666.2	0.455396029	1.36E-25	postive

Pyroptosis-related Genes	lncRNA	cor	pvalue	Regulation
PJVK	AF230666.2	0.4304657	9.27E-23	postive
SCAF11	AF230666.2	0.693973951	3.36E-69	postive
PJVK	LINC02532	0.411543887	9.27E-21	postive
SCAF11	GPRC5D-AS1	0.433076436	4.80E-23	postive
GSDME	AL512328.1	0.412700851	7.05E-21	postive
IL1B	AL512328.1	0.52651421	4.34E-35	postive
NLRC4	AL512328.1	0.402270832	7.98E-20	postive
NLRP3	AL512328.1	0.408422158	1.93E-20	postive
CHMP2A	GABPB1-AS1	-0.409642312	1.45E-20	negative
GSDME	GABPB1-AS1	0.441038414	6.22E-24	postive
NLRC4	GABPB1-AS1	0.403278875	6.33E-20	postive
PJVK	GABPB1-AS1	0.420660497	1.05E-21	postive
SCAF11	GABPB1-AS1	0.56450078	3.70E-41	postive
GSDME	DLEU2	0.479935869	1.27E-28	postive
NLRC4	DLEU2	0.428271365	1.61E-22	postive
NOD1	DLEU2	0.482231807	6.44E-29	postive
SCAF11	DLEU2	0.661639049	7.06E-61	postive
PLCG1	CCDC183-AS1	0.411662403	9.01E-21	postive
CHMP2A	AC010976.1	-0.428810143	1.40E-22	negative
CHMP6	AC010976.1	-0.410999307	1.05E-20	negative
GSDME	AC010976.1	0.563320474	5.88E-41	postive
IL1A	AC010976.1	0.408081483	2.09E-20	postive
IL1B	AC010976.1	0.421767805	7.98E-22	postive
NLRC4	AC010976.1	0.486487919	1.79E-29	postive
NOD1	AC010976.1	0.402060738	8.37E-20	postive
PJVK	AC010976.1	0.423175423	5.66E-22	postive
SCAF11	AC010976.1	0.716963923	8.01E-76	postive
PJVK	AC037487.1	0.440025599	8.09E-24	postive
SCAF11	AC037487.1	0.462130284	2.12E-26	postive
SCAF11	SKAP1-AS1	0.466555265	6.11E-27	postive
CHMP2A	AL022097.1	-0.408007218	2.12E-20	negative
GSDME	AL022097.1	0.596972861	5.25E-47	postive
IL1A	AL022097.1	0.440766653	6.67E-24	postive
IL1B	AL022097.1	0.455915977	1.18E-25	postive
NLRC4	AL022097.1	0.508382474	1.88E-32	postive
NOD1	AL022097.1	0.405717055	3.61E-20	postive
PJVK	AL022097.1	0.416563216	2.81E-21	postive
SCAF11	AL022097.1	0.687300914	2.15E-67	postive

Pyroptosis-related Genes	lncRNA	cor	pvalue	Regulation
NOD1	AC233280.1	0.44169226	5.24E-24	postive
GSDME	AC010327.5	0.426741178	2.35E-22	postive
SCAF11	AC010327.5	0.408461142	1.91E-20	postive
PJVK	AC012360.1	0.412683306	7.08E-21	postive
SCAF11	AC012360.1	0.631755538	4.66E-54	postive
GSDME	AC025580.2	0.51749322	9.32E-34	postive
IL1A	AC025580.2	0.478470617	1.96E-28	postive
IL1B	AC025580.2	0.567680992	1.06E-41	postive
NLRC4	AC025580.2	0.482797223	5.44E-29	postive
NLRP3	AC025580.2	0.421474556	8.58E-22	postive
SCAF11	AC025580.2	0.54739875	2.50E-38	postive
GSDME	AC083862.1	0.546613715	3.34E-38	postive
NLRC4	AC083862.1	0.534452181	2.71E-36	postive
NLRP3	AC083862.1	0.475299518	4.96E-28	postive
NOD1	AC083862.1	0.432979368	4.92E-23	postive
SCAF11	AC083862.1	0.488916934	8.59E-30	postive
GSDME	ADNP-AS1	0.428108348	1.67E-22	postive
PJVK	ADNP-AS1	0.491045216	4.48E-30	postive
SCAF11	ADNP-AS1	0.661543433	7.45E-61	postive
GSDME	AC087392.4	0.45719315	8.30E-26	postive
IL1B	AC087392.4	0.409214462	1.60E-20	postive
NLRC4	AC087392.4	0.40210455	8.28E-20	postive
SCAF11	AC087392.4	0.468827425	3.20E-27	postive
PJVK	PVT1	0.442066467	4.76E-24	postive
CHMP2A	AP4B1-AS1	-0.425208607	3.43E-22	negative
SCAF11	AP4B1-AS1	0.658105841	4.96E-60	postive
CASP8	SNHG4	0.429212836	1.27E-22	postive
PJVK	SNHG4	0.448797873	8.05E-25	postive
SCAF11	SNHG4	0.509716744	1.22E-32	postive
CHMP2A	PABPC4-AS1	-0.411605784	9.14E-21	negative
GSDME	PABPC4-AS1	0.409123266	1.64E-20	postive
NLRC4	PABPC4-AS1	0.403792912	5.63E-20	postive
PJVK	PABPC4-AS1	0.447515948	1.13E-24	postive
SCAF11	PABPC4-AS1	0.718197261	3.39E-76	postive
NOD1	AC005253.1	0.462465802	1.93E-26	postive
SCAF11	AC005253.1	0.431748876	6.71E-23	postive
GSDME	EDIL3-DT	0.634614482	1.12E-54	postive
IL1A	EDIL3-DT	0.419794722	1.29E-21	postive

Pyroptosis-related Genes	lncRNA	cor	pvalue	Regulation
IL1B	EDIL3-DT	0.468349775	3.67E-27	postive
NLRC4	EDIL3-DT	0.495304279	1.20E-30	postive
NLRP3	EDIL3-DT	0.511777398	6.20E-33	postive
SCAF11	EDIL3-DT	0.457588647	7.44E-26	postive
CHMP2A	NARF-IT1	-0.44086205	6.51E-24	negative
GSDME	NARF-IT1	0.539887245	3.88E-37	postive
IL1A	NARF-IT1	0.434542103	3.31E-23	postive
IL1B	NARF-IT1	0.463852175	1.31E-26	postive
NLRC4	NARF-IT1	0.467478654	4.70E-27	postive
NLRP3	NARF-IT1	0.462202062	2.08E-26	postive
SCAF11	NARF-IT1	0.640769047	4.91E-56	postive
ELANE	AC093278.2	0.403088394	6.61E-20	postive
GSDME	AC093278.2	0.46979251	2.43E-27	postive
NLRC4	AC093278.2	0.577191974	2.29E-43	postive
NLRP1	AC093278.2	0.402874211	6.95E-20	postive
CHMP2A	AC016831.1	-0.402060108	8.37E-20	negative
GSDME	AC016831.1	0.402755246	7.14E-20	postive
SCAF11	AC016831.1	0.697471218	3.63E-70	postive
GSDME	AL513534.3	0.512922719	4.25E-33	postive
PJVK	AL513534.3	0.413257683	6.18E-21	postive
SCAF11	AL513534.3	0.530413543	1.12E-35	postive
NLRC4	AC008972.2	0.455900898	1.18E-25	postive
GSDME	AL442067.2	0.55161435	5.19E-39	postive
IL1B	AL442067.2	0.448547263	8.60E-25	postive
NLRC4	AL442067.2	0.491823616	3.53E-30	postive
SCAF11	AL442067.2	0.571551921	2.26E-42	postive
CHMP2A	ST7-AS2	-0.420636458	1.05E-21	negative
GSDME	ST7-AS2	0.565603223	2.40E-41	postive
IL1A	ST7-AS2	0.458381562	5.98E-26	postive
IL1B	ST7-AS2	0.468760533	3.26E-27	postive
NLRC4	ST7-AS2	0.479147755	1.61E-28	postive
SCAF11	ST7-AS2	0.713913613	6.61E-75	postive
PLCG1	PTOV1-AS2	0.450746112	4.78E-25	postive
CHMP4A	AL139423.1	0.40540223	3.89E-20	postive
PJVK	AP003352.1	0.513262241	3.80E-33	postive
CHMP6	AC026368.1	-0.401940047	8.60E-20	negative
GSDME	AC026368.1	0.495610411	1.09E-30	postive
IL1B	AC026368.1	0.420725022	1.03E-21	postive

Pyroptosis-related Genes	lncRNA	cor	pvalue	Regulation
NLRC4	AC026368.1	0.410776645	1.11E-20	postive
PJVK	AC026368.1	0.46391704	1.28E-26	postive
SCAF11	AC026368.1	0.688848209	8.29E-68	postive
GSDME	MACORIS	0.464075204	1.23E-26	postive
NLRC4	MACORIS	0.521019284	2.84E-34	postive
GSDME	LINC00562	0.407628669	2.32E-20	postive
NOD1	LINC00562	0.490483423	5.32E-30	postive
SCAF11	LINC00562	0.42003774	1.22E-21	postive
GSDME	SNHG14	0.561209461	1.34E-40	postive
NLRC4	SNHG14	0.43893504	1.07E-23	postive
NOD1	SNHG14	0.463544299	1.43E-26	postive
PJVK	SNHG14	0.460047558	3.78E-26	postive
SCAF11	SNHG14	0.57321514	1.16E-42	postive
GSDME	MPRIP-AS1	0.539748375	4.08E-37	postive
IL1A	MPRIP-AS1	0.432385462	5.72E-23	postive
IL1B	MPRIP-AS1	0.478750481	1.81E-28	postive
NLRC4	MPRIP-AS1	0.460678149	3.17E-26	postive
NLRP3	MPRIP-AS1	0.436687006	1.91E-23	postive
SCAF11	MPRIP-AS1	0.59249753	3.68E-46	postive
GSDME	SOCAR	0.595411711	1.04E-46	postive
IL1A	SOCAR	0.445975244	1.70E-24	postive
IL1B	SOCAR	0.520625398	3.25E-34	postive
NLRC4	SOCAR	0.506141582	3.88E-32	postive
NLRP3	SOCAR	0.556949513	6.88E-40	postive
SCAF11	SOCAR	0.47908262	1.64E-28	postive
GSDME	SLC7A11-AS1	0.454481928	1.74E-25	postive
IL1A	SLC7A11-AS1	0.437457232	1.57E-23	postive
IL1B	SLC7A11-AS1	0.510529651	9.34E-33	postive
NLRP3	SLC7A11-AS1	0.473609158	8.11E-28	postive
SCAF11	SLC7A11-AS1	0.47603755	4.00E-28	postive
BAX	PCBP1-AS1	-0.402905033	6.90E-20	negative
CHMP2A	PCBP1-AS1	-0.433575749	4.23E-23	negative
CHMP6	PCBP1-AS1	-0.410227394	1.26E-20	negative
GSDME	PCBP1-AS1	0.518133603	7.52E-34	postive
NLRC4	PCBP1-AS1	0.478251731	2.09E-28	postive
PJVK	PCBP1-AS1	0.459554412	4.33E-26	postive
SCAF11	PCBP1-AS1	0.598682373	2.48E-47	postive
PJVK	LINC02416	0.561096773	1.40E-40	postive

Pyroptosis-related Genes	lncRNA	cor	pvalue	Regulation
GSDME	PLAC4	0.402414144	7.72E-20	postive
SCAF11	PLAC4	0.419826987	1.28E-21	postive
CHMP2A	AC012447.1	-0.405903905	3.46E-20	negative
GSDME	AC012447.1	0.586577352	4.62E-45	postive
IL1A	AC012447.1	0.493786038	1.93E-30	postive
IL1B	AC012447.1	0.514372838	2.63E-33	postive
NLRC4	AC012447.1	0.493485193	2.11E-30	postive
NLRP3	AC012447.1	0.44204959	4.78E-24	postive
SCAF11	AC012447.1	0.680968511	1.01E-65	postive
CASP8	AC012467.2	0.417254519	2.38E-21	postive
PJVK	AC012467.2	0.573002582	1.26E-42	postive
SCAF11	AC012467.2	0.53199082	6.45E-36	postive
CHMP2A	AL022311.1	-0.429958628	1.05E-22	negative
CHMP6	AL022311.1	-0.420294743	1.14E-21	negative
GSDME	AL022311.1	0.446943614	1.32E-24	postive
NLRC4	AL022311.1	0.421013587	9.59E-22	postive
PJVK	AL022311.1	0.501065266	1.97E-31	postive
SCAF11	AL022311.1	0.655307941	2.28E-59	postive
NOD1	ZNF252P-AS1	0.448314159	9.15E-25	postive
PJVK	ZNF252P-AS1	0.51656781	1.27E-33	postive
SCAF11	ZNF252P-AS1	0.407621781	2.32E-20	postive
CHMP4A	AC095057.3	0.415478072	3.64E-21	postive
PJVK	AC095057.3	0.47582507	4.26E-28	postive
SCAF11	AC095057.3	0.467052016	5.31E-27	postive
CHMP2A	AC020915.1	-0.423346043	5.43E-22	negative
GSDME	AC020915.1	0.508733131	1.68E-32	postive
NLRC4	AC020915.1	0.47345202	8.48E-28	postive
NOD1	AC020915.1	0.442706197	4.02E-24	postive
SCAF11	AC020915.1	0.675951913	1.98E-64	postive
GSDME	AC010768.2	0.42927217	1.25E-22	postive
SCAF11	AC010768.2	0.415717224	3.44E-21	postive
GSDME	AC129507.1	0.406652988	2.91E-20	postive
NOD1	AL031846.2	0.456335609	1.05E-25	postive
PJVK	AL031846.2	0.478450549	1.97E-28	postive
TIRAP	AL031846.2	0.438362715	1.24E-23	postive
GSDME	LINC02656	0.57985449	7.67E-44	postive
IL1A	LINC02656	0.61210625	5.75E-50	postive
IL1B	LINC02656	0.776596452	1.46E-96	postive

Pyroptosis-related Genes	lncRNA	cor	pvalue	Regulation
NLRC4	LINC02656	0.497266375	6.51E-31	postive
NLRP3	LINC02656	0.595109491	1.19E-46	postive
SCAF11	LINC02656	0.506607101	3.34E-32	postive
SCAF11	SREBF2-AS1	0.428855766	1.39E-22	postive
CHMP2A	PSMD6-AS2	-0.445979429	1.70E-24	negative
CHMP6	PSMD6-AS2	-0.401253049	1.01E-19	negative
GSDME	PSMD6-AS2	0.526772216	3.97E-35	postive
NLRC4	PSMD6-AS2	0.462733187	1.79E-26	postive
NOD1	PSMD6-AS2	0.414068333	5.10E-21	postive
SCAF11	PSMD6-AS2	0.706760195	8.37E-73	postive
CHMP4B	LINC02441	0.459967366	3.86E-26	postive
NOD1	AL355472.3	0.402521537	7.53E-20	postive
PJVK	AL355472.3	0.459034813	5.00E-26	postive
SCAF11	AL355472.3	0.528165657	2.45E-35	postive
CHMP2A	AC138207.4	-0.413227087	6.23E-21	negative
GSDME	AC138207.4	0.519746767	4.37E-34	postive
NLRC4	AC138207.4	0.486251925	1.93E-29	postive
PJVK	AC138207.4	0.565790066	2.23E-41	postive
SCAF11	AC138207.4	0.673522314	8.19E-64	postive
CHMP2A	AC008115.3	-0.429114434	1.30E-22	negative
CHMP6	AC008115.3	-0.420459372	1.10E-21	negative
GSDME	AC008115.3	0.424111609	4.50E-22	postive
SCAF11	AC008115.3	0.66575599	7.03E-62	postive
GSDME	AC019254.1	0.568774614	6.85E-42	postive
IL1A	AC019254.1	0.517966783	7.95E-34	postive
IL1B	AC019254.1	0.615779732	1.04E-50	postive
NLRC4	AC019254.1	0.475792898	4.30E-28	postive
NLRP3	AC019254.1	0.553492735	2.56E-39	postive
SCAF11	AC019254.1	0.411891652	8.54E-21	postive
GSDME	AL157938.3	0.517074697	1.07E-33	postive
NLRC4	AL157938.3	0.468972797	3.07E-27	postive
PJVK	AL157938.3	0.473726567	7.83E-28	postive
SCAF11	AL157938.3	0.711230481	4.13E-74	postive
NOD1	AL391095.4	0.416751005	2.69E-21	postive
SCAF11	AL391095.4	0.411312991	9.79E-21	postive
GSDME	AC112722.1	0.417385543	2.31E-21	postive
SCAF11	AC112722.1	0.556739096	7.46E-40	postive
IL1B	LINC00528	0.40940398	1.53E-20	postive

Pyroptosis-related Genes	lncRNA	cor	pvalue	Regulation
NLRC4	LINC00528	0.564157954	4.24E-41	postive
NLRP1	LINC00528	0.47169647	1.41E-27	postive
NLRP3	LINC00528	0.441624578	5.34E-24	postive
TNF	LINC00528	0.4916331	3.74E-30	postive
CHMP2A	PRC1-AS1	-0.43486834	3.04E-23	negative
CHMP6	PRC1-AS1	-0.407477961	2.40E-20	negative
GSDME	PRC1-AS1	0.472524666	1.11E-27	postive
IL1B	PRC1-AS1	0.402083259	8.32E-20	postive
NLRC4	PRC1-AS1	0.425075577	3.55E-22	postive
SCAF11	PRC1-AS1	0.661775057	6.55E-61	postive
CHMP2A	LINC01355	-0.420057301	1.21E-21	negative
CHMP6	LINC01355	-0.406648134	2.91E-20	negative
GSDME	LINC01355	0.491551384	3.84E-30	postive
NLRC4	LINC01355	0.456217294	1.08E-25	postive
NOD1	LINC01355	0.419195975	1.49E-21	postive
PJVK	LINC01355	0.521966326	2.06E-34	postive
SCAF11	LINC01355	0.6891353	6.94E-68	postive
PJVK	TMCC1-AS1	0.488537195	9.64E-30	postive
GSDME	AC007285.2	0.529376787	1.61E-35	postive
NLRC4	AC007285.2	0.442545174	4.20E-24	postive
NOD1	AC007285.2	0.41715681	2.44E-21	postive
SCAF11	AC007285.2	0.699714141	8.56E-71	postive
PJVK	AL133551.1	0.55692545	6.95E-40	postive
PJVK	PRANCR	0.414637874	4.45E-21	postive
SCAF11	PRANCR	0.412659813	7.12E-21	postive
GSDME	AC040169.3	0.471352623	1.55E-27	postive
SCAF11	AC040169.3	0.575384097	4.80E-43	postive
GSDME	AC007728.3	0.529195463	1.71E-35	postive
IL1A	AC007728.3	0.49349303	2.11E-30	postive
IL1B	AC007728.3	0.561328002	1.28E-40	postive
NLRC4	AC007728.3	0.481287939	8.52E-29	postive
NLRP3	AC007728.3	0.501087184	1.95E-31	postive
SCAF11	AC007728.3	0.634839176	9.99E-55	postive
SCAF11	AC005277.2	0.541026494	2.57E-37	postive
CHMP2A	SAP30L-AS1	-0.430497937	9.20E-23	negative
CHMP6	SAP30L-AS1	-0.404939208	4.32E-20	negative
GSDME	SAP30L-AS1	0.564544562	3.64E-41	postive
NLRC4	SAP30L-AS1	0.463584209	1.41E-26	postive

Pyroptosis-related Genes	lncRNA	cor	pvalue	Regulation
SCAF11	SAP30L-AS1	0.688467798	1.05E-67	postive
PJVK	AL110115.2	0.476292644	3.71E-28	postive
NOD1	AC046134.2	0.482835247	5.38E-29	postive
SCAF11	AC046134.2	0.435541714	2.56E-23	postive
NOD1	LINC00265	0.494501484	1.54E-30	postive
PLCG1	LINC00265	0.566275239	1.84E-41	postive
GSDME	AC113361.1	0.493574732	2.06E-30	postive
NOD1	AC113361.1	0.456353917	1.04E-25	postive
SCAF11	AC113361.1	0.481913519	7.08E-29	postive
CHMP2A	AL592435.1	-0.419163949	1.50E-21	negative
GSDME	AL592435.1	0.463734374	1.35E-26	postive
NOD1	AL592435.1	0.432908343	5.01E-23	postive
PJVK	AL592435.1	0.514792061	2.29E-33	postive
SCAF11	AL592435.1	0.669175861	1.01E-62	postive
GSDME	AC022960.1	0.43883377	1.10E-23	postive
SCAF11	AC022960.1	0.558328457	4.06E-40	postive
SCAF11	AC092301.1	0.520139894	3.83E-34	postive
CHMP2A	THOC7-AS1	-0.438507416	1.20E-23	negative
CHMP6	THOC7-AS1	-0.414052258	5.12E-21	negative
GSDME	THOC7-AS1	0.493603856	2.04E-30	postive
NLRC4	THOC7-AS1	0.41754445	2.22E-21	postive
SCAF11	THOC7-AS1	0.700473816	5.23E-71	postive
CHMP6	AC073333.1	-0.404839236	4.42E-20	negative
GSDME	AC073333.1	0.437411262	1.59E-23	postive
NLRC4	AC073333.1	0.401325619	9.89E-20	postive
NOD1	AC073333.1	0.420917643	9.82E-22	postive
SCAF11	AC073333.1	0.652743708	9.11E-59	postive
CHMP2A	LINC00630	-0.451541261	3.86E-25	negative
GSDME	LINC00630	0.454608591	1.68E-25	postive
NLRC4	LINC00630	0.413069853	6.46E-21	postive
NOD1	LINC00630	0.424159643	4.44E-22	postive
PJVK	LINC00630	0.458443766	5.88E-26	postive
SCAF11	LINC00630	0.708556874	2.52E-73	postive
GSDME	ATP11A-AS1	0.555329781	1.28E-39	postive
IL1A	ATP11A-AS1	0.404322105	4.98E-20	postive
IL1B	ATP11A-AS1	0.417638878	2.17E-21	postive
NLRC4	ATP11A-AS1	0.469091242	2.97E-27	postive
NOD1	ATP11A-AS1	0.427563725	1.92E-22	postive

Pyroptosis-related Genes	lncRNA	cor	pvalue	Regulation
SCAF11	ATP11A-AS1	0.670999169	3.53E-63	postive
SCAF11	AP001469.2	0.499048498	3.72E-31	postive
CHMP2A	AC019080.1	-0.472576493	1.09E-27	negative
CHMP6	AC019080.1	-0.423908713	4.73E-22	negative
GSDME	AC019080.1	0.564240671	4.10E-41	postive
NLRC4	AC019080.1	0.485762609	2.23E-29	postive
NOD1	AC019080.1	0.43740655	1.59E-23	postive
PJVK	AC019080.1	0.51495266	2.17E-33	postive
SCAF11	AC019080.1	0.70776173	4.29E-73	postive
PJVK	AC116025.1	0.481183988	8.79E-29	postive
GSDME	AC027097.2	0.623218071	3.03E-52	postive
IL1A	AC027097.2	0.423920647	4.71E-22	postive
IL1B	AC027097.2	0.473276725	8.92E-28	postive
NLRC4	AC027097.2	0.552059212	4.39E-39	postive
NLRP3	AC027097.2	0.5321029	6.20E-36	postive
SCAF11	AC027097.2	0.646959321	1.97E-57	postive
CHMP6	MAFG-DT	0.424354404	4.24E-22	postive
PJVK	AC022306.2	0.444511785	2.51E-24	postive
SCAF11	AC022306.2	0.571262946	2.54E-42	postive
CHMP2A	MIR155HG	-0.432805529	5.14E-23	negative
GSDME	MIR155HG	0.432189087	6.01E-23	postive
CASP8	MIR155HG	0.486295226	1.90E-29	postive
NLRC4	MIR155HG	0.536768583	1.19E-36	postive
SCAF11	MIR155HG	0.625139722	1.20E-52	postive
CHMP2A	AC053513.2	-0.438436792	1.22E-23	negative
GSDME	AC053513.2	0.507261697	2.70E-32	postive
NLRC4	AC053513.2	0.438464494	1.21E-23	postive
PJVK	AC053513.2	0.450333796	5.34E-25	postive
SCAF11	AC053513.2	0.726628742	8.29E-79	postive
CHMP2A	AP001107.4	-0.410458265	1.20E-20	negative
NLRP3	AP001107.4	0.415943121	3.26E-21	postive
SCAF11	AP001107.4	0.486409911	1.84E-29	postive
CHMP6	AP000254.2	-0.40714014	2.60E-20	negative
PJVK	AP000254.2	0.467973486	4.08E-27	postive
SCAF11	AP000254.2	0.531746499	7.03E-36	postive
GSDME	AL929236.1	0.530563484	1.06E-35	postive
NLRC4	AL929236.1	0.425466246	3.22E-22	postive
NOD1	AL929236.1	0.401107892	1.04E-19	postive

Pyroptosis-related Genes	lncRNA	cor	pvalue	Regulation
PJVK	AL929236.1	0.442407219	4.35E-24	postive
SCAF11	AL929236.1	0.558452837	3.87E-40	postive
PJVK	AC137932.3	0.468087082	3.96E-27	postive
SCAF11	AC137932.3	0.471904611	1.33E-27	postive
GSDME	LINC02345	0.576063957	3.64E-43	postive
NLRC4	LINC02345	0.509473433	1.32E-32	postive
NLRP3	LINC02345	0.465819309	7.52E-27	postive
CHMP2A	AC105429.1	-0.411365232	9.67E-21	negative
GSDME	AC105429.1	0.536776082	1.19E-36	postive
IL1A	AC105429.1	0.439833429	8.50E-24	postive
IL1B	AC105429.1	0.447471468	1.15E-24	postive
NLRC4	AC105429.1	0.433359837	4.47E-23	postive
NLRP3	AC105429.1	0.417529569	2.23E-21	postive
PJVK	AC105429.1	0.48026417	1.15E-28	postive
SCAF11	AC105429.1	0.652788912	8.90E-59	postive
PYCARD	AL023803.1	0.410216523	1.27E-20	postive
GSDME	AC020915.2	0.473707135	7.88E-28	postive
SCAF11	AC020915.2	0.539220082	4.93E-37	postive
PJVK	AC104785.1	0.481790076	7.34E-29	postive
SCAF11	AC104785.1	0.505718947	4.45E-32	postive
GSDME	MIR17HG	0.408531417	1.88E-20	postive
NOD1	MIR17HG	0.462030805	2.18E-26	postive
PJVK	MIR17HG	0.433825224	3.97E-23	postive
SCAF11	MIR17HG	0.679564793	2.33E-65	postive
SCAF11	AC092718.6	0.406173722	3.25E-20	postive
GSDME	AC113143.1	0.491331572	4.10E-30	postive
NLRC4	AC113143.1	0.451270024	4.15E-25	postive
PJVK	AC113143.1	0.416364552	2.95E-21	postive
SCAF11	AC113143.1	0.667135916	3.22E-62	postive
SCAF11	AC106037.3	0.543536877	1.03E-37	postive
SCAF11	AC073288.2	0.440798528	6.62E-24	postive
GSDME	ATP2B1-AS1	0.498484906	4.44E-31	postive
IL1A	ATP2B1-AS1	0.462783184	1.76E-26	postive
IL1B	ATP2B1-AS1	0.55986742	2.24E-40	postive
NLRC4	ATP2B1-AS1	0.429924706	1.06E-22	postive
NLRP3	ATP2B1-AS1	0.558184664	4.29E-40	postive
SCAF11	ATP2B1-AS1	0.450626639	4.93E-25	postive
HMGB1	AL139089.1	0.488647426	9.32E-30	postive

Pyroptosis-related Genes	lncRNA	cor	pvalue	Regulation
GSDME	AC002044.1	0.500560357	2.31E-31	postive
NLRC4	AC002044.1	0.407518465	2.38E-20	postive
PJVK	AC002044.1	0.423830543	4.82E-22	postive
SCAF11	AC002044.1	0.554454478	1.78E-39	postive
SCAF11	AC006960.4	0.462762612	1.77E-26	postive
GSDME	AC025280.1	0.58758994	3.01E-45	postive
IL1A	AC025280.1	0.569059627	6.12E-42	postive
IL1B	AC025280.1	0.685333304	7.19E-67	postive
NLRC4	AC025280.1	0.513609324	3.39E-33	postive
NLRP3	AC025280.1	0.516480689	1.31E-33	postive
SCAF11	AC025280.1	0.544401471	7.52E-38	postive
PJVK	LINC02027	0.401697242	9.09E-20	postive
CHMP2A	AC020663.3	-0.412087786	8.15E-21	negative
GSDME	AC020663.3	0.542859976	1.32E-37	postive
IL1A	AC020663.3	0.43412976	3.67E-23	postive
IL1B	AC020663.3	0.455029666	1.50E-25	postive
NLRC4	AC020663.3	0.432059858	6.21E-23	postive
NLRP3	AC020663.3	0.459374362	4.55E-26	postive
SCAF11	AC020663.3	0.637943204	2.08E-55	postive
SCAF11	Z98884.1	0.436824758	1.85E-23	postive
GSDME	AL031666.1	0.45749412	7.64E-26	postive
NOD1	AL031666.1	0.41198444	8.36E-21	postive
PJVK	AL031666.1	0.432489279	5.57E-23	postive
SCAF11	AL031666.1	0.645784437	3.65E-57	postive
SCAF11	AC024560.3	0.425422481	3.26E-22	postive
CHMP2A	AC012181.2	-0.445315842	2.03E-24	negative
GSDME	AC012181.2	0.513153346	3.94E-33	postive
IL1A	AC012181.2	0.475038038	5.35E-28	postive
IL1B	AC012181.2	0.552820376	3.30E-39	postive
NLRC4	AC012181.2	0.577409169	2.10E-43	postive
NLRP3	AC012181.2	0.609966666	1.54E-49	postive
SCAF11	AC012181.2	0.663613419	2.35E-61	postive
GSDME	SPON1-AS1	0.428525765	1.51E-22	postive
SCAF11	SPON1-AS1	0.453519531	2.26E-25	postive
GSDME	AC008537.2	0.628974152	1.84E-53	postive
IL1A	AC008537.2	0.44810621	9.68E-25	postive
IL1B	AC008537.2	0.480581365	1.05E-28	postive
NLRC4	AC008537.2	0.494771404	1.42E-30	postive

Pyroptosis-related Genes	lncRNA	cor	pvalue	Regulation
NLRP3	AC008537.2	0.438858538	1.09E-23	postive
NOD1	AC008537.2	0.419334692	1.44E-21	postive
SCAF11	AC008537.2	0.643644683	1.11E-56	postive
SCAF11	AC138956.2	0.493900532	1.86E-30	postive
TIRAP	AC138956.2	0.404056609	5.30E-20	postive
CHMP2A	AL391834.1	-0.421268651	9.02E-22	negative
CHMP6	AL391834.1	-0.414937169	4.15E-21	negative
GSDME	AL391834.1	0.418310751	1.85E-21	postive
PJVK	AL391834.1	0.520614216	3.26E-34	postive
SCAF11	AL391834.1	0.712606176	1.62E-74	postive
SCAF11	AC246817.1	0.442987756	3.74E-24	postive
PJVK	AL139123.1	0.454470397	1.75E-25	postive
GSDME	ODF2-AS1	0.440296588	7.54E-24	postive
SCAF11	ODF2-AS1	0.562675328	7.56E-41	postive
CHMP2A	AL133243.3	-0.438456501	1.21E-23	negative
CHMP6	AL133243.3	-0.415283657	3.82E-21	negative
GSDME	AL133243.3	0.564560558	3.62E-41	postive
IL1A	AL133243.3	0.4182226	1.89E-21	postive
IL1B	AL133243.3	0.416025051	3.20E-21	postive
NLRC4	AL133243.3	0.50330233	9.66E-32	postive
NOD1	AL133243.3	0.40273615	7.17E-20	postive
PJVK	AL133243.3	0.419201781	1.49E-21	postive
SCAF11	AL133243.3	0.758426198	1.32E-89	postive
GSDME	AC108463.3	0.65449677	3.54E-59	postive
IL1A	AC108463.3	0.47121349	1.62E-27	postive
IL1B	AC108463.3	0.490313171	5.61E-30	postive
NLRC4	AC108463.3	0.521368729	2.52E-34	postive
NLRP3	AC108463.3	0.467904727	4.17E-27	postive
NOD1	AC108463.3	0.4142314	4.91E-21	postive
SCAF11	AC108463.3	0.642152139	2.41E-56	postive
CHMP2A	PAXBP1-AS1	-0.45198143	3.43E-25	negative
GSDME	PAXBP1-AS1	0.572694261	1.43E-42	postive
NLRC4	PAXBP1-AS1	0.50879459	1.64E-32	postive
NOD1	PAXBP1-AS1	0.44206598	4.76E-24	postive
PJVK	PAXBP1-AS1	0.498351097	4.64E-31	postive
SCAF11	PAXBP1-AS1	0.740885189	1.88E-83	postive
GSDME	AC009148.1	0.416541901	2.83E-21	postive
NOD1	AC009148.1	0.45442635	1.77E-25	postive

Pyroptosis-related Genes	lncRNA	cor	pvalue	Regulation
PJVK	AC009148.1	0.431410175	7.31E-23	postive
SCAF11	AC009148.1	0.504067581	7.56E-32	postive
PJVK	AC124045.1	0.485847603	2.18E-29	postive
SCAF11	AC124045.1	0.53024431	1.19E-35	postive
GSDME	AL139081.1	0.500010092	2.75E-31	postive
NLRC4	AL139081.1	0.407072411	2.64E-20	postive
NOD1	AL139081.1	0.488950156	8.50E-30	postive
SCAF11	AL139081.1	0.522828449	1.54E-34	postive
GSDME	AC125437.1	0.499453097	3.28E-31	postive
IL1A	AC125437.1	0.45669711	9.51E-26	postive
IL1B	AC125437.1	0.512391295	5.07E-33	postive
NLRC4	AC125437.1	0.458874893	5.22E-26	postive
NLRP3	AC125437.1	0.463502269	1.44E-26	postive
SCAF11	AC125437.1	0.614158293	2.22E-50	postive
GSDME	AL442128.2	0.471295516	1.58E-27	postive
NOD1	AL442128.2	0.498479294	4.45E-31	postive
PJVK	AL442128.2	0.452011795	3.40E-25	postive
SCAF11	AL442128.2	0.573022015	1.25E-42	postive
CHMP2A	AC025031.4	-0.409742128	1.42E-20	negative
GSDME	AC025031.4	0.51054729	9.28E-33	postive
NLRC4	AC025031.4	0.477658676	2.49E-28	postive
PJVK	AC025031.4	0.480982722	9.33E-29	postive
SCAF11	AC025031.4	0.733296661	6.07E-81	postive
CASP3	AC027601.2	-0.415489689	3.63E-21	negative
CHMP2B	AC027601.2	-0.436331846	2.09E-23	negative
CHMP2A	AC108058.1	-0.410727547	1.12E-20	negative
GSDME	AC108058.1	0.472370667	1.16E-27	postive
NLRC4	AC108058.1	0.426141378	2.73E-22	postive
SCAF11	AC108058.1	0.643418935	1.25E-56	postive
CHMP2A	AC018557.2	-0.41731883	2.34E-21	negative
GSDME	AC018557.2	0.565062027	2.97E-41	postive
IL1A	AC018557.2	0.463844235	1.31E-26	postive
IL1B	AC018557.2	0.489066838	8.20E-30	postive
NLRC4	AC018557.2	0.483406562	4.53E-29	postive
NLRP3	AC018557.2	0.416206031	3.06E-21	postive
SCAF11	AC018557.2	0.710560253	6.51E-74	postive
GSDME	AC010320.4	0.442287588	4.49E-24	postive
NOD1	AC010320.4	0.454003916	1.98E-25	postive

Pyroptosis-related Genes	lncRNA	cor	pvalue	Regulation
SCAF11	AC010320.4	0.560964444	1.47E-40	postive
GSDME	AL157395.1	0.514824317	2.27E-33	postive
IL1A	AL157395.1	0.463679393	1.37E-26	postive
IL1B	AL157395.1	0.54771154	2.22E-38	postive
NLRC4	AL157395.1	0.430256695	9.77E-23	postive
NLRP3	AL157395.1	0.400498567	1.19E-19	postive
SCAF11	AL157395.1	0.520854617	3.00E-34	postive
PJVK	AP006545.1	0.536969766	1.11E-36	postive
SCAF11	AP006545.1	0.418575532	1.73E-21	postive
GSDME	AL138820.1	0.496589802	8.05E-31	postive
NLRC4	AL138820.1	0.432185655	6.01E-23	postive
NOD1	AL138820.1	0.453037962	2.57E-25	postive
SCAF11	AL138820.1	0.665983625	6.19E-62	postive
GSDME	LINC02611	0.528684016	2.05E-35	postive
GSDMC	LINC02611	0.425262196	3.39E-22	postive
NLRC4	LINC02611	0.639861776	7.82E-56	postive
NLRP1	LINC02611	0.463568673	1.42E-26	postive
CHMP4B	AC009403.1	0.405218022	4.05E-20	postive
GSDME	AC020915.3	0.431734418	6.74E-23	postive
SCAF11	AC020915.3	0.456613504	9.73E-26	postive
GSDME	AC007128.2	0.402387308	7.77E-20	postive
NOD1	AC007128.2	0.483581957	4.30E-29	postive
SCAF11	AC007128.2	0.471536827	1.47E-27	postive
NOD1	AP000553.2	0.411037577	1.04E-20	postive
PJVK	AP000553.2	0.505389281	4.95E-32	postive
GSDME	SIDT1-AS1	0.446012991	1.69E-24	postive
IL1B	SIDT1-AS1	0.533720565	3.51E-36	postive
NLRC4	SIDT1-AS1	0.439555362	9.13E-24	postive
SCAF11	SIDT1-AS1	0.455614085	1.28E-25	postive
NLRC4	LINC02362	0.408945055	1.71E-20	postive
SCAF11	AL031710.2	0.531530227	7.58E-36	postive
NLRC4	AC110995.1	0.430157971	1.00E-22	postive
SCAF11	AC011330.2	0.512294059	5.23E-33	postive
IRF1	AC138466.1	0.420550088	1.07E-21	postive
GSDME	AC005540.1	0.462691022	1.81E-26	postive
NLRP3	AC005540.1	0.424333755	4.26E-22	postive
NOD1	AC005540.1	0.477106201	2.93E-28	postive
SCAF11	AC005540.1	0.578116424	1.57E-43	postive

Pyroptosis-related Genes	lncRNA	cor	pvalue	Regulation
GSDME	AL359715.2	0.546381761	3.63E-38	postive
IL1A	AL359715.2	0.45420165	1.88E-25	postive
IL1B	AL359715.2	0.546616891	3.33E-38	postive
NLRC4	AL359715.2	0.470639089	1.91E-27	postive
SCAF11	AL359715.2	0.601429409	7.33E-48	postive
CHMP4A	AC090589.3	0.409505392	1.50E-20	postive
SCAF11	AC090589.3	0.478297414	2.06E-28	postive
GSDME	AC018682.1	0.589399182	1.39E-45	postive
IL1A	AC018682.1	0.566618526	1.61E-41	postive
IL1B	AC018682.1	0.673626046	7.71E-64	postive
NLRC4	AC018682.1	0.491081357	4.43E-30	postive
NLRP3	AC018682.1	0.542374424	1.58E-37	postive
SCAF11	AC018682.1	0.559646737	2.44E-40	postive
SCAF11	AL138999.1	0.456443893	1.02E-25	postive
PJVK	LINC02175	0.564949949	3.11E-41	postive
SCAF11	LINC02175	0.469412325	2.71E-27	postive
CHMP2A	NDUFV2-AS1	-0.418950261	1.58E-21	negative
GSDME	NDUFV2-AS1	0.510012571	1.11E-32	postive
IL1A	NDUFV2-AS1	0.431384029	7.36E-23	postive
IL1B	NDUFV2-AS1	0.474393772	6.46E-28	postive
NLRC4	NDUFV2-AS1	0.430304932	9.65E-23	postive
NLRP3	NDUFV2-AS1	0.446837769	1.36E-24	postive
SCAF11	NDUFV2-AS1	0.612233503	5.42E-50	postive
CHMP2A	AC026356.1	-0.433689603	4.11E-23	negative
GSDME	AC026356.1	0.57555557	4.47E-43	postive
IL1A	AC026356.1	0.435296573	2.73E-23	postive
IL1B	AC026356.1	0.424835625	3.76E-22	postive
NLRC4	AC026356.1	0.45641101	1.03E-25	postive
PJVK	AC026356.1	0.433001566	4.89E-23	postive
SCAF11	AC026356.1	0.695066242	1.68E-69	postive
SCAF11	AC012640.1	0.418797601	1.64E-21	postive
GSDME	AC005479.2	0.425634344	3.09E-22	postive
SCAF11	AC005479.2	0.506236947	3.76E-32	postive
GSDME	PRR34	0.487310177	1.40E-29	postive
NLRC4	PRR34	0.401063121	1.05E-19	postive
PJVK	PRR34	0.525008725	7.29E-35	postive
SCAF11	PRR34	0.415001464	4.08E-21	postive
PJVK	TTC28-AS1	0.550713317	7.27E-39	postive

Pyroptosis-related Genes	lncRNA	cor	pvalue	Regulation
PJVK	MRPS9-AS1	0.509364409	1.37E-32	postive
SCAF11	MRPS9-AS1	0.539314168	4.77E-37	postive
PJVK	AL133410.1	0.480390748	1.11E-28	postive
GSDME	GORAB-AS1	0.621946279	5.59E-52	postive
IL1A	GORAB-AS1	0.476860477	3.14E-28	postive
IL1B	GORAB-AS1	0.549764663	1.04E-38	postive
NLRC4	GORAB-AS1	0.577757479	1.82E-43	postive
NLRP3	GORAB-AS1	0.517801636	8.40E-34	postive
SCAF11	GORAB-AS1	0.501505531	1.71E-31	postive
GSDME	AL050320.1	0.508181716	2.01E-32	postive
SCAF11	AL050320.1	0.594631088	1.46E-46	postive
GSDME	LPP-AS1	0.597812779	3.63E-47	postive
IL1A	LPP-AS1	0.470422313	2.03E-27	postive
IL1B	LPP-AS1	0.492261285	3.08E-30	postive
NLRC4	LPP-AS1	0.495181058	1.25E-30	postive
NLRP3	LPP-AS1	0.425266884	3.38E-22	postive
SCAF11	LPP-AS1	0.669611022	7.85E-63	postive
GSDME	AC011676.1	0.460320258	3.50E-26	postive
IL1B	AC011676.1	0.473063052	9.49E-28	postive
NLRP3	AC011676.1	0.464095652	1.22E-26	postive
SCAF11	AC011676.1	0.494660564	1.47E-30	postive
NOD1	HCG27	0.515576859	1.77E-33	postive
ELANE	FENDRR	0.437883558	1.41E-23	postive
CHMP6	AC110769.2	-0.402058224	8.37E-20	negative
GSDME	AC110769.2	0.54007129	3.63E-37	postive
NLRC4	AC110769.2	0.439899908	8.35E-24	postive
PJVK	AC110769.2	0.440876395	6.48E-24	postive
SCAF11	AC110769.2	0.675326535	2.86E-64	postive
GSDME	AL080317.1	0.564136817	4.27E-41	postive
IL1A	AL080317.1	0.415409934	3.70E-21	postive
IL1B	AL080317.1	0.439147296	1.02E-23	postive
NLRC4	AL080317.1	0.495439916	1.15E-30	postive
PJVK	AL080317.1	0.445328192	2.02E-24	postive
SCAF11	AL080317.1	0.701492679	2.70E-71	postive
CHMP2A	AC009065.3	0.493110206	2.37E-30	postive
CHMP4B	AC009065.3	0.489270904	7.71E-30	postive
PYCARD	AC009065.3	0.460608139	3.23E-26	postive
SCAF11	AC009065.3	-0.40905516	1.66E-20	negative

Pyroptosis-related Genes	lncRNA	cor	pvalue	Regulation
CHMP2A	AC009095.1	-0.438961637	1.06E-23	negative
GSDME	AC009095.1	0.555990108	9.92E-40	postive
IL1B	AC009095.1	0.401149471	1.03E-19	postive
NLRC4	AC009095.1	0.507932108	2.18E-32	postive
NOD1	AC009095.1	0.421450791	8.63E-22	postive
PJVK	AC009095.1	0.456802077	9.24E-26	postive
SCAF11	AC009095.1	0.725910518	1.40E-78	postive
CHMP2A	RHOA-IT1	-0.439094263	1.03E-23	negative
CHMP6	RHOA-IT1	-0.402188287	8.13E-20	negative
GSDME	RHOA-IT1	0.534100297	3.07E-36	postive
IL1A	RHOA-IT1	0.439952243	8.24E-24	postive
IL1B	RHOA-IT1	0.50572057	4.45E-32	postive
NLRC4	RHOA-IT1	0.478275142	2.08E-28	postive
NLRP3	RHOA-IT1	0.439534808	9.18E-24	postive
SCAF11	RHOA-IT1	0.669184664	1.00E-62	postive
PJVK	HMGN3-AS1	0.494504756	1.54E-30	postive
SCAF11	HMGN3-AS1	0.431281762	7.55E-23	postive
PJVK	AC025175.1	0.45327067	2.42E-25	postive
SCAF11	AC025175.1	0.58643579	4.90E-45	postive
GSDME	AC093788.1	0.48377304	4.06E-29	postive
NLRC4	AC093788.1	0.430846031	8.43E-23	postive
PJVK	AC093788.1	0.544516686	7.21E-38	postive
SCAF11	AC093788.1	0.6780478	5.74E-65	postive
GSDME	LINC00987	0.563791644	4.89E-41	postive
NLRC4	LINC00987	0.580229236	6.57E-44	postive
NLRP1	LINC00987	0.44178136	5.12E-24	postive
NLRP3	LINC00987	0.458194007	6.30E-26	postive
PJVK	AC116552.1	0.400164848	1.29E-19	postive
GSDME	AL353801.1	0.453671721	2.17E-25	postive
NOD1	AL353801.1	0.479951217	1.27E-28	postive
SCAF11	AL353801.1	0.528268608	2.37E-35	postive
CHMP2A	AP001001.1	-0.434204897	3.60E-23	negative
GSDME	AP001001.1	0.52901105	1.83E-35	postive
NLRC4	AP001001.1	0.458801516	5.33E-26	postive
PJVK	AP001001.1	0.478451185	1.97E-28	postive
SCAF11	AP001001.1	0.726078188	1.24E-78	postive
GSDME	AP001434.1	0.70663179	9.12E-73	postive
IL1A	AP001434.1	0.699451434	1.01E-70	postive

Pyroptosis-related Genes	lncRNA	cor	pvalue	Regulation
IL1B	AP001434.1	0.848915766	1.59E-132	postive
GSDMC	AP001434.1	0.446528937	1.47E-24	postive
NLRC4	AP001434.1	0.582813708	2.25E-44	postive
NLRP3	AP001434.1	0.822482548	1.71E-117	postive
SCAF11	AP001434.1	0.421018624	9.58E-22	postive
NOD1	LENG8-AS1	0.410660586	1.14E-20	postive
PLCG1	LENG8-AS1	0.496070343	9.47E-31	postive
GSDME	AC021851.1	0.410588537	1.16E-20	postive
PJVK	AC021851.1	0.55204567	4.41E-39	postive
SCAF11	AC021851.1	0.652156669	1.25E-58	postive
GSDME	MANEA-DT	0.544793518	6.51E-38	postive
NLRC4	MANEA-DT	0.466793894	5.71E-27	postive
NOD1	MANEA-DT	0.401328283	9.89E-20	postive
PJVK	MANEA-DT	0.55292556	3.17E-39	postive
SCAF11	MANEA-DT	0.633832908	1.65E-54	postive
GSDME	ZNF32-AS2	0.403603929	5.88E-20	postive
NOD1	ZNF32-AS2	0.41374824	5.50E-21	postive
PJVK	ZNF32-AS2	0.467737793	4.37E-27	postive
SCAF11	ZNF32-AS2	0.5866893	4.41E-45	postive
SCAF11	AC005089.1	0.400113738	1.30E-19	postive
GSDME	AL138689.1	0.516858555	1.15E-33	postive
IL1B	AL138689.1	0.489222701	7.82E-30	postive
NLRC4	AL138689.1	0.488436283	9.94E-30	postive
SCAF11	AL138689.1	0.536617898	1.25E-36	postive
PJVK	AL356481.3	0.455422892	1.35E-25	postive
GSDME	AC074050.4	0.419867837	1.27E-21	postive
NLRP3	AC074050.4	0.403771233	5.66E-20	postive
SCAF11	AC074050.4	0.615151117	1.40E-50	postive
CHMP2A	AC010834.3	-0.401626993	9.24E-20	negative
GSDME	AC010834.3	0.530412887	1.12E-35	postive
NLRC4	AC010834.3	0.443389748	3.36E-24	postive
NOD1	AC010834.3	0.427398977	2.00E-22	postive
PJVK	AC010834.3	0.540024856	3.69E-37	postive
SCAF11	AC010834.3	0.689777039	4.66E-68	postive
PJVK	AC007405.2	0.428448475	1.54E-22	postive
GSDME	PCED1B-AS1	0.537681298	8.57E-37	postive
NLRC4	PCED1B-AS1	0.635822411	6.09E-55	postive
NLRP1	PCED1B-AS1	0.607265495	5.30E-49	postive

Pyroptosis-related Genes	lncRNA	cor	pvalue	Regulation
NLRP3	PCED1B-AS1	0.516230735	1.42E-33	postive
TNF	PCED1B-AS1	0.450325727	5.35E-25	postive
SCAF11	AC127024.3	0.420358864	1.12E-21	postive
GSDME	AC124312.2	0.480714113	1.01E-28	postive
SCAF11	AC124312.2	0.483844629	3.97E-29	postive
GSDME	AC211476.4	0.431109429	7.89E-23	postive
SCAF11	AC211476.4	0.57735532	2.15E-43	postive
PJVK	AC007608.1	0.420038058	1.22E-21	postive
SCAF11	AC007608.1	0.464848536	9.89E-27	postive
GSDME	Z99289.3	0.541400588	2.24E-37	postive
IL1A	Z99289.3	0.407344125	2.48E-20	postive
IL1B	Z99289.3	0.40137028	9.79E-20	postive
NLRC4	Z99289.3	0.456607276	9.74E-26	postive
PJVK	Z99289.3	0.471143082	1.65E-27	postive
SCAF11	Z99289.3	0.614285949	2.09E-50	postive
NOD1	AC060780.1	0.428680662	1.45E-22	postive
PJVK	AC060780.1	0.447521108	1.13E-24	postive
SCAF11	AC060780.1	0.470219358	2.15E-27	postive
CHMP2A	AL136115.1	-0.439941816	8.26E-24	negative
CHMP6	AL136115.1	-0.406689953	2.88E-20	negative
GSDME	AL136115.1	0.541296207	2.33E-37	postive
IL1A	AL136115.1	0.490854438	4.75E-30	postive
IL1B	AL136115.1	0.542590397	1.46E-37	postive
NLRC4	AL136115.1	0.459675105	4.19E-26	postive
NLRP3	AL136115.1	0.516838163	1.16E-33	postive
SCAF11	AL136115.1	0.676088166	1.83E-64	postive
GSDME	AC009032.1	0.504499648	6.58E-32	postive
NLRC4	AC009032.1	0.457602677	7.42E-26	postive
PJVK	AC009032.1	0.44791548	1.02E-24	postive
SCAF11	AC009032.1	0.643044644	1.52E-56	postive
PJVK	SMILR	0.405801357	3.54E-20	postive
GSDME	AL137003.1	0.44720715	1.23E-24	postive
PJVK	AL137003.1	0.480792691	9.87E-29	postive
SCAF11	AL137003.1	0.578435743	1.38E-43	postive
NOD1	LINC00954	0.499220319	3.53E-31	postive
CHMP4A	AL049780.1	0.429591784	1.15E-22	postive
GSDME	AL049780.1	0.470205536	2.16E-27	postive
NLRC4	AL049780.1	0.40885906	1.74E-20	postive

Pyroptosis-related Genes	lncRNA	cor	pvalue	Regulation
PJVK	AL049780.1	0.442019513	4.81E-24	postive
SCAF11	AL049780.1	0.596771723	5.74E-47	postive
NOD1	MORF4L2-AS1	0.463192665	1.57E-26	postive
PJVK	MORF4L2-AS1	0.542856576	1.32E-37	postive
SCAF11	MORF4L2-AS1	0.53090164	9.45E-36	postive
GSDME	AC004466.2	0.559283562	2.81E-40	postive
IL1A	AC004466.2	0.412925282	6.69E-21	postive
IL1B	AC004466.2	0.435252911	2.76E-23	postive
NLRC4	AC004466.2	0.488850729	8.76E-30	postive
NLRP3	AC004466.2	0.457403359	7.83E-26	postive
SCAF11	AC004466.2	0.654646963	3.27E-59	postive
NOD1	LINC02569	0.441979105	4.87E-24	postive
PJVK	LINC02569	0.448485026	8.75E-25	postive
SCAF11	LINC02569	0.431402188	7.33E-23	postive
PJVK	AC008635.2	0.408500106	1.89E-20	postive
SCAF11	AC008635.2	0.408304478	1.98E-20	postive
NOD1	AC007128.1	0.560135153	2.02E-40	postive
PJVK	AC007128.1	0.445445012	1.96E-24	postive
SCAF11	AC007128.1	0.509163262	1.46E-32	postive
GSDME	PLS1-AS1	0.421504285	8.51E-22	postive
PJVK	PLS1-AS1	0.424096294	4.51E-22	postive
SCAF11	PLS1-AS1	0.691490122	1.60E-68	postive
CHMP2A	AC132192.1	-0.458498171	5.80E-26	negative
CHMP6	AC132192.1	-0.423511251	5.21E-22	negative
GSDME	AC132192.1	0.477242906	2.81E-28	postive
NLRC4	AC132192.1	0.463546554	1.43E-26	postive
SCAF11	AC132192.1	0.716698356	9.64E-76	postive
NLRC4	AC120049.1	0.41893182	1.59E-21	postive
HMGB1	AL354696.1	0.451900726	3.50E-25	postive
SCAF11	AL354696.1	0.485614896	2.34E-29	postive
GSDME	RSF1-IT1	0.567758266	1.03E-41	postive
IL1A	RSF1-IT1	0.403231704	6.40E-20	postive
IL1B	RSF1-IT1	0.440715274	6.76E-24	postive
NLRC4	RSF1-IT1	0.520809659	3.05E-34	postive
PJVK	RSF1-IT1	0.423497197	5.23E-22	postive
SCAF11	RSF1-IT1	0.716591753	1.04E-75	postive
GSDME	AL022157.1	0.423721869	4.95E-22	postive
PJVK	AL022157.1	0.413222381	6.23E-21	postive

Pyroptosis-related Genes	lncRNA	cor	pvalue	Regulation
SCAF11	AL022157.1	0.613669817	2.79E-50	postive
GSDME	LINC00622	0.523975096	1.04E-34	postive
IL1B	LINC00622	0.50573498	4.43E-32	postive
NLRC4	LINC00622	0.476915157	3.10E-28	postive
SCAF11	LINC00622	0.428859987	1.39E-22	postive
CHMP2A	AC011815.1	-0.409110914	1.64E-20	negative
GSDME	AC011815.1	0.50291625	1.09E-31	postive
IL1A	AC011815.1	0.420888239	9.89E-22	postive
IL1B	AC011815.1	0.46776071	4.34E-27	postive
CASP8	AC011815.1	0.405460407	3.83E-20	postive
NLRC4	AC011815.1	0.487701328	1.24E-29	postive
NLRP3	AC011815.1	0.402547917	7.49E-20	postive
SCAF11	AC011815.1	0.667926768	2.05E-62	postive
PJVK	AC007497.1	0.406852886	2.78E-20	postive
SCAF11	AC007497.1	0.528083577	2.52E-35	postive
GSDME	AL031667.3	0.470573754	1.94E-27	postive
PJVK	AL031667.3	0.492354743	3.00E-30	postive
SCAF11	AL031667.3	0.670472705	4.78E-63	postive
CHMP2A	OIP5-AS1	-0.580779388	5.23E-44	negative
CHMP4B	OIP5-AS1	-0.463314336	1.52E-26	negative
CHMP6	OIP5-AS1	-0.450782674	4.73E-25	negative
GSDMD	OIP5-AS1	-0.404160351	5.17E-20	negative
GSDME	OIP5-AS1	0.452681627	2.84E-25	postive
CASP8	OIP5-AS1	0.455340676	1.38E-25	postive
GPX4	OIP5-AS1	-0.459663658	4.20E-26	negative
NLRC4	OIP5-AS1	0.489538254	7.10E-30	postive
SCAF11	OIP5-AS1	0.732402257	1.18E-80	postive
GSDME	AL049539.1	0.486989734	1.54E-29	postive
PJVK	AL049539.1	0.468357673	3.66E-27	postive
SCAF11	AL049539.1	0.679024493	3.22E-65	postive
PJVK	LINC01765	0.413616177	5.68E-21	postive
GSDME	AC013403.2	0.401104937	1.04E-19	postive
SCAF11	AC013403.2	0.532507386	5.38E-36	postive
PJVK	AC012184.3	0.492182605	3.16E-30	postive
GSDME	TTC3-AS1	0.510934781	8.18E-33	postive
NLRC4	TTC3-AS1	0.480167479	1.19E-28	postive
PJVK	TTC3-AS1	0.460376351	3.45E-26	postive
SCAF11	TTC3-AS1	0.672559738	1.43E-63	postive

Pyroptosis-related Genes	lncRNA	cor	pvalue	Regulation
GSDME	AC011472.4	0.520973835	2.89E-34	postive
IL1A	AC011472.4	0.401093496	1.04E-19	postive
SCAF11	AC011472.4	0.661416134	7.99E-61	postive
PJVK	AC008115.4	0.458566894	5.69E-26	postive
CHMP4A	AL928654.2	0.51262667	4.69E-33	postive
GSDME	LINC01614	0.657363273	7.46E-60	postive
NLRC4	LINC01614	0.58142468	4.00E-44	postive
NLRP3	LINC01614	0.546141383	3.97E-38	postive
GSDME	AL445309.1	0.513248729	3.82E-33	postive
NLRC4	AL445309.1	0.449148423	7.33E-25	postive
NOD1	AL445309.1	0.410343226	1.23E-20	postive
SCAF11	AL445309.1	0.604246338	2.08E-48	postive
CHMP2A	AL596247.1	-0.413636424	5.65E-21	negative
GSDME	AL596247.1	0.502491663	1.25E-31	postive
IL1B	AL596247.1	0.410810317	1.10E-20	postive
NLRC4	AL596247.1	0.467704548	4.41E-27	postive
PJVK	AL596247.1	0.407279461	2.52E-20	postive
SCAF11	AL596247.1	0.719978102	9.69E-77	postive
CHMP2A	UGDH-AS1	-0.433352471	4.48E-23	negative
GSDME	UGDH-AS1	0.485729176	2.26E-29	postive
IL1B	UGDH-AS1	0.420952635	9.74E-22	postive
SCAF11	UGDH-AS1	0.580518875	5.83E-44	postive
GSDME	AP005899.1	0.433217371	4.63E-23	postive
IL1A	AP005899.1	0.423273745	5.52E-22	postive
IL1B	AP005899.1	0.490438847	5.39E-30	postive
NLRC4	AP005899.1	0.403891991	5.50E-20	postive
NLRP3	AP005899.1	0.524820633	7.78E-35	postive
SCAF11	AP005899.1	0.577303683	2.19E-43	postive
GSDME	AL033504.1	0.425224047	3.42E-22	postive
IL1B	AL033504.1	0.448064737	9.78E-25	postive
NLRC4	AL033504.1	0.418342399	1.83E-21	postive
SCAF11	AL033504.1	0.638570918	1.51E-55	postive
CHMP2A	KLF7-IT1	-0.407994925	2.13E-20	negative
GSDME	KLF7-IT1	0.598057378	3.26E-47	postive
IL1A	KLF7-IT1	0.482816391	5.41E-29	postive
IL1B	KLF7-IT1	0.506730878	3.21E-32	postive
NLRC4	KLF7-IT1	0.503802528	8.23E-32	postive
NLRP3	KLF7-IT1	0.43604117	2.26E-23	postive

Pyroptosis-related Genes	lncRNA	cor	pvalue	Regulation
PJVK	KLF7-IT1	0.401089508	1.04E-19	postive
SCAF11	KLF7-IT1	0.688926889	7.90E-68	postive
GSDME	AL121772.2	0.43453892	3.31E-23	postive
NOD1	AL121772.2	0.421504606	8.51E-22	postive
SCAF11	AL121772.2	0.545945287	4.27E-38	postive
CHMP2A	AC103740.1	-0.41445387	4.65E-21	negative
CHMP6	AC103740.1	-0.404906446	4.36E-20	negative
GSDME	AC103740.1	0.547504671	2.40E-38	postive
IL1B	AC103740.1	0.403355037	6.22E-20	postive
NLRC4	AC103740.1	0.439364763	9.60E-24	postive
NLRP3	AC103740.1	0.427021217	2.19E-22	postive
SCAF11	AC103740.1	0.667010825	3.46E-62	postive
SCAF11	AC007608.2	0.450562621	5.02E-25	postive
CHMP2A	AC058791.1	-0.414380289	4.73E-21	negative
CHMP6	AC058791.1	-0.402284016	7.95E-20	negative
GSDME	AC058791.1	0.452951612	2.64E-25	postive
PJVK	AC058791.1	0.415902577	3.29E-21	postive
SCAF11	AC058791.1	0.697027735	4.82E-70	postive
SCAF11	ITGA9-AS1	0.400790909	1.12E-19	postive
GSDME	AC022778.1	0.529312432	1.65E-35	postive
IL1A	AC022778.1	0.459845567	3.99E-26	postive
IL1B	AC022778.1	0.581641795	3.66E-44	postive
NLRC4	AC022778.1	0.505216353	5.23E-32	postive
SCAF11	AC022778.1	0.553726951	2.34E-39	postive
SCAF11	AL132642.1	0.473787208	7.70E-28	postive
GSDME	AL391832.3	0.496275808	8.88E-31	postive
IL1A	AL391832.3	0.50874814	1.67E-32	postive
IL1B	AL391832.3	0.619259021	2.01E-51	postive
NLRC4	AL391832.3	0.474909942	5.56E-28	postive
NLRP3	AL391832.3	0.544546856	7.13E-38	postive
AIM2	LINC00941	0.429682037	1.13E-22	postive
GSDME	AC007663.4	0.499068067	3.70E-31	postive
NLRP3	AC007663.4	0.430209967	9.89E-23	postive
SCAF11	AC007663.4	0.555815974	1.06E-39	postive
GSDME	AC019186.1	0.561898012	1.02E-40	postive
IL1A	AC019186.1	0.41225185	7.84E-21	postive
IL1B	AC019186.1	0.407736223	2.26E-20	postive
NLRC4	AC019186.1	0.458166722	6.35E-26	postive

Pyroptosis-related Genes	lncRNA	cor	pvalue	Regulation
NOD1	AC019186.1	0.405150073	4.12E-20	postive
SCAF11	AC019186.1	0.652947515	8.17E-59	postive
PJVK	STAM-AS1	0.588253334	2.27E-45	postive
SCAF11	STAM-AS1	0.437151459	1.70E-23	postive
GSDME	LINC01050	0.625087414	1.23E-52	postive
IL1A	LINC01050	0.452084412	3.33E-25	postive
IL1B	LINC01050	0.598041381	3.28E-47	postive
GSDMC	LINC01050	0.401818291	8.84E-20	postive
NLRC4	LINC01050	0.557470296	5.64E-40	postive
NLRP3	LINC01050	0.459125523	4.87E-26	postive
SCAF11	LINC01050	0.469311285	2.79E-27	postive
CHMP7	AC100861.1	0.726711296	7.81E-79	postive
GSDME	AC107081.2	0.456665598	9.59E-26	postive
NLRC4	AC107081.2	0.453310097	2.39E-25	postive
PJVK	AC107081.2	0.461843853	2.29E-26	postive
SCAF11	AC107081.2	0.613699157	2.75E-50	postive
PJVK	AC104463.2	0.417156118	2.44E-21	postive
GSDME	AC090197.1	0.567566044	1.11E-41	postive
IL1A	AC090197.1	0.501344083	1.80E-31	postive
IL1B	AC090197.1	0.553993334	2.12E-39	postive
NLRC4	AC090197.1	0.443610132	3.18E-24	postive
NLRP3	AC090197.1	0.555195003	1.34E-39	postive
SCAF11	AC090197.1	0.549476877	1.15E-38	postive
GSDME	MMP2-AS1	0.51538772	1.88E-33	postive
NLRC4	MMP2-AS1	0.526042338	5.11E-35	postive
PJVK	LINC02163	0.416500653	2.85E-21	postive
SCAF11	LINC02163	0.430630495	8.90E-23	postive
GSDME	AL139011.1	0.484774327	3.01E-29	postive
PJVK	AL139011.1	0.438189927	1.30E-23	postive
SCAF11	AL139011.1	0.618766653	2.54E-51	postive
SCAF11	AC092944.1	0.50364087	8.67E-32	postive
PJVK	AL731569.1	0.494057237	1.77E-30	postive
GSDME	AOAH-IT1	0.461861816	2.28E-26	postive
NOD1	AOAH-IT1	0.475976764	4.07E-28	postive
PJVK	AOAH-IT1	0.460530329	3.30E-26	postive
SCAF11	AOAH-IT1	0.592929712	3.05E-46	postive
PJVK	AC017083.1	0.585531753	7.18E-45	postive
CHMP2A	AC095055.1	-0.416551009	2.82E-21	negative

Pyroptosis-related Genes	lncRNA	cor	pvalue	Regulation
GSDME	AC095055.1	0.521220428	2.65E-34	postive
NLRC4	AC095055.1	0.437251038	1.65E-23	postive
PJVK	AC095055.1	0.451244595	4.18E-25	postive
SCAF11	AC095055.1	0.684405857	1.26E-66	postive
PJVK	AL390195.2	0.43436452	3.46E-23	postive
SCAF11	AL390195.2	0.636000843	5.57E-55	postive
GSDME	AC006111.2	0.462650196	1.83E-26	postive
NOD1	AC006111.2	0.415980189	3.23E-21	postive
PJVK	AC006111.2	0.404812546	4.45E-20	postive
SCAF11	AC006111.2	0.538175638	7.18E-37	postive
PJVK	AC011477.2	0.461994753	2.20E-26	postive
SCAF11	AC011477.2	0.589425678	1.38E-45	postive
GSDME	AP000695.2	0.621223369	7.90E-52	postive
IL1B	AP000695.2	0.45418702	1.89E-25	postive
GSDMC	AP000695.2	0.410717366	1.13E-20	postive
NLRC4	AP000695.2	0.4973499	6.35E-31	postive
NLRP3	AP000695.2	0.594404614	1.61E-46	postive
PJVK	AP000350.6	0.533552935	3.72E-36	postive
SCAF11	AP000350.6	0.562899694	6.93E-41	postive
CHMP4A	AP000845.1	0.404971408	4.29E-20	postive
PJVK	AP000845.1	0.451300829	4.11E-25	postive
GSDME	AP002954.1	0.40313369	6.55E-20	postive
NLRC4	AP002954.1	0.424369163	4.22E-22	postive
GSDME	AC084117.1	0.49365264	2.01E-30	postive
IL1A	AC084117.1	0.470122527	2.21E-27	postive
IL1B	AC084117.1	0.562184705	9.15E-41	postive
NLRC4	AC084117.1	0.488791401	8.92E-30	postive
SCAF11	AC084117.1	0.613409798	3.14E-50	postive
NOD1	INTS6-AS1	0.503944653	7.86E-32	postive
PJVK	INTS6-AS1	0.402875063	6.95E-20	postive
SCAF11	INTS6-AS1	0.432000666	6.30E-23	postive
PJVK	TMEM9B-AS1	0.474459038	6.33E-28	postive
IL1B	AL078644.2	0.463281295	1.54E-26	postive
NLRP3	AL078644.2	0.479873272	1.30E-28	postive
CHMP2A	AC011676.2	-0.403504414	6.01E-20	negative
GSDME	AC011676.2	0.490314914	5.60E-30	postive
IL1A	AC011676.2	0.403787076	5.64E-20	postive
IL1B	AC011676.2	0.455799331	1.22E-25	postive

Pyroptosis-related Genes	lncRNA	cor	pvalue	Regulation
NLRC4	AC011676.2	0.40081997	1.11E-19	postive
NLRP3	AC011676.2	0.462964262	1.68E-26	postive
SCAF11	AC011676.2	0.614975259	1.52E-50	postive
CHMP2A	AC009269.5	-0.40165576	9.18E-20	negative
GSDME	AC009269.5	0.579925606	7.45E-44	postive
IL1A	AC009269.5	0.500930629	2.05E-31	postive
IL1B	AC009269.5	0.52983095	1.37E-35	postive
NLRC4	AC009269.5	0.469944715	2.33E-27	postive
NLRP3	AC009269.5	0.440334953	7.46E-24	postive
SCAF11	AC009269.5	0.671568226	2.54E-63	postive
CHMP2A	KDM4A-AS1	-0.41072604	1.12E-20	negative
PJVK	KDM4A-AS1	0.446167455	1.62E-24	postive
SCAF11	KDM4A-AS1	0.534147655	3.02E-36	postive
CHMP4A	AGBL5-IT1	0.438731937	1.13E-23	postive
PJVK	AGBL5-IT1	0.534969423	2.25E-36	postive
CASP6	AC008966.1	0.40509644	4.17E-20	postive
GSDME	AL138963.1	0.53895396	5.43E-37	postive
NLRC4	AL138963.1	0.450117382	5.65E-25	postive
NOD1	AL138963.1	0.423442898	5.30E-22	postive
PJVK	AL138963.1	0.424945495	3.66E-22	postive
SCAF11	AL138963.1	0.666873747	3.74E-62	postive
GSDME	AL365361.1	0.510404088	9.73E-33	postive
IL1A	AL365361.1	0.468850587	3.18E-27	postive
IL1B	AL365361.1	0.550492496	7.90E-39	postive
NLRC4	AL365361.1	0.583991339	1.37E-44	postive
NLRP1	AL365361.1	0.422154718	7.26E-22	postive
NLRP3	AL365361.1	0.610272017	1.34E-49	postive
SCAF11	AL365361.1	0.582827952	2.23E-44	postive
GSDME	AL513327.2	0.551685986	5.05E-39	postive
NLRC4	AL513327.2	0.429718447	1.12E-22	postive
NLRP3	AL513327.2	0.401538748	9.43E-20	postive
NOD1	AL513327.2	0.422076708	7.40E-22	postive
SCAF11	AL513327.2	0.572565058	1.50E-42	postive
CHMP2A	AC040904.1	-0.404020109	5.34E-20	negative
GSDME	AC040904.1	0.540156732	3.52E-37	postive
IL1A	AC040904.1	0.409418586	1.53E-20	postive
IL1B	AC040904.1	0.421879312	7.77E-22	postive
NLRC4	AC040904.1	0.480349848	1.13E-28	postive

Pyroptosis-related Genes	lncRNA	cor	pvalue	Regulation
PJVK	AC040904.1	0.440085271	7.96E-24	postive
SCAF11	AC040904.1	0.722681432	1.42E-77	postive
GSDME	AL109761.1	0.503455608	9.19E-32	postive
NLRC4	AL109761.1	0.400816139	1.11E-19	postive
NOD1	AL109761.1	0.423823181	4.83E-22	postive
SCAF11	AL109761.1	0.611069022	9.29E-50	postive
PJVK	AL390037.1	0.403515107	6.00E-20	postive
PJVK	SPAG5-AS1	0.465824672	7.51E-27	postive
SCAF11	SPAG5-AS1	0.550385143	8.22E-39	postive
GSDME	AC002091.1	0.578195847	1.52E-43	postive
IL1B	AC002091.1	0.480802058	9.84E-29	postive
NLRC4	AC002091.1	0.642974913	1.58E-56	postive
NLRP1	AC002091.1	0.418303656	1.85E-21	postive
NLRP3	AC002091.1	0.601040176	8.71E-48	postive
TNF	AC002091.1	0.439392356	9.53E-24	postive
GSDME	LINC02254	0.447908072	1.02E-24	postive
NLRC4	LINC02254	0.401153963	1.03E-19	postive
SCAF11	LINC02254	0.492942804	2.50E-30	postive
GSDME	AC020661.3	0.558377932	3.98E-40	postive
IL1A	AC020661.3	0.465761852	7.64E-27	postive
IL1B	AC020661.3	0.518671136	6.28E-34	postive
NLRC4	AC020661.3	0.487229749	1.43E-29	postive
SCAF11	AC020661.3	0.630998653	6.79E-54	postive
CHMP2A	AP002026.1	-0.42039187	1.12E-21	negative
GSDME	AP002026.1	0.421593025	8.33E-22	postive
PJVK	AP002026.1	0.525982319	5.22E-35	postive
SCAF11	AP002026.1	0.580685559	5.44E-44	postive
PJVK	AL358072.1	0.567950063	9.51E-42	postive
SCAF11	AL358072.1	0.526306893	4.66E-35	postive
GSDME	AL136988.2	0.588618859	1.94E-45	postive
NLRC4	AL136988.2	0.467245854	5.02E-27	postive
NOD1	AL136988.2	0.487140397	1.47E-29	postive
SCAF11	AL136988.2	0.65303459	7.80E-59	postive
GSDME	AC090753.1	0.407073374	2.64E-20	postive
IL1B	AC090753.1	0.519632618	4.54E-34	postive
NLRC4	AC090753.1	0.418266879	1.87E-21	postive
PJVK	SOX9-AS1	0.415681657	3.47E-21	postive
PJVK	AC036214.2	0.510073003	1.08E-32	postive

Pyroptosis-related Genes	lncRNA	cor	pvalue	Regulation
SCAF11	AC036214.2	0.555690127	1.11E-39	postive
GSDME	AC015853.1	0.441517671	5.49E-24	postive
IL1B	AC015853.1	0.404312083	4.99E-20	postive
SCAF11	AC015853.1	0.456572796	9.84E-26	postive
GSDME	ZBED3-AS1	0.488046975	1.12E-29	postive
NLRC4	ZBED3-AS1	0.426734713	2.35E-22	postive
PJVK	ZBED3-AS1	0.563724875	5.02E-41	postive
SCAF11	ZBED3-AS1	0.600758999	9.87E-48	postive
CHMP4A	AL355388.1	0.411813063	8.70E-21	postive
GSDME	AL355388.1	0.422960549	5.96E-22	postive
NOD1	AL355388.1	0.409234141	1.59E-20	postive
PJVK	AL355388.1	0.445786019	1.79E-24	postive
SCAF11	AL355388.1	0.489059631	8.22E-30	postive
GSDME	LINC01561	0.701970116	1.97E-71	postive
IL1B	LINC01561	0.447501946	1.14E-24	postive
NLRC4	LINC01561	0.440069646	7.99E-24	postive
NLRP3	LINC01561	0.563094593	6.42E-41	postive
NLRP1	FAM30A	0.491773893	3.58E-30	postive
PJVK	AC100814.2	0.521581475	2.35E-34	postive
CHMP4A	AC010761.1	0.408103067	2.08E-20	postive
PJVK	AC010761.1	0.40799581	2.13E-20	postive
SCAF11	AC010761.1	0.459655005	4.21E-26	postive
GSDME	AC008957.1	0.580594418	5.65E-44	postive
IL1B	AC008957.1	0.418871897	1.61E-21	postive
NLRC4	AC008957.1	0.582023023	3.12E-44	postive
SCAF11	AC008957.1	0.581528465	3.84E-44	postive
GSDME	LINC00539	0.451114637	4.33E-25	postive
NOD1	LINC00539	0.541475618	2.18E-37	postive
SCAF11	LINC00539	0.443004851	3.72E-24	postive
GSDME	DLGAP4-AS1	0.484381993	3.38E-29	postive
NOD1	DLGAP4-AS1	0.423255309	5.55E-22	postive
PJVK	DLGAP4-AS1	0.457415183	7.81E-26	postive
SCAF11	DLGAP4-AS1	0.630163256	1.03E-53	postive
GSDME	AC010618.3	0.535677352	1.75E-36	postive
IL1B	AC010618.3	0.403413072	6.14E-20	postive
NLRC4	AC010618.3	0.444974616	2.22E-24	postive
NLRP3	AC010618.3	0.414971771	4.11E-21	postive
NOD1	AC010618.3	0.436444656	2.03E-23	postive

Pyroptosis-related Genes	lncRNA	cor	pvalue	Regulation
PJVK	AC010618.3	0.465860835	7.43E-27	postive
SCAF11	AC010618.3	0.585541154	7.15E-45	postive
GSDME	AC012020.1	0.530108427	1.25E-35	postive
NLRC4	AC012020.1	0.42040701	1.11E-21	postive
NOD1	AC012020.1	0.471749367	1.39E-27	postive
SCAF11	AC012020.1	0.564426169	3.81E-41	postive
PJVK	AC103591.3	0.432079983	6.18E-23	postive
SCAF11	AC103591.3	0.606621929	7.10E-49	postive
GSDME	FSIP2-AS1	0.452836519	2.72E-25	postive
NOD1	FSIP2-AS1	0.490839559	4.77E-30	postive
PJVK	FSIP2-AS1	0.547724045	2.21E-38	postive
SCAF11	FSIP2-AS1	0.574245341	7.62E-43	postive
GSDME	AL358216.1	0.566121176	1.96E-41	postive
IL1A	AL358216.1	0.481270165	8.57E-29	postive
IL1B	AL358216.1	0.486584508	1.74E-29	postive
NLRC4	AL358216.1	0.461876082	2.27E-26	postive
NLRP3	AL358216.1	0.451369228	4.04E-25	postive
PJVK	AL358216.1	0.411609676	9.13E-21	postive
SCAF11	AL358216.1	0.675129075	3.21E-64	postive
GSDME	LINC01615	0.594678344	1.43E-46	postive
NLRC4	LINC01615	0.522073164	1.99E-34	postive
NLRP3	LINC01615	0.459123308	4.88E-26	postive
PJVK	TMEM202-AS1	0.590788981	7.68E-46	postive
SCAF11	TMEM202-AS1	0.4981177	4.99E-31	postive
CHMP2A	AC138956.1	-0.409589711	1.47E-20	negative
GSDME	AC138956.1	0.4578362	6.95E-26	postive
PJVK	AC138956.1	0.410127107	1.29E-20	postive
SCAF11	AC138956.1	0.673079826	1.06E-63	postive
NOD1	AL139082.1	0.50907262	1.50E-32	postive
SCAF11	AL139082.1	0.442096793	4.72E-24	postive
SCAF11	AL135902.2	0.523376538	1.27E-34	postive
GSDME	AL139393.3	0.56056704	1.71E-40	postive
NLRP3	AL139393.3	0.40122197	1.01E-19	postive
GSDME	ALKBH3-AS1	0.502534444	1.23E-31	postive
NLRC4	ALKBH3-AS1	0.411278012	9.87E-21	postive
PJVK	ALKBH3-AS1	0.446250167	1.58E-24	postive
SCAF11	ALKBH3-AS1	0.65797086	5.35E-60	postive
GSDME	LINC00924	0.464295623	1.16E-26	postive

Pyroptosis-related Genes	lncRNA	cor	pvalue	Regulation
NLRC4	LINC00924	0.537575651	8.90E-37	postive
NLRP3	LINC00924	0.400391907	1.22E-19	postive
CASP8	IQCH-AS1	0.434078446	3.72E-23	postive
PJVK	IQCH-AS1	0.587954131	2.58E-45	postive
SCAF11	IQCH-AS1	0.573765656	9.25E-43	postive
SCAF11	LINC01909	0.419874246	1.27E-21	postive
IL1B	AC023157.2	0.417769451	2.10E-21	postive
NLRP3	AC023157.2	0.445197644	2.09E-24	postive
CHMP2A	AP005131.1	-0.410396401	1.21E-20	negative
GSDME	AP005131.1	0.573908357	8.73E-43	postive
IL1A	AP005131.1	0.402970753	6.80E-20	postive
NLRC4	AP005131.1	0.459017571	5.02E-26	postive
NOD1	AP005131.1	0.412619162	7.19E-21	postive
PJVK	AP005131.1	0.428148925	1.66E-22	postive
SCAF11	AP005131.1	0.694964874	1.79E-69	postive
PJVK	AL358472.2	0.523418232	1.26E-34	postive
SCAF11	FIRRE	0.414670974	4.42E-21	postive
NOD1	LINC00654	0.432886941	5.04E-23	postive
PJVK	LINC00654	0.435080145	2.88E-23	postive
CHMP2A	AP001429.1	-0.423663784	5.02E-22	negative
GSDME	AP001429.1	0.52520162	6.82E-35	postive
IL1A	AP001429.1	0.457588554	7.44E-26	postive
IL1B	AP001429.1	0.455019111	1.50E-25	postive
NLRC4	AP001429.1	0.437617104	1.51E-23	postive
PJVK	AP001429.1	0.405644474	3.67E-20	postive
SCAF11	AP001429.1	0.72594984	1.36E-78	postive
CHMP2A	AL139807.1	-0.400647617	1.15E-19	negative
GSDME	AL139807.1	0.550716338	7.26E-39	postive
IL1A	AL139807.1	0.558839095	3.34E-40	postive
IL1B	AL139807.1	0.595090382	1.20E-46	postive
NLRC4	AL139807.1	0.41517374	3.92E-21	postive
NLRP3	AL139807.1	0.597315145	4.52E-47	postive
SCAF11	AL139807.1	0.592252053	4.09E-46	postive
HMGB1	DNAJC3-DT	0.497644829	5.79E-31	postive
NOD1	DNAJC3-DT	0.454285594	1.84E-25	postive
GSDME	AC020704.1	0.441816388	5.08E-24	postive
SCAF11	AC020704.1	0.497411524	6.22E-31	postive
CHMP2A	AP000692.1	-0.405137663	4.13E-20	negative

Pyroptosis-related Genes	lncRNA	cor	pvalue	Regulation
CHMP6	AP000692.1	-0.410863061	1.09E-20	negative
GSDME	AP000692.1	0.526422354	4.48E-35	postive
IL1A	AP000692.1	0.54545677	5.11E-38	postive
IL1B	AP000692.1	0.624678914	1.50E-52	postive
NLRC4	AP000692.1	0.445942753	1.72E-24	postive
NLRP3	AP000692.1	0.558479222	3.83E-40	postive
SCAF11	AP000692.1	0.61643316	7.65E-51	postive
PJVK	AC104035.1	0.530946149	9.31E-36	postive
CHMP2A	AC007216.3	-0.426422111	2.54E-22	negative
CHMP6	AC007216.3	-0.411092748	1.03E-20	negative
GSDME	AC007216.3	0.525901054	5.36E-35	postive
IL1A	AC007216.3	0.402624511	7.36E-20	postive
IL1B	AC007216.3	0.41734513	2.33E-21	postive
NLRC4	AC007216.3	0.445448124	1.96E-24	postive
SCAF11	AC007216.3	0.704037901	5.08E-72	postive
NOD1	AC145207.8	0.462804346	1.75E-26	postive
TIRAP	AC145207.8	0.447408731	1.16E-24	postive
GSDME	AL133260.2	0.50844835	1.84E-32	postive
NLRC4	AL133260.2	0.432030112	6.25E-23	postive
PJVK	AL133260.2	0.456048364	1.14E-25	postive
SCAF11	AL133260.2	0.687311718	2.14E-67	postive
NLRC4	AC124312.3	0.424627334	3.96E-22	postive
SCAF11	AC124312.3	0.471413029	1.53E-27	postive
CHMP2A	AL512656.2	-0.401616206	9.26E-20	negative
GSDME	AL512656.2	0.485965455	2.10E-29	postive
NLRC4	AL512656.2	0.42017595	1.18E-21	postive
SCAF11	AL512656.2	0.637256783	2.95E-55	postive
GSDME	ZNF433-AS1	0.448956905	7.71E-25	postive
NLRC4	ZNF433-AS1	0.441876894	5.00E-24	postive
SCAF11	ZNF433-AS1	0.573180937	1.17E-42	postive
PLCG1	SNHG20	0.452598887	2.90E-25	postive
CHMP2A	CARNMT1-AS1	-0.423077409	5.80E-22	negative
GSDME	CARNMT1-AS1	0.535803497	1.68E-36	postive
IL1B	CARNMT1-AS1	0.406014418	3.37E-20	postive
NLRC4	CARNMT1-AS1	0.495529153	1.12E-30	postive
SCAF11	CARNMT1-AS1	0.716662187	9.89E-76	postive
PJVK	AC084357.2	0.4055867	3.72E-20	postive
SCAF11	AC084357.2	0.548267724	1.81E-38	postive

Pyroptosis-related Genes	lncRNA	cor	pvalue	Regulation
CHMP2A	AC100821.2	-0.406497325	3.02E-20	negative
GSDME	AC100821.2	0.547568176	2.34E-38	postive
NLRC4	AC100821.2	0.476808385	3.19E-28	postive
PJVK	AC100821.2	0.467239825	5.03E-27	postive
SCAF11	AC100821.2	0.717504508	5.50E-76	postive
GSDME	ITPRIP-AS1	0.563307967	5.91E-41	postive
IL1A	ITPRIP-AS1	0.593561115	2.32E-46	postive
IL1B	ITPRIP-AS1	0.731026482	3.29E-80	postive
NLRC4	ITPRIP-AS1	0.473120305	9.34E-28	postive
NLRP3	ITPRIP-AS1	0.574431037	7.07E-43	postive
SCAF11	ITPRIP-AS1	0.503781154	8.29E-32	postive
GSDME	LMO7-AS1	0.494757857	1.42E-30	postive
NOD1	LMO7-AS1	0.418129563	1.93E-21	postive
SCAF11	LMO7-AS1	0.493936514	1.84E-30	postive
PJVK	LINC02561	0.409651168	1.45E-20	postive
SCAF11	LINC02561	0.43531352	2.72E-23	postive
TNF	U62317.4	0.409409221	1.53E-20	postive
GSDME	AC048341.2	0.569333741	5.48E-42	postive
IL1A	AC048341.2	0.465641233	7.91E-27	postive
IL1B	AC048341.2	0.535135154	2.13E-36	postive
NLRC4	AC048341.2	0.512388517	5.07E-33	postive
NLRP3	AC048341.2	0.418778345	1.65E-21	postive
PJVK	AC048341.2	0.475085461	5.28E-28	postive
SCAF11	AC048341.2	0.671597553	2.50E-63	postive
GSDME	AC087854.1	0.571632099	2.19E-42	postive
NLRC4	AC087854.1	0.512479771	4.92E-33	postive
NOD1	AC087854.1	0.429180999	1.28E-22	postive
PJVK	AC087854.1	0.466968334	5.43E-27	postive
SCAF11	AC087854.1	0.681846184	5.95E-66	postive
CHMP2A	SNHG26	-0.424920889	3.68E-22	negative
GSDME	SNHG26	0.531175443	8.59E-36	postive
IL1A	SNHG26	0.413490907	5.85E-21	postive
IL1B	SNHG26	0.517689087	8.72E-34	postive
NLRC4	SNHG26	0.553656616	2.40E-39	postive
SCAF11	SNHG26	0.587474549	3.16E-45	postive
GSDME	ITGB5-AS1	0.581002547	4.77E-44	postive
IL1A	ITGB5-AS1	0.42978323	1.10E-22	postive
IL1B	ITGB5-AS1	0.486639317	1.71E-29	postive

Pyroptosis-related Genes	lncRNA	cor	pvalue	Regulation
NLRC4	ITGB5-AS1	0.510341616	9.93E-33	postive
SCAF11	ITGB5-AS1	0.62880721	2.00E-53	postive
GSDME	AC133644.1	0.595683118	9.24E-47	postive
IL1A	AC133644.1	0.537752072	8.36E-37	postive
IL1B	AC133644.1	0.631141092	6.32E-54	postive
NLRC4	AC133644.1	0.536891403	1.14E-36	postive
NLRP3	AC133644.1	0.491088851	4.42E-30	postive
SCAF11	AC133644.1	0.59419573	1.76E-46	postive
NLRP1	A1BG-AS1	0.428915717	1.37E-22	postive
SCAF11	AC096536.1	0.491481439	3.92E-30	postive
PJVK	RBM26-AS1	0.493940698	1.84E-30	postive
SCAF11	RBM26-AS1	0.47830466	2.06E-28	postive
PLCG1	TMEM147-AS1	0.440227902	7.67E-24	postive
CHMP2A	AC087501.4	-0.430157447	1.00E-22	negative
GSDME	AC087501.4	0.500618187	2.27E-31	postive
NLRC4	AC087501.4	0.496810244	7.51E-31	postive
SCAF11	AC087501.4	0.605474848	1.19E-48	postive
GSDME	AC004921.1	0.455023449	1.50E-25	postive
NLRC4	AC004921.1	0.458005657	6.64E-26	postive
GSDME	AC015922.3	0.498187056	4.88E-31	postive
GSDME	JARID2-AS1	0.575973265	3.77E-43	postive
IL1A	JARID2-AS1	0.459128909	4.87E-26	postive
IL1B	JARID2-AS1	0.498274729	4.75E-31	postive
NLRC4	JARID2-AS1	0.470086505	2.24E-27	postive
NLRP3	JARID2-AS1	0.417973216	2.00E-21	postive
SCAF11	JARID2-AS1	0.636972956	3.41E-55	postive
CHMP2A	AL807757.2	-0.404614937	4.66E-20	negative
GSDME	AL807757.2	0.559308837	2.78E-40	postive
IL1A	AL807757.2	0.51059092	9.15E-33	postive
IL1B	AL807757.2	0.542085833	1.75E-37	postive
NLRC4	AL807757.2	0.458143865	6.39E-26	postive
NLRP3	AL807757.2	0.50327648	9.74E-32	postive
PJVK	AL807757.2	0.424845999	3.75E-22	postive
SCAF11	AL807757.2	0.680745108	1.15E-65	postive
IRF1	LINC02446	0.660890862	1.07E-60	postive
AIM2	LINC02446	0.569886486	4.40E-42	postive
GZMA	LINC02446	0.686387526	3.77E-67	postive
CHMP2A	AC092755.1	-0.43311811	4.75E-23	negative

Pyroptosis-related Genes	lncRNA	cor	pvalue	Regulation
GSDME	AC092755.1	0.531374079	8.01E-36	postive
IL1A	AC092755.1	0.487311346	1.40E-29	postive
IL1B	AC092755.1	0.516226442	1.42E-33	postive
NLRC4	AC092755.1	0.43483719	3.07E-23	postive
NLRP3	AC092755.1	0.496608906	8.00E-31	postive
SCAF11	AC092755.1	0.6586777	3.63E-60	postive
GSDME	DNM3OS	0.608483689	3.04E-49	postive
NLRC4	DNM3OS	0.520768066	3.09E-34	postive
PJVK	DNM3OS	0.429786346	1.10E-22	postive
SCAF11	DNM3OS	0.613592199	2.89E-50	postive
CHMP2A	AC211433.1	-0.431532646	7.09E-23	negative
CHMP6	AC211433.1	-0.416890616	2.60E-21	negative
GSDME	AC211433.1	0.549067797	1.34E-38	postive
IL1A	AC211433.1	0.4175583	2.21E-21	postive
IL1B	AC211433.1	0.427370099	2.01E-22	postive
NLRC4	AC211433.1	0.469775912	2.44E-27	postive
SCAF11	AC211433.1	0.708673138	2.33E-73	postive
GSDME	AC104083.1	0.619245069	2.03E-51	postive
NLRC4	AC104083.1	0.481254755	8.61E-29	postive
NLRP3	AC104083.1	0.402421719	7.71E-20	postive
SCAF11	AC104083.1	0.493835398	1.90E-30	postive
PJVK	LINC01811	0.426247322	2.65E-22	postive
SCAF11	LINC01811	0.538654039	6.05E-37	postive
NOD1	AC109460.2	0.412152434	8.03E-21	postive
PJVK	AC109460.2	0.440716349	6.76E-24	postive
PLCG1	AC109460.2	0.426535167	2.47E-22	postive
SCAF11	AC109460.2	0.461256055	2.70E-26	postive
CHMP2A	ARRDC3-AS1	-0.409694664	1.43E-20	negative
NLRC4	ARRDC3-AS1	0.416696137	2.72E-21	postive
SCAF11	ARRDC3-AS1	0.621798002	6.00E-52	postive
CHMP2A	AC004594.1	-0.428117674	1.67E-22	negative
GSDME	AC004594.1	0.48781176	1.20E-29	postive
NLRC4	AC004594.1	0.439140269	1.02E-23	postive
PJVK	AC004594.1	0.428564693	1.49E-22	postive
SCAF11	AC004594.1	0.709490948	1.34E-73	postive
PJVK	AC132192.2	0.432573208	5.45E-23	postive
CASP8	AC012360.3	0.405803694	3.54E-20	postive
NOD1	AC012360.3	0.496849111	7.42E-31	postive

Pyroptosis-related Genes	lncRNA	cor	pvalue	Regulation
PJVK	AC012360.3	0.555266913	1.31E-39	postive
SCAF11	AC012360.3	0.494710801	1.45E-30	postive
CHMP2A	AL049840.2	-0.438004223	1.36E-23	negative
GSDME	AL049840.2	0.572335938	1.65E-42	postive
IL1A	AL049840.2	0.44060834	6.95E-24	postive
IL1B	AL049840.2	0.493444502	2.14E-30	postive
NLRC4	AL049840.2	0.500735646	2.18E-31	postive
NLRP3	AL049840.2	0.468435007	3.58E-27	postive
SCAF11	AL049840.2	0.65796622	5.36E-60	postive
GSDME	AC055811.1	0.527365376	3.24E-35	postive
IL1A	AC055811.1	0.400800254	1.12E-19	postive
IL1B	AC055811.1	0.427528809	1.93E-22	postive
NLRC4	AC055811.1	0.434818256	3.08E-23	postive
NLRP3	AC055811.1	0.450200832	5.53E-25	postive
SCAF11	AC055811.1	0.574108961	8.05E-43	postive
PJVK	LINC01952	0.418322586	1.84E-21	postive
SCAF11	AL024498.1	0.467873997	4.20E-27	postive
PJVK	AC010618.2	0.60075755	9.88E-48	postive
SCAF11	AC010618.2	0.512900171	4.29E-33	postive
GSDME	SHANK2-AS2	0.44490935	2.26E-24	postive
SCAF11	SHANK2-AS2	0.546404075	3.60E-38	postive
GSDME	AL138759.1	0.458869781	5.23E-26	postive
NLRC4	AL138759.1	0.424953945	3.65E-22	postive
SCAF11	AL138759.1	0.683890245	1.73E-66	postive
CHMP6	AC004908.2	-0.412933473	6.68E-21	negative
GSDME	AC004908.2	0.43851109	1.20E-23	postive
PJVK	AC004908.2	0.509072024	1.50E-32	postive
SCAF11	AC004908.2	0.649471194	5.23E-58	postive
GSDME	MIS18A-AS1	0.456896516	9.00E-26	postive
NLRC4	MIS18A-AS1	0.4148974	4.19E-21	postive
NOD1	MIS18A-AS1	0.458542487	5.73E-26	postive
PJVK	MIS18A-AS1	0.496059394	9.50E-31	postive
SCAF11	MIS18A-AS1	0.59843224	2.77E-47	postive
GSDME	AC007684.2	0.566743541	1.53E-41	postive
IL1A	AC007684.2	0.47052331	1.97E-27	postive
IL1B	AC007684.2	0.498346036	4.64E-31	postive
NLRC4	AC007684.2	0.497361838	6.32E-31	postive
PJVK	AC007684.2	0.4270662	2.17E-22	postive

Pyroptosis-related Genes	lncRNA	cor	pvalue	Regulation
SCAF11	AC007684.2	0.694924634	1.84E-69	postive
GSDME	AC026367.3	0.469458708	2.68E-27	postive
PJVK	AC026367.3	0.520769206	3.09E-34	postive
SCAF11	AC026367.3	0.567571099	1.10E-41	postive
HMGB1	AL390728.6	0.523381569	1.27E-34	postive
PJVK	AL390728.6	0.463891787	1.29E-26	postive
SCAF11	AL390728.6	0.554723302	1.61E-39	postive
NOD1	AL034550.1	0.501394328	1.77E-31	postive
PLCG1	AL034550.1	0.437581069	1.52E-23	postive
SCAF11	AL034550.1	0.516046288	1.51E-33	postive
SCAF11	AC004837.2	0.522401112	1.78E-34	postive
GSDME	AC005838.3	0.544404845	7.51E-38	postive
IL1A	AC005838.3	0.41080349	1.10E-20	postive
IL1B	AC005838.3	0.418599414	1.72E-21	postive
NLRC4	AC005838.3	0.42122347	9.12E-22	postive
NLRP3	AC005838.3	0.467122791	5.20E-27	postive
NOD1	AC005838.3	0.430817522	8.49E-23	postive
SCAF11	AC005838.3	0.594442681	1.59E-46	postive
PJVK	LINC02177	0.451996918	3.41E-25	postive
SCAF11	LINC02177	0.413795963	5.44E-21	postive
GPX4	AP002387.2	0.410721974	1.12E-20	postive
NOD1	UCKL1-AS1	0.474472113	6.31E-28	postive
PLCG1	UCKL1-AS1	0.553852141	2.23E-39	postive
PJVK	AC004943.3	0.487724309	1.23E-29	postive
SCAF11	AC004943.3	0.616620432	7.01E-51	postive
GSDME	AL591848.4	0.413444998	5.91E-21	postive
PJVK	AL591848.4	0.543812397	9.33E-38	postive
SCAF11	AL591848.4	0.624889928	1.35E-52	postive
GSDME	AL162734.1	0.429289423	1.25E-22	postive
NOD1	AL162734.1	0.413198965	6.27E-21	postive
PJVK	AL162734.1	0.437035811	1.75E-23	postive
SCAF11	AL162734.1	0.602076076	5.49E-48	postive
PJVK	AC132938.3	0.576369208	3.21E-43	postive
NOD1	SMG7-AS1	0.460734624	3.12E-26	postive
SCAF11	SMG7-AS1	0.446403767	1.52E-24	postive
TIRAP	SMG7-AS1	0.448699273	8.26E-25	postive
PJVK	AC010463.3	0.472220251	1.21E-27	postive
SCAF11	AC010463.3	0.474523099	6.22E-28	postive

Pyroptosis-related Genes	lncRNA	cor	pvalue	Regulation
NOD1	LINC00653	0.408253996	2.00E-20	postive
PJVK	LINC00653	0.459271725	4.68E-26	postive
PYCARD	MCF2L-AS1	0.483390867	4.55E-29	postive
CHMP2A	TNFRSF10A-AS1	-0.408629664	1.84E-20	negative
CHMP7	TNFRSF10A-AS1	0.423340002	5.44E-22	postive
CASP8	TNFRSF10A-AS1	0.471328877	1.57E-27	postive
GPX4	TNFRSF10A-AS1	-0.511221603	7.44E-33	negative
SCAF11	TNFRSF10A-AS1	0.619197807	2.07E-51	postive
CHMP2A	AC009318.2	-0.418258178	1.87E-21	negative
CHMP6	AC009318.2	-0.403205352	6.44E-20	negative
GSDME	AC009318.2	0.415947108	3.26E-21	postive
PJVK	AC009318.2	0.49762822	5.82E-31	postive
SCAF11	AC009318.2	0.733515068	5.16E-81	postive
IL1B	SMAD9-IT1	0.410981509	1.06E-20	postive
GSDME	AC079313.2	0.420325703	1.13E-21	postive
GSDME	AP000919.1	0.584614549	1.06E-44	postive
IL1A	AP000919.1	0.598073441	3.24E-47	postive
IL1B	AP000919.1	0.661552142	7.41E-61	postive
NLRC4	AP000919.1	0.448544294	8.61E-25	postive
NLRP3	AP000919.1	0.641460413	3.44E-56	postive
SCAF11	AP000919.1	0.619294102	1.98E-51	postive
PJVK	ZKSCAN2-DT	0.514562247	2.47E-33	postive
SCAF11	ZKSCAN2-DT	0.447596498	1.11E-24	postive
SCAF11	AL049870.3	0.499177109	3.57E-31	postive
GSDME	AC093567.1	0.484138854	3.64E-29	postive
IL1A	AC093567.1	0.421384838	8.77E-22	postive
IL1B	AC093567.1	0.454044942	1.96E-25	postive
NLRP3	AC093567.1	0.474294726	6.64E-28	postive
SCAF11	AC093567.1	0.503220201	9.91E-32	postive
CHMP2A	AC011405.1	-0.411149298	1.02E-20	negative
CHMP6	AC011405.1	-0.400530166	1.19E-19	negative
GSDME	AC011405.1	0.538464888	6.47E-37	postive
NLRC4	AC011405.1	0.466965717	5.44E-27	postive
PJVK	AC011405.1	0.426835963	2.29E-22	postive
SCAF11	AC011405.1	0.695966987	9.49E-70	postive
GSDME	MIR3150BHG	0.438839177	1.10E-23	postive
PJVK	MIR3150BHG	0.463400868	1.48E-26	postive
SCAF11	MIR3150BHG	0.580995475	4.79E-44	postive

Pyroptosis-related Genes	lncRNA	cor	pvalue	Regulation
GSDME	AC130371.2	0.417574762	2.20E-21	postive
GSDME	AP001793.1	0.536794375	1.18E-36	postive
IL1A	AP001793.1	0.409818791	1.39E-20	postive
IL1B	AP001793.1	0.505297705	5.09E-32	postive
NLRC4	AP001793.1	0.522631779	1.64E-34	postive
SCAF11	AP001793.1	0.65554576	2.01E-59	postive
GSDME	MIR100HG	0.721181941	4.14E-77	postive
IL1A	MIR100HG	0.45695056	8.87E-26	postive
IL1B	MIR100HG	0.516519897	1.29E-33	postive
NLRC4	MIR100HG	0.523577109	1.19E-34	postive
NLRP3	MIR100HG	0.607842827	4.07E-49	postive
SCAF11	MIR100HG	0.506632076	3.31E-32	postive
GSDME	COX10-AS1	0.444342796	2.62E-24	postive
GPX4	COX10-AS1	-0.415436949	3.68E-21	negative
NOD1	COX10-AS1	0.451099374	4.34E-25	postive
SCAF11	COX10-AS1	0.59733011	4.49E-47	postive
GSDME	AC027682.4	0.420630635	1.05E-21	postive
NOD1	AC027682.4	0.408091566	2.08E-20	postive
PJVK	AC027682.4	0.498609064	4.27E-31	postive
SCAF11	AC027682.4	0.558733925	3.47E-40	postive
SCAF11	AL158837.1	0.506938759	3.00E-32	postive
GSDMD	IGFBP7-AS1	0.406488091	3.02E-20	postive
NOD1	CAPN10-DT	0.487399729	1.36E-29	postive
PJVK	CAPN10-DT	0.487950338	1.15E-29	postive
PLCG1	CAPN10-DT	0.431366637	7.39E-23	postive
CHMP6	AC010245.2	-0.40013498	1.30E-19	negative
GSDME	AC010245.2	0.418462067	1.78E-21	postive
NOD1	AC010245.2	0.402666132	7.29E-20	postive
PJVK	AC010245.2	0.538933462	5.47E-37	postive
SCAF11	AC010245.2	0.610964079	9.75E-50	postive
GSDME	AC012213.3	0.521922824	2.09E-34	postive
NLRC4	AC012213.3	0.472296895	1.18E-27	postive
PJVK	AC012213.3	0.479425213	1.48E-28	postive
SCAF11	AC012213.3	0.67569746	2.30E-64	postive
GSDME	AC007362.1	0.612133148	5.68E-50	postive
IL1A	AC007362.1	0.504627688	6.32E-32	postive
IL1B	AC007362.1	0.534201824	2.96E-36	postive
NLRC4	AC007362.1	0.502562226	1.22E-31	postive

Pyroptosis-related Genes	lncRNA	cor	pvalue	Regulation
NLRP3	AC007362.1	0.494357285	1.61E-30	postive
SCAF11	AC007362.1	0.650609497	2.86E-58	postive
CHMP2A	AL133230.2	-0.4198332	1.28E-21	negative
GSDME	AL133230.2	0.557995592	4.61E-40	postive
IL1A	AL133230.2	0.435630324	2.51E-23	postive
IL1B	AL133230.2	0.481230169	8.67E-29	postive
NLRC4	AL133230.2	0.48484987	2.94E-29	postive
NLRP3	AL133230.2	0.44325496	3.49E-24	postive
SCAF11	AL133230.2	0.691190728	1.93E-68	postive
CHMP2A	AP000763.3	-0.410458089	1.20E-20	negative
GSDME	AP000763.3	0.635503423	7.15E-55	postive
IL1A	AP000763.3	0.545739026	4.60E-38	postive
IL1B	AP000763.3	0.621412123	7.22E-52	postive
NLRC4	AP000763.3	0.562106485	9.43E-41	postive
NLRP3	AP000763.3	0.545289444	5.43E-38	postive
SCAF11	AP000763.3	0.637288681	2.90E-55	postive
CHMP2A	MKNK1-AS1	-0.401688249	9.11E-20	negative
GSDME	MKNK1-AS1	0.597143863	4.87E-47	postive
IL1A	MKNK1-AS1	0.458097753	6.47E-26	postive
IL1B	MKNK1-AS1	0.524473585	8.76E-35	postive
NLRC4	MKNK1-AS1	0.533530655	3.75E-36	postive
NLRP3	MKNK1-AS1	0.457178473	8.33E-26	postive
PJVK	MKNK1-AS1	0.433262968	4.58E-23	postive
SCAF11	MKNK1-AS1	0.639972377	7.39E-56	postive
IRF1	AC115522.1	0.445472024	1.95E-24	postive
SCAF11	AC018653.3	0.4012484	1.01E-19	postive
PJVK	AC063948.1	0.538187293	7.15E-37	postive
SCAF11	AD001527.1	0.524460651	8.80E-35	postive
GSDME	AL512652.1	0.406296559	3.16E-20	postive
NOD1	AL512652.1	0.5080867	2.07E-32	postive
PJVK	AL512652.1	0.408167252	2.05E-20	postive
SCAF11	AL512652.1	0.570281169	3.76E-42	postive
GSDME	AC008121.3	0.570035036	4.15E-42	postive
IL1A	AC008121.3	0.459237064	4.73E-26	postive
IL1B	AC008121.3	0.504455465	6.68E-32	postive
NLRC4	AC008121.3	0.497644596	5.79E-31	postive
SCAF11	AC008121.3	0.641255266	3.83E-56	postive
CHMP2A	POLH-AS1	-0.423689271	4.99E-22	negative

Pyroptosis-related Genes	lncRNA	cor	pvalue	Regulation
SCAF11	POLH-AS1	0.518801838	6.01E-34	postive
GSDME	AC105036.3	0.429680589	1.13E-22	postive
SCAF11	AC105036.3	0.593110319	2.83E-46	postive
SCAF11	INE1	0.476540189	3.45E-28	postive
PJVK	PCAT1	0.524595264	8.40E-35	postive
SCAF11	PCAT1	0.439244539	9.90E-24	postive
GSDME	AC011352.3	0.61167261	7.03E-50	postive
IL1A	AC011352.3	0.616269139	8.26E-51	postive
IL1B	AC011352.3	0.788799223	1.29E-101	postive
NLRC4	AC011352.3	0.528812858	1.96E-35	postive
NLRP3	AC011352.3	0.596274667	7.13E-47	postive
SCAF11	AC011352.3	0.413646551	5.64E-21	postive
GSDME	AC007431.2	0.532059696	6.30E-36	postive
IL1B	AC007431.2	0.412745972	6.98E-21	postive
NLRC4	AC007431.2	0.428619183	1.47E-22	postive
SCAF11	AC007431.2	0.572453715	1.57E-42	postive
PJVK	LINC00471	0.474670853	5.96E-28	postive
CHMP4B	FAM83C-AS1	0.473023368	9.60E-28	postive
PLCG1	FAM83C-AS1	0.406628743	2.93E-20	postive
CASP8	MIR4453HG	0.405186943	4.08E-20	postive
PJVK	MIR4453HG	0.519487179	4.77E-34	postive
SCAF11	MIR4453HG	0.565207612	2.81E-41	postive
CHMP2A	AL020997.2	-0.407619405	2.32E-20	negative
GSDME	AL020997.2	0.528105457	2.50E-35	postive
IL1A	AL020997.2	0.426584519	2.44E-22	postive
IL1B	AL020997.2	0.429751906	1.11E-22	postive
NLRC4	AL020997.2	0.471994352	1.29E-27	postive
NLRP3	AL020997.2	0.417608027	2.19E-21	postive
SCAF11	AL020997.2	0.706122221	1.28E-72	postive
GSDME	AC019205.1	0.532567995	5.27E-36	postive
NLRC4	AC019205.1	0.501113218	1.94E-31	postive
NLRP1	AC019205.1	0.407350023	2.47E-20	postive
NLRP3	AC019205.1	0.41962954	1.34E-21	postive
PJVK	AC019205.1	0.442300966	4.47E-24	postive
SCAF11	AC019205.1	0.437330717	1.62E-23	postive
IRF1	LINC02195	0.614393708	1.99E-50	postive
AIM2	LINC02195	0.508995041	1.54E-32	postive
GZMA	LINC02195	0.69978093	8.19E-71	postive

Pyroptosis-related Genes	lncRNA	cor	pvalue	Regulation
GSDME	AC131953.2	0.532829467	4.80E-36	postive
NLRC4	AC131953.2	0.476477491	3.52E-28	postive
SCAF11	AC131953.2	0.625392286	1.06E-52	postive
GSDME	AC010536.2	0.456730637	9.42E-26	postive
IL1B	AC010536.2	0.453458682	2.30E-25	postive
NLRP3	AC010536.2	0.403875822	5.52E-20	postive
SCAF11	AC010536.2	0.55076869	7.12E-39	postive
GSDME	AC092718.5	0.47505536	5.33E-28	postive
IL1A	AC092718.5	0.424799302	3.80E-22	postive
IL1B	AC092718.5	0.43593965	2.32E-23	postive
NLRC4	AC092718.5	0.422017784	7.51E-22	postive
PJVK	AC092718.5	0.451488667	3.91E-25	postive
SCAF11	AC092718.5	0.588012585	2.51E-45	postive
CHMP3	NNT-AS1	0.508745748	1.67E-32	postive
HMGB1	NNT-AS1	0.454989575	1.52E-25	postive
PJVK	NNT-AS1	0.459284845	4.66E-26	postive
SCAF11	NNT-AS1	0.471531465	1.48E-27	postive
NOD1	AL590133.1	0.410246739	1.26E-20	postive
PJVK	AL590133.1	0.40685852	2.77E-20	postive
SCAF11	AL590133.1	0.403683681	5.77E-20	postive
GSDME	AC011978.2	0.444003641	2.86E-24	postive
NLRC4	AC011978.2	0.415606449	3.53E-21	postive
PJVK	AC011978.2	0.504460165	6.67E-32	postive
SCAF11	AC011978.2	0.678776389	3.73E-65	postive
SCAF11	LINC01967	0.41081312	1.10E-20	postive
CHMP2A	AL136115.2	-0.406064696	3.33E-20	negative
GSDME	AL136115.2	0.535132098	2.13E-36	postive
IL1A	AL136115.2	0.584333162	1.19E-44	postive
IL1B	AL136115.2	0.632946962	2.58E-54	postive
NLRC4	AL136115.2	0.425252985	3.39E-22	postive
NLRP3	AL136115.2	0.563515486	5.45E-41	postive
SCAF11	AL136115.2	0.672162674	1.80E-63	postive
SCAF11	AC004241.4	0.560979898	1.46E-40	postive
CHMP2A	AC008906.1	-0.444196422	2.72E-24	negative
CASP8	AC008906.1	0.414705892	4.38E-21	postive
PJVK	AC008906.1	0.40761225	2.33E-20	postive
SCAF11	AC008906.1	0.703129446	9.24E-72	postive
SCAF11	PDE9A-AS1	0.410126613	1.29E-20	postive

Pyroptosis-related Genes	lncRNA	cor	pvalue	Regulation
GSDME	MIR99AHG	0.491515046	3.88E-30	postive
PJVK	MIR99AHG	0.450548833	5.04E-25	postive
SCAF11	MIR99AHG	0.533114782	4.34E-36	postive
SCAF11	AC074124.1	0.530269989	1.18E-35	postive
PJVK	AC132872.2	0.560185781	1.99E-40	postive
GSDME	CASC15	0.609316673	2.08E-49	postive
NLRC4	CASC15	0.467531044	4.63E-27	postive
NOD1	CASC15	0.437759369	1.45E-23	postive
PJVK	CASC15	0.440277837	7.57E-24	postive
SCAF11	CASC15	0.606684251	6.90E-49	postive
CHMP6	AC005261.1	-0.466484771	6.23E-27	negative
GSDME	AC005261.1	0.483649299	4.21E-29	postive
NLRC4	AC005261.1	0.433420607	4.40E-23	postive
PJVK	AC005261.1	0.503817522	8.19E-32	postive
SCAF11	AC005261.1	0.744244377	1.36E-84	postive
CHMP6	RUSC1-AS1	-0.408035332	2.11E-20	negative
SCAF11	RUSC1-AS1	0.586850679	4.12E-45	postive
GSDME	AC242426.2	0.482649686	5.68E-29	postive
NOD1	AC242426.2	0.509302214	1.39E-32	postive
SCAF11	AC242426.2	0.5404314	3.19E-37	postive
GSDME	AC107021.1	0.624018471	2.06E-52	postive
IL1A	AC107021.1	0.583508813	1.68E-44	postive
IL1B	AC107021.1	0.657477303	7.00E-60	postive
NLRC4	AC107021.1	0.522598992	1.66E-34	postive
NLRP3	AC107021.1	0.54584114	4.43E-38	postive
SCAF11	AC107021.1	0.658854128	3.29E-60	postive
GSDME	AL138962.1	0.464632024	1.05E-26	postive
NOD1	AL138962.1	0.424940955	3.67E-22	postive
SCAF11	AL138962.1	0.485177367	2.66E-29	postive
CHMP2A	ALG13-AS1	-0.402155239	8.19E-20	negative
GSDME	ALG13-AS1	0.490369251	5.51E-30	postive
NLRC4	ALG13-AS1	0.423302164	5.49E-22	postive
PJVK	ALG13-AS1	0.50755154	2.46E-32	postive
SCAF11	ALG13-AS1	0.68683086	2.87E-67	postive
GSDME	AL442125.2	0.590670249	8.08E-46	postive
IL1A	AL442125.2	0.40657689	2.96E-20	postive
IL1B	AL442125.2	0.473125113	9.32E-28	postive
NLRC4	AL442125.2	0.480931795	9.47E-29	postive

Pyroptosis-related Genes	lncRNA	cor	pvalue	Regulation
SCAF11	AL442125.2	0.626834917	5.26E-53	postive
GSDME	FAM87B	0.40277169	7.11E-20	postive
NOD1	FAM87B	0.483368835	4.58E-29	postive
SCAF11	FAM87B	0.509162689	1.46E-32	postive
CHMP3	AC011451.1	0.407837592	2.21E-20	postive
HMGB1	AC011451.1	0.403830394	5.58E-20	postive
SCAF11	AC087222.1	0.543989758	8.74E-38	postive
NOD1	AC232271.1	0.446818453	1.36E-24	postive
PJVK	AC232271.1	0.466709046	5.85E-27	postive
PYCARD	AC026471.3	0.465639052	7.91E-27	postive
PJVK	LINC00390	0.403541275	5.96E-20	postive
SCAF11	LINC00390	0.513545245	3.46E-33	postive
CHMP3	AC011511.3	-0.416460532	2.88E-21	negative
PJVK	AC007663.3	0.483660937	4.20E-29	postive
GSDME	MRPS30-DT	0.525993839	5.20E-35	postive
NLRC4	MRPS30-DT	0.497235493	6.58E-31	postive
PJVK	MRPS30-DT	0.4558809	1.19E-25	postive
SCAF11	MRPS30-DT	0.653219909	7.06E-59	postive
PJVK	AC098820.2	0.441506877	5.50E-24	postive
PJVK	AC027682.1	0.463124283	1.60E-26	postive
SCAF11	AC027682.1	0.452492049	2.98E-25	postive
GSDME	AC027279.1	0.448593296	8.50E-25	postive
PJVK	AC027279.1	0.541874999	1.89E-37	postive
SCAF11	AC027279.1	0.620602705	1.06E-51	postive
GSDME	AC016722.2	0.477970613	2.27E-28	postive
NLRC4	AC016722.2	0.409768558	1.41E-20	postive
NOD1	AC016722.2	0.458325557	6.08E-26	postive
SCAF11	AC016722.2	0.589512745	1.33E-45	postive
SCAF11	AC004982.1	0.400986371	1.07E-19	postive
PLCG1	AC055717.2	0.445715009	1.82E-24	postive
GSDME	PTPRG-AS1	0.500050251	2.71E-31	postive
NLRC4	PTPRG-AS1	0.424892193	3.71E-22	postive
PJVK	PTPRG-AS1	0.432518033	5.53E-23	postive
SCAF11	PTPRG-AS1	0.643763063	1.05E-56	postive
CHMP2A	AC007285.1	-0.406104369	3.30E-20	negative
GSDME	AC007285.1	0.542527058	1.49E-37	postive
NLRC4	AC007285.1	0.442915273	3.81E-24	postive
NOD1	AC007285.1	0.455994958	1.15E-25	postive

Pyroptosis-related Genes	lncRNA	cor	pvalue	Regulation
SCAF11	AC007285.1	0.636012924	5.53E-55	postive
SCAF11	AC012464.1	0.482851314	5.35E-29	postive
GSDME	AC005332.7	0.545984107	4.21E-38	postive
NLRC4	AC005332.7	0.454456296	1.75E-25	postive
PJVK	AC005332.7	0.421714081	8.09E-22	postive
SCAF11	AC005332.7	0.555254881	1.31E-39	postive
GSDME	EML4-AS1	0.474405779	6.43E-28	postive
NLRC4	EML4-AS1	0.463914128	1.29E-26	postive
NLRP3	EML4-AS1	0.429722012	1.12E-22	postive
SCAF11	EML4-AS1	0.546738144	3.19E-38	postive
CHMP2A	AC020978.3	-0.424260159	4.34E-22	negative
CHMP6	AC020978.3	-0.407141505	2.60E-20	negative
GSDME	AC020978.3	0.496093117	9.40E-31	postive
NLRC4	AC020978.3	0.421323847	8.90E-22	postive
PJVK	AC020978.3	0.41479305	4.29E-21	postive
SCAF11	AC020978.3	0.69203439	1.14E-68	postive
GSDME	AC007620.3	0.603363478	3.09E-48	postive
IL1A	AC007620.3	0.456932055	8.91E-26	postive
IL1B	AC007620.3	0.478612626	1.88E-28	postive
NLRC4	AC007620.3	0.512324643	5.18E-33	postive
SCAF11	AC007620.3	0.676061877	1.85E-64	postive
IL1B	MANCR	0.419153894	1.51E-21	postive
NLRC4	MANCR	0.424741397	3.85E-22	postive
GSDME	AC096642.1	0.471266944	1.59E-27	postive
PJVK	AC096642.1	0.494904636	1.36E-30	postive
SCAF11	AC096642.1	0.567063259	1.35E-41	postive
SCAF11	AC006449.3	0.438875824	1.09E-23	postive
CHMP2A	AC083806.2	-0.415900196	3.29E-21	negative
GSDME	AC083806.2	0.571794936	2.05E-42	postive
IL1A	AC083806.2	0.448742841	8.17E-25	postive
IL1B	AC083806.2	0.474093261	7.04E-28	postive
NLRC4	AC083806.2	0.508131183	2.04E-32	postive
PJVK	AC083806.2	0.401312185	9.92E-20	postive
SCAF11	AC083806.2	0.73480629	1.95E-81	postive
CHMP4B	GASAL1	0.448510983	8.69E-25	postive
GSDME	SMIM25	0.4516264	3.77E-25	postive
NLRC4	SMIM25	0.521720282	2.24E-34	postive
NLRP3	SMIM25	0.430731525	8.67E-23	postive

Pyroptosis-related Genes	lncRNA	cor	pvalue	Regulation
TNF	SMIM25	0.466434427	6.32E-27	postive
NOD1	AC008456.1	0.423667211	5.02E-22	postive
PJVK	AC008456.1	0.468543605	3.47E-27	postive
SCAF11	AC008456.1	0.624296116	1.80E-52	postive
GSDME	AC090589.2	0.51930822	5.06E-34	postive
IL1B	AC090589.2	0.405828273	3.52E-20	postive
NLRC4	AC090589.2	0.474353822	6.53E-28	postive
SCAF11	AC090589.2	0.626393154	6.52E-53	postive
PJVK	AL096701.3	0.464121501	1.21E-26	postive
SCAF11	AL096701.3	0.557976326	4.65E-40	postive
NOD1	HOTAIRM1	0.417573333	2.21E-21	postive
CASP8	AC099850.4	0.465421142	8.41E-27	postive
SCAF11	AC099850.4	0.413571337	5.74E-21	postive
GSDME	AC078852.1	0.449519193	6.64E-25	postive
PJVK	AC078852.1	0.413839931	5.38E-21	postive
SCAF11	AC078852.1	0.559910123	2.21E-40	postive
CHMP2A	AC091185.1	-0.427078793	2.16E-22	negative
GSDME	AC091185.1	0.478303736	2.06E-28	postive
NLRC4	AC091185.1	0.432161144	6.05E-23	postive
NLRP3	AC091185.1	0.40346423	6.07E-20	postive
SCAF11	AC091185.1	0.62330383	2.91E-52	postive
GSDME	AL359076.1	0.516155016	1.46E-33	postive
NLRC4	AL359076.1	0.44905132	7.52E-25	postive
NOD1	AL359076.1	0.448959108	7.71E-25	postive
PJVK	AL359076.1	0.430579305	9.01E-23	postive
SCAF11	AL359076.1	0.68548628	6.55E-67	postive
IL1B	LINC00355	0.450023073	5.80E-25	postive
SCAF11	AC011468.5	0.492749672	2.65E-30	postive
GSDME	ANKRD44-AS1	0.5465259	3.45E-38	postive
IL1A	ANKRD44-AS1	0.427297584	2.05E-22	postive
IL1B	ANKRD44-AS1	0.496947831	7.20E-31	postive
NLRC4	ANKRD44-AS1	0.534267193	2.89E-36	postive
NLRP3	ANKRD44-AS1	0.491873015	3.47E-30	postive
SCAF11	ANKRD44-AS1	0.537070275	1.07E-36	postive
PJVK	AC116535.2	0.612796208	4.18E-50	postive
SCAF11	AC116535.2	0.407464876	2.41E-20	postive
CHMP2A	AC005632.3	-0.440231778	7.67E-24	negative
CHMP6	AC005632.3	-0.408696374	1.81E-20	negative

Pyroptosis-related Genes	lncRNA	cor	pvalue	Regulation
GSDME	AC005632.3	0.619455936	1.83E-51	postive
IL1A	AC005632.3	0.485630391	2.32E-29	postive
IL1B	AC005632.3	0.519093602	5.44E-34	postive
NLRC4	AC005632.3	0.540605996	2.99E-37	postive
NLRP3	AC005632.3	0.455132037	1.46E-25	postive
SCAF11	AC005632.3	0.719414254	1.44E-76	postive
HMGB1	PLUT	0.43050314	9.19E-23	postive
PLCG1	PLUT	0.4043175	4.99E-20	postive
CHMP2A	AL590723.1	-0.428201188	1.63E-22	negative
CHMP6	AL590723.1	-0.402238601	8.03E-20	negative
GSDME	AL590723.1	0.566723971	1.54E-41	postive
IL1A	AL590723.1	0.486452144	1.81E-29	postive
IL1B	AL590723.1	0.522598941	1.66E-34	postive
NLRC4	AL590723.1	0.4708882	1.78E-27	postive
NLRP3	AL590723.1	0.427680545	1.86E-22	postive
SCAF11	AL590723.1	0.725801302	1.51E-78	postive
GSDME	AC007406.3	0.476743781	3.25E-28	postive
IL1B	AC007406.3	0.401940056	8.60E-20	postive
NLRC4	AC007406.3	0.488287298	1.04E-29	postive
NLRP3	AC007406.3	0.475181433	5.13E-28	postive
PLCG1	AL121906.2	0.490339307	5.56E-30	postive
CHMP2A	Z69666.1	-0.41231212	7.73E-21	negative
GSDME	Z69666.1	0.463781573	1.33E-26	postive
NLRC4	Z69666.1	0.403319078	6.27E-20	postive
SCAF11	Z69666.1	0.638949521	1.25E-55	postive
GSDME	AC244093.3	0.484718941	3.06E-29	postive
NLRC4	AC244093.3	0.432823128	5.12E-23	postive
PJVK	AC244093.3	0.470528627	1.97E-27	postive
SCAF11	AC244093.3	0.661803078	6.45E-61	postive
GSDME	AC025031.3	0.518835859	5.94E-34	postive
NLRC4	AC025031.3	0.477413371	2.67E-28	postive
PJVK	AC025031.3	0.467433199	4.76E-27	postive
SCAF11	AC025031.3	0.729743529	8.50E-80	postive
NOD1	MMADHC-DT	0.414696811	4.39E-21	postive
NOD1	AC013652.1	0.455750432	1.23E-25	postive
PJVK	AC013652.1	0.493028634	2.43E-30	postive
SCAF11	AC013652.1	0.482824753	5.39E-29	postive
CHMP6	AC019080.5	-0.403218994	6.42E-20	negative

Pyroptosis-related Genes	lncRNA	cor	pvalue	Regulation
GSDME	AC019080.5	0.457554188	7.51E-26	postive
PJVK	AC019080.5	0.495249108	1.22E-30	postive
SCAF11	AC019080.5	0.674006653	6.18E-64	postive
GSDME	AL031005.1	0.47917299	1.59E-28	postive
NLRC4	AL031005.1	0.422951065	5.98E-22	postive
SCAF11	AL031005.1	0.567378657	1.19E-41	postive
GSDME	LINC00900	0.673929768	6.46E-64	postive
IL1B	LINC00900	0.404742057	4.52E-20	postive
NLRC4	LINC00900	0.717323094	6.24E-76	postive
NLRP3	LINC00900	0.560581326	1.70E-40	postive
CHMP6	AF127577.4	-0.433211305	4.64E-23	negative
HMGB1	AF127577.4	0.461411972	2.59E-26	postive
NLRC4	AF127577.4	0.410835566	1.10E-20	postive
SCAF11	AF127577.4	0.667888582	2.10E-62	postive
PJVK	AC027020.2	0.404134943	5.20E-20	postive
CHMP4C	AC064807.1	0.432247724	5.92E-23	postive
NOD1	LINC01176	0.579365357	9.39E-44	postive
PLCG1	LINC01176	0.405638993	3.68E-20	postive
GSDME	BCL2L1-AS1	0.469624071	2.55E-27	postive
NOD1	BCL2L1-AS1	0.416567018	2.81E-21	postive
SCAF11	BCL2L1-AS1	0.642541838	1.97E-56	postive
CHMP4B	AL139384.1	0.456603353	9.75E-26	postive
CHMP2A	AC110813.1	-0.433039115	4.85E-23	negative
CHMP6	AC110813.1	-0.403893083	5.50E-20	negative
GSDME	AC110813.1	0.537186749	1.02E-36	postive
NLRC4	AC110813.1	0.439859265	8.44E-24	postive
SCAF11	AC110813.1	0.700667742	4.61E-71	postive
CHMP2A	HLCS-IT1	-0.405961986	3.41E-20	negative
GSDME	HLCS-IT1	0.464407143	1.12E-26	postive
SCAF11	HLCS-IT1	0.571060719	2.75E-42	postive
CHMP4A	AP006621.2	0.402659295	7.30E-20	postive
NOD1	AP006621.2	0.434262035	3.55E-23	postive
PLCG1	AC233728.1	0.402619527	7.36E-20	postive
CHMP2A	AC012181.1	-0.447307636	1.20E-24	negative
GSDME	AC012181.1	0.477072801	2.96E-28	postive
IL1A	AC012181.1	0.469130711	2.94E-27	postive
IL1B	AC012181.1	0.484490086	3.28E-29	postive
NLRC4	AC012181.1	0.525763384	5.62E-35	postive

Pyroptosis-related Genes	lncRNA	cor	pvalue	Regulation
NLRP3	AC012181.1	0.492539727	2.83E-30	postive
PJVK	AC012181.1	0.420811645	1.01E-21	postive
SCAF11	AC012181.1	0.695301639	1.45E-69	postive
SCAF11	AC112220.2	0.428491819	1.52E-22	postive
CHMP4A	AL160314.2	0.437525622	1.54E-23	postive
PJVK	AL160314.2	0.568867827	6.60E-42	postive
GSDME	MCCC1-AS1	0.408565526	1.86E-20	postive
NOD1	MCCC1-AS1	0.410620099	1.15E-20	postive
PJVK	MCCC1-AS1	0.539616641	4.28E-37	postive
SCAF11	MCCC1-AS1	0.5657891	2.23E-41	postive
PJVK	AC019131.2	0.430868991	8.38E-23	postive
SCAF11	AC019131.2	0.606834202	6.45E-49	postive
GSDME	AC010132.4	0.512983646	4.17E-33	postive
NLRC4	AC010132.4	0.428567944	1.49E-22	postive
NOD1	AC010132.4	0.44922551	7.18E-25	postive
PJVK	AC010132.4	0.406345282	3.12E-20	postive
SCAF11	AC010132.4	0.602881499	3.83E-48	postive
GSDME	MEIS1-AS2	0.574514201	6.83E-43	postive
IL1A	MEIS1-AS2	0.555708094	1.10E-39	postive
IL1B	MEIS1-AS2	0.670028272	6.18E-63	postive
NLRC4	MEIS1-AS2	0.466371788	6.43E-27	postive
NLRP3	MEIS1-AS2	0.513499172	3.52E-33	postive
SCAF11	MEIS1-AS2	0.52276722	1.57E-34	postive
GSDME	AC016727.1	0.450895414	4.59E-25	postive
SCAF11	AC016727.1	0.635675856	6.56E-55	postive
GSDME	AC138932.6	0.558488838	3.82E-40	postive
IL1A	AC138932.6	0.409622961	1.46E-20	postive
IL1B	AC138932.6	0.446019066	1.68E-24	postive
NLRC4	AC138932.6	0.499236606	3.51E-31	postive
NLRP3	AC138932.6	0.406275753	3.17E-20	postive
NOD1	AC138932.6	0.413125411	6.38E-21	postive
SCAF11	AC138932.6	0.657308941	7.68E-60	postive
CHMP2A	AC091057.1	-0.427161861	2.12E-22	negative
SCAF11	AC091057.1	0.530542023	1.07E-35	postive
PJVK	AC114811.2	0.429498654	1.18E-22	postive
CHMP4A	AL132780.2	0.45188291	3.52E-25	postive
PJVK	AL132780.2	0.443419613	3.34E-24	postive
PJVK	LCMT1-AS1	0.405551328	3.75E-20	postive

Pyroptosis-related Genes	lncRNA	cor	pvalue	Regulation
GSDME	TPM1-AS	0.482033144	6.83E-29	postive
IL1A	TPM1-AS	0.442674247	4.06E-24	postive
IL1B	TPM1-AS	0.492444959	2.91E-30	postive
NLRC4	TPM1-AS	0.426884385	2.27E-22	postive
NLRP3	TPM1-AS	0.445807562	1.78E-24	postive
SCAF11	TPM1-AS	0.583548193	1.65E-44	postive
GSDME	RNF144A-AS1	0.700103625	6.65E-71	postive
IL1B	RNF144A-AS1	0.471917307	1.32E-27	postive
GSDMC	RNF144A-AS1	0.461228834	2.72E-26	postive
NLRC4	RNF144A-AS1	0.624977784	1.30E-52	postive
NLRP3	RNF144A-AS1	0.465644775	7.90E-27	postive
SCAF11	RNF144A-AS1	0.418765045	1.65E-21	postive
SCAF11	AC121761.1	0.411907619	8.51E-21	postive
SCAF11	AC141002.1	0.521147108	2.72E-34	postive
SCAF11	AC104365.2	0.444826907	2.31E-24	postive
PJVK	AC120042.2	0.49691181	7.28E-31	postive
SCAF11	AC120042.2	0.453852652	2.06E-25	postive
CHMP2A	AL080317.2	-0.400563278	1.18E-19	negative
GSDME	AL080317.2	0.532187444	6.02E-36	postive
NLRC4	AL080317.2	0.475624904	4.51E-28	postive
NOD1	AL080317.2	0.421306086	8.94E-22	postive
PJVK	AL080317.2	0.516854322	1.15E-33	postive
SCAF11	AL080317.2	0.714856761	3.45E-75	postive
SCAF11	AC018695.3	0.467063144	5.29E-27	postive
GSDME	AL008718.3	0.499977629	2.78E-31	postive
NLRC4	AL008718.3	0.505612419	4.60E-32	postive
PJVK	AL008718.3	0.445298387	2.04E-24	postive
SCAF11	AL008718.3	0.681320478	8.15E-66	postive
CHMP2A	AC114760.2	-0.402434117	7.68E-20	negative
GSDME	AC114760.2	0.56644904	1.72E-41	postive
IL1A	AC114760.2	0.582638062	2.42E-44	postive
IL1B	AC114760.2	0.655607026	1.94E-59	postive
NLRC4	AC114760.2	0.47354747	8.25E-28	postive
NLRP3	AC114760.2	0.556301078	8.82E-40	postive
SCAF11	AC114760.2	0.678304423	4.93E-65	postive
GSDME	AC022973.5	0.55749632	5.58E-40	postive
IL1A	AC022973.5	0.448623833	8.43E-25	postive
IL1B	AC022973.5	0.549413663	1.18E-38	postive

Pyroptosis-related Genes	lncRNA	cor	pvalue	Regulation
NLRC4	AC022973.5	0.532161336	6.08E-36	postive
SCAF11	AC022973.5	0.641939091	2.69E-56	postive
PJVK	AL021707.4	0.536172246	1.47E-36	postive
GSDME	LINC00582	0.473266111	8.95E-28	postive
NLRC4	LINC00582	0.540868097	2.72E-37	postive
SCAF11	LINC00582	0.472935868	9.85E-28	postive
GSDME	AC004466.3	0.590909267	7.29E-46	postive
IL1A	AC004466.3	0.454212292	1.87E-25	postive
IL1B	AC004466.3	0.503378788	9.42E-32	postive
NLRC4	AC004466.3	0.489359041	7.50E-30	postive
NLRP3	AC004466.3	0.422024656	7.50E-22	postive
SCAF11	AC004466.3	0.605552239	1.15E-48	postive
PJVK	AL356299.3	0.596369374	6.84E-47	postive
PLCG1	AL356299.3	0.400733198	1.13E-19	postive
SCAF11	AL356299.3	0.456533389	9.94E-26	postive
GSDME	AC090510.2	0.443451907	3.31E-24	postive
SCAF11	AC090510.2	0.563722308	5.02E-41	postive
PJVK	AC024361.3	0.473934384	7.38E-28	postive
PJVK	AC245884.8	0.442021213	4.81E-24	postive
CHMP2A	AC069542.1	-0.425440302	3.24E-22	negative
CHMP6	AC069542.1	-0.402003683	8.48E-20	negative
GSDME	AC069542.1	0.585774638	6.48E-45	postive
IL1A	AC069542.1	0.485641243	2.32E-29	postive
IL1B	AC069542.1	0.53871376	5.92E-37	postive
NLRC4	AC069542.1	0.519493548	4.76E-34	postive
NLRP3	AC069542.1	0.418003419	1.99E-21	postive
SCAF11	AC069542.1	0.721250496	3.94E-77	postive
GSDME	AC008649.2	0.411137769	1.02E-20	postive
SCAF11	AC008649.2	0.50593098	4.15E-32	postive
GSDME	AP003555.1	0.407782015	2.24E-20	postive
SCAF11	LINC02100	0.4789513	1.70E-28	postive
ELANE	SERTAD4-AS1	0.426219633	2.67E-22	postive
PJVK	AC008764.2	0.460657642	3.19E-26	postive
SCAF11	AC008764.2	0.410665384	1.14E-20	postive
NOD1	SEC24B-AS1	0.409071986	1.66E-20	postive
PJVK	SEC24B-AS1	0.596753471	5.78E-47	postive
SCAF11	SEC24B-AS1	0.476959486	3.06E-28	postive
PJVK	AC099518.4	0.47765978	2.49E-28	postive

Pyroptosis-related Genes	lncRNA	cor	pvalue	Regulation
GSDME	AC108063.1	0.540933655	2.66E-37	postive
IL1A	AC108063.1	0.437459902	1.57E-23	postive
IL1B	AC108063.1	0.486421602	1.83E-29	postive
NLRC4	AC108063.1	0.471087624	1.68E-27	postive
SCAF11	AC108063.1	0.650513566	3.00E-58	postive
SCAF11	ARHGEF38-IT1	0.566050059	2.01E-41	postive
GSDME	AC020594.1	0.439309911	9.73E-24	postive
SCAF11	AC020594.1	0.44987619	6.03E-25	postive
PJVK	AC007785.3	0.448580897	8.53E-25	postive
SCAF11	AC007785.3	0.429730753	1.11E-22	postive
GSDME	AC104984.3	0.460267846	3.55E-26	postive
NLRC4	AC104984.3	0.428908307	1.37E-22	postive
NOD1	AC104984.3	0.424358642	4.23E-22	postive
SCAF11	AC104984.3	0.667969446	2.00E-62	postive
PJVK	AC009113.1	0.41825149	1.87E-21	postive
GSDME	AC009159.3	0.570420817	3.55E-42	postive
IL1B	AC009159.3	0.446520739	1.47E-24	postive
NLRC4	AC009159.3	0.548480262	1.67E-38	postive
NLRP3	AC009159.3	0.487010076	1.53E-29	postive
SCAF11	AC009159.3	0.412149362	8.04E-21	postive
GSDME	AC006213.5	0.46538229	8.51E-27	postive
PJVK	AC006213.5	0.419691179	1.32E-21	postive
SCAF11	AC006213.5	0.514345973	2.66E-33	postive
CHMP2A	SCARNA9	-0.420822415	1.01E-21	negative
CHMP6	SCARNA9	-0.421239997	9.08E-22	negative
GSDME	SCARNA9	0.523996765	1.03E-34	postive
IL1A	SCARNA9	0.432738924	5.23E-23	postive
IL1B	SCARNA9	0.426548622	2.46E-22	postive
NLRC4	SCARNA9	0.456342983	1.05E-25	postive
NLRP3	SCARNA9	0.408845886	1.75E-20	postive
PJVK	SCARNA9	0.438273488	1.27E-23	postive
SCAF11	SCARNA9	0.710362679	7.44E-74	postive
GSDME	AC018552.3	0.418206361	1.89E-21	postive
NOD1	AC018552.3	0.41304438	6.50E-21	postive
SCAF11	AC018552.3	0.537500551	9.15E-37	postive
CHMP2A	AL390961.2	-0.418288329	1.86E-21	negative
GSDME	AL390961.2	0.415226964	3.87E-21	postive
SCAF11	AL390961.2	0.685821344	5.34E-67	postive

Pyroptosis-related Genes	lncRNA	cor	pvalue	Regulation
NLRC4	AC007569.1	0.431316806	7.49E-23	postive
SCAF11	AC007569.1	0.45883782	5.28E-26	postive
CHMP2A	ALMS1-IT1	-0.428882279	1.38E-22	negative
CHMP6	ALMS1-IT1	-0.400395908	1.22E-19	negative
GSDME	ALMS1-IT1	0.453716313	2.14E-25	postive
SCAF11	ALMS1-IT1	0.682709458	3.53E-66	postive
CHMP2A	ERCC8-AS1	-0.402043958	8.40E-20	negative
GSDME	ERCC8-AS1	0.433361587	4.46E-23	postive
SCAF11	ERCC8-AS1	0.696913626	5.19E-70	postive
CHMP2A	AL365356.1	-0.412002544	8.32E-21	negative
GSDME	AL365356.1	0.539016089	5.31E-37	postive
IL1A	AL365356.1	0.408119855	2.07E-20	postive
IL1B	AL365356.1	0.408453816	1.91E-20	postive
NLRC4	AL365356.1	0.473935364	7.37E-28	postive
PJVK	AL365356.1	0.40498758	4.28E-20	postive
SCAF11	AL365356.1	0.732998143	7.59E-81	postive
NOD1	AC097641.2	0.527281966	3.33E-35	postive
PJVK	AC097641.2	0.499875371	2.87E-31	postive
SCAF11	AC097641.2	0.536447011	1.33E-36	postive
GSDME	AL445223.1	0.4246837	3.91E-22	postive
NOD1	AL445223.1	0.40159048	9.32E-20	postive
SCAF11	AL445223.1	0.610793939	1.05E-49	postive
CHMP2A	TIMM23B-AGAP6	-0.448969387	7.69E-25	negative
CHMP6	TIMM23B-AGAP6	-0.432744502	5.22E-23	negative
GSDME	TIMM23B-AGAP6	0.546526269	3.44E-38	postive
IL1A	TIMM23B-AGAP6	0.414072111	5.10E-21	postive
IL1B	TIMM23B-AGAP6	0.426025733	2.80E-22	postive
NLRC4	TIMM23B-AGAP6	0.463160239	1.59E-26	postive
SCAF11	TIMM23B-AGAP6	0.714597267	4.13E-75	postive
SCAF11	LINC01814	0.538030114	7.57E-37	postive
CHMP2A	AL731563.4	-0.413372641	6.02E-21	negative
GSDME	AL731563.4	0.531411232	7.91E-36	postive
NLRC4	AL731563.4	0.460159951	3.66E-26	postive
NOD1	AL731563.4	0.411596395	9.16E-21	postive
PJVK	AL731563.4	0.46811163	3.93E-27	postive
SCAF11	AL731563.4	0.639134952	1.13E-55	postive
GSDME	GARS1-DT	0.502266808	1.34E-31	postive
NOD1	GARS1-DT	0.567548135	1.11E-41	postive

Pyroptosis-related Genes	lncRNA	cor	pvalue	Regulation
PJVK	GARS1-DT	0.433479183	4.33E-23	postive
SCAF11	GARS1-DT	0.593873534	2.03E-46	postive
PJVK	LINC01424	0.472786709	1.03E-27	postive
CHMP2A	AC020612.3	-0.409414997	1.53E-20	negative
GSDME	AC020612.3	0.512847433	4.36E-33	postive
IL1A	AC020612.3	0.462111968	2.13E-26	postive
IL1B	AC020612.3	0.487000531	1.54E-29	postive
NLRP3	AC020612.3	0.488379815	1.01E-29	postive
SCAF11	AC020612.3	0.626498534	6.19E-53	postive
PJVK	AL161729.4	0.476081939	3.95E-28	postive
PJVK	AC005261.3	0.458884195	5.21E-26	postive
SCAF11	AC005261.3	0.430535892	9.11E-23	postive
GSDME	AC073641.1	0.468434455	3.58E-27	postive
NLRC4	AC073641.1	0.446357952	1.54E-24	postive
PJVK	AC073641.1	0.465538144	8.14E-27	postive
SCAF11	AC073641.1	0.654242579	4.06E-59	postive
PJVK	AL161729.2	0.433011242	4.88E-23	postive
SCAF11	AL161729.2	0.52586383	5.43E-35	postive
PJVK	AC022007.1	0.419192432	1.49E-21	postive
GSDME	AL589743.5	0.449916463	5.97E-25	postive
IL1B	AL589743.5	0.429853627	1.08E-22	postive
NLRC4	AL589743.5	0.417183151	2.42E-21	postive
SCAF11	AL589743.5	0.458512038	5.77E-26	postive
GSDME	AC002128.1	0.512030569	5.71E-33	postive
IL1B	AC002128.1	0.439231334	9.93E-24	postive
NLRC4	AC002128.1	0.472892752	9.97E-28	postive
SCAF11	AC002128.1	0.645028994	5.42E-57	postive
GSDME	AL161891.1	0.413724899	5.53E-21	postive
NOD1	AL161891.1	0.496812231	7.51E-31	postive
PJVK	AL161891.1	0.419473326	1.39E-21	postive
SCAF11	AL161891.1	0.614123813	2.25E-50	postive
CHMP2A	SH3BP5-AS1	-0.440959226	6.35E-24	negative
CHMP4A	SH3BP5-AS1	0.424617884	3.97E-22	postive
GSDME	SH3BP5-AS1	0.497930145	5.29E-31	postive
NLRC4	SH3BP5-AS1	0.490595344	5.14E-30	postive
NLRP3	SH3BP5-AS1	0.415949097	3.26E-21	postive
PJVK	SH3BP5-AS1	0.444510219	2.51E-24	postive
SCAF11	SH3BP5-AS1	0.572173246	1.76E-42	postive

Pyroptosis-related Genes	lncRNA	cor	pvalue	Regulation
NOD1	AC007637.1	0.501874309	1.52E-31	postive
SCAF11	AC007637.1	0.541887875	1.88E-37	postive
PJVK	AC107958.3	0.490909983	4.67E-30	postive
CHMP4A	ZFHX2-AS1	0.497717945	5.65E-31	postive
GSDME	ZFHX2-AS1	0.408746192	1.79E-20	postive
PJVK	ZFHX2-AS1	0.52641057	4.50E-35	postive
SCAF11	ZFHX2-AS1	0.530269109	1.18E-35	postive
NLRC4	DTNB-AS1	0.435234234	2.77E-23	postive
CHMP2A	AL355488.1	-0.461481687	2.54E-26	negative
CHMP6	AL355488.1	-0.435071621	2.89E-23	negative
GSDME	AL355488.1	0.436972524	1.78E-23	postive
NLRC4	AL355488.1	0.408529927	1.88E-20	postive
PJVK	AL355488.1	0.534186194	2.98E-36	postive
SCAF11	AL355488.1	0.709788284	1.10E-73	postive
GSDME	AC027808.2	0.509949662	1.13E-32	postive
IL1B	AC027808.2	0.407550691	2.36E-20	postive
NLRC4	AC027808.2	0.44913604	7.35E-25	postive
SCAF11	AC027808.2	0.595152	1.16E-46	postive
SCAF11	AL133215.3	0.401129851	1.03E-19	postive
GSDME	AC104462.2	0.513578665	3.43E-33	postive
IL1A	AC104462.2	0.62613225	7.40E-53	postive
IL1B	AC104462.2	0.7075837	4.83E-73	postive
NLRP3	AC104462.2	0.659792701	1.96E-60	postive
SCAF11	AC104462.2	0.455902989	1.18E-25	postive
GSDME	HIF1A-AS3	0.540985524	2.61E-37	postive
IL1A	HIF1A-AS3	0.531365699	8.04E-36	postive
IL1B	HIF1A-AS3	0.686060038	4.61E-67	postive
NLRC4	HIF1A-AS3	0.485317766	2.55E-29	postive
NLRP3	HIF1A-AS3	0.451872873	3.53E-25	postive
SCAF11	HIF1A-AS3	0.486055435	2.04E-29	postive
GSDME	AC002091.2	0.515094759	2.07E-33	postive
NLRC4	AC002091.2	0.531787624	6.93E-36	postive
NLRP1	AC002091.2	0.430689754	8.76E-23	postive
NLRP3	AC002091.2	0.48639353	1.85E-29	postive
GSDME	AC064801.1	0.524832189	7.75E-35	postive
IL1B	AC064801.1	0.401419749	9.68E-20	postive
NLRC4	AC064801.1	0.449294352	7.05E-25	postive
NLRP3	AC064801.1	0.420981189	9.67E-22	postive

Pyroptosis-related Genes	lncRNA	cor	pvalue	Regulation
SCAF11	AC064801.1	0.595314686	1.08E-46	postive
CHMP2A	AC011933.2	-0.419525165	1.38E-21	negative
GSDME	AC011933.2	0.559075447	3.05E-40	postive
IL1A	AC011933.2	0.470617447	1.92E-27	postive
IL1B	AC011933.2	0.492063071	3.28E-30	postive
NLRC4	AC011933.2	0.473757853	7.76E-28	postive
NLRP3	AC011933.2	0.425188151	3.45E-22	postive
SCAF11	AC011933.2	0.707586208	4.82E-73	postive
PJVK	AC009118.3	0.459435461	4.47E-26	postive
SCAF11	AC009118.3	0.553953093	2.15E-39	postive
PJVK	CTBP1-AS	0.452861449	2.70E-25	postive
CASP3	AC092171.2	-0.40733619	2.48E-20	negative
GSDME	LINC01914	0.618663582	2.67E-51	postive
IL1A	LINC01914	0.458278727	6.16E-26	postive
IL1B	LINC01914	0.561900991	1.02E-40	postive
NLRC4	LINC01914	0.488892221	8.65E-30	postive
NLRP3	LINC01914	0.725106329	2.50E-78	postive
PJVK	TMED2-DT	0.4383698	1.24E-23	postive
NLRC4	VIM-AS1	0.425782392	2.98E-22	postive
NOD1	NCBP2-AS1	0.43744955	1.57E-23	postive
PJVK	NCBP2-AS1	0.40931023	1.57E-20	postive
SCAF11	NCBP2-AS1	0.515901466	1.59E-33	postive
PJVK	AC004812.2	0.447163629	1.24E-24	postive
GSDME	AC138393.3	0.561633745	1.13E-40	postive
NLRC4	AC138393.3	0.485677496	2.29E-29	postive
NOD1	AC138393.3	0.49776245	5.58E-31	postive
SCAF11	AC138393.3	0.660253589	1.52E-60	postive
SCAF11	AL031985.3	0.453528698	2.25E-25	postive
CHMP2A	AC007622.2	-0.461137266	2.79E-26	negative
CHMP6	AC007622.2	-0.423923339	4.71E-22	negative
GSDME	AC007622.2	0.442790891	3.94E-24	postive
NLRC4	AC007622.2	0.407227668	2.55E-20	postive
SCAF11	AC007622.2	0.72788445	3.32E-79	postive
CHMP4B	AL133520.1	0.528417705	2.25E-35	postive
PLCG1	AL133520.1	0.561660221	1.12E-40	postive
CHMP4A	AC009950.1	0.433103045	4.77E-23	postive
GSDME	AC009950.1	0.55513219	1.38E-39	postive
NLRC4	AC009950.1	0.590371841	9.19E-46	postive

Pyroptosis-related Genes	lncRNA	cor	pvalue	Regulation
NLRP1	AC009950.1	0.437264862	1.65E-23	postive
NLRP3	AC009950.1	0.41942183	1.41E-21	postive
NOD1	AC009950.1	0.42226328	7.07E-22	postive
PJVK	AC009950.1	0.412917224	6.70E-21	postive
SCAF11	AC009950.1	0.541116515	2.49E-37	postive
CHMP2A	AC018752.1	-0.435678379	2.48E-23	negative
GSDME	AC018752.1	0.501423349	1.76E-31	postive
NLRC4	AC018752.1	0.43037582	9.48E-23	postive
SCAF11	AC018752.1	0.685735637	5.62E-67	postive
GSDME	AC022558.3	0.55057951	7.65E-39	postive
IL1A	AC022558.3	0.411882957	8.56E-21	postive
IL1B	AC022558.3	0.486608643	1.73E-29	postive
NLRC4	AC022558.3	0.480721232	1.01E-28	postive
PJVK	AC022558.3	0.422941263	5.99E-22	postive
SCAF11	AC022558.3	0.66858216	1.41E-62	postive
GSDME	ITFG1-AS1	0.549200014	1.28E-38	postive
IL1B	ITFG1-AS1	0.407053141	2.65E-20	postive
NLRC4	ITFG1-AS1	0.474401355	6.44E-28	postive
PJVK	ITFG1-AS1	0.4636811	1.37E-26	postive
SCAF11	ITFG1-AS1	0.665703761	7.24E-62	postive
CHMP2A	AC105339.2	-0.470436191	2.02E-27	negative
CHMP6	AC105339.2	-0.462692385	1.81E-26	negative
GSDME	AC105339.2	0.486172276	1.97E-29	postive
NLRC4	AC105339.2	0.436219232	2.16E-23	postive
PJVK	AC105339.2	0.476310312	3.69E-28	postive
SCAF11	AC105339.2	0.771328826	1.76E-94	postive
PJVK	AC000061.1	0.438438835	1.22E-23	postive
SCAF11	AC000061.1	0.589415161	1.38E-45	postive
GSDMD	AC084125.4	0.493618763	2.03E-30	postive
GPX4	AC084125.4	0.401848527	8.78E-20	postive
GSDME	AL049838.1	0.59822221	3.03E-47	postive
NLRP3	AL049838.1	0.432059443	6.21E-23	postive
GSDME	AC245884.10	0.439912333	8.33E-24	postive
SCAF11	AC245884.10	0.593297357	2.61E-46	postive
GSDME	AC025857.2	0.451554044	3.84E-25	postive
IL1A	AC025857.2	0.526645525	4.15E-35	postive
IL1B	AC025857.2	0.582210275	2.89E-44	postive
NLRC4	AC025857.2	0.420703299	1.03E-21	postive

Pyroptosis-related Genes	lncRNA	cor	pvalue	Regulation
NLRP3	AC025857.2	0.444934076	2.24E-24	postive
SCAF11	AC025857.2	0.53861707	6.13E-37	postive
GSDME	AC104695.4	0.447125268	1.26E-24	postive
IL1A	AC104695.4	0.478893577	1.73E-28	postive
IL1B	AC104695.4	0.533276349	4.10E-36	postive
NLRP3	AC104695.4	0.444481572	2.53E-24	postive
SCAF11	AC104695.4	0.479852852	1.30E-28	postive
GSDME	LINC01943	0.682812422	3.32E-66	postive
IL1A	LINC01943	0.467547955	4.61E-27	postive
IL1B	LINC01943	0.597470823	4.22E-47	postive
GSDMC	LINC01943	0.45580769	1.21E-25	postive
IL6	LINC01943	0.402680924	7.26E-20	postive
NLRC4	LINC01943	0.679749794	2.09E-65	postive
NLRP1	LINC01943	0.413622594	5.67E-21	postive
NLRP3	LINC01943	0.597059941	5.06E-47	postive
SCAF11	LINC01943	0.466807517	5.69E-27	postive
GSDME	AL035071.2	0.444616756	2.44E-24	postive
NLRC4	AL035071.2	0.421561751	8.40E-22	postive
PJVK	AL035071.2	0.443374158	3.38E-24	postive
SCAF11	AL035071.2	0.706952063	7.36E-73	postive
GSDME	AC104791.1	0.477940842	2.29E-28	postive
NOD1	AC104791.1	0.464665657	1.04E-26	postive
PJVK	AC104791.1	0.404260833	5.05E-20	postive
SCAF11	AC104791.1	0.520754368	3.11E-34	postive
PJVK	AC020907.4	0.441617264	5.35E-24	postive
PLCG1	AC020907.4	0.456754585	9.36E-26	postive
GSDME	AC007014.2	0.536012728	1.56E-36	postive
IL1A	AC007014.2	0.420717928	1.03E-21	postive
IL1B	AC007014.2	0.447393523	1.17E-24	postive
NLRC4	AC007014.2	0.468445862	3.57E-27	postive
SCAF11	AC007014.2	0.700918627	3.92E-71	postive
PJVK	AC127502.2	0.455262932	1.41E-25	postive
SCAF11	AC127502.2	0.4294426	1.20E-22	postive
CHMP4B	AP001505.1	0.445488976	1.94E-24	postive
NLRP1	AC025244.1	0.575818657	4.02E-43	postive
GSDME	AC007336.1	0.527130621	3.51E-35	postive
IL1A	AC007336.1	0.405409738	3.88E-20	postive
IL1B	AC007336.1	0.427345558	2.02E-22	postive

Pyroptosis-related Genes	lncRNA	cor	pvalue	Regulation
NLRC4	AC007336.1	0.464006362	1.25E-26	postive
PJVK	AC007336.1	0.486059703	2.04E-29	postive
SCAF11	AC007336.1	0.685205563	7.77E-67	postive
CHMP6	AC004076.2	-0.404890818	4.37E-20	negative
GSDME	AC004076.2	0.479347178	1.51E-28	postive
NLRC4	AC004076.2	0.428433569	1.54E-22	postive
PJVK	AC004076.2	0.406687254	2.89E-20	postive
SCAF11	AC004076.2	0.636935173	3.47E-55	postive
SCAF11	AC039056.2	0.479701959	1.36E-28	postive
GSDME	AL592148.3	0.6069213	6.20E-49	postive
IL1A	AL592148.3	0.479103867	1.63E-28	postive
IL1B	AL592148.3	0.542747618	1.38E-37	postive
NLRC4	AL592148.3	0.550676061	7.37E-39	postive
NLRP3	AL592148.3	0.414711224	4.38E-21	postive
PJVK	AL592148.3	0.404153779	5.18E-20	postive
SCAF11	AL592148.3	0.679942514	1.86E-65	postive
PLCG1	AL354836.1	0.482277556	6.35E-29	postive
NLRP3	LINC02773	0.412942639	6.66E-21	postive
PJVK	MALINC1	0.494631462	1.48E-30	postive
SCAF11	MALINC1	0.436680255	1.92E-23	postive
GSDME	AC093752.2	0.426171293	2.71E-22	postive
PJVK	AC093752.2	0.442890296	3.83E-24	postive
SCAF11	AC093752.2	0.650522818	2.99E-58	postive
GSDME	LINC02381	0.50349951	9.07E-32	postive
GSDMC	LINC02381	0.434623525	3.24E-23	postive
NLRC4	LINC02381	0.505611958	4.60E-32	postive
NLRP1	LINC02381	0.42043925	1.10E-21	postive
CHMP4A	AL136295.7	0.674314077	5.16E-64	postive
TIRAP	FAM160A1-DT	0.420846455	9.99E-22	postive
PJVK	SUCLG2-AS1	0.493738697	1.95E-30	postive
SCAF11	SUCLG2-AS1	0.52172117	2.24E-34	postive
PJVK	LINC01545	0.540228393	3.43E-37	postive
SCAF11	LINC01545	0.502027748	1.45E-31	postive
GSDME	LINC01235	0.505326969	5.05E-32	postive
NLRC4	LINC01235	0.527311972	3.30E-35	postive
NLRP3	LINC01235	0.459334278	4.60E-26	postive
GSDME	Z97989.1	0.621812261	5.96E-52	postive
NLRC4	Z97989.1	0.51359902	3.40E-33	postive

Pyroptosis-related Genes	lncRNA	cor	pvalue	Regulation
NOD1	Z97989.1	0.462940226	1.69E-26	postive
PJVK	Z97989.1	0.438769354	1.12E-23	postive
SCAF11	Z97989.1	0.602115686	5.40E-48	postive
GSDME	AC004846.1	0.598356615	2.86E-47	postive
IL1B	AC004846.1	0.424493633	4.09E-22	postive
NLRC4	AC004846.1	0.561506036	1.19E-40	postive
NLRP3	AC004846.1	0.450530306	5.06E-25	postive
SCAF11	AC004846.1	0.406461475	3.04E-20	postive
PJVK	AP002449.1	0.493179068	2.32E-30	postive
SCAF11	AP002449.1	0.65795212	5.40E-60	postive
CHMP4B	AL121832.2	0.564123787	4.29E-41	postive
CHMP2A	AC092436.3	-0.409044039	1.67E-20	negative
GSDME	AC092436.3	0.40225706	8.00E-20	postive
SCAF11	AC092436.3	0.572570449	1.50E-42	postive
SCAF11	AC079766.1	0.493204622	2.30E-30	postive
GSDME	EHD4-AS1	0.453243946	2.44E-25	postive
SCAF11	EHD4-AS1	0.559017863	3.11E-40	postive
CHMP2A	AP002336.2	-0.402171305	8.16E-20	negative
GSDME	AP002336.2	0.512556735	4.80E-33	postive
IL1A	AP002336.2	0.45284115	2.72E-25	postive
IL1B	AP002336.2	0.467445028	4.75E-27	postive
NLRC4	AP002336.2	0.449618453	6.46E-25	postive
PJVK	AP002336.2	0.446571907	1.45E-24	postive
SCAF11	AP002336.2	0.687806364	1.58E-67	postive
SCAF11	VIPR1-AS1	0.526738896	4.02E-35	postive
GSDME	AC103746.1	0.487422509	1.35E-29	postive
NLRC4	AC103746.1	0.418005037	1.99E-21	postive
NLRP3	AC103746.1	0.474910478	5.56E-28	postive
ELANE	AL109741.1	0.403468701	6.06E-20	postive
NLRC4	AL109741.1	0.432017966	6.27E-23	postive
NLRP1	AC021188.1	0.426365553	2.58E-22	postive
SCAF11	AC022400.5	0.480977918	9.34E-29	postive
CHMP2A	AC131159.1	-0.41101786	1.05E-20	negative
GSDME	AC131159.1	0.502419781	1.28E-31	postive
NLRC4	AC131159.1	0.450200687	5.53E-25	postive
PJVK	AC131159.1	0.42991462	1.06E-22	postive
SCAF11	AC131159.1	0.678254529	5.08E-65	postive
GSDME	AC078777.1	0.42283696	6.15E-22	postive

Pyroptosis-related Genes	lncRNA	cor	pvalue	Regulation
SCAF11	AC078777.1	0.594772885	1.37E-46	postive
GSDME	MRAP-AS1	0.454833489	1.58E-25	postive
NOD1	MRAP-AS1	0.414218648	4.92E-21	postive
SCAF11	MRAP-AS1	0.591371006	5.98E-46	postive
SCAF11	AC078883.2	0.591821281	4.93E-46	postive
CHMP2A	AC020900.1	-0.408411191	1.93E-20	negative
CHMP6	AC020900.1	-0.404812815	4.45E-20	negative
GSDME	AC020900.1	0.546939555	2.96E-38	postive
IL1A	AC020900.1	0.441173786	6.00E-24	postive
IL1B	AC020900.1	0.465795837	7.57E-27	postive
NLRC4	AC020900.1	0.495082262	1.29E-30	postive
SCAF11	AC020900.1	0.7266451	8.20E-79	postive
PJVK	AL032819.1	0.46863447	3.38E-27	postive
IRF1	NRIR	0.4007318	1.13E-19	postive
GSDME	Z83843.1	0.44197968	4.86E-24	postive
PJVK	Z83843.1	0.460947679	2.94E-26	postive
SCAF11	Z83843.1	0.613943643	2.45E-50	postive
GSDME	AC243967.2	0.453807002	2.09E-25	postive
PJVK	AC243967.2	0.418279475	1.86E-21	postive
SCAF11	AC243967.2	0.52026754	3.66E-34	postive
GSDME	AL157932.1	0.50983701	1.17E-32	postive
NLRC4	AL157932.1	0.409123977	1.64E-20	postive
NOD1	AL157932.1	0.515634082	1.73E-33	postive
SCAF11	AL157932.1	0.632373495	3.43E-54	postive
NOD1	TPT1-AS1	0.499136943	3.62E-31	postive
SCAF11	TPT1-AS1	0.482467638	6.00E-29	postive
PJVK	LINC01954	0.402729258	7.18E-20	postive
CHMP2A	AC130650.2	-0.443248744	3.49E-24	negative
CHMP6	AC130650.2	-0.42492295	3.68E-22	negative
GSDME	AC130650.2	0.51988468	4.17E-34	postive
NLRC4	AC130650.2	0.482216938	6.46E-29	postive
PJVK	AC130650.2	0.448403002	8.94E-25	postive
SCAF11	AC130650.2	0.748124133	6.25E-86	postive
CHMP2A	SMYD3-IT1	-0.438207995	1.29E-23	negative
CHMP6	SMYD3-IT1	-0.410785915	1.11E-20	negative
GSDME	SMYD3-IT1	0.555293478	1.29E-39	postive
IL1A	SMYD3-IT1	0.420099494	1.20E-21	postive
IL1B	SMYD3-IT1	0.409331866	1.56E-20	postive

Pyroptosis-related Genes	lncRNA	cor	pvalue	Regulation
NLRC4	SMYD3-IT1	0.468676079	3.34E-27	postive
SCAF11	SMYD3-IT1	0.705412122	2.05E-72	postive
CHMP2A	GSTCD-AS1	-0.419662501	1.33E-21	negative
CHMP6	GSTCD-AS1	-0.400679748	1.15E-19	negative
GSDME	GSTCD-AS1	0.484804406	2.98E-29	postive
NLRC4	GSTCD-AS1	0.445199623	2.09E-24	postive
SCAF11	GSTCD-AS1	0.714774851	3.65E-75	postive
SCAF11	CFTR-AS1	0.58599682	5.90E-45	postive
GSDME	AL355001.1	0.520830263	3.03E-34	postive
NLRC4	AL355001.1	0.444123444	2.77E-24	postive
NOD1	AL355001.1	0.46344805	1.46E-26	postive
SCAF11	AL355001.1	0.574287862	7.49E-43	postive
CHMP2A	AC009754.1	-0.406763128	2.84E-20	negative
GSDME	AC009754.1	0.579392839	9.29E-44	postive
IL1A	AC009754.1	0.480046844	1.23E-28	postive
IL1B	AC009754.1	0.475981575	4.07E-28	postive
NLRC4	AC009754.1	0.455561651	1.30E-25	postive
NLRP3	AC009754.1	0.436335607	2.09E-23	postive
SCAF11	AC009754.1	0.698916169	1.43E-70	postive
CHMP2A	AC006033.2	-0.425349603	3.31E-22	negative
GSDME	AC006033.2	0.570153306	3.95E-42	postive
IL1B	AC006033.2	0.40932114	1.56E-20	postive
NLRC4	AC006033.2	0.659579306	2.21E-60	postive
NLRP3	AC006033.2	0.512863654	4.34E-33	postive
SCAF11	AC006033.2	0.526047683	5.10E-35	postive
GPX4	CHKB-DT	0.406954659	2.71E-20	postive
NOD1	C2-AS1	0.452713444	2.81E-25	postive
SCAF11	C2-AS1	0.532041595	6.34E-36	postive
GSDME	AC024075.3	0.490783147	4.85E-30	postive
NLRC4	AC024075.3	0.41323125	6.22E-21	postive
PJVK	AC024075.3	0.414275828	4.85E-21	postive
SCAF11	AC024075.3	0.648259015	9.94E-58	postive
GSDME	AC027373.1	0.428727156	1.43E-22	postive
NLRC4	AC027373.1	0.408549702	1.87E-20	postive
SCAF11	AC027373.1	0.583293768	1.84E-44	postive
GSDME	AC008760.2	0.536745127	1.20E-36	postive
IL1B	AC008760.2	0.402492314	7.58E-20	postive
NLRC4	AC008760.2	0.419934605	1.25E-21	postive

Pyroptosis-related Genes	lncRNA	cor	pvalue	Regulation
NLRP3	AC008760.2	0.489501454	7.18E-30	postive
GSDME	AL354696.2	0.502231619	1.36E-31	postive
NLRC4	AL354696.2	0.417375666	2.31E-21	postive
NOD1	AL354696.2	0.491079206	4.43E-30	postive
PJVK	AL354696.2	0.450645699	4.91E-25	postive
SCAF11	AL354696.2	0.58131507	4.19E-44	postive
PJVK	AC012313.8	0.410398651	1.21E-20	postive
CHMP2A	RERE-AS1	-0.447968799	1.00E-24	negative
CHMP6	RERE-AS1	-0.421519556	8.48E-22	negative
GSDME	RERE-AS1	0.496476482	8.34E-31	postive
NLRC4	RERE-AS1	0.437754757	1.45E-23	postive
SCAF11	RERE-AS1	0.674242609	5.38E-64	postive
CHMP2A	AC012085.2	-0.42196461	7.61E-22	negative
GSDME	AC012085.2	0.497949786	5.26E-31	postive
SCAF11	AC012085.2	0.668012593	1.96E-62	postive
CHMP4A	RNF213-AS1	0.400545281	1.18E-19	postive
GSDME	RNF213-AS1	0.414462625	4.64E-21	postive
SCAF11	RNF213-AS1	0.441284222	5.83E-24	postive
GSDME	AC004253.1	0.431796494	6.63E-23	postive
PJVK	AC004253.1	0.470546537	1.96E-27	postive
SCAF11	AC004253.1	0.552078621	4.36E-39	postive
NOD1	HM13-IT1	0.438158158	1.31E-23	postive
PLCG1	HM13-IT1	0.452630577	2.87E-25	postive
PJVK	AC069222.1	0.555375764	1.25E-39	postive
SCAF11	AC069222.1	0.407283199	2.51E-20	postive
GSDME	MIR223HG	0.54046545	3.15E-37	postive
IL1A	MIR223HG	0.524993898	7.33E-35	postive
IL1B	MIR223HG	0.733584264	4.90E-81	postive
NLRC4	MIR223HG	0.692059622	1.12E-68	postive
NLRP3	MIR223HG	0.65091269	2.43E-58	postive
SCAF11	MIR223HG	0.411336662	9.73E-21	postive
TNF	MIR223HG	0.484993324	2.82E-29	postive
PJVK	AC105020.5	0.410764134	1.11E-20	postive
GSDME	AP000695.1	0.65569358	1.85E-59	postive
NLRC4	AP000695.1	0.50236451	1.30E-31	postive
NLRP3	AP000695.1	0.508970588	1.55E-32	postive
PJVK	AC004839.2	0.431075978	7.95E-23	postive
SCAF11	AC004839.2	0.405848551	3.50E-20	postive

Pyroptosis-related Genes	lncRNA	cor	pvalue	Regulation
GSDME	FAM66C	0.59714992	4.86E-47	postive
NLRC4	FAM66C	0.43271034	5.27E-23	postive
NOD1	FAM66C	0.426304376	2.62E-22	postive
PJVK	FAM66C	0.468885267	3.15E-27	postive
SCAF11	FAM66C	0.539946311	3.80E-37	postive
NOD1	ZNF337-AS1	0.484264308	3.50E-29	postive
PJVK	ZNF337-AS1	0.456154052	1.10E-25	postive
SCAF11	ZNF337-AS1	0.415702069	3.45E-21	postive
GSDME	Z97200.1	0.57164315	2.18E-42	postive
IL1A	Z97200.1	0.497378405	6.29E-31	postive
IL1B	Z97200.1	0.634370597	1.26E-54	postive
NLRC4	Z97200.1	0.510238869	1.03E-32	postive
NLRP3	Z97200.1	0.449367141	6.91E-25	postive
SCAF11	Z97200.1	0.479725567	1.35E-28	postive
IL1A	AP005329.1	0.428935247	1.36E-22	postive
IL1B	AP005329.1	0.503024219	1.06E-31	postive
CHMP2A	AC005072.1	-0.422785353	6.23E-22	negative
GSDME	AC005072.1	0.548783648	1.49E-38	postive
IL1A	AC005072.1	0.438669095	1.15E-23	postive
IL1B	AC005072.1	0.464619028	1.05E-26	postive
NLRC4	AC005072.1	0.467514898	4.65E-27	postive
SCAF11	AC005072.1	0.688504855	1.03E-67	postive
GSDME	GHRLOS	0.410350727	1.23E-20	postive
NOD1	GHRLOS	0.506922773	3.02E-32	postive
PJVK	GHRLOS	0.416685705	2.73E-21	postive
SCAF11	GHRLOS	0.564487075	3.72E-41	postive
PJVK	AC009806.1	0.520104538	3.87E-34	postive
SCAF11	TRIM52-AS1	-0.400764403	1.12E-19	negative
CHMP2A	AC021078.1	-0.445533611	1.91E-24	negative
CHMP6	AC021078.1	-0.426760376	2.34E-22	negative
GSDME	AC021078.1	0.554045885	2.08E-39	postive
IL1A	AC021078.1	0.419904536	1.26E-21	postive
IL1B	AC021078.1	0.419758957	1.30E-21	postive
NLRC4	AC021078.1	0.502313726	1.32E-31	postive
PJVK	AC021078.1	0.482004012	6.89E-29	postive
SCAF11	AC021078.1	0.772888944	4.32E-95	postive
PJVK	AL359232.1	0.433368663	4.46E-23	postive
GSDMD	AC109322.1	0.438331958	1.25E-23	postive

Pyroptosis-related Genes	lncRNA	cor	pvalue	Regulation
PJVK	SMC5-AS1	0.458392335	5.97E-26	postive
SCAF11	SMC5-AS1	0.57936452	9.39E-44	postive
CHMP2A	RAP2C-AS1	-0.434178743	3.63E-23	negative
GSDME	RAP2C-AS1	0.525527189	6.10E-35	postive
NLRC4	RAP2C-AS1	0.407683674	2.29E-20	postive
PJVK	RAP2C-AS1	0.477036536	2.99E-28	postive
SCAF11	RAP2C-AS1	0.66796709	2.01E-62	postive
GSDME	AC134312.5	0.455202427	1.43E-25	postive
GSDME	ZNF571-AS1	0.515947241	1.56E-33	postive
NLRC4	ZNF571-AS1	0.404472222	4.81E-20	postive
SCAF11	ZNF571-AS1	0.663592924	2.37E-61	postive
GSDME	LINC01138	0.445660931	1.85E-24	postive
NOD1	LINC01138	0.502859181	1.11E-31	postive
SCAF11	LINC01138	0.441056146	6.19E-24	postive
NOD1	Z94721.1	0.541990508	1.81E-37	postive
SCAF11	Z94721.1	0.466206413	6.74E-27	postive
NOD1	DCUN1D2-AS	0.541508427	2.16E-37	postive
SCAF11	DCUN1D2-AS	0.481055622	9.13E-29	postive
CASP3	AP001372.2	0.409267732	1.58E-20	postive
CHMP2B	AP001372.2	0.439401467	9.51E-24	postive
CHMP3	AP001372.2	0.425310701	3.35E-22	postive
GSDME	AL442125.1	0.615547532	1.16E-50	postive
IL1A	AL442125.1	0.44462946	2.43E-24	postive
IL1B	AL442125.1	0.524482297	8.73E-35	postive
NLRC4	AL442125.1	0.513041439	4.09E-33	postive
NOD1	AL442125.1	0.405215263	4.06E-20	postive
SCAF11	AL442125.1	0.641837738	2.83E-56	postive
GSDME	AC011337.1	0.401606141	9.28E-20	postive
PJVK	AC011337.1	0.598317054	2.91E-47	postive
SCAF11	AC011337.1	0.416083128	3.15E-21	postive
CHMP2A	AC007216.4	-0.409398522	1.53E-20	negative
CHMP6	AC007216.4	-0.405898835	3.46E-20	negative
GSDME	AC007216.4	0.510024828	1.10E-32	postive
NLRC4	AC007216.4	0.468211662	3.82E-27	postive
SCAF11	AC007216.4	0.67981318	2.01E-65	postive
CHMP2A	PITRM1-AS1	-0.403332754	6.25E-20	negative
GSDME	PITRM1-AS1	0.500946574	2.04E-31	postive
NLRC4	PITRM1-AS1	0.47260629	1.08E-27	postive

Pyroptosis-related Genes	lncRNA	cor	pvalue	Regulation
PJVK	PITRM1-AS1	0.471666242	1.42E-27	postive
SCAF11	PITRM1-AS1	0.682192288	4.83E-66	postive
SCAF11	NARF-AS1	0.474334044	6.57E-28	postive
GSDME	AC083906.3	0.520919675	2.94E-34	postive
NLRC4	AC083906.3	0.431720028	6.76E-23	postive
NOD1	AC083906.3	0.406178454	3.25E-20	postive
SCAF11	AC083906.3	0.4902954	5.64E-30	postive
NOD1	STX18-AS1	0.428320827	1.59E-22	postive
PJVK	STX18-AS1	0.572746493	1.40E-42	postive
SCAF11	STX18-AS1	0.579779779	7.91E-44	postive
GSDME	MIR222HG	0.454857302	1.57E-25	postive
NLRC4	MIR222HG	0.412990834	6.59E-21	postive
SCAF11	MIR222HG	0.675903415	2.04E-64	postive
GSDME	ACAP2-IT1	0.548570094	1.62E-38	postive
IL1A	ACAP2-IT1	0.432578587	5.44E-23	postive
IL1B	ACAP2-IT1	0.447044737	1.28E-24	postive
NLRC4	ACAP2-IT1	0.498584036	4.31E-31	postive
PJVK	ACAP2-IT1	0.445740817	1.81E-24	postive
SCAF11	ACAP2-IT1	0.727274907	5.18E-79	postive
GSDME	AC015922.2	0.441874098	5.00E-24	postive
GSDME	CASC2	0.411640729	9.06E-21	postive
PJVK	CASC2	0.56436814	3.90E-41	postive
SCAF11	CASC2	0.591084804	6.76E-46	postive
PJVK	AC016394.3	0.509742782	1.21E-32	postive
SCAF11	AC016394.3	0.540692445	2.90E-37	postive
PLCG1	LINC02604	0.405131124	4.14E-20	postive
NOD1	AC007255.1	0.527633003	2.95E-35	postive
PJVK	AC007255.1	0.409003268	1.68E-20	postive
CHMP2A	AC009996.1	-0.442737185	3.99E-24	negative
CHMP6	AC009996.1	-0.408487488	1.90E-20	negative
GSDME	AC009996.1	0.502608013	1.21E-31	postive
IL1A	AC009996.1	0.431249894	7.61E-23	postive
IL1B	AC009996.1	0.457500155	7.63E-26	postive
NLRC4	AC009996.1	0.423678402	5.00E-22	postive
NLRP3	AC009996.1	0.425020494	3.60E-22	postive
SCAF11	AC009996.1	0.644814117	6.06E-57	postive
GSDME	AC005104.1	0.513896513	3.08E-33	postive
IL1B	AC005104.1	0.448560857	8.57E-25	postive

Pyroptosis-related Genes	lncRNA	cor	pvalue	Regulation
NLRC4	AC005104.1	0.438682606	1.14E-23	postive
NLRP3	AC005104.1	0.414170284	4.98E-21	postive
PJVK	AC005104.1	0.495028391	1.31E-30	postive
SCAF11	AC005104.1	0.649307127	5.71E-58	postive
CHMP2A	CERNA1	-0.403686602	5.77E-20	negative
GSDME	CERNA1	0.593979235	1.94E-46	postive
IL1B	CERNA1	0.47959246	1.41E-28	postive
NLRC4	CERNA1	0.548226039	1.84E-38	postive
NLRP3	CERNA1	0.523543278	1.20E-34	postive
PJVK	CERNA1	0.40719392	2.57E-20	postive
SCAF11	CERNA1	0.62117811	8.07E-52	postive
GSDME	AC090739.1	0.447925195	1.02E-24	postive
NLRC4	AC090739.1	0.419413314	1.41E-21	postive
PJVK	AC090739.1	0.503410924	9.33E-32	postive
SCAF11	AC090739.1	0.692167276	1.05E-68	postive
SCAF11	AC135050.4	0.459081893	4.93E-26	postive
PLCG1	AL031714.1	0.540521119	3.09E-37	postive
CHMP2A	AC016949.1	-0.416061485	3.17E-21	negative
CHMP6	AC016949.1	-0.406311827	3.15E-20	negative
GSDME	AC016949.1	0.558178487	4.30E-40	postive
IL1A	AC016949.1	0.406215457	3.22E-20	postive
IL1B	AC016949.1	0.405737481	3.60E-20	postive
NLRC4	AC016949.1	0.47680106	3.20E-28	postive
PJVK	AC016949.1	0.409367159	1.55E-20	postive
SCAF11	AC016949.1	0.720444099	6.97E-77	postive
NOD1	RNF139-AS1	0.434946978	2.98E-23	postive
PJVK	RNF139-AS1	0.604274146	2.05E-48	postive
SCAF11	RNF139-AS1	0.572454307	1.57E-42	postive
CHMP2A	GTF3C2-AS1	-0.431051732	8.00E-23	negative
CHMP6	GTF3C2-AS1	-0.427379335	2.00E-22	negative
GSDME	GTF3C2-AS1	0.568758271	6.90E-42	postive
IL1A	GTF3C2-AS1	0.449437784	6.78E-25	postive
IL1B	GTF3C2-AS1	0.485698956	2.28E-29	postive
NLRC4	GTF3C2-AS1	0.480623311	1.04E-28	postive
NLRP3	GTF3C2-AS1	0.426484722	2.50E-22	postive
PJVK	GTF3C2-AS1	0.402009248	8.47E-20	postive
SCAF11	GTF3C2-AS1	0.709669231	1.19E-73	postive
CHMP2B	FGD5-AS1	0.501936233	1.49E-31	postive

Pyroptosis-related Genes	lncRNA	cor	pvalue	Regulation
CHMP3	FGD5-AS1	0.510376421	9.81E-33	postive
CASP8	FGD5-AS1	0.447085568	1.27E-24	postive
PJVK	AC018690.1	0.40068647	1.14E-19	postive
SCAF11	AC018690.1	0.630490998	8.73E-54	postive
GSDME	WASHC5-AS1	0.442947714	3.78E-24	postive
NOD1	WASHC5-AS1	0.402359377	7.82E-20	postive
PJVK	WASHC5-AS1	0.477048257	2.98E-28	postive
SCAF11	WASHC5-AS1	0.562483233	8.15E-41	postive
CHMP2A	AC098869.2	-0.415163184	3.93E-21	negative
PJVK	AC098869.2	0.404001654	5.36E-20	postive
SCAF11	AC098869.2	0.668740591	1.29E-62	postive
GSDME	LINC00702	0.466836279	5.64E-27	postive
SCAF11	HEIH	-0.426222202	2.67E-22	negative
PJVK	AC004803.1	0.440153724	7.82E-24	postive
SCAF11	AC004803.1	0.500619705	2.27E-31	postive
NOD1	MED4-AS1	0.42882823	1.40E-22	postive
SCAF11	MED4-AS1	0.474592205	6.09E-28	postive
GSDME	AL391421.1	0.42769725	1.85E-22	postive
SCAF11	AL391421.1	0.521940451	2.08E-34	postive
PJVK	AP006545.2	0.608398662	3.16E-49	postive
SCAF11	AC002563.1	0.543939661	8.91E-38	postive
CHMP2A	AC005726.2	-0.410104255	1.30E-20	negative
CHMP6	AC005726.2	-0.411414199	9.56E-21	negative
GSDME	AC005726.2	0.477647767	2.50E-28	postive
NLRC4	AC005726.2	0.434192565	3.62E-23	postive
PJVK	AC005726.2	0.460004944	3.82E-26	postive
SCAF11	AC005726.2	0.728920287	1.56E-79	postive
PJVK	AC113139.1	0.497152092	6.75E-31	postive
SCAF11	AC113139.1	0.542325434	1.60E-37	postive
SCAF11	AC022784.6	0.42399917	4.62E-22	postive
GSDME	AC079336.1	0.429570997	1.16E-22	postive
SCAF11	AC079336.1	0.574089241	8.12E-43	postive
PJVK	AC093157.2	0.444252771	2.68E-24	postive
SCAF11	AC093157.2	0.59840094	2.80E-47	postive
GSDME	MIR181A2HG	0.492013368	3.33E-30	postive
NLRC4	MIR181A2HG	0.41809506	1.95E-21	postive
PJVK	MIR181A2HG	0.466985907	5.41E-27	postive
SCAF11	MIR181A2HG	0.631502223	5.29E-54	postive

Pyroptosis-related Genes	lncRNA	cor	pvalue	Regulation
PJVK	AL360181.1	0.40990099	1.36E-20	postive
CHMP4B	AL135818.1	-0.407930271	2.16E-20	negative
GSDME	AL135818.1	0.441968721	4.88E-24	postive
NLRC4	AL135818.1	0.544659463	6.84E-38	postive
NLRP1	AL135818.1	0.435973555	2.30E-23	postive
NLRP3	AL135818.1	0.428742612	1.43E-22	postive
CHMP2A	AC092828.1	-0.428389653	1.56E-22	negative
CHMP6	AC092828.1	-0.413795323	5.44E-21	negative
GSDME	AC092828.1	0.46504082	9.37E-27	postive
SCAF11	AC092828.1	0.662349128	4.76E-61	postive
GSDME	AC004988.1	0.661103503	9.51E-61	postive
IL1A	AC004988.1	0.558419849	3.92E-40	postive
IL1B	AC004988.1	0.641943998	2.68E-56	postive
NLRC4	AC004988.1	0.582613408	2.44E-44	postive
NLRP3	AC004988.1	0.580612323	5.61E-44	postive
SCAF11	AC004988.1	0.625382352	1.07E-52	postive
GSDME	AC007938.3	0.40853438	1.88E-20	postive
PJVK	AC007938.3	0.477930065	2.30E-28	postive
SCAF11	AC007938.3	0.640983714	4.40E-56	postive
NLRC4	AL844908.1	0.454003943	1.98E-25	postive
CHMP2A	MALAT1	-0.403393535	6.17E-20	negative
CHMP6	MALAT1	-0.401654467	9.18E-20	negative
GSDME	MALAT1	0.52432914	9.20E-35	postive
IL1A	MALAT1	0.482772245	5.48E-29	postive
IL1B	MALAT1	0.526001461	5.18E-35	postive
NLRC4	MALAT1	0.4248812	3.72E-22	postive
NLRP3	MALAT1	0.476171822	3.85E-28	postive
SCAF11	MALAT1	0.637651402	2.41E-55	postive
GSDME	AC116366.2	0.436989939	1.77E-23	postive
NOD1	AC116366.2	0.419658664	1.33E-21	postive
SCAF11	AC116366.2	0.517719791	8.64E-34	postive
PJVK	ADD3-AS1	0.566830459	1.48E-41	postive
SCAF11	ADD3-AS1	0.413108041	6.41E-21	postive
SCAF11	AL139021.2	0.570189126	3.90E-42	postive
GSDME	AC007278.1	0.437570868	1.52E-23	postive
IL1A	AC007278.1	0.569126628	5.96E-42	postive
IL1B	AC007278.1	0.689133813	6.95E-68	postive
NLRP3	AC007278.1	0.671786422	2.24E-63	postive

Pyroptosis-related Genes	lncRNA	cor	pvalue	Regulation
SCAF11	AC007278.1	0.443305527	3.44E-24	postive
NOD1	AC129510.1	0.426557107	2.46E-22	postive
PJVK	AC129510.1	0.474059212	7.11E-28	postive
SCAF11	AC129510.1	0.43504942	2.91E-23	postive
CHMP2B	AC006333.1	0.428700959	1.44E-22	postive
CHMP3	AC006333.1	0.405780552	3.56E-20	postive
GSDME	AC092338.1	0.515059042	2.10E-33	postive
IL1A	AC092338.1	0.408976651	1.69E-20	postive
IL1B	AC092338.1	0.417271812	2.37E-21	postive
NLRC4	AC092338.1	0.457709268	7.20E-26	postive
SCAF11	AC092338.1	0.69290283	6.60E-69	postive
GSDME	AP001893.1	0.417579823	2.20E-21	postive
NLRC4	AP001893.1	0.431376958	7.37E-23	postive
PJVK	AP001893.1	0.534102146	3.07E-36	postive
SCAF11	AP001893.1	0.692567801	8.15E-69	postive
GSDME	SEPTIN7-DT	0.434237527	3.57E-23	postive
NOD1	SEPTIN7-DT	0.483567608	4.32E-29	postive
PJVK	SEPTIN7-DT	0.480029265	1.24E-28	postive
CHMP2A	AC087683.2	-0.415184067	3.91E-21	negative
GSDME	AC087683.2	0.547224985	2.66E-38	postive
IL1A	AC087683.2	0.437178018	1.69E-23	postive
IL1B	AC087683.2	0.478540931	1.92E-28	postive
NLRC4	AC087683.2	0.493983068	1.81E-30	postive
SCAF11	AC087683.2	0.656512968	1.19E-59	postive
SCAF11	AC093732.1	0.423503429	5.22E-22	postive
PJVK	SEPSECS-AS1	0.52446549	8.78E-35	postive
SCAF11	SEPSECS-AS1	0.543257963	1.14E-37	postive
GSDME	AC011503.2	0.404202539	5.12E-20	postive
IL1B	AC011503.2	0.444831685	2.30E-24	postive
NLRC4	AC011503.2	0.427335846	2.03E-22	postive
CHMP6	AP002490.1	-0.426421492	2.54E-22	negative
GSDME	AP002490.1	0.518333633	7.03E-34	postive
NLRC4	AP002490.1	0.438144048	1.32E-23	postive
NOD1	AP002490.1	0.416372604	2.94E-21	postive
SCAF11	AP002490.1	0.644874711	5.87E-57	postive
NOD1	LINC02256	0.406885606	2.76E-20	postive
CHMP2A	AL157394.1	-0.43168308	6.83E-23	negative
GSDME	AL157394.1	0.550129567	9.04E-39	postive

Pyroptosis-related Genes	lncRNA	cor	pvalue	Regulation
IL1A	AL157394.1	0.481034576	9.19E-29	postive
IL1B	AL157394.1	0.497307335	6.43E-31	postive
NLRC4	AL157394.1	0.527278465	3.34E-35	postive
NLRP3	AL157394.1	0.431031751	8.04E-23	postive
SCAF11	AL157394.1	0.655915604	1.64E-59	postive
GSDME	AC009054.1	0.450439804	5.19E-25	postive
SCAF11	AC009054.1	0.520789572	3.07E-34	postive
PLCG1	AL359881.2	0.521540586	2.38E-34	postive
GSDME	AC007639.1	0.479769736	1.34E-28	postive
NOD1	AC007639.1	0.405684436	3.64E-20	postive
PJVK	AC007639.1	0.403186485	6.47E-20	postive
SCAF11	AC007639.1	0.547488311	2.41E-38	postive
CHMP4B	AL096828.3	0.42018732	1.17E-21	postive
CHMP2A	AC048341.1	-0.428122744	1.67E-22	negative
CHMP6	AC048341.1	-0.410425572	1.21E-20	negative
GSDME	AC048341.1	0.547631623	2.29E-38	postive
NLRC4	AC048341.1	0.487715259	1.24E-29	postive
PJVK	AC048341.1	0.494992587	1.32E-30	postive
SCAF11	AC048341.1	0.688659935	9.32E-68	postive
NLRC4	AC243960.1	0.463152422	1.59E-26	postive
NLRP1	AC243960.1	0.538444555	6.52E-37	postive
PJVK	AC145285.2	0.474057437	7.12E-28	postive
PJVK	AC011466.3	0.427715856	1.84E-22	postive
CHMP2A	AC022211.2	-0.448331298	9.11E-25	negative
CHMP6	AC022211.2	-0.403881174	5.51E-20	negative
GSDME	AC022211.2	0.404190472	5.14E-20	postive
SCAF11	AC022211.2	0.665433554	8.44E-62	postive
CHMP2A	AC025034.1	-0.422801624	6.20E-22	negative
CHMP6	AC025034.1	-0.412114322	8.10E-21	negative
GSDME	AC025034.1	0.565404001	2.60E-41	postive
IL1A	AC025034.1	0.480460735	1.09E-28	postive
IL1B	AC025034.1	0.513547924	3.46E-33	postive
NLRC4	AC025034.1	0.492014196	3.33E-30	postive
NLRP3	AC025034.1	0.426543791	2.47E-22	postive
PJVK	AC025034.1	0.413705918	5.56E-21	postive
SCAF11	AC025034.1	0.733570016	4.95E-81	postive
GSDME	AL121929.3	0.525293564	6.61E-35	postive
IL1A	AL121929.3	0.414027131	5.15E-21	postive

Pyroptosis-related Genes	lncRNA	cor	pvalue	Regulation
IL1B	AL121929.3	0.484915326	2.88E-29	postive
NLRC4	AL121929.3	0.441033397	6.22E-24	postive
NLRP3	AL121929.3	0.461389019	2.60E-26	postive
SCAF11	AL121929.3	0.563127287	6.34E-41	postive
GSDME	AL121987.2	0.413989633	5.20E-21	postive
NOD1	AL121987.2	0.423268166	5.53E-22	postive
PJVK	AL121987.2	0.483248438	4.75E-29	postive
SCAF11	AL121987.2	0.559502603	2.58E-40	postive
GSDME	ANKRD10-IT1	0.496171121	9.17E-31	postive
NLRC4	ANKRD10-IT1	0.423273885	5.52E-22	postive
NOD1	ANKRD10-IT1	0.513093095	4.02E-33	postive
PJVK	ANKRD10-IT1	0.404241961	5.08E-20	postive
SCAF11	ANKRD10-IT1	0.647121871	1.81E-57	postive
CHMP2A	AC112512.1	-0.432282673	5.87E-23	negative
GSDME	AC112512.1	0.509564208	1.28E-32	postive
NLRC4	AC112512.1	0.421858865	7.81E-22	postive
SCAF11	AC112512.1	0.688097311	1.32E-67	postive
CASP8	AC092910.3	0.411975043	8.37E-21	postive
NOD1	AC092910.3	0.571305295	2.49E-42	postive
PJVK	AC092910.3	0.521017882	2.84E-34	postive
SCAF11	AC092910.3	0.518656631	6.31E-34	postive
CHMP4A	AL359921.1	0.418222979	1.89E-21	postive
GSDME	AL359921.1	0.444522027	2.50E-24	postive
NLRP3	AL359921.1	0.455052648	1.49E-25	postive
SCAF11	AL359921.1	0.554687136	1.63E-39	postive
GSDME	AC079793.1	0.589650894	1.25E-45	postive
IL1A	AC079793.1	0.574335569	7.34E-43	postive
IL1B	AC079793.1	0.63947999	9.51E-56	postive
NLRC4	AC079793.1	0.49780962	5.49E-31	postive
NLRP3	AC079793.1	0.54639614	3.61E-38	postive
SCAF11	AC079793.1	0.685011763	8.75E-67	postive
GSDME	AL137186.2	0.417589583	2.20E-21	postive
CHMP2A	AL021328.1	-0.404206732	5.12E-20	negative
GSDME	AL021328.1	0.554284659	1.90E-39	postive
NLRC4	AL021328.1	0.447427067	1.16E-24	postive
SCAF11	AL021328.1	0.661555949	7.39E-61	postive
GSDME	AC002550.2	0.512494473	4.90E-33	postive
IL1A	AC002550.2	0.450211481	5.51E-25	postive

Pyroptosis-related Genes	lncRNA	cor	pvalue	Regulation
IL1B	AC002550.2	0.533956487	3.23E-36	postive
NLRC4	AC002550.2	0.453724084	2.14E-25	postive
SCAF11	AC002550.2	0.569054377	6.13E-42	postive
SCAF11	CASK-AS1	0.477281947	2.78E-28	postive
SCAF11	FARSA-AS1	0.542307648	1.61E-37	postive
CHMP4A	AC002398.1	0.428878974	1.38E-22	postive
GSDME	AL021578.1	0.413602391	5.70E-21	postive
SCAF11	AL021578.1	0.649011185	6.67E-58	postive
PYCARD	AC137630.3	0.412085062	8.16E-21	postive
SCAF11	LINC00449	0.504322902	6.97E-32	postive
CHMP2A	CRTC3-AS1	-0.417028532	2.51E-21	negative
GSDME	CRTC3-AS1	0.465197248	8.96E-27	postive
NLRC4	CRTC3-AS1	0.433511563	4.30E-23	postive
PJVK	CRTC3-AS1	0.443572305	3.21E-24	postive
SCAF11	CRTC3-AS1	0.640616712	5.31E-56	postive
CHMP2A	ACSL3-AS1	-0.406563278	2.97E-20	negative
GSDME	ACSL3-AS1	0.497577886	5.91E-31	postive
IL1A	ACSL3-AS1	0.415116991	3.97E-21	postive
IL1B	ACSL3-AS1	0.426769383	2.33E-22	postive
NLRC4	ACSL3-AS1	0.458648081	5.56E-26	postive
PJVK	ACSL3-AS1	0.43104826	8.01E-23	postive
SCAF11	ACSL3-AS1	0.745033691	7.31E-85	postive
PJVK	LINC00893	0.522794976	1.55E-34	postive
SCAF11	LINC00893	0.528632244	2.09E-35	postive
GSDME	AC007431.1	0.48498745	2.82E-29	postive
NLRC4	AC007431.1	0.411646085	9.05E-21	postive
SCAF11	AC007431.1	0.544990317	6.06E-38	postive
PLCG1	AC245140.2	0.431407562	7.32E-23	postive
NOD1	AC013731.1	0.426009627	2.82E-22	postive
PJVK	AC093382.1	0.457822097	6.98E-26	postive
SCAF11	AC093382.1	0.598104826	3.19E-47	postive
SCAF11	NPSR1-AS1	0.486740558	1.66E-29	postive
SCAF11	AC002306.1	0.524603877	8.38E-35	postive
CHMP4A	AC068888.1	0.417854339	2.06E-21	postive
PJVK	AC068888.1	0.483777355	4.06E-29	postive
SCAF11	AC068888.1	0.503582249	8.83E-32	postive
GSDME	ALOX12-AS1	0.484409047	3.36E-29	postive
NLRC4	ALOX12-AS1	0.418116141	1.94E-21	postive

Pyroptosis-related Genes	lncRNA	cor	pvalue	Regulation
NLRP3	ALOX12-AS1	0.401765315	8.95E-20	postive
SCAF11	ALOX12-AS1	0.529181203	1.72E-35	postive
GSDME	BNC2-AS1	0.672984478	1.12E-63	postive
IL1A	BNC2-AS1	0.595856015	8.56E-47	postive
IL1B	BNC2-AS1	0.676590507	1.36E-64	postive
NLRC4	BNC2-AS1	0.487561805	1.30E-29	postive
NLRP3	BNC2-AS1	0.630815364	7.43E-54	postive
SCAF11	BNC2-AS1	0.540251553	3.40E-37	postive
PJVK	AC021321.1	0.581268521	4.27E-44	postive
SCAF11	AC021321.1	0.414280618	4.85E-21	postive
CHMP2A	AC021087.3	-0.403241241	6.39E-20	negative
SCAF11	AC021087.3	0.513907783	3.07E-33	postive
CHMP2A	AL122035.1	-0.449761975	6.22E-25	negative
GSDME	AL122035.1	0.605362772	1.26E-48	postive
IL1B	AL122035.1	0.436303027	2.11E-23	postive
NLRC4	AL122035.1	0.562419001	8.35E-41	postive
NLRP3	AL122035.1	0.443524088	3.25E-24	postive
SCAF11	AL122035.1	0.665283542	9.18E-62	postive
GSDME	PSPC1-AS2	0.541742341	1.98E-37	postive
NLRC4	PSPC1-AS2	0.467752667	4.35E-27	postive
NOD1	PSPC1-AS2	0.435240333	2.77E-23	postive
SCAF11	PSPC1-AS2	0.59015197	1.01E-45	postive
GSDME	AC107308.1	0.48074323	1.00E-28	postive
IL1A	AC107308.1	0.429293131	1.24E-22	postive
IL1B	AC107308.1	0.539842115	3.94E-37	postive
NLRC4	AC107308.1	0.449004799	7.61E-25	postive
SCAF11	AC107308.1	0.467838935	4.24E-27	postive
NOD1	AF064858.2	0.405301666	3.98E-20	postive
GSDME	AL031432.3	0.472639268	1.07E-27	postive
SCAF11	AL031432.3	0.437025277	1.75E-23	postive
BAK1	TFAP2A-AS1	0.420640368	1.05E-21	postive
IRF1	TFAP2A-AS1	0.589543092	1.31E-45	postive
SCAF11	AC091153.2	0.472771242	1.03E-27	postive
CHMP4B	AC136475.5	0.404391583	4.90E-20	postive
PJVK	AC010491.1	0.411859677	8.60E-21	postive
GSDME	AL078587.1	0.424968355	3.64E-22	postive
NOD1	AL078587.1	0.400657484	1.15E-19	postive
SCAF11	AL078587.1	0.523825577	1.09E-34	postive

Pyroptosis-related Genes	lncRNA	cor	pvalue	Regulation
PJVK	AC074117.1	0.424258529	4.34E-22	postive
PLCG1	AC074117.1	0.486730692	1.67E-29	postive
GSDME	AC022079.1	0.467000369	5.38E-27	postive
NLRC4	AC022079.1	0.41633046	2.97E-21	postive
PJVK	AC022079.1	0.464798536	1.00E-26	postive
SCAF11	AC022079.1	0.695080671	1.67E-69	postive
CHMP2A	AC022150.4	-0.418714805	1.68E-21	negative
GSDME	AC022150.4	0.476558439	3.44E-28	postive
NLRC4	AC022150.4	0.487163882	1.46E-29	postive
SCAF11	AC022150.4	0.696700058	5.94E-70	postive
CHMP2A	LAMTOR5-AS1	-0.437140222	1.70E-23	negative
GSDME	LAMTOR5-AS1	0.451004138	4.46E-25	postive
CASP8	LAMTOR5-AS1	0.421723426	8.07E-22	postive
NLRC4	LAMTOR5-AS1	0.460630185	3.21E-26	postive
NOD1	LAMTOR5-AS1	0.462251437	2.05E-26	postive
PJVK	LAMTOR5-AS1	0.564702624	3.42E-41	postive
SCAF11	LAMTOR5-AS1	0.687177085	2.32E-67	postive
PJVK	JPX	0.500267703	2.53E-31	postive
SCAF11	JPX	0.540282136	3.36E-37	postive
GSDME	AP001347.1	0.446355936	1.54E-24	postive
NOD1	AP001347.1	0.491193495	4.28E-30	postive
IL1B	DOCK8-AS1	0.428079447	1.68E-22	postive
NLRC4	AC012676.3	0.402595938	7.40E-20	postive
PJVK	AC012676.3	0.432526291	5.52E-23	postive
SCAF11	AC012676.3	0.649521722	5.09E-58	postive
GSDME	LINC02042	0.415640457	3.51E-21	postive
PJVK	LINC02042	0.470779685	1.83E-27	postive
SCAF11	LINC02042	0.565628362	2.38E-41	postive
GSDME	AC110792.3	0.550311726	8.45E-39	postive
NLRC4	AC110792.3	0.475226218	5.07E-28	postive
PJVK	AC110792.3	0.442108668	4.70E-24	postive
SCAF11	AC110792.3	0.646725202	2.23E-57	postive
SCAF11	LINC00626	0.431061123	7.98E-23	postive
SCAF11	AC010530.1	0.505944523	4.14E-32	postive
IL1B	AC091544.4	0.440902543	6.44E-24	postive
NLRC4	AC091544.4	0.409323601	1.56E-20	postive
CHMP2A	AC026470.2	-0.403115655	6.57E-20	negative
GSDME	AC026470.2	0.591458212	5.76E-46	postive

Pyroptosis-related Genes	lncRNA	cor	pvalue	Regulation
IL1A	AC026470.2	0.504397078	6.80E-32	postive
IL1B	AC026470.2	0.552754263	3.38E-39	postive
NLRC4	AC026470.2	0.502151078	1.39E-31	postive
NLRP3	AC026470.2	0.470571978	1.95E-27	postive
SCAF11	AC026470.2	0.671332004	2.92E-63	postive
PJVK	AC019330.1	0.501688498	1.61E-31	postive
SCAF11	AC019330.1	0.491892886	3.45E-30	postive
SCAF11	AC144548.1	0.447008256	1.30E-24	postive
NOD1	AC012178.1	0.410531934	1.18E-20	postive
GSDME	LINC01686	0.405064585	4.20E-20	postive
NLRC4	LINC01686	0.409522402	1.49E-20	postive
PJVK	AC079414.3	0.5970412	5.10E-47	postive
PLCG1	AL139349.1	0.518064013	7.69E-34	postive
SCAF11	AC092127.1	0.51518504	2.01E-33	postive
SCAF11	AC092718.2	0.545892062	4.35E-38	postive
GSDME	AC009812.3	0.435309383	2.72E-23	postive
PJVK	AC009812.3	0.596177788	7.44E-47	postive
SCAF11	AC009812.3	0.681118676	9.20E-66	postive
GSDME	AC007319.1	0.512585529	4.75E-33	postive
NLRC4	AC007319.1	0.464586761	1.06E-26	postive
SCAF11	AC007319.1	0.645252821	4.82E-57	postive
CHMP4A	AC009097.2	0.43297502	4.92E-23	postive
CHMP2A	AL354726.1	-0.441713596	5.21E-24	negative
CHMP6	AL354726.1	-0.407200562	2.56E-20	negative
GSDME	AL354726.1	0.445827569	1.77E-24	postive
SCAF11	AL354726.1	0.653235687	7.00E-59	postive
GSDME	AL359962.2	0.569621543	4.89E-42	postive
IL1A	AL359962.2	0.416406887	2.92E-21	postive
IL1B	AL359962.2	0.522801319	1.55E-34	postive
NLRC4	AL359962.2	0.487877912	1.18E-29	postive
SCAF11	AL359962.2	0.550015234	9.44E-39	postive
NOD1	AC092123.1	0.440074463	7.98E-24	postive
PJVK	AC092123.1	0.559802887	2.30E-40	postive
GSDME	GK-AS1	0.594130523	1.82E-46	postive
IL1A	GK-AS1	0.662270467	4.97E-61	postive
IL1B	GK-AS1	0.796161629	7.92E-105	postive
NLRC4	GK-AS1	0.485043255	2.77E-29	postive
NLRP3	GK-AS1	0.624708479	1.48E-52	postive

Pyroptosis-related Genes	lncRNA	cor	pvalue	Regulation
SCAF11	GK-AS1	0.547164847	2.72E-38	postive
PJVK	AC245884.9	0.428420994	1.55E-22	postive
GSDME	AC011700.1	0.402443567	7.67E-20	postive
PJVK	AC015813.1	0.576109676	3.57E-43	postive
SCAF11	AC015813.1	0.573658741	9.66E-43	postive
CHMP4B	LINC01006	0.430430106	9.36E-23	postive
SCAF11	AC009041.3	0.469023145	3.03E-27	postive
GSDME	AP000593.3	0.513095195	4.02E-33	postive
IL1B	AP000593.3	0.439777265	8.62E-24	postive
NLRC4	AP000593.3	0.464865302	9.84E-27	postive
NLRP3	AP000593.3	0.435209373	2.79E-23	postive
NOD1	AP000593.3	0.471407347	1.53E-27	postive
PJVK	AP000593.3	0.406302942	3.15E-20	postive
SCAF11	AP000593.3	0.675853242	2.10E-64	postive
PJVK	AC010203.2	0.470053771	2.26E-27	postive
GSDME	LINC02257	0.486404917	1.84E-29	postive
NLRC4	LINC02257	0.43928475	9.80E-24	postive
SCAF11	LINC02257	0.406829743	2.79E-20	postive
PJVK	AC009163.7	0.532938065	4.62E-36	postive
CHMP2A	AP005131.7	-0.407274724	2.52E-20	negative
GSDME	AP005131.7	0.541071282	2.53E-37	postive
IL1A	AP005131.7	0.438156246	1.31E-23	postive
IL1B	AP005131.7	0.402565348	7.46E-20	postive
NLRC4	AP005131.7	0.413626946	5.66E-21	postive
NLRP3	AP005131.7	0.442758499	3.97E-24	postive
PJVK	AP005131.7	0.41898017	1.57E-21	postive
SCAF11	AP005131.7	0.673123089	1.03E-63	postive
GSDME	LINC01772	0.511190922	7.52E-33	postive
NLRC4	LINC01772	0.412968388	6.62E-21	postive
PJVK	LINC01772	0.536492101	1.31E-36	postive
SCAF11	LINC01772	0.500340639	2.48E-31	postive
GSDME	AC084782.3	0.499055836	3.71E-31	postive
NLRC4	AC084782.3	0.408228228	2.02E-20	postive
SCAF11	AC084782.3	0.559068629	3.05E-40	postive
CHMP2A	ZNF451-AS1	-0.421169372	9.24E-22	negative
GSDME	ZNF451-AS1	0.605638281	1.11E-48	postive
IL1A	ZNF451-AS1	0.480113986	1.21E-28	postive
IL1B	ZNF451-AS1	0.496218928	9.04E-31	postive

Pyroptosis-related Genes	lncRNA	cor	pvalue	Regulation
NLRC4	ZNF451-AS1	0.512770252	4.47E-33	postive
NLRP3	ZNF451-AS1	0.417521357	2.23E-21	postive
PJVK	ZNF451-AS1	0.420837638	1.00E-21	postive
SCAF11	ZNF451-AS1	0.722416658	1.72E-77	postive
SCAF11	AP003392.4	0.411805572	8.72E-21	postive
GSDME	AC091180.3	0.415317285	3.79E-21	postive
SCAF11	AC091180.3	0.426452066	2.52E-22	postive
IRF1	DLGAP1-AS5	0.584497315	1.11E-44	postive
AIM2	DLGAP1-AS5	0.508329068	1.91E-32	postive
GZMA	DLGAP1-AS5	0.491568951	3.81E-30	postive
GSDME	AL365436.2	0.432372544	5.74E-23	postive
NLRC4	AL365436.2	0.41032857	1.23E-20	postive
NOD1	AL365436.2	0.468554492	3.46E-27	postive
SCAF11	AL365436.2	0.654451995	3.63E-59	postive
GSDME	AC090877.2	0.45424074	1.86E-25	postive
IL1B	AC090877.2	0.518872316	5.86E-34	postive
NLRC4	AC090877.2	0.443438459	3.32E-24	postive
NLRP3	AC245100.7	0.402046613	8.39E-20	postive
PJVK	AC006504.7	0.478967202	1.69E-28	postive
SCAF11	AC006504.7	0.515636767	1.73E-33	postive
GSDME	VAC14-AS1	0.400167902	1.29E-19	postive
PJVK	VAC14-AS1	0.449250867	7.13E-25	postive
SCAF11	VAC14-AS1	0.561391331	1.24E-40	postive
GSDME	AC080013.1	0.594169831	1.78E-46	postive
IL1B	AC080013.1	0.41154532	9.27E-21	postive
NLRC4	AC080013.1	0.501098275	1.95E-31	postive
NLRP3	AC080013.1	0.429311274	1.24E-22	postive
SCAF11	AC080013.1	0.587491496	3.14E-45	postive
CHMP2A	SND1-IT1	-0.400437522	1.21E-19	negative
GSDME	SND1-IT1	0.53149947	7.67E-36	postive
IL1B	SND1-IT1	0.424497301	4.09E-22	postive
NLRC4	SND1-IT1	0.468866009	3.17E-27	postive
SCAF11	SND1-IT1	0.666511751	4.59E-62	postive
NOD1	AL357033.4	0.429206077	1.27E-22	postive
PLCG1	AL357033.4	0.413056707	6.48E-21	postive
GSDME	LINC02614	0.470025236	2.28E-27	postive
NOD1	LINC02614	0.433166932	4.69E-23	postive
PJVK	LINC02614	0.43564354	2.50E-23	postive

Pyroptosis-related Genes	lncRNA	cor	pvalue	Regulation
SCAF11	LINC02614	0.612698453	4.37E-50	postive
CHMP2A	AL450998.3	-0.407865217	2.19E-20	negative
GSDME	AL450998.3	0.520804085	3.06E-34	postive
IL1A	AL450998.3	0.431173291	7.76E-23	postive
IL1B	AL450998.3	0.473459361	8.47E-28	postive
NLRC4	AL450998.3	0.443403692	3.35E-24	postive
NLRP3	AL450998.3	0.506406134	3.56E-32	postive
SCAF11	AL450998.3	0.580410408	6.10E-44	postive
GSDME	AC022306.3	0.41552284	3.61E-21	postive
SCAF11	AC022306.3	0.551993969	4.50E-39	postive
PJVK	AC016737.1	0.585255962	8.07E-45	postive
SCAF11	AC016737.1	0.486667527	1.70E-29	postive
CHMP2B	MRPL20-AS1	-0.406396128	3.09E-20	negative
CHMP2A	AC118344.1	-0.407371662	2.46E-20	negative
GSDME	AC118344.1	0.422311989	6.99E-22	postive
SCAF11	AC118344.1	0.604675999	1.71E-48	postive
PLCG1	ZNF213-AS1	0.416484631	2.86E-21	postive
GSDME	LINC00578	0.768966466	1.45E-93	postive
IL1B	LINC00578	0.436857148	1.83E-23	postive
NLRC4	LINC00578	0.678449963	4.52E-65	postive
NLRP3	LINC00578	0.540602053	3.00E-37	postive
SCAF11	LINC00578	0.563253904	6.03E-41	postive
HMGB1	AC018645.3	0.459324695	4.61E-26	postive
PJVK	AC018645.3	0.453427643	2.32E-25	postive
SCAF11	AC018645.3	0.508234913	1.97E-32	postive
IRF1	LINC02084	0.431892608	6.47E-23	postive
IRF2	LINC02084	0.408246216	2.01E-20	postive
NLRP1	LINC02084	0.527177165	3.45E-35	postive
CHMP2A	AC105339.3	-0.437026553	1.75E-23	negative
CHMP6	AC105339.3	-0.406964062	2.71E-20	negative
GSDME	AC105339.3	0.483380246	4.57E-29	postive
NLRC4	AC105339.3	0.427729528	1.84E-22	postive
PJVK	AC105339.3	0.448926205	7.78E-25	postive
SCAF11	AC105339.3	0.64963785	4.79E-58	postive
NOD1	AL109804.1	0.511788062	6.18E-33	postive
NOD1	AL031717.1	0.427265106	2.06E-22	postive
SCAF11	AL031717.1	0.426018089	2.81E-22	postive
TIRAP	AL031717.1	0.435453829	2.62E-23	postive

Pyroptosis-related Genes	lncRNA	cor	pvalue	Regulation
CHMP2A	AC243773.2	-0.414015069	5.16E-21	negative
GSDME	AC243773.2	0.484285911	3.48E-29	postive
NLRC4	AC243773.2	0.403086845	6.62E-20	postive
PJVK	AC243773.2	0.459832	4.01E-26	postive
SCAF11	AC243773.2	0.701685587	2.38E-71	postive
GSDME	AC009549.1	0.426617431	2.42E-22	postive
CHMP2A	AC099811.1	-0.424324462	4.27E-22	negative
GSDME	AC099811.1	0.553732407	2.34E-39	postive
IL1A	AC099811.1	0.422285377	7.04E-22	postive
IL1B	AC099811.1	0.401121982	1.04E-19	postive
NLRC4	AC099811.1	0.471580906	1.46E-27	postive
PJVK	AC099811.1	0.45191668	3.49E-25	postive
SCAF11	AC099811.1	0.723363081	8.74E-78	postive
PJVK	AC008870.4	0.477629072	2.51E-28	postive
SCAF11	AC008870.4	0.494304128	1.64E-30	postive
CHMP4A	AL033384.2	0.40277758	7.10E-20	postive
SCAF11	AL033384.2	0.419769563	1.30E-21	postive
CHMP2A	AC025917.1	-0.428200443	1.63E-22	negative
GSDME	AC025917.1	0.601446671	7.27E-48	postive
IL1A	AC025917.1	0.504261301	7.10E-32	postive
IL1B	AC025917.1	0.512994016	4.15E-33	postive
NLRC4	AC025917.1	0.491362425	4.06E-30	postive
NLRP3	AC025917.1	0.47356111	8.22E-28	postive
PJVK	AC025917.1	0.418340841	1.83E-21	postive
SCAF11	AC025917.1	0.698246146	2.21E-70	postive
GSDME	AC026401.1	0.573928411	8.66E-43	postive
IL1A	AC026401.1	0.436764757	1.87E-23	postive
IL1B	AC026401.1	0.440042661	8.05E-24	postive
NLRC4	AC026401.1	0.460424698	3.40E-26	postive
NLRP3	AC026401.1	0.426555979	2.46E-22	postive
PJVK	AC026401.1	0.412315773	7.73E-21	postive
SCAF11	AC026401.1	0.6718863	2.12E-63	postive
GSDME	AC004847.1	0.419236075	1.48E-21	postive
IRF1	AC004847.1	0.485284355	2.58E-29	postive
NLRC4	AC004847.1	0.516679481	1.22E-33	postive
NLRP1	AC004847.1	0.503366071	9.46E-32	postive
NLRP3	AC004847.1	0.472228928	1.21E-27	postive
TNF	AC004847.1	0.451580805	3.82E-25	postive

Pyroptosis-related Genes	lncRNA	cor	pvalue	Regulation
GZMA	AC004847.1	0.405671951	3.65E-20	postive
NOD1	U47924.1	0.418275438	1.86E-21	postive
CHMP2A	AL158207.2	-0.407689409	2.29E-20	negative
GSDME	AL158207.2	0.523324062	1.30E-34	postive
IL1A	AL158207.2	0.415381889	3.73E-21	postive
IL1B	AL158207.2	0.416358531	2.95E-21	postive
NLRC4	AL158207.2	0.438238236	1.28E-23	postive
SCAF11	AL158207.2	0.685706497	5.72E-67	postive
CHMP2A	AL596202.1	-0.412303991	7.75E-21	negative
GSDME	AL596202.1	0.43672688	1.89E-23	postive
NLRC4	AL596202.1	0.407354993	2.47E-20	postive
PJVK	AL596202.1	0.488140043	1.09E-29	postive
SCAF11	AL596202.1	0.646673585	2.29E-57	postive
GSDME	AC027288.3	0.632386936	3.41E-54	postive
IL1A	AC027288.3	0.424308408	4.28E-22	postive
IL1B	AC027288.3	0.462422384	1.95E-26	postive
NLRC4	AC027288.3	0.453137753	2.51E-25	postive
NLRP3	AC027288.3	0.560182726	1.99E-40	postive
SCAF11	AC027288.3	0.512572309	4.77E-33	postive
SCAF11	AC083900.1	0.541293607	2.33E-37	postive
NOD1	AL121894.2	0.405406638	3.88E-20	postive
GSDME	AC090825.1	0.695187274	1.56E-69	postive
IL1A	AC090825.1	0.410972563	1.06E-20	postive
IL1B	AC090825.1	0.52398446	1.04E-34	postive
NLRC4	AC090825.1	0.597648994	3.90E-47	postive
NLRP3	AC090825.1	0.519431872	4.86E-34	postive
SCAF11	AC090825.1	0.496267592	8.90E-31	postive
CHMP2A	AC022893.1	-0.4118951	8.53E-21	negative
GSDME	AC022893.1	0.431288558	7.54E-23	postive
PJVK	AC022893.1	0.405270553	4.00E-20	postive
SCAF11	AC022893.1	0.59469069	1.42E-46	postive
CHMP2A	AC078795.1	-0.453484383	2.28E-25	negative
CHMP6	AC078795.1	-0.432125519	6.10E-23	negative
GSDME	AC078795.1	0.551590405	5.24E-39	postive
IL1A	AC078795.1	0.438828613	1.10E-23	postive
IL1B	AC078795.1	0.460761976	3.10E-26	postive
NLRC4	AC078795.1	0.50116774	1.90E-31	postive
NLRP3	AC078795.1	0.406220196	3.22E-20	postive

Pyroptosis-related Genes	lncRNA	cor	pvalue	Regulation
SCAF11	AC078795.1	0.776378088	1.78E-96	postive
CHMP2A	AC004241.3	-0.420657581	1.05E-21	negative
SCAF11	AC004241.3	0.611339252	8.20E-50	postive
CHMP2A	AC093484.4	-0.461829962	2.30E-26	negative
CASP8	AC093484.4	0.412655654	7.13E-21	postive
NLRC4	AC093484.4	0.402412157	7.72E-20	postive
SCAF11	AC093484.4	0.637411923	2.73E-55	postive
SCAF11	AL022322.1	0.526291202	4.69E-35	postive
GSDME	APOA1-AS	0.532241958	5.91E-36	postive
IL1A	APOA1-AS	0.459754742	4.10E-26	postive
IL1B	APOA1-AS	0.521643011	2.30E-34	postive
NLRC4	APOA1-AS	0.463417505	1.48E-26	postive
NLRP3	APOA1-AS	0.452898043	2.67E-25	postive
SCAF11	APOA1-AS	0.652983744	8.01E-59	postive
GSDME	AC011676.3	0.473646944	8.02E-28	postive
SCAF11	AC011676.3	0.550213027	8.77E-39	postive
GSDME	AC010422.4	0.562661115	7.60E-41	postive
NLRC4	AC010422.4	0.479995156	1.25E-28	postive
PJVK	AC010422.4	0.407757895	2.25E-20	postive
SCAF11	AC010422.4	0.699706275	8.60E-71	postive
CHMP4A	AL359962.1	0.405529261	3.77E-20	postive
PJVK	AL359962.1	0.446433939	1.51E-24	postive
SCAF11	STXBP5-AS1	0.474028694	7.18E-28	postive
GSDME	AL078581.2	0.489167886	7.95E-30	postive
IL1A	AL078581.2	0.528452637	2.22E-35	postive
IL1B	AL078581.2	0.597244783	4.66E-47	postive
NLRC4	AL078581.2	0.431625138	6.93E-23	postive
NLRP3	AL078581.2	0.493503526	2.10E-30	postive
SCAF11	AL078581.2	0.526142644	4.94E-35	postive
GSDME	AC011595.1	0.64272461	1.79E-56	postive
IL1A	AC011595.1	0.603247413	3.25E-48	postive
IL1B	AC011595.1	0.693075221	5.93E-69	postive
NLRC4	AC011595.1	0.506395492	3.58E-32	postive
NLRP3	AC011595.1	0.58112089	4.54E-44	postive
SCAF11	AC011595.1	0.571431154	2.37E-42	postive
GSDME	AC108449.1	0.426678783	2.39E-22	postive
PJVK	AC108449.1	0.406033428	3.36E-20	postive
SCAF11	AC108449.1	0.618971931	2.31E-51	postive

Pyroptosis-related Genes	lncRNA	cor	pvalue	Regulation
NOD1	AL512303.1	0.44316388	3.57E-24	postive
SCAF11	AL512303.1	0.458412148	5.93E-26	postive
GSDME	C1orf147	0.436682024	1.91E-23	postive
SCAF11	C1orf147	0.502918919	1.09E-31	postive
CHMP2A	CEP250-AS1	-0.410516301	1.18E-20	negative
GSDME	CEP250-AS1	0.571853982	2.00E-42	postive
NLRC4	CEP250-AS1	0.557762721	5.04E-40	postive
NOD1	CEP250-AS1	0.416349689	2.96E-21	postive
PJVK	CEP250-AS1	0.454790437	1.60E-25	postive
SCAF11	CEP250-AS1	0.678966785	3.33E-65	postive
GSDME	TRG-AS1	0.435031241	2.92E-23	postive
IRF1	TRG-AS1	0.496346253	8.69E-31	postive
AIM2	TRG-AS1	0.420429026	1.11E-21	postive
NLRC4	TRG-AS1	0.625098948	1.22E-52	postive
NLRP1	TRG-AS1	0.496561975	8.12E-31	postive
NLRP3	TRG-AS1	0.414456459	4.65E-21	postive
SCAF11	TRG-AS1	0.427268656	2.06E-22	postive
GZMA	TRG-AS1	0.552600719	3.58E-39	postive
CHMP2A	AC099811.5	-0.421011336	9.60E-22	negative
GSDME	AC099811.5	0.574919325	5.80E-43	postive
IL1A	AC099811.5	0.452389683	3.07E-25	postive
IL1B	AC099811.5	0.454365101	1.80E-25	postive
NLRC4	AC099811.5	0.481041845	9.17E-29	postive
NLRP3	AC099811.5	0.454599594	1.69E-25	postive
PJVK	AC099811.5	0.423491065	5.24E-22	postive
SCAF11	AC099811.5	0.700430946	5.38E-71	postive
GSDME	AC060766.7	0.70566599	1.73E-72	postive
IL1A	AC060766.7	0.501176927	1.90E-31	postive
IL1B	AC060766.7	0.561609153	1.14E-40	postive
NLRC4	AC060766.7	0.632538492	3.16E-54	postive
NLRP3	AC060766.7	0.578554199	1.31E-43	postive
SCAF11	AC060766.7	0.586023962	5.84E-45	postive
GSDME	AC093627.4	0.421802645	7.92E-22	postive
CHMP2A	AC100774.1	-0.416662436	2.74E-21	negative
GSDME	AC100774.1	0.551264129	5.92E-39	postive
IL1A	AC100774.1	0.460534656	3.30E-26	postive
IL1B	AC100774.1	0.481346813	8.37E-29	postive
NLRC4	AC100774.1	0.462608012	1.85E-26	postive

Pyroptosis-related Genes	lncRNA	cor	pvalue	Regulation
SCAF11	AC100774.1	0.695493799	1.28E-69	postive
CHMP6	AC104109.4	-0.41334672	6.05E-21	negative
GSDME	AC104109.4	0.474480038	6.30E-28	postive
IL1A	AC104109.4	0.430599733	8.97E-23	postive
IL1B	AC104109.4	0.465158814	9.06E-27	postive
NLRC4	AC104109.4	0.439951133	8.24E-24	postive
NLRP3	AC104109.4	0.441922814	4.94E-24	postive
PJVK	AC104109.4	0.407069484	2.64E-20	postive
SCAF11	AC104109.4	0.697175085	4.39E-70	postive
NOD1	AL031600.1	0.405041583	4.22E-20	postive
PLCG1	AL031600.1	0.527631121	2.95E-35	postive
NOD1	TSPOAP1-AS1	0.423489111	5.24E-22	postive
PJVK	TSPOAP1-AS1	0.460154717	3.67E-26	postive
SCAF11	TSPOAP1-AS1	0.428565655	1.49E-22	postive
GSDME	AC106786.1	0.532101517	6.21E-36	postive
GSDME	AP003486.1	0.432366465	5.74E-23	postive
GPX4	AP003486.1	-0.427571581	1.91E-22	negative
NOD1	AP003486.1	0.425044959	3.57E-22	postive
PJVK	AP003486.1	0.461385054	2.61E-26	postive
SCAF11	AP003486.1	0.574630168	6.52E-43	postive
PJVK	AC005828.4	0.426603568	2.43E-22	postive
GSDME	MYOSLID	0.684859724	9.59E-67	postive
IL1A	MYOSLID	0.457652433	7.31E-26	postive
IL1B	MYOSLID	0.581846722	3.36E-44	postive
GSDMC	MYOSLID	0.487606492	1.28E-29	postive
IL6	MYOSLID	0.410417111	1.21E-20	postive
NLRC4	MYOSLID	0.629161175	1.68E-53	postive
NLRP3	MYOSLID	0.667312023	2.91E-62	postive
NOD1	LINC00628	0.415257448	3.84E-21	postive
SCAF11	LINC00628	0.447999863	9.95E-25	postive
IRF1	AC004865.2	0.401798638	8.88E-20	postive
NLRC4	AC004865.2	0.463940752	1.28E-26	postive
NLRP1	AC004865.2	0.53980419	4.00E-37	postive
PJVK	AC090578.2	0.514093928	2.89E-33	postive
GSDME	AC087392.5	0.457961695	6.72E-26	postive
IL1B	AC087392.5	0.4528928	2.68E-25	postive
NLRC4	AC087392.5	0.413647176	5.64E-21	postive
NLRP3	AC087392.5	0.434876885	3.04E-23	postive

Pyroptosis-related Genes	lncRNA	cor	pvalue	Regulation
CHMP2A	AC004832.4	-0.416426711	2.90E-21	negative
GSDME	AC004832.4	0.577225403	2.26E-43	postive
IL1A	AC004832.4	0.440883899	6.47E-24	postive
IL1B	AC004832.4	0.446993828	1.30E-24	postive
NLRC4	AC004832.4	0.491622661	3.75E-30	postive
PJVK	AC004832.4	0.451916597	3.49E-25	postive
SCAF11	AC004832.4	0.710436271	7.08E-74	postive
SCAF11	OVOL1-AS1	0.542334165	1.60E-37	postive
SCAF11	AC244517.7	0.420752563	1.02E-21	postive
CHMP2A	AC127164.1	-0.443440567	3.32E-24	negative
CHMP6	AC127164.1	-0.417751468	2.11E-21	negative
GSDME	AC127164.1	0.538127735	7.31E-37	postive
IL1A	AC127164.1	0.423513115	5.21E-22	postive
IL1B	AC127164.1	0.433952211	3.84E-23	postive
NLRC4	AC127164.1	0.475985279	4.06E-28	postive
SCAF11	AC127164.1	0.726373791	9.98E-79	postive
IL6	AC113346.1	0.437425977	1.58E-23	postive
CHMP2A	HECW2-AS1	-0.420384795	1.12E-21	negative
GSDME	HECW2-AS1	0.639752782	8.27E-56	postive
IL1A	HECW2-AS1	0.428253064	1.61E-22	postive
IL1B	HECW2-AS1	0.481930372	7.04E-29	postive
NLRC4	HECW2-AS1	0.575187687	5.20E-43	postive
NLRP3	HECW2-AS1	0.559134066	2.98E-40	postive
SCAF11	HECW2-AS1	0.56504452	2.99E-41	postive
GSDME	AL359880.1	0.474599204	6.08E-28	postive
NLRC4	AL359880.1	0.431070281	7.96E-23	postive
SCAF11	AL359880.1	0.655572371	1.98E-59	postive
IL1B	MED8-AS1	0.421784226	7.95E-22	postive
NLRP3	MED8-AS1	0.443650739	3.14E-24	postive
SCAF11	MED8-AS1	0.482386343	6.15E-29	postive
CHMP6	AL390195.3	-0.404658674	4.61E-20	negative
GSDME	AL390195.3	0.4598333	4.01E-26	postive
NLRC4	AL390195.3	0.404446293	4.84E-20	postive
PJVK	AL390195.3	0.463484794	1.45E-26	postive
SCAF11	AL390195.3	0.653615396	5.70E-59	postive
CHMP2A	CD44-AS1	-0.415812201	3.36E-21	negative
CHMP6	CD44-AS1	-0.409606754	1.46E-20	negative
GSDME	CD44-AS1	0.541616626	2.08E-37	postive

Pyroptosis-related Genes	lncRNA	cor	pvalue	Regulation
IL1A	CD44-AS1	0.507012913	2.93E-32	postive
IL1B	CD44-AS1	0.557695638	5.17E-40	postive
NLRC4	CD44-AS1	0.466369927	6.44E-27	postive
NLRP3	CD44-AS1	0.43292291	4.99E-23	postive
SCAF11	CD44-AS1	0.678947237	3.37E-65	postive
PJVK	AC108681.1	0.549938876	9.71E-39	postive
GSDME	RDH10-AS1	0.559413156	2.67E-40	postive
IL1A	RDH10-AS1	0.429976353	1.05E-22	postive
IL1B	RDH10-AS1	0.524271016	9.39E-35	postive
NLRC4	RDH10-AS1	0.560427752	1.81E-40	postive
SCAF11	RDH10-AS1	0.642511466	2.00E-56	postive
GSDME	AC091906.1	0.453203794	2.46E-25	postive
NLRC4	AC091906.1	0.410400383	1.21E-20	postive
PJVK	AC091906.1	0.44598171	1.70E-24	postive
SCAF11	AC091906.1	0.577351728	2.15E-43	postive
SCAF11	HDAC2-AS2	0.456792034	9.26E-26	postive
GSDME	AC068790.5	0.466720699	5.83E-27	postive
NOD1	AC068790.5	0.405388647	3.90E-20	postive
PJVK	AC068790.5	0.415674462	3.48E-21	postive
SCAF11	AC068790.5	0.68083443	1.09E-65	postive
NOD1	AL590004.3	0.408874315	1.73E-20	postive
NOD1	UBE2Q1-AS1	0.484174181	3.60E-29	postive
SCAF11	UBE2Q1-AS1	0.476755873	3.24E-28	postive
GSDME	LINC01711	0.496543288	8.17E-31	postive
GSDME	AC107884.1	0.50084739	2.11E-31	postive
NLRC4	AC107884.1	0.454777227	1.61E-25	postive
PJVK	AC107884.1	0.571243459	2.56E-42	postive
SCAF11	AC107884.1	0.485666052	2.30E-29	postive
PJVK	AC092119.2	0.522388008	1.79E-34	postive
ELANE	ZNF582-AS1	0.416100209	3.14E-21	postive
NLRP1	ZNF582-AS1	0.401152607	1.03E-19	postive
NOD1	AC093620.1	0.419529038	1.38E-21	postive
CHMP2A	AC026202.2	-0.438412735	1.23E-23	negative
GSDME	AC026202.2	0.511867091	6.02E-33	postive
IL1A	AC026202.2	0.429735204	1.11E-22	postive
IL1B	AC026202.2	0.481285443	8.53E-29	postive
NLRC4	AC026202.2	0.475521977	4.65E-28	postive
NLRP3	AC026202.2	0.457723246	7.17E-26	postive

Pyroptosis-related Genes	lncRNA	cor	pvalue	Regulation
SCAF11	AC026202.2	0.660455416	1.36E-60	postive
NOD1	C5orf66	0.420811739	1.01E-21	postive
NOD1	AL117379.1	0.439706794	8.78E-24	postive
PJVK	AL117379.1	0.492346553	3.00E-30	postive
SCAF11	AL117379.1	0.486582534	1.74E-29	postive
GSDME	AL662844.3	0.536788247	1.18E-36	postive
NLRC4	AL662844.3	0.421503313	8.52E-22	postive
NOD1	AL662844.3	0.533621561	3.63E-36	postive
SCAF11	AL662844.3	0.53286773	4.74E-36	postive
CHMP3	AL021707.6	-0.431544533	7.07E-23	negative
SCAF11	DSG2-AS1	0.502430448	1.28E-31	postive
GSDME	DUBR	0.656215398	1.39E-59	postive
NLRC4	DUBR	0.498066533	5.07E-31	postive
NLRP3	DUBR	0.422328347	6.96E-22	postive
SCAF11	DUBR	0.527686783	2.90E-35	postive
CHMP2A	AL035448.1	-0.402935457	6.85E-20	negative
GSDME	AL035448.1	0.521827451	2.16E-34	postive
IL1A	AL035448.1	0.40949969	1.50E-20	postive
SCAF11	AL035448.1	0.655225397	2.39E-59	postive
CHMP2A	AC015911.3	-0.449658082	6.39E-25	negative
GSDME	AC015911.3	0.548676909	1.55E-38	postive
NLRC4	AC015911.3	0.636392077	4.57E-55	postive
SCAF11	AC015911.3	0.713869902	6.81E-75	postive
CHMP2A	MCM3AP-AS1	-0.441971537	4.87E-24	negative
GSDME	MCM3AP-AS1	0.437090481	1.72E-23	postive
CASP8	MCM3AP-AS1	0.403864503	5.54E-20	postive
NOD1	MCM3AP-AS1	0.457510773	7.60E-26	postive
PJVK	MCM3AP-AS1	0.511115712	7.70E-33	postive
SCAF11	MCM3AP-AS1	0.686758885	3.00E-67	postive
CHMP2A	AC007216.2	-0.422090015	7.38E-22	negative
CHMP6	AC007216.2	-0.409232448	1.60E-20	negative
SCAF11	AC007216.2	0.620069602	1.37E-51	postive
GSDME	AC026356.2	0.554171587	1.98E-39	postive
IL1A	AC026356.2	0.4092763	1.58E-20	postive
NLRC4	AC026356.2	0.442358341	4.41E-24	postive
PJVK	AC026356.2	0.479506413	1.44E-28	postive
SCAF11	AC026356.2	0.637246667	2.96E-55	postive
PLCG1	AL596223.2	0.450705196	4.83E-25	postive

Pyroptosis-related Genes	lncRNA	cor	pvalue	Regulation
CHMP2A	AC006059.1	-0.422492774	6.69E-22	negative
GSDME	AC006059.1	0.547433256	2.46E-38	postive
IL1A	AC006059.1	0.404621264	4.65E-20	postive
IL1B	AC006059.1	0.409481195	1.50E-20	postive
NLRC4	AC006059.1	0.497109408	6.84E-31	postive
PJVK	AC006059.1	0.447164441	1.24E-24	postive
SCAF11	AC006059.1	0.752622527	1.63E-87	postive
GSDME	C2orf27A	0.630596281	8.28E-54	postive
NLRC4	C2orf27A	0.532432323	5.52E-36	postive
PJVK	C2orf27A	0.502535792	1.23E-31	postive
SCAF11	C2orf27A	0.605814968	1.02E-48	postive
PJVK	Z69733.1	0.492629384	2.75E-30	postive
PJVK	AC073254.1	0.444907771	2.26E-24	postive
SCAF11	AC073254.1	0.432512928	5.54E-23	postive
GSDME	AL121845.4	0.493473612	2.12E-30	postive
NLRC4	AL121845.4	0.401474997	9.56E-20	postive
NOD1	AL121845.4	0.44138743	5.68E-24	postive
SCAF11	AL121845.4	0.583835779	1.46E-44	postive
CHMP2A	MIR133A1HG	-0.412498965	7.40E-21	negative
GSDME	MIR133A1HG	0.600760512	9.87E-48	postive
IL1A	MIR133A1HG	0.479328017	1.52E-28	postive
IL1B	MIR133A1HG	0.506861986	3.08E-32	postive
NLRC4	MIR133A1HG	0.523338811	1.29E-34	postive
NLRP3	MIR133A1HG	0.407687804	2.29E-20	postive
SCAF11	MIR133A1HG	0.726039131	1.27E-78	postive
CHMP2A	AL158163.1	-0.418714285	1.68E-21	negative
GSDME	AL158163.1	0.575677091	4.26E-43	postive
IL1A	AL158163.1	0.427595087	1.90E-22	postive
IL1B	AL158163.1	0.40682311	2.80E-20	postive
NLRC4	AL158163.1	0.467531061	4.63E-27	postive
PJVK	AL158163.1	0.475719005	4.39E-28	postive
SCAF11	AL158163.1	0.694182829	2.95E-69	postive
GSDME	RNASEH2B-AS1	0.503183343	1.00E-31	postive
NOD1	RNASEH2B-AS1	0.48531342	2.56E-29	postive
PJVK	RNASEH2B-AS1	0.410681847	1.14E-20	postive
SCAF11	RNASEH2B-AS1	0.545779892	4.53E-38	postive
GSDME	AC004832.5	0.507442081	2.55E-32	postive
NLRC4	AC004832.5	0.426064178	2.78E-22	postive

Pyroptosis-related Genes	lncRNA	cor	pvalue	Regulation
PJVK	AC004832.5	0.44742068	1.16E-24	postive
SCAF11	AC004832.5	0.644236082	8.19E-57	postive
GSDME	AC010300.1	0.453429427	2.32E-25	postive
NOD1	AC010300.1	0.411548592	9.26E-21	postive
SCAF11	AC010300.1	0.579651965	8.34E-44	postive
CHMP2A	AL355075.2	-0.434538334	3.31E-23	negative
CHMP6	AL355075.2	-0.409052875	1.66E-20	negative
GSDME	AL355075.2	0.524917848	7.52E-35	postive
IL1A	AL355075.2	0.432142493	6.08E-23	postive
IL1B	AL355075.2	0.462139243	2.11E-26	postive
NLRC4	AL355075.2	0.474642474	6.01E-28	postive
PJVK	AL355075.2	0.468330929	3.69E-27	postive
SCAF11	AL355075.2	0.703338113	8.06E-72	postive
GSDME	LINC02595	0.507518665	2.49E-32	postive
NLRC4	LINC02595	0.445105816	2.14E-24	postive
SCAF11	LINC02595	0.629414088	1.48E-53	postive
NOD1	CRYZL2P-SEC16B	0.443128211	3.60E-24	postive
SCAF11	CRYZL2P-SEC16B	0.502921188	1.09E-31	postive
CHMP2A	MAGI1-IT1	-0.421549168	8.42E-22	negative
GSDME	MAGI1-IT1	0.535469923	1.89E-36	postive
IL1A	MAGI1-IT1	0.435339463	2.70E-23	postive
IL1B	MAGI1-IT1	0.428898237	1.37E-22	postive
NLRC4	MAGI1-IT1	0.4518641	3.54E-25	postive
PJVK	MAGI1-IT1	0.403143908	6.53E-20	postive
SCAF11	MAGI1-IT1	0.694152519	3.00E-69	postive
GSDME	AP000255.1	0.41154484	9.27E-21	postive
PJVK	AP000255.1	0.422087955	7.38E-22	postive
SCAF11	AP000255.1	0.467290988	4.96E-27	postive
GSDME	FAM78B-AS1	0.462200212	2.08E-26	postive
NLRC4	FAM78B-AS1	0.404609343	4.66E-20	postive
NOD1	FAM78B-AS1	0.429631884	1.14E-22	postive
SCAF11	FAM78B-AS1	0.483668972	4.19E-29	postive
GSDME	AC018648.1	0.416956741	2.56E-21	postive
NOD1	AC018648.1	0.493038696	2.43E-30	postive
PJVK	AC018648.1	0.427415387	1.99E-22	postive
SCAF11	AC018648.1	0.51213965	5.50E-33	postive
SCAF11	AC092903.2	0.505340414	5.02E-32	postive
GSDME	AC114781.2	0.528282136	2.36E-35	postive

Pyroptosis-related Genes	lncRNA	cor	pvalue	Regulation
IL1A	AC114781.2	0.454097148	1.93E-25	postive
IL1B	AC114781.2	0.493425739	2.15E-30	postive
NLRC4	AC114781.2	0.451240987	4.18E-25	postive
NLRP3	AC114781.2	0.505254329	5.17E-32	postive
PJVK	AC114781.2	0.428802623	1.41E-22	postive
SCAF11	AC114781.2	0.582115321	3.00E-44	postive
GSDME	AL360093.1	0.508430565	1.85E-32	postive
NLRC4	AL360093.1	0.452244081	3.19E-25	postive
SCAF11	AL360093.1	0.640365167	6.05E-56	postive
CHMP2A	NPTN-IT1	-0.452064333	3.35E-25	negative
CHMP6	NPTN-IT1	-0.416917439	2.58E-21	negative
GSDME	NPTN-IT1	0.524728618	8.03E-35	postive
IL1A	NPTN-IT1	0.435348024	2.69E-23	postive
IL1B	NPTN-IT1	0.468304642	3.72E-27	postive
NLRC4	NPTN-IT1	0.445698901	1.83E-24	postive
NLRP3	NPTN-IT1	0.467552676	4.60E-27	postive
SCAF11	NPTN-IT1	0.672905723	1.17E-63	postive
NOD1	AC139795.2	0.4660521	7.04E-27	postive
PJVK	AC139795.2	0.491751243	3.61E-30	postive
SCAF11	AC139795.2	0.430072923	1.02E-22	postive
NOD1	AL731571.1	0.462486933	1.92E-26	postive
SCAF11	AL731571.1	0.401679294	9.13E-20	postive
TIRAP	AL731571.1	0.465812024	7.54E-27	postive
GSDME	FOCAD-AS1	0.453121885	2.52E-25	postive
SCAF11	FOCAD-AS1	0.571713622	2.12E-42	postive
NOD1	AL121906.1	0.421216858	9.13E-22	postive
SCAF11	AL121906.1	0.517588102	9.02E-34	postive
PJVK	AP002812.2	0.424710319	3.88E-22	postive
CHMP2A	AC004067.1	-0.416351431	2.96E-21	negative
GSDME	AC004067.1	0.41049681	1.19E-20	postive
CASP8	AC004067.1	0.40102169	1.06E-19	postive
NOD1	AC004067.1	0.469888484	2.37E-27	postive
PJVK	AC004067.1	0.450740047	4.78E-25	postive
SCAF11	AC004067.1	0.660093821	1.66E-60	postive
CHMP4A	AL139287.1	0.423171348	5.66E-22	postive
GSDME	AC109597.2	0.563199921	6.16E-41	postive
IL1A	AC109597.2	0.428451959	1.53E-22	postive
IL1B	AC109597.2	0.448073456	9.76E-25	postive

Pyroptosis-related Genes	lncRNA	cor	pvalue	Regulation
NLRC4	AC109597.2	0.476467297	3.53E-28	postive
SCAF11	AC109597.2	0.670715785	4.16E-63	postive
CHMP4A	AC005519.1	0.480466333	1.09E-28	postive
GSDME	AC005519.1	0.445657609	1.85E-24	postive
NOD1	AC005519.1	0.44474293	2.36E-24	postive
PJVK	AC005519.1	0.462415917	1.96E-26	postive
SCAF11	AC005519.1	0.540194456	3.47E-37	postive
PJVK	ZNF32-AS1	0.404943959	4.32E-20	postive
SCAF11	ZNF32-AS1	0.424796796	3.80E-22	postive
GSDME	AC091182.1	0.562329166	8.65E-41	postive
IL1A	AC091182.1	0.414499104	4.60E-21	postive
IL1B	AC091182.1	0.502364356	1.30E-31	postive
NLRC4	AC091182.1	0.484532979	3.23E-29	postive
NLRP3	AC091182.1	0.598824086	2.33E-47	postive
CHMP2A	GLYCTK-AS1	-0.424810938	3.79E-22	negative
GSDME	GLYCTK-AS1	0.46349517	1.45E-26	postive
NLRC4	GLYCTK-AS1	0.41483639	4.25E-21	postive
NOD1	GLYCTK-AS1	0.402983667	6.78E-20	postive
PJVK	GLYCTK-AS1	0.431574747	7.01E-23	postive
SCAF11	GLYCTK-AS1	0.689296003	6.28E-68	postive
CHMP2A	ENTPD1-AS1	-0.473534129	8.28E-28	negative
GSDME	ENTPD1-AS1	0.555441575	1.22E-39	postive
NLRC4	ENTPD1-AS1	0.483635206	4.23E-29	postive
NOD1	ENTPD1-AS1	0.409614756	1.46E-20	postive
PJVK	ENTPD1-AS1	0.460357536	3.47E-26	postive
SCAF11	ENTPD1-AS1	0.722336486	1.82E-77	postive
GSDME	AP006333.3	0.405381547	3.90E-20	postive
SCAF11	AP006333.3	0.530500635	1.09E-35	postive
PJVK	TAPT1-AS1	0.453523686	2.26E-25	postive
SCAF11	AL451042.1	0.43074727	8.64E-23	postive
TIRAP	AL645941.1	0.427469093	1.96E-22	postive
CHMP2A	AC109992.2	-0.411293509	9.83E-21	negative
GSDME	AC109992.2	0.448888718	7.85E-25	postive
NOD1	AC109992.2	0.423734566	4.93E-22	postive
PJVK	AC109992.2	0.433540108	4.27E-23	postive
SCAF11	AC109992.2	0.721231631	3.99E-77	postive
GSDME	AC068491.3	0.590260949	9.63E-46	postive
IL1A	AC068491.3	0.433224074	4.62E-23	postive

Pyroptosis-related Genes	lncRNA	cor	pvalue	Regulation
IL1B	AC068491.3	0.484511369	3.25E-29	postive
NLRC4	AC068491.3	0.485197367	2.65E-29	postive
NLRP3	AC068491.3	0.448334588	9.11E-25	postive
SCAF11	AC068491.3	0.599827876	1.49E-47	postive
NOD1	AC137770.1	0.479893921	1.29E-28	postive
PJVK	AC137770.1	0.402151939	8.20E-20	postive
SCAF11	AC137770.1	0.47531345	4.94E-28	postive
CHMP2A	AC096564.1	-0.404025393	5.34E-20	negative
GSDME	AC096564.1	0.447228001	1.22E-24	postive
SCAF11	AC096564.1	0.668044139	1.92E-62	postive
GSDME	AL121672.1	0.457942457	6.75E-26	postive
NOD1	AL121672.1	0.429205417	1.27E-22	postive
SCAF11	AL121672.1	0.575578662	4.43E-43	postive
PJVK	AC010655.2	0.456708873	9.48E-26	postive
SCAF11	AC010655.2	0.592383887	3.87E-46	postive
GSDME	LINC02328	0.580010948	7.19E-44	postive
IL1B	LINC02328	0.457275896	8.11E-26	postive
NLRC4	LINC02328	0.624892762	1.35E-52	postive
NLRP3	LINC02328	0.5304716	1.10E-35	postive
SCAF11	LINC02328	0.534421866	2.74E-36	postive
GSDME	AC139887.2	0.440715674	6.76E-24	postive
NOD1	AC139887.2	0.530164496	1.22E-35	postive
SCAF11	AC139887.2	0.549202855	1.28E-38	postive
TIRAP	AC139887.2	0.452763243	2.77E-25	postive
CHMP2A	AC002128.2	-0.402518067	7.54E-20	negative
GSDME	AC002128.2	0.440571248	7.02E-24	postive
NOD1	AC002128.2	0.440000921	8.14E-24	postive
SCAF11	AC002128.2	0.652362432	1.12E-58	postive
PJVK	AC008669.1	0.496328485	8.73E-31	postive
SCAF11	AC008669.1	0.623200619	3.06E-52	postive
CHMP2A	AC022001.2	-0.425712021	3.03E-22	negative
CHMP6	AC022001.2	-0.404679128	4.59E-20	negative
GSDME	AC022001.2	0.569550152	5.03E-42	postive
IL1A	AC022001.2	0.482062284	6.77E-29	postive
IL1B	AC022001.2	0.509414485	1.34E-32	postive
NLRC4	AC022001.2	0.471190941	1.63E-27	postive
NLRP3	AC022001.2	0.458952971	5.11E-26	postive
SCAF11	AC022001.2	0.677529309	7.81E-65	postive

Pyroptosis-related Genes	lncRNA	cor	pvalue	Regulation
NOD1	DTX2P1-UPK3BP1-PMS2P11	0.451476437	3.92E-25	postive
SCAF11	DTX2P1-UPK3BP1-PMS2P11	0.414012865	5.17E-21	postive
GSDME	AP002993.1	0.476658183	3.34E-28	postive
SCAF11	AP002993.1	0.637923774	2.10E-55	postive
GSDME	AC009093.1	0.627287562	4.21E-53	postive
IL1A	AC009093.1	0.468862254	3.17E-27	postive
IL1B	AC009093.1	0.518182143	7.40E-34	postive
NLRC4	AC009093.1	0.444086279	2.80E-24	postive
NLRP3	AC009093.1	0.610444444	1.24E-49	postive
SCAF11	AC009093.1	0.452507943	2.97E-25	postive
CHMP2A	AL356599.1	-0.418137761	1.93E-21	negative
GSDME	AL356599.1	0.590466152	8.82E-46	postive
NLRC4	AL356599.1	0.491392905	4.03E-30	postive
NOD1	AL356599.1	0.416398391	2.92E-21	postive
PJVK	AL356599.1	0.466577876	6.07E-27	postive
SCAF11	AL356599.1	0.690643566	2.72E-68	postive
SCAF11	EIPR1-IT1	0.507372479	2.61E-32	postive
CASP6	AC098487.1	0.407499447	2.39E-20	postive
CHMP2A	AC109347.2	-0.402761785	7.13E-20	negative
GSDME	AC109347.2	0.421014627	9.59E-22	postive
NLRC4	AC109347.2	0.418282059	1.86E-21	postive
SCAF11	AC109347.2	0.598869475	2.28E-47	postive
PJVK	PRR7-AS1	0.416506157	2.85E-21	postive
GSDMD	AC138696.2	0.486453149	1.81E-29	postive
GPX4	AC138696.2	0.456054254	1.13E-25	postive
NOD1	C1orf220	0.506282906	3.71E-32	postive
PJVK	C1orf220	0.489349298	7.53E-30	postive
SCAF11	C1orf220	0.413607101	5.69E-21	postive
GSDME	MIRLET7A1HG	0.457211045	8.26E-26	postive
NLRC4	MIRLET7A1HG	0.424352575	4.24E-22	postive
PJVK	MIRLET7A1HG	0.52335592	1.28E-34	postive
SCAF11	MIRLET7A1HG	0.702346499	1.54E-71	postive
NLRC4	AC026369.3	0.474414171	6.42E-28	postive
CHMP2A	AC068790.3	-0.405120645	4.15E-20	negative
GSDME	AC068790.3	0.500726566	2.19E-31	postive
NLRC4	AC068790.3	0.429620453	1.15E-22	postive
PJVK	AC068790.3	0.408173252	2.04E-20	postive
SCAF11	AC068790.3	0.677909148	6.23E-65	postive

Pyroptosis-related Genes	lncRNA	cor	pvalue	Regulation
CHMP2A	AC096741.1	-0.417795842	2.09E-21	negative
GSDME	AC096741.1	0.494536891	1.53E-30	postive
NLRC4	AC096741.1	0.425820736	2.95E-22	postive
PJVK	AC096741.1	0.446935852	1.32E-24	postive
SCAF11	AC096741.1	0.685181606	7.89E-67	postive
CHMP2A	AP000786.1	-0.420787431	1.01E-21	negative
GSDME	AP000786.1	0.426691981	2.38E-22	postive
PJVK	AP000786.1	0.407749384	2.25E-20	postive
SCAF11	AP000786.1	0.656010717	1.56E-59	postive
PJVK	AC008429.1	0.451650071	3.75E-25	postive
SCAF11	AC008429.1	0.415519952	3.61E-21	postive
GSDME	AC092535.5	0.473395502	8.62E-28	postive
NLRC4	AC092535.5	0.402579491	7.43E-20	postive
PJVK	HEXA-AS1	0.48782599	1.20E-29	postive
GSDME	AC083967.1	0.443718117	3.09E-24	postive
NLRC4	AC083967.1	0.522064711	1.99E-34	postive
GSDME	AC026771.1	0.406384381	3.10E-20	postive
PJVK	AC026771.1	0.423328422	5.45E-22	postive
SCAF11	AC026771.1	0.621432612	7.15E-52	postive
GSDME	AC107993.1	0.568888926	6.55E-42	postive
IL1A	AC107993.1	0.459653238	4.21E-26	postive
IL1B	AC107993.1	0.48558541	2.36E-29	postive
NLRC4	AC107993.1	0.47143036	1.52E-27	postive
NLRP3	AC107993.1	0.423495008	5.23E-22	postive
SCAF11	AC107993.1	0.633474446	1.98E-54	postive
CHMP2A	TH2LCRR	-0.41286226	6.79E-21	negative
GSDME	TH2LCRR	0.517944234	8.01E-34	postive
NLRC4	TH2LCRR	0.438177744	1.30E-23	postive
PJVK	TH2LCRR	0.44038418	7.37E-24	postive
SCAF11	TH2LCRR	0.684137685	1.49E-66	postive
SCAF11	AC004551.1	0.540627811	2.97E-37	postive
NOD1	AL450384.2	0.446785408	1.37E-24	postive
PJVK	AL450384.2	0.508653983	1.72E-32	postive
CHMP2A	AC015849.3	-0.460601122	3.24E-26	negative
PJVK	AC015849.3	0.457086467	8.54E-26	postive
SCAF11	AC015849.3	0.672558197	1.43E-63	postive
SCAF11	AC006449.2	0.432309255	5.83E-23	postive
CHMP2A	CDC42-IT1	-0.419193225	1.49E-21	negative

Pyroptosis-related Genes	lncRNA	cor	pvalue	Regulation
GSDME	CDC42-IT1	0.476632273	3.36E-28	postive
NLRC4	CDC42-IT1	0.416932817	2.57E-21	postive
PJVK	CDC42-IT1	0.454086531	1.94E-25	postive
SCAF11	CDC42-IT1	0.711460327	3.54E-74	postive
GSDME	ACTA2-AS1	0.641556598	3.28E-56	postive
NLRC4	ACTA2-AS1	0.519268931	5.13E-34	postive
NLRP3	ACTA2-AS1	0.465807343	7.55E-27	postive
NOD1	ACTA2-AS1	0.400487417	1.20E-19	postive
PJVK	ACTA2-AS1	0.458749487	5.41E-26	postive
SCAF11	ACTA2-AS1	0.528171989	2.45E-35	postive
GSDME	PRR26	0.450148791	5.61E-25	postive
IL1A	PRR26	0.454001694	1.98E-25	postive
IL1B	PRR26	0.469921088	2.34E-27	postive
NLRP3	PRR26	0.449863744	6.05E-25	postive
SCAF11	PRR26	0.539297451	4.80E-37	postive
NOD1	AC008758.2	0.435960098	2.30E-23	postive
PJVK	AC008758.2	0.5191255	5.39E-34	postive
SCAF11	AC008758.2	0.5417685	1.96E-37	postive
GSDME	GEMIN7-AS1	0.426748358	2.34E-22	postive
SCAF11	GEMIN7-AS1	0.407148016	2.59E-20	postive
PJVK	PPP1R12A-AS1	0.423772992	4.89E-22	postive
SCAF11	PPP1R12A-AS1	0.463337269	1.51E-26	postive
PJVK	AC034229.4	0.565694189	2.32E-41	postive
SCAF11	AC034229.4	0.564443983	3.79E-41	postive
GSDME	CARMN	0.545800384	4.50E-38	postive
NLRC4	CARMN	0.454533604	1.72E-25	postive
SCAF11	CARMN	0.616504167	7.40E-51	postive
CASP8	WARS2-AS1	0.415414316	3.70E-21	postive
PJVK	WARS2-AS1	0.618050761	3.57E-51	postive
SCAF11	WARS2-AS1	0.572454016	1.57E-42	postive
GSDME	AC005776.2	0.515170444	2.02E-33	postive
IL1A	AC005776.2	0.438172418	1.31E-23	postive
IL1B	AC005776.2	0.488525752	9.67E-30	postive
NLRC4	AC005776.2	0.45080018	4.71E-25	postive
NLRP3	AC005776.2	0.433290358	4.55E-23	postive
PJVK	AC005776.2	0.502023086	1.45E-31	postive
SCAF11	AC005776.2	0.595334666	1.08E-46	postive
SCAF11	AC005046.2	0.576328109	3.27E-43	postive

Pyroptosis-related Genes	lncRNA	cor	pvalue	Regulation
PJVK	AC055822.1	0.545651757	4.75E-38	postive
SCAF11	AC055822.1	0.608748267	2.70E-49	postive
CASP8	AC068620.2	0.403220014	6.42E-20	postive
PJVK	AC068620.2	0.61376292	2.67E-50	postive
SCAF11	AC068620.2	0.602803699	3.97E-48	postive
GSDME	AL008729.1	0.617039217	5.75E-51	postive
IL1A	AL008729.1	0.467271268	4.99E-27	postive
IL1B	AL008729.1	0.559113517	3.00E-40	postive
NLRC4	AL008729.1	0.517818512	8.35E-34	postive
NLRP3	AL008729.1	0.500319566	2.49E-31	postive
NOD1	AL008729.1	0.426305287	2.62E-22	postive
SCAF11	AL008729.1	0.559729154	2.37E-40	postive
CHMP2A	Z93930.3	-0.430855596	8.41E-23	negative
GSDME	Z93930.3	0.447488494	1.14E-24	postive
NLRC4	Z93930.3	0.418687744	1.69E-21	postive
NOD1	Z93930.3	0.405489968	3.81E-20	postive
PJVK	Z93930.3	0.429340791	1.23E-22	postive
SCAF11	Z93930.3	0.578387066	1.41E-43	postive
GSDME	AC009299.2	0.457908754	6.82E-26	postive
NLRC4	AC009299.2	0.41941993	1.41E-21	postive
PJVK	AC009299.2	0.542785921	1.36E-37	postive
SCAF11	AC009299.2	0.652262221	1.18E-58	postive
GSDME	AC069549.1	0.553861937	2.23E-39	postive
IL1A	AC069549.1	0.546932984	2.96E-38	postive
IL1B	AC069549.1	0.635777738	6.23E-55	postive
NLRC4	AC069549.1	0.478372003	2.02E-28	postive
NLRP3	AC069549.1	0.525883598	5.40E-35	postive
SCAF11	AC069549.1	0.590429825	8.96E-46	postive
CHMP4A	AC005387.2	0.403299319	6.30E-20	postive
CHMP2A	AC137932.2	-0.402086284	8.32E-20	negative
GSDME	AC137932.2	0.486419367	1.83E-29	postive
NLRC4	AC137932.2	0.408432544	1.92E-20	postive
SCAF11	AC137932.2	0.577077971	2.40E-43	postive
CHMP2A	PKP4-AS1	-0.416274385	3.01E-21	negative
CHMP6	PKP4-AS1	-0.410744329	1.12E-20	negative
GSDME	PKP4-AS1	0.517652238	8.83E-34	postive
NLRC4	PKP4-AS1	0.430182535	9.96E-23	postive
SCAF11	PKP4-AS1	0.641457856	3.45E-56	postive

Pyroptosis-related Genes	lncRNA	cor	pvalue	Regulation
GSDME	WNT5A-AS1	0.542849587	1.33E-37	postive
IL1B	WNT5A-AS1	0.445526508	1.92E-24	postive
GSDMC	WNT5A-AS1	0.493980144	1.81E-30	postive
NLRC4	WNT5A-AS1	0.561358316	1.26E-40	postive
NLRP3	WNT5A-AS1	0.50712727	2.82E-32	postive
GSDME	POC1B-AS1	0.41152493	9.31E-21	postive
NOD1	POC1B-AS1	0.427370876	2.01E-22	postive
SCAF11	POC1B-AS1	0.521641362	2.30E-34	postive
GSDME	AC245128.3	0.554356587	1.85E-39	postive
IL1A	AC245128.3	0.737666268	2.23E-82	postive
IL1B	AC245128.3	0.929732578	1.75E-206	postive
NLRC4	AC245128.3	0.496550457	8.15E-31	postive
NLRP3	AC245128.3	0.794024054	7.00E-104	postive
GSDME	AL445489.1	0.438509147	1.20E-23	postive
SCAF11	AL445489.1	0.534470009	2.69E-36	postive
GSDME	ARNTL2-AS1	0.575550973	4.48E-43	postive
IL1A	ARNTL2-AS1	0.439411541	9.48E-24	postive
IL1B	ARNTL2-AS1	0.516681437	1.22E-33	postive
NLRC4	ARNTL2-AS1	0.560587975	1.70E-40	postive
NLRP3	ARNTL2-AS1	0.401144469	1.03E-19	postive
SCAF11	ARNTL2-AS1	0.698925274	1.42E-70	postive
PJVK	AC079148.1	0.405933131	3.44E-20	postive
SCAF11	AC079148.1	0.4773845	2.70E-28	postive
CASP1	LINC00114	0.42025989	1.15E-21	postive
GSDME	U91328.1	0.409075701	1.65E-20	postive
SCAF11	U91328.1	0.418178057	1.91E-21	postive
CHMP2A	AC020978.7	-0.409937357	1.35E-20	negative
CHMP6	AC020978.7	-0.422601448	6.51E-22	negative
GSDME	AC020978.7	0.46978801	2.43E-27	postive
NLRC4	AC020978.7	0.419162648	1.50E-21	postive
PJVK	AC020978.7	0.476672281	3.32E-28	postive
SCAF11	AC020978.7	0.682434931	4.17E-66	postive
GSDME	AC022165.1	0.511983228	5.80E-33	postive
NLRP3	AC022165.1	0.47284463	1.01E-27	postive
SCAF11	AC022165.1	0.570910895	2.92E-42	postive
SCAF11	AF131215.4	0.500757994	2.17E-31	postive
GSDME	AC003991.1	0.520284928	3.64E-34	postive
IL1A	AC003991.1	0.559030624	3.10E-40	postive

Pyroptosis-related Genes	lncRNA	cor	pvalue	Regulation
IL1B	AC003991.1	0.647233132	1.71E-57	postive
NLRC4	AC003991.1	0.446250689	1.58E-24	postive
NLRP3	AC003991.1	0.545844191	4.43E-38	postive
SCAF11	AC003991.1	0.620844642	9.47E-52	postive
SCAF11	HCFC1-AS1	0.486956219	1.56E-29	postive
NOD1	AC006017.1	0.408733302	1.79E-20	postive
PJVK	AC006017.1	0.467354255	4.87E-27	postive
SCAF11	AC006017.1	0.665977583	6.21E-62	postive
NOD1	AL021408.1	0.42609509	2.76E-22	postive
GSDME	AL139147.1	0.54799251	2.00E-38	postive
IL1A	AL139147.1	0.602442527	4.66E-48	postive
IL1B	AL139147.1	0.670298174	5.29E-63	postive
NLRP3	AL139147.1	0.605298988	1.29E-48	postive
SCAF11	AL139147.1	0.542128185	1.72E-37	postive
GSDME	AC090517.2	0.409899894	1.36E-20	postive
PJVK	AC090517.2	0.590877032	7.40E-46	postive
SCAF11	AC090517.2	0.598277673	2.96E-47	postive
PJVK	AL365330.1	0.400451813	1.21E-19	postive
NOD1	LINC01126	0.501475258	1.73E-31	postive
PJVK	LINC01126	0.44095223	6.36E-24	postive
SCAF11	LINC01126	0.498523933	4.39E-31	postive
GSDME	AL355073.2	0.474748489	5.82E-28	postive
NOD1	AL355073.2	0.400359975	1.23E-19	postive
PJVK	AL355073.2	0.449519079	6.64E-25	postive
SCAF11	AL355073.2	0.511001603	8.00E-33	postive
SCAF11	AC073575.4	0.458329546	6.07E-26	postive
NOD1	AC092953.2	0.404417162	4.88E-20	postive
PJVK	AC092953.2	0.445638296	1.86E-24	postive
SCAF11	AC092953.2	0.670289078	5.32E-63	postive
CHMP2A	AC018926.1	-0.413953434	5.24E-21	negative
GSDME	AC018926.1	0.541883104	1.88E-37	postive
NLRC4	AC018926.1	0.456108938	1.12E-25	postive
SCAF11	AC018926.1	0.684326725	1.33E-66	postive
GSDME	AC006504.1	0.52027793	3.65E-34	postive
NLRC4	AC006504.1	0.414641453	4.45E-21	postive
NOD1	AC006504.1	0.447531236	1.13E-24	postive
PJVK	AC006504.1	0.423185028	5.65E-22	postive
SCAF11	AC006504.1	0.555629133	1.14E-39	postive

Pyroptosis-related Genes	lncRNA	cor	pvalue	Regulation
CHMP2A	AC130371.1	-0.403321837	6.27E-20	negative
GSDME	AC130371.1	0.578053534	1.61E-43	postive
IL1A	AC130371.1	0.506724931	3.22E-32	postive
IL1B	AC130371.1	0.550050134	9.32E-39	postive
NLRC4	AC130371.1	0.482543789	5.86E-29	postive
NLRP3	AC130371.1	0.479223734	1.57E-28	postive
SCAF11	AC130371.1	0.665814372	6.81E-62	postive
SCAF11	HNF1A-AS1	0.490736765	4.92E-30	postive
NOD1	THUMPD3-AS1	0.418516307	1.76E-21	postive
PJVK	THUMPD3-AS1	0.560039838	2.10E-40	postive
SCAF11	THUMPD3-AS1	0.527985305	2.61E-35	postive
GSDME	LURAP1L-AS1	0.468696997	3.32E-27	postive
PJVK	LURAP1L-AS1	0.470441041	2.02E-27	postive
SCAF11	LURAP1L-AS1	0.663821391	2.09E-61	postive
GSDME	AC104825.1	0.577775696	1.81E-43	postive
IL1B	AC104825.1	0.401092365	1.04E-19	postive
NLRC4	AC104825.1	0.405583548	3.73E-20	postive
NLRP3	AC104825.1	0.488593616	9.48E-30	postive
GSDME	FTX	0.482081982	6.73E-29	postive
PJVK	FTX	0.421021261	9.58E-22	postive
SCAF11	FTX	0.646902742	2.03E-57	postive
PJVK	SLC16A1-AS1	0.474466711	6.32E-28	postive
SCAF11	SLC16A1-AS1	0.51126728	7.33E-33	postive
GSDME	AL136985.2	0.475809255	4.28E-28	postive
NLRC4	AL136985.2	0.406571897	2.96E-20	postive
SCAF11	AL136985.2	0.549827252	1.01E-38	postive
CHMP2A	LINC01578	-0.423385643	5.37E-22	negative
CHMP6	LINC01578	-0.402403551	7.74E-20	negative
GSDME	LINC01578	0.555129678	1.38E-39	postive
IL1A	LINC01578	0.456608676	9.74E-26	postive
IL1B	LINC01578	0.511048478	7.88E-33	postive
NLRC4	LINC01578	0.501832099	1.54E-31	postive
NLRP3	LINC01578	0.40876052	1.78E-20	postive
PJVK	LINC01578	0.432900508	5.02E-23	postive
SCAF11	LINC01578	0.734449673	2.56E-81	postive
PJVK	AP001574.1	0.456491918	1.01E-25	postive
CHMP2A	AL158825.2	-0.422317112	6.98E-22	negative
CHMP6	AL158825.2	-0.411172516	1.01E-20	negative

Pyroptosis-related Genes	lncRNA	cor	pvalue	Regulation
GSDME	AL158825.2	0.465251943	8.83E-27	postive
NLRC4	AL158825.2	0.425529356	3.17E-22	postive
PJVK	AL158825.2	0.415515463	3.61E-21	postive
SCAF11	AL158825.2	0.759505775	5.29E-90	postive
GSDME	AC012358.4	0.569320296	5.51E-42	postive
IL1A	AC012358.4	0.499750763	2.98E-31	postive
IL1B	AC012358.4	0.601414183	7.38E-48	postive
NLRC4	AC012358.4	0.517440632	9.48E-34	postive
NLRP3	AC012358.4	0.467595219	4.55E-27	postive
SCAF11	AC012358.4	0.623628019	2.49E-52	postive
GSDME	RPS6KA2-IT1	0.554219717	1.94E-39	postive
IL1B	RPS6KA2-IT1	0.442988279	3.74E-24	postive
NLRC4	RPS6KA2-IT1	0.515385345	1.88E-33	postive
NLRP3	RPS6KA2-IT1	0.43978841	8.60E-24	postive
SCAF11	RPS6KA2-IT1	0.538092931	7.40E-37	postive
GSDME	ECE1-AS1	0.489051951	8.24E-30	postive
NLRC4	ECE1-AS1	0.448104829	9.68E-25	postive
NLRP3	ECE1-AS1	0.470813875	1.81E-27	postive
SCAF11	ECE1-AS1	0.540633618	2.96E-37	postive
BAK1	USP30-AS1	0.567047855	1.36E-41	postive
CASP1	USP30-AS1	0.495351047	1.18E-30	postive
IRF1	USP30-AS1	0.756777663	5.25E-89	postive
AIM2	USP30-AS1	0.56443211	3.80E-41	postive
GZMA	USP30-AS1	0.554581451	1.69E-39	postive
GSDME	PHC2-AS1	0.460673107	3.18E-26	postive
NLRC4	PHC2-AS1	0.428115367	1.67E-22	postive
PJVK	PHC2-AS1	0.451132023	4.31E-25	postive
SCAF11	PHC2-AS1	0.575122081	5.34E-43	postive
CHMP2A	EIF1AX-AS1	-0.40636389	3.11E-20	negative
GSDME	EIF1AX-AS1	0.424728574	3.86E-22	postive
NLRC4	EIF1AX-AS1	0.401740336	9.00E-20	postive
SCAF11	EIF1AX-AS1	0.6945921	2.27E-69	postive
NOD1	AL590096.1	0.446524509	1.47E-24	postive
CHMP2A	AC004908.1	-0.414017041	5.16E-21	negative
CHMP6	AC004908.1	-0.411871408	8.58E-21	negative
PJVK	AC004908.1	0.409321378	1.56E-20	postive
SCAF11	AC004908.1	0.593142944	2.79E-46	postive
GSDME	AL137782.1	0.504688516	6.20E-32	postive

Pyroptosis-related Genes	lncRNA	cor	pvalue	Regulation
IL1A	AL137782.1	0.418914419	1.60E-21	postive
IL1B	AL137782.1	0.486766848	1.65E-29	postive
NLRC4	AL137782.1	0.454075093	1.94E-25	postive
NLRP3	AL137782.1	0.459578088	4.30E-26	postive
SCAF11	AL137782.1	0.582104322	3.02E-44	postive
SCAF11	AC010524.1	0.457763532	7.09E-26	postive
PJVK	AL596223.1	0.409563727	1.48E-20	postive
SCAF11	AL596223.1	0.563541935	5.39E-41	postive
CHMP2A	AC002558.2	-0.428723002	1.43E-22	negative
CHMP6	AC002558.2	-0.421505506	8.51E-22	negative
GSDME	AC002558.2	0.507017942	2.93E-32	postive
IL1A	AC002558.2	0.42118158	9.21E-22	postive
IL1B	AC002558.2	0.445800883	1.78E-24	postive
NLRC4	AC002558.2	0.463808099	1.32E-26	postive
NLRP3	AC002558.2	0.446354802	1.54E-24	postive
SCAF11	AC002558.2	0.696533469	6.61E-70	postive
NOD1	AC073111.1	0.422860555	6.11E-22	postive
PJVK	AC073111.1	0.526977259	3.70E-35	postive
GSDME	AC007036.2	0.473040403	9.56E-28	postive
NLRP3	AC007036.2	0.420560897	1.07E-21	postive
SCAF11	AC007036.2	0.570618361	3.28E-42	postive
IRF1	XXYLT1-AS2	0.433740304	4.06E-23	postive
GSDME	AC048344.4	0.528132037	2.48E-35	postive
NLRC4	AC048344.4	0.450634862	4.92E-25	postive
PJVK	AC048344.4	0.496898014	7.31E-31	postive
SCAF11	AC048344.4	0.650281809	3.40E-58	postive
CHMP2A	AC087521.2	-0.425373059	3.30E-22	negative
GSDME	AC087521.2	0.519591385	4.60E-34	postive
IL1B	AC087521.2	0.411867425	8.59E-21	postive
NLRC4	AC087521.2	0.471272649	1.59E-27	postive
SCAF11	AC087521.2	0.715600628	2.06E-75	postive
PLCG1	AL671710.1	0.457752891	7.12E-26	postive
NOD1	LINC02615	0.426553115	2.46E-22	postive
PJVK	LINC02615	0.587480467	3.15E-45	postive
GSDME	TM4SF19-AS1	0.442283403	4.49E-24	postive
NLRC4	TM4SF19-AS1	0.407451513	2.42E-20	postive
PJVK	TM4SF19-AS1	0.450950294	4.52E-25	postive
CHMP2A	LNCSRLR	-0.403016701	6.72E-20	negative

Pyroptosis-related Genes	lncRNA	cor	pvalue	Regulation
GSDME	LNCSRLR	0.537717894	8.46E-37	postive
NLRC4	LNCSRLR	0.527153229	3.48E-35	postive
PJVK	LNCSRLR	0.475176955	5.14E-28	postive
SCAF11	LNCSRLR	0.71201603	2.42E-74	postive
SCAF11	AL451069.2	0.442883081	3.84E-24	postive
PJVK	AC008870.2	0.534265246	2.89E-36	postive
SCAF11	AC008870.2	0.59451137	1.54E-46	postive
GSDME	AP001528.1	0.54195921	1.83E-37	postive
NLRC4	AP001528.1	0.473284086	8.91E-28	postive
SCAF11	AP001528.1	0.607520914	4.72E-49	postive
CHMP4A	AC116914.2	0.419997599	1.23E-21	postive
SCAF11	AC116914.2	0.433159092	4.70E-23	postive
GSDMA	AC131025.1	0.421333237	8.88E-22	postive
GSDME	AC018695.4	0.452419889	3.04E-25	postive
NOD1	AC018695.4	0.444300744	2.65E-24	postive
PJVK	AC018695.4	0.511082955	7.79E-33	postive
SCAF11	AC018695.4	0.622428488	4.44E-52	postive
PJVK	AL035071.1	0.450389952	5.26E-25	postive
GSDME	HMGA2-AS1	0.635852063	6.00E-55	postive
IL1A	HMGA2-AS1	0.499890378	2.85E-31	postive
IL1B	HMGA2-AS1	0.567170321	1.29E-41	postive
NLRC4	HMGA2-AS1	0.540719373	2.87E-37	postive
NLRP3	HMGA2-AS1	0.469090983	2.97E-27	postive
SCAF11	HMGA2-AS1	0.668749193	1.28E-62	postive
CHMP4B	SNHG11	0.716689669	9.70E-76	postive
PLCG1	SNHG11	0.591997387	4.57E-46	postive
SCAF11	UBE2R2-AS1	0.497936633	5.28E-31	postive
CHMP2A	USP3-AS1	-0.459534784	4.35E-26	negative
CHMP6	USP3-AS1	-0.428471185	1.53E-22	negative
GSDME	USP3-AS1	0.525018563	7.27E-35	postive
IL1A	USP3-AS1	0.429374079	1.22E-22	postive
IL1B	USP3-AS1	0.478242128	2.10E-28	postive
NLRC4	USP3-AS1	0.470162379	2.19E-27	postive
SCAF11	USP3-AS1	0.73061799	4.46E-80	postive
GSDME	TSC22D1-AS1	0.4085674	1.86E-20	postive
NOD1	TSC22D1-AS1	0.541287841	2.34E-37	postive
PJVK	TSC22D1-AS1	0.450691925	4.85E-25	postive
SCAF11	TSC22D1-AS1	0.461433667	2.57E-26	postive

Pyroptosis-related Genes	lncRNA	cor	pvalue	Regulation
GSDME	DIAPH2-AS1	0.487903679	1.17E-29	postive
NLRC4	DIAPH2-AS1	0.413264956	6.17E-21	postive
PJVK	DIAPH2-AS1	0.434865465	3.05E-23	postive
SCAF11	DIAPH2-AS1	0.677850114	6.46E-65	postive
GSDME	LINC01762	0.447182458	1.24E-24	postive
NLRC4	LINC01762	0.417426544	2.28E-21	postive
SCAF11	LINC01762	0.512131336	5.52E-33	postive
GSDME	LINC02762	0.481752452	7.42E-29	postive
PJVK	LINC02762	0.423155427	5.69E-22	postive
SCAF11	LINC02762	0.482823483	5.39E-29	postive
GSDME	AC114763.1	0.513867473	3.11E-33	postive
IL1B	AC114763.1	0.412121569	8.09E-21	postive
NLRC4	AC114763.1	0.457832118	6.96E-26	postive
PJVK	AC114763.1	0.408953901	1.70E-20	postive
SCAF11	AC114763.1	0.604281146	2.04E-48	postive
GSDME	AL512506.1	0.501152933	1.91E-31	postive
NLRC4	AL512506.1	0.413112222	6.40E-21	postive
NOD1	AL512506.1	0.454322929	1.82E-25	postive
PJVK	AL512506.1	0.424611382	3.98E-22	postive
SCAF11	AL512506.1	0.640790616	4.86E-56	postive
CHMP2A	AL138921.2	-0.402503708	7.56E-20	negative
GSDME	AL138921.2	0.435744219	2.43E-23	postive
NLRC4	AL138921.2	0.440375076	7.39E-24	postive
PJVK	AL138921.2	0.478095929	2.19E-28	postive
SCAF11	AL138921.2	0.657799014	5.87E-60	postive
CHMP2A	AL513550.1	-0.408769317	1.78E-20	negative
GSDME	AL513550.1	0.45658105	9.81E-26	postive
NLRC4	AL513550.1	0.403907399	5.48E-20	postive
SCAF11	AL513550.1	0.530377176	1.14E-35	postive
NOD1	AC073957.3	0.60866589	2.80E-49	postive
PJVK	AC073957.3	0.443768065	3.05E-24	postive
SCAF11	AL450344.3	0.522349254	1.81E-34	postive
CHMP2A	AC010226.1	-0.409328134	1.56E-20	negative
GSDME	AC010226.1	0.513084423	4.03E-33	postive
NLRC4	AC010226.1	0.492432326	2.92E-30	postive
PJVK	AC010226.1	0.443745963	3.06E-24	postive
SCAF11	AC010226.1	0.760665352	1.98E-90	postive
CHMP2A	AC018926.3	-0.412027498	8.27E-21	negative

Pyroptosis-related Genes	lncRNA	cor	pvalue	Regulation
GSDME	AC018926.3	0.528781529	1.98E-35	postive
NLRC4	AC018926.3	0.429162939	1.29E-22	postive
PJVK	AC018926.3	0.40439886	4.90E-20	postive
SCAF11	AC018926.3	0.684435883	1.24E-66	postive
GSDME	AC005062.1	0.476668531	3.33E-28	postive
NOD1	AC005062.1	0.460586004	3.25E-26	postive
SCAF11	AC005062.1	0.618699387	2.63E-51	postive
GSDME	ADAMTS9-AS1	0.615544438	1.16E-50	postive
IL1A	ADAMTS9-AS1	0.461895353	2.26E-26	postive
IL1B	ADAMTS9-AS1	0.464723917	1.02E-26	postive
NLRC4	ADAMTS9-AS1	0.495137121	1.27E-30	postive
NLRP3	ADAMTS9-AS1	0.46550559	8.22E-27	postive
PJVK	ADAMTS9-AS1	0.427313439	2.04E-22	postive
SCAF11	ADAMTS9-AS1	0.673267648	9.50E-64	postive
GSDME	CNOT10-AS1	0.478442635	1.98E-28	postive
NLRC4	CNOT10-AS1	0.417884693	2.05E-21	postive
PJVK	CNOT10-AS1	0.464893194	9.76E-27	postive
SCAF11	CNOT10-AS1	0.69292832	6.50E-69	postive
GSDME	AC019118.1	0.419425271	1.41E-21	postive
SCAF11	AC019118.1	0.594874045	1.31E-46	postive
GSDME	RNF216-IT1	0.509767121	1.20E-32	postive
NLRC4	RNF216-IT1	0.415207093	3.89E-21	postive
NOD1	RNF216-IT1	0.457990773	6.66E-26	postive
SCAF11	RNF216-IT1	0.60633164	8.10E-49	postive
NLRC4	PDE2A-AS2	0.415187928	3.91E-21	postive
NLRP1	PDE2A-AS2	0.424789259	3.81E-22	postive
GSDME	AL157400.4	0.458954936	5.11E-26	postive
NOD1	AL157400.4	0.501255221	1.85E-31	postive
PJVK	AL157400.4	0.496739772	7.68E-31	postive
SCAF11	AL157400.4	0.58674774	4.30E-45	postive
SCAF11	AC012317.1	0.414879346	4.20E-21	postive
CHMP2A	AL358115.1	-0.438250115	1.28E-23	negative
CHMP6	AL358115.1	-0.428376573	1.56E-22	negative
GSDME	AL358115.1	0.538627814	6.11E-37	postive
IL1A	AL358115.1	0.408273974	2.00E-20	postive
IL1B	AL358115.1	0.446657005	1.42E-24	postive
NLRC4	AL358115.1	0.446876758	1.34E-24	postive
PJVK	AL358115.1	0.414213769	4.93E-21	postive

Pyroptosis-related Genes	lncRNA	cor	pvalue	Regulation
SCAF11	AL358115.1	0.669711921	7.41E-63	postive
PJVK	MCPH1-AS1	0.413114063	6.40E-21	postive
SCAF11	MCPH1-AS1	0.521431223	2.47E-34	postive
NOD1	AL162595.1	0.479381811	1.50E-28	postive
PJVK	AL162595.1	0.413225747	6.23E-21	postive
SCAF11	AL162595.1	0.423777275	4.88E-22	postive
PLCG1	ATXN1-AS1	0.456581891	9.81E-26	postive
CHMP2A	AC010168.1	-0.40050312	1.19E-19	negative
GSDME	AC010168.1	0.480016276	1.24E-28	postive
NLRC4	AC010168.1	0.439297759	9.76E-24	postive
SCAF11	AC010168.1	0.659764409	1.99E-60	postive
NOD1	ZNF460-AS1	0.43071545	8.71E-23	postive
PJVK	ZNF460-AS1	0.498500438	4.42E-31	postive
SCAF11	ZNF460-AS1	0.478410803	2.00E-28	postive
CHMP2A	AC012464.2	-0.410186296	1.28E-20	negative
GSDME	AC012464.2	0.469600564	2.57E-27	postive
NLRC4	AC012464.2	0.430576113	9.02E-23	postive
SCAF11	AC012464.2	0.623859688	2.23E-52	postive
CHMP2A	AC024075.1	-0.413584353	5.72E-21	negative
GSDME	AC024075.1	0.426218594	2.67E-22	postive
NLRC4	AC024075.1	0.417062056	2.49E-21	postive
NOD1	AC024075.1	0.406312502	3.15E-20	postive
PJVK	AC024075.1	0.500181746	2.60E-31	postive
SCAF11	AC024075.1	0.621016837	8.72E-52	postive
PJVK	AC107375.1	0.432587092	5.43E-23	postive
PLCG1	AC107375.1	0.406816549	2.80E-20	postive
NLRP1	WASIR2	0.433888983	3.91E-23	postive
SCAF11	LRP4-AS1	0.475026636	5.37E-28	postive
GSDME	A2M-AS1	0.662486671	4.40E-61	postive
IL1B	A2M-AS1	0.416509008	2.85E-21	postive
NLRC4	A2M-AS1	0.553987271	2.12E-39	postive
NLRP3	A2M-AS1	0.482260896	6.38E-29	postive
PJVK	A2M-AS1	0.437987374	1.37E-23	postive
SCAF11	A2M-AS1	0.594955131	1.27E-46	postive
GSDME	AC013468.1	0.41022629	1.26E-20	postive
PJVK	AC013468.1	0.518592803	6.44E-34	postive
SCAF11	AC013468.1	0.537729053	8.43E-37	postive
PJVK	AC009120.2	0.608314634	3.29E-49	postive

Pyroptosis-related Genes	lncRNA	cor	pvalue	Regulation
SCAF11	AC009120.2	0.547225452	2.66E-38	postive
CHMP2A	AC131971.1	-0.432669209	5.32E-23	negative
GSDME	AC131971.1	0.523418766	1.26E-34	postive
IL1A	AC131971.1	0.406393601	3.09E-20	postive
IL1B	AC131971.1	0.403554667	5.94E-20	postive
NLRC4	AC131971.1	0.473288705	8.89E-28	postive
PJVK	AC131971.1	0.410856171	1.09E-20	postive
SCAF11	AC131971.1	0.75306234	1.14E-87	postive
PJVK	ZNF197-AS1	0.436928912	1.80E-23	postive
SCAF11	ZNF197-AS1	0.525186943	6.86E-35	postive
CHMP2A	AL021707.7	-0.409516618	1.49E-20	negative
GSDME	AL021707.7	0.445629109	1.87E-24	postive
NLRC4	AL021707.7	0.424406604	4.18E-22	postive
NOD1	AL021707.7	0.438679145	1.15E-23	postive
SCAF11	AL021707.7	0.58053284	5.80E-44	postive
PLCG1	AC254629.1	0.412383474	7.60E-21	postive
NOD1	AC009404.1	0.403545302	5.96E-20	postive
PJVK	AC009404.1	0.509325487	1.38E-32	postive
SCAF11	AC009404.1	0.495195538	1.24E-30	postive
SCAF11	AC010973.1	0.487620818	1.27E-29	postive
PJVK	MAST4-AS1	0.410601458	1.16E-20	postive
SCAF11	MAST4-AS1	0.400838161	1.11E-19	postive
GSDME	AC006042.3	0.588528175	2.02E-45	postive
IL1A	AC006042.3	0.433530147	4.28E-23	postive
IL1B	AC006042.3	0.479587009	1.41E-28	postive
NLRC4	AC006042.3	0.495227256	1.23E-30	postive
SCAF11	AC006042.3	0.611637405	7.15E-50	postive
PJVK	GRM8-AS1	0.519508931	4.73E-34	postive
SCAF11	GRM8-AS1	0.49969658	3.03E-31	postive
GSDME	AC084876.1	0.543517318	1.04E-37	postive
NLRC4	AC084876.1	0.482995016	5.13E-29	postive
PJVK	AC084876.1	0.42868127	1.45E-22	postive
SCAF11	AC084876.1	0.663634447	2.32E-61	postive
GSDME	SYNJ2-IT1	0.452851347	2.71E-25	postive
IL1B	SYNJ2-IT1	0.404809397	4.45E-20	postive
NLRP3	SYNJ2-IT1	0.429487188	1.19E-22	postive
SCAF11	SYNJ2-IT1	0.58011301	6.90E-44	postive
CHMP2A	Z98884.2	-0.432810911	5.13E-23	negative

Pyroptosis-related Genes	lncRNA	cor	pvalue	Regulation
CHMP6	Z98884.2	-0.406911102	2.74E-20	negative
GSDME	Z98884.2	0.442628169	4.11E-24	postive
NLRC4	Z98884.2	0.433348967	4.48E-23	postive
SCAF11	Z98884.2	0.692154363	1.06E-68	postive
PJVK	AC005534.1	0.452213358	3.22E-25	postive
SCAF11	AC005534.1	0.416494183	2.86E-21	postive
HMGB1	AL391121.1	-0.440986195	6.30E-24	negative
PLCG1	LINC02418	0.411148184	1.02E-20	postive
PJVK	MIR3936HG	0.519880158	4.18E-34	postive
CHMP2A	MRTFA-AS1	-0.405021129	4.24E-20	negative
GSDME	MRTFA-AS1	0.591074621	6.79E-46	postive
IL1A	MRTFA-AS1	0.498971028	3.81E-31	postive
IL1B	MRTFA-AS1	0.558136573	4.37E-40	postive
NLRC4	MRTFA-AS1	0.501197787	1.89E-31	postive
NLRP3	MRTFA-AS1	0.46145462	2.56E-26	postive
SCAF11	MRTFA-AS1	0.667983559	1.99E-62	postive
SCAF11	AL121782.1	0.543185705	1.17E-37	postive
GSDME	AL021392.1	0.451937737	3.47E-25	postive
NLRP1	AC246787.2	0.446557735	1.46E-24	postive
PJVK	AC008114.1	0.460163444	3.66E-26	postive
SCAF11	AC008114.1	0.66365189	2.30E-61	postive
GSDME	AC112496.1	0.613065201	3.69E-50	postive
IL1A	AC112496.1	0.576722109	2.78E-43	postive
IL1B	AC112496.1	0.695135989	1.61E-69	postive
NLRC4	AC112496.1	0.529856495	1.36E-35	postive
NLRP3	AC112496.1	0.546812564	3.10E-38	postive
SCAF11	AC112496.1	0.613293504	3.32E-50	postive
CHMP2A	AC096992.2	-0.425368282	3.30E-22	negative
GSDME	AC096992.2	0.412094923	8.14E-21	postive
PJVK	AC096992.2	0.557246298	6.14E-40	postive
SCAF11	AC096992.2	0.608612438	2.87E-49	postive
PJVK	AC073326.1	0.487809225	1.20E-29	postive
SCAF11	AC073326.1	0.616065215	9.09E-51	postive
GSDME	LINC01655	0.621232899	7.87E-52	postive
IL1A	LINC01655	0.4074812	2.40E-20	postive
IL1B	LINC01655	0.466765705	5.75E-27	postive
NLRC4	LINC01655	0.513076079	4.04E-33	postive
NLRP3	LINC01655	0.401219177	1.01E-19	postive

Pyroptosis-related Genes	lncRNA	cor	pvalue	Regulation
SCAF11	LINC01655	0.534325816	2.83E-36	postive
PLCG1	AC040162.3	0.421406434	8.72E-22	postive
GSDME	GAS5-AS1	0.535477426	1.88E-36	postive
NLRC4	GAS5-AS1	0.4606563	3.19E-26	postive
NOD1	GAS5-AS1	0.404155129	5.18E-20	postive
PJVK	GAS5-AS1	0.449759121	6.22E-25	postive
SCAF11	GAS5-AS1	0.707538026	4.98E-73	postive
CHMP3	AC115618.2	0.448182211	9.48E-25	postive
SCAF11	AC115618.2	0.400137388	1.30E-19	postive
CHMP2A	AP000873.2	-0.454606959	1.68E-25	negative
CHMP6	AP000873.2	-0.404470064	4.82E-20	negative
GSDME	AP000873.2	0.466833882	5.64E-27	postive
CASP8	AP000873.2	0.40429784	5.01E-20	postive
NLRC4	AP000873.2	0.405225455	4.05E-20	postive
PJVK	AP000873.2	0.490402032	5.46E-30	postive
SCAF11	AP000873.2	0.670789808	3.99E-63	postive
PJVK	AC005785.1	0.427411239	1.99E-22	postive
SCAF11	AC005785.1	0.405768718	3.57E-20	postive
CHMP4A	AL136295.6	0.499222059	3.52E-31	postive
CHMP2A	EP300-AS1	-0.413481631	5.86E-21	negative
GSDME	EP300-AS1	0.422590735	6.53E-22	postive
IL1A	EP300-AS1	0.416201006	3.07E-21	postive
IL1B	EP300-AS1	0.416220437	3.05E-21	postive
SCAF11	EP300-AS1	0.610603599	1.15E-49	postive
SCAF11	AC027277.2	0.491181644	4.30E-30	postive
SCAF11	AC004691.1	0.423521887	5.20E-22	postive
CHMP2A	YEATS2-AS1	-0.42860759	1.48E-22	negative
CHMP6	YEATS2-AS1	-0.406084281	3.32E-20	negative
GSDME	YEATS2-AS1	0.502093613	1.42E-31	postive
NLRC4	YEATS2-AS1	0.45457128	1.70E-25	postive
NOD1	YEATS2-AS1	0.464930998	9.66E-27	postive
PJVK	YEATS2-AS1	0.457835577	6.96E-26	postive
SCAF11	YEATS2-AS1	0.644389463	7.56E-57	postive
GSDME	GAS8-AS1	0.423819861	4.83E-22	postive
PJVK	GAS8-AS1	0.501284553	1.84E-31	postive
SCAF11	GAS8-AS1	0.484518434	3.25E-29	postive
GSDME	AL158163.2	0.438522072	1.19E-23	postive
PJVK	AL158163.2	0.518510225	6.62E-34	postive

Pyroptosis-related Genes	lncRNA	cor	pvalue	Regulation
SCAF11	AL158163.2	0.607019916	5.93E-49	postive
GSDME	AC096708.2	0.567003734	1.38E-41	postive
IL1A	AC096708.2	0.454123006	1.92E-25	postive
IL1B	AC096708.2	0.521813865	2.17E-34	postive
NLRC4	AC096708.2	0.526851642	3.87E-35	postive
PJVK	AC096708.2	0.40005207	1.32E-19	postive
SCAF11	AC096708.2	0.632173786	3.79E-54	postive
GSDME	AC131934.1	0.542298026	1.62E-37	postive
IL1A	AC131934.1	0.454096328	1.93E-25	postive
IL1B	AC131934.1	0.484513844	3.25E-29	postive
NLRC4	AC131934.1	0.446423111	1.51E-24	postive
NLRP3	AC131934.1	0.437681305	1.48E-23	postive
SCAF11	AC131934.1	0.625087477	1.23E-52	postive
CHMP2A	AL359922.2	-0.438533127	1.19E-23	negative
CHMP6	AL359922.2	-0.404584798	4.69E-20	negative
GSDME	AL359922.2	0.498636163	4.24E-31	postive
NLRC4	AL359922.2	0.452536364	2.95E-25	postive
PJVK	AL359922.2	0.434677497	3.20E-23	postive
SCAF11	AL359922.2	0.701427953	2.81E-71	postive
CHMP2A	AL596325.2	-0.422848983	6.13E-22	negative
CHMP6	AL596325.2	-0.412851158	6.81E-21	negative
GSDME	AL596325.2	0.573545999	1.01E-42	postive
IL1A	AL596325.2	0.536399505	1.36E-36	postive
IL1B	AL596325.2	0.619248117	2.02E-51	postive
NLRC4	AL596325.2	0.50860687	1.75E-32	postive
NLRP3	AL596325.2	0.523455223	1.24E-34	postive
SCAF11	AL596325.2	0.697688971	3.16E-70	postive
GSDME	JMJD1C-AS1	0.461825603	2.31E-26	postive
SCAF11	JMJD1C-AS1	0.474368532	6.50E-28	postive
GSDME	AC011468.1	0.427873871	1.77E-22	postive
SCAF11	AC011468.1	0.587037419	3.80E-45	postive
PJVK	C9orf147	0.548841176	1.46E-38	postive
SCAF11	C9orf147	0.553696046	2.37E-39	postive
PJVK	TRMT2B-AS1	0.508953479	1.56E-32	postive
GSDME	AC011773.1	0.408890153	1.73E-20	postive
PJVK	AC011773.1	0.453692046	2.16E-25	postive
SCAF11	AC011773.1	0.536766576	1.19E-36	postive
NOD1	SAMD12-AS1	0.402517446	7.54E-20	postive

Pyroptosis-related Genes	lncRNA	cor	pvalue	Regulation
PJVK	SAMD12-AS1	0.435211219	2.79E-23	postive
SCAF11	SAMD12-AS1	0.550457257	8.00E-39	postive
CHMP2A	Z98885.3	-0.437212922	1.67E-23	negative
GSDME	Z98885.3	0.471367322	1.55E-27	postive
NLRC4	Z98885.3	0.414015336	5.16E-21	postive
PJVK	Z98885.3	0.446690784	1.41E-24	postive
SCAF11	Z98885.3	0.689137575	6.93E-68	postive
GSDME	L3MBTL4-AS1	0.609202919	2.19E-49	postive
IL1A	L3MBTL4-AS1	0.406705276	2.87E-20	postive
IL1B	L3MBTL4-AS1	0.412293566	7.77E-21	postive
NLRC4	L3MBTL4-AS1	0.543905339	9.02E-38	postive
NLRP3	L3MBTL4-AS1	0.492557778	2.81E-30	postive
SCAF11	L3MBTL4-AS1	0.624983103	1.29E-52	postive
SCAF11	AC021739.3	0.428890438	1.38E-22	postive
CHMP4B	AL117382.2	0.411055898	1.04E-20	postive
HMGB1	AL117382.2	0.442949201	3.78E-24	postive
NOD1	AL117382.2	0.423221136	5.60E-22	postive
PLCG1	AL117382.2	0.551631749	5.15E-39	postive
SCAF11	AC023825.2	0.401262433	1.00E-19	postive
CHMP2A	EPN2-AS1	-0.401451591	9.61E-20	negative
GSDME	EPN2-AS1	0.540443309	3.17E-37	postive
IL1A	EPN2-AS1	0.418212485	1.89E-21	postive
IL1B	EPN2-AS1	0.407897087	2.18E-20	postive
NLRC4	EPN2-AS1	0.428877838	1.38E-22	postive
NLRP3	EPN2-AS1	0.450434837	5.19E-25	postive
NOD1	EPN2-AS1	0.409038093	1.67E-20	postive
SCAF11	EPN2-AS1	0.632294078	3.57E-54	postive
HMGB1	AL137058.2	0.560052114	2.09E-40	postive
SCAF11	MYB-AS1	0.662895342	3.51E-61	postive
GSDME	AC112721.1	0.618499208	2.89E-51	postive
IL1A	AC112721.1	0.441742711	5.17E-24	postive
IL1B	AC112721.1	0.499196792	3.55E-31	postive
NLRC4	AC112721.1	0.459874778	3.96E-26	postive
NLRP3	AC112721.1	0.720263417	7.92E-77	postive
SCAF11	AC112721.1	0.422376664	6.88E-22	postive
GSDME	MAL2-AS1	0.511259118	7.35E-33	postive
IL1A	MAL2-AS1	0.419845828	1.27E-21	postive
IL1B	MAL2-AS1	0.485245862	2.61E-29	postive

Pyroptosis-related Genes	lncRNA	cor	pvalue	Regulation
NLRC4	MAL2-AS1	0.474483834	6.29E-28	postive
NLRP3	MAL2-AS1	0.416399897	2.92E-21	postive
SCAF11	MAL2-AS1	0.637901366	2.13E-55	postive
CHMP2A	TBL1XR1-AS1	-0.402749151	7.15E-20	negative
GSDME	TBL1XR1-AS1	0.567780202	1.02E-41	postive
IL1A	TBL1XR1-AS1	0.413378678	6.01E-21	postive
IL1B	TBL1XR1-AS1	0.451748969	3.65E-25	postive
NLRC4	TBL1XR1-AS1	0.51867144	6.27E-34	postive
SCAF11	TBL1XR1-AS1	0.685593043	6.13E-67	postive
GSDME	AC123768.2	0.402950156	6.83E-20	postive
SCAF11	AC123768.2	0.525194403	6.84E-35	postive
CHMP2A	Z82217.1	-0.428190839	1.64E-22	negative
CHMP6	Z82217.1	-0.402349947	7.83E-20	negative
GSDME	Z82217.1	0.532708902	5.01E-36	postive
IL1A	Z82217.1	0.446990184	1.30E-24	postive
IL1B	Z82217.1	0.443489863	3.28E-24	postive
NLRC4	Z82217.1	0.436667325	1.92E-23	postive
NLRP3	Z82217.1	0.424147406	4.46E-22	postive
SCAF11	Z82217.1	0.697021879	4.84E-70	postive
IL1B	AL606834.1	0.433959788	3.84E-23	postive
NLRP3	AL606834.1	0.453682262	2.16E-25	postive
SCAF11	AL606834.1	0.462167014	2.10E-26	postive
GSDME	AL158212.3	0.434681553	3.19E-23	postive
NOD1	AL158212.3	0.478893092	1.73E-28	postive
PJVK	AL158212.3	0.469282401	2.81E-27	postive
SCAF11	AL158212.3	0.567483076	1.14E-41	postive
CHMP4A	AC046143.2	0.43259722	5.42E-23	postive
GSDME	KANSL1L-AS1	0.502846118	1.12E-31	postive
NLRC4	KANSL1L-AS1	0.41968836	1.32E-21	postive
NOD1	KANSL1L-AS1	0.429335874	1.23E-22	postive
PJVK	KANSL1L-AS1	0.496589734	8.05E-31	postive
SCAF11	KANSL1L-AS1	0.646687397	2.27E-57	postive
GSDME	IGBP1-AS2	0.410705202	1.13E-20	postive
SCAF11	IGBP1-AS2	0.542176358	1.69E-37	postive
GSDME	AC010149.1	0.45199297	3.41E-25	postive
PJVK	AC010149.1	0.462406193	1.96E-26	postive
SCAF11	AC010149.1	0.551326366	5.78E-39	postive
GSDME	ITPK1-AS1	0.469809568	2.42E-27	postive

Pyroptosis-related Genes	lncRNA	cor	pvalue	Regulation
NLRC4	ITPK1-AS1	0.404329856	4.97E-20	postive
SCAF11	ITPK1-AS1	0.608159743	3.53E-49	postive
CHMP2A	AC007823.1	-0.409849186	1.38E-20	negative
GSDME	AC007823.1	0.402880254	6.94E-20	postive
NLRC4	AC007823.1	0.431922381	6.43E-23	postive
PJVK	AC007823.1	0.544983216	6.08E-38	postive
SCAF11	AC007823.1	0.674674737	4.18E-64	postive
SCAF11	AL021707.1	0.41604519	3.18E-21	postive
SCAF11	SEMA6A-AS1	0.665398997	8.60E-62	postive
CHMP2A	AL122035.2	-0.438008495	1.36E-23	negative
CHMP6	AL122035.2	-0.410659858	1.14E-20	negative
GSDME	AL122035.2	0.55361343	2.44E-39	postive
IL1B	AL122035.2	0.427195976	2.10E-22	postive
NLRC4	AL122035.2	0.508146357	2.03E-32	postive
PJVK	AL122035.2	0.429228847	1.26E-22	postive
SCAF11	AL122035.2	0.679544475	2.36E-65	postive
GSDME	AL354892.3	0.453929422	2.02E-25	postive
NLRC4	AL354892.3	0.4297004	1.12E-22	postive
NOD1	AL354892.3	0.4030755	6.63E-20	postive
PJVK	AL354892.3	0.464062537	1.23E-26	postive
SCAF11	AL354892.3	0.610362929	1.29E-49	postive
GSDME	DLEU1	0.52505238	7.18E-35	postive
NLRC4	DLEU1	0.453017257	2.59E-25	postive
NOD1	DLEU1	0.455329258	1.38E-25	postive
PJVK	DLEU1	0.42160435	8.31E-22	postive
SCAF11	DLEU1	0.676384064	1.53E-64	postive
IRF1	AC007991.4	0.435207381	2.79E-23	postive
NLRC4	AC007991.4	0.408245757	2.01E-20	postive
SCAF11	AC007991.4	0.441391688	5.67E-24	postive
NOD1	AL121583.1	0.521214781	2.66E-34	postive
PJVK	AL121583.1	0.468726044	3.30E-27	postive
SCAF11	AL121583.1	0.454738811	1.62E-25	postive
PJVK	AL031673.1	0.494049455	1.77E-30	postive
SCAF11	AL031673.1	0.475159079	5.17E-28	postive
PJVK	AC008280.2	0.44710848	1.26E-24	postive
SCAF11	AC008280.2	0.431440641	7.26E-23	postive
GSDME	AC107959.1	0.569337414	5.48E-42	postive
NLRC4	AC107959.1	0.542414833	1.55E-37	postive

Pyroptosis-related Genes	lncRNA	cor	pvalue	Regulation
NLRP1	AC107959.1	0.471677806	1.42E-27	postive
NLRP3	AC107959.1	0.589008088	1.65E-45	postive
SCAF11	AC067747.1	0.523268745	1.32E-34	postive
GSDME	SNHG22	0.41174976	8.83E-21	postive
SCAF11	SNHG22	0.446869315	1.34E-24	postive
PJVK	AC091887.1	0.458850317	5.26E-26	postive
SCAF11	AC091887.1	0.513982631	3.00E-33	postive
GSDME	CLMAT3	0.442739497	3.99E-24	postive
IL1A	CLMAT3	0.416404802	2.92E-21	postive
IL1B	CLMAT3	0.49631801	8.76E-31	postive
NLRP3	CLMAT3	0.462400428	1.96E-26	postive
GSDME	AC021205.3	0.472290255	1.19E-27	postive
IL1B	AC021205.3	0.424934473	3.67E-22	postive
NLRC4	AC021205.3	0.501416429	1.76E-31	postive
PJVK	AC021205.3	0.420414272	1.11E-21	postive
SCAF11	AC021205.3	0.547472868	2.43E-38	postive
GSDME	LINC02544	0.519416667	4.88E-34	postive
NLRC4	LINC02544	0.410439508	1.20E-20	postive
PJVK	LINC01252	0.401275069	1.00E-19	postive
PJVK	AC087277.2	0.490217493	5.77E-30	postive
SCAF11	AC087277.2	0.540493573	3.12E-37	postive
SCAF11	LINC02244	0.466806878	5.69E-27	postive
NLRC4	LINC01857	0.470040729	2.26E-27	postive
NLRP1	LINC01857	0.504118069	7.44E-32	postive
GSDME	MBNL1-AS1	0.466185106	6.78E-27	postive
SCAF11	MBNL1-AS1	0.438242454	1.28E-23	postive
PJVK	BDNF-AS	0.410333936	1.23E-20	postive
CHMP2A	AL133243.2	-0.406104548	3.30E-20	negative
CHMP6	AL133243.2	-0.40751186	2.38E-20	negative
GSDME	AL133243.2	0.56262775	7.70E-41	postive
IL1A	AL133243.2	0.419452412	1.40E-21	postive
IL1B	AL133243.2	0.41143318	9.52E-21	postive
NLRC4	AL133243.2	0.463921699	1.28E-26	postive
PJVK	AL133243.2	0.438008667	1.36E-23	postive
SCAF11	AL133243.2	0.737544296	2.45E-82	postive
GPX4	AL138724.2	0.426349344	2.59E-22	postive
CHMP2A	AC015914.1	-0.406823073	2.80E-20	negative
GSDME	AC015914.1	0.522228403	1.88E-34	postive

Pyroptosis-related Genes	lncRNA	cor	pvalue	Regulation
IL1A	AC015914.1	0.401730078	9.02E-20	postive
IL1B	AC015914.1	0.400010494	1.33E-19	postive
NLRC4	AC015914.1	0.401642905	9.20E-20	postive
SCAF11	AC015914.1	0.643779454	1.04E-56	postive
CHMP2A	AC005070.3	-0.415589893	3.55E-21	negative
CHMP6	AC005070.3	-0.401390781	9.75E-20	negative
GSDME	AC005070.3	0.544739734	6.64E-38	postive
IL1A	AC005070.3	0.408725388	1.80E-20	postive
IL1B	AC005070.3	0.414358267	4.76E-21	postive
NLRC4	AC005070.3	0.485877035	2.16E-29	postive
PJVK	AC005070.3	0.425136896	3.49E-22	postive
SCAF11	AC005070.3	0.744807958	8.73E-85	postive
CHMP4C	SNHG6	0.500200562	2.59E-31	postive
CHMP2A	AP002907.1	-0.429998923	1.04E-22	negative
CHMP6	AP002907.1	-0.401365198	9.81E-20	negative
GSDME	AP002907.1	0.544809263	6.48E-38	postive
IL1A	AP002907.1	0.43648836	2.01E-23	postive
IL1B	AP002907.1	0.457486924	7.65E-26	postive
NLRC4	AP002907.1	0.467690553	4.43E-27	postive
PJVK	AP002907.1	0.475546673	4.62E-28	postive
SCAF11	AP002907.1	0.699392635	1.05E-70	postive
GSDME	AL360219.2	0.54354681	1.03E-37	postive
IL1A	AL360219.2	0.507929018	2.18E-32	postive
IL1B	AL360219.2	0.552752959	3.38E-39	postive
NLRC4	AL360219.2	0.436639522	1.94E-23	postive
NLRP3	AL360219.2	0.479196299	1.58E-28	postive
PJVK	AL360219.2	0.424888849	3.71E-22	postive
SCAF11	AL360219.2	0.607638348	4.47E-49	postive
IL1B	ERVMER61-1	0.513358953	3.68E-33	postive
NLRC4	ERVMER61-1	0.408044577	2.10E-20	postive
NLRC4	AL592114.3	0.445230403	2.07E-24	postive
NOD1	AL592114.3	0.43483658	3.07E-23	postive
SCAF11	AL592114.3	0.501190573	1.89E-31	postive
GSDME	Z93022.1	0.41167084	9.00E-21	postive
SCAF11	Z93022.1	0.47760325	2.53E-28	postive
CHMP3	AL121603.2	0.454804108	1.59E-25	postive
CHMP2A	ZNF346-IT1	-0.434406132	3.42E-23	negative
CHMP6	ZNF346-IT1	-0.401779221	8.92E-20	negative

Pyroptosis-related Genes	lncRNA	cor	pvalue	Regulation
GSDME	ZNF346-IT1	0.498871436	3.94E-31	postive
IL1B	ZNF346-IT1	0.410469895	1.19E-20	postive
NLRC4	ZNF346-IT1	0.415299255	3.80E-21	postive
PJVK	ZNF346-IT1	0.424681697	3.91E-22	postive
SCAF11	ZNF346-IT1	0.698948926	1.40E-70	postive
SCAF11	AL158212.2	0.452809572	2.74E-25	postive
GSDME	AC079336.2	0.420438767	1.10E-21	postive
PJVK	AC079336.2	0.400534956	1.18E-19	postive
SCAF11	AC079336.2	0.596977138	5.24E-47	postive
GSDME	AC036108.3	0.553931614	2.17E-39	postive
PJVK	AC036108.3	0.420514444	1.08E-21	postive
SCAF11	AC036108.3	0.563140513	6.30E-41	postive
NOD1	AL357079.1	0.406053235	3.34E-20	postive
PJVK	AL357079.1	0.471833636	1.35E-27	postive
PLCG1	AFDN-DT	0.471713005	1.40E-27	postive
GSDME	LINC00571	0.452203843	3.23E-25	postive
NOD1	LINC00571	0.488707003	9.15E-30	postive
SCAF11	LINC00571	0.65386024	5.00E-59	postive
CHMP2A	ASAP1-IT2	-0.404076885	5.27E-20	negative
GSDME	ASAP1-IT2	0.55621298	9.12E-40	postive
IL1A	ASAP1-IT2	0.427728134	1.84E-22	postive
IL1B	ASAP1-IT2	0.466077676	6.99E-27	postive
NLRC4	ASAP1-IT2	0.467900116	4.17E-27	postive
NLRP3	ASAP1-IT2	0.403657821	5.81E-20	postive
SCAF11	ASAP1-IT2	0.66263058	4.07E-61	postive
GSDME	ST3GAL6-AS1	0.588370604	2.16E-45	postive
NLRC4	ST3GAL6-AS1	0.46792206	4.15E-27	postive
GSDME	AL157838.1	0.55316849	2.89E-39	postive
NLRC4	AL157838.1	0.456342042	1.05E-25	postive
NOD1	AL157838.1	0.455163605	1.45E-25	postive
SCAF11	AL157838.1	0.634954272	9.43E-55	postive
PJVK	AC090527.4	0.436418962	2.05E-23	postive
GSDME	AC062037.3	0.436421467	2.05E-23	postive
IL1B	AC062037.3	0.410388919	1.22E-20	postive
NLRP3	AC062037.3	0.412464309	7.46E-21	postive
SCAF11	AC062037.3	0.567628608	1.08E-41	postive
GSDME	AC087392.2	0.473997145	7.24E-28	postive
IL1A	AC087392.2	0.512832729	4.38E-33	postive

Pyroptosis-related Genes	lncRNA	cor	pvalue	Regulation
IL1B	AC087392.2	0.577992363	1.65E-43	postive
NLRP3	AC087392.2	0.530368717	1.14E-35	postive
SCAF11	AC087392.2	0.471322263	1.57E-27	postive
CHMP2A	AL033397.2	-0.411879	8.57E-21	negative
GSDME	AL033397.2	0.474929279	5.53E-28	postive
IL1B	AL033397.2	0.402026538	8.43E-20	postive
NLRC4	AL033397.2	0.452823222	2.73E-25	postive
PJVK	AL033397.2	0.431327053	7.47E-23	postive
SCAF11	AL033397.2	0.718659652	2.45E-76	postive
CHMP6	AL683813.2	-0.407855926	2.20E-20	negative
GSDME	AL683813.2	0.447215125	1.23E-24	postive
NOD1	AL683813.2	0.431111639	7.88E-23	postive
PJVK	AL683813.2	0.545269882	5.47E-38	postive
SCAF11	AL683813.2	0.587244999	3.48E-45	postive
SCAF11	AC087588.2	0.658931819	3.15E-60	postive
GSDME	EGOT	0.569427057	5.28E-42	postive
NLRC4	EGOT	0.47029369	2.11E-27	postive
SCAF11	EGOT	0.661620302	7.13E-61	postive
PJVK	PIK3IP1-AS1	0.404731591	4.53E-20	postive
SCAF11	AC024451.4	0.489005521	8.36E-30	postive
CHMP2A	CCDC18-AS1	-0.408987304	1.69E-20	negative
PJVK	CCDC18-AS1	0.568631451	7.25E-42	postive
SCAF11	CCDC18-AS1	0.591136099	6.62E-46	postive
CHMP2A	AC004492.1	-0.423773921	4.89E-22	negative
CHMP6	AC004492.1	-0.417567436	2.21E-21	negative
GSDME	AC004492.1	0.541735203	1.99E-37	postive
IL1A	AC004492.1	0.402171414	8.16E-20	postive
IL1B	AC004492.1	0.421687843	8.14E-22	postive
NLRC4	AC004492.1	0.478093771	2.19E-28	postive
PJVK	AC004492.1	0.432856578	5.07E-23	postive
SCAF11	AC004492.1	0.740890457	1.87E-83	postive
BAX	EBLN3P	-0.43929607	9.77E-24	negative
CHMP2A	EBLN3P	-0.504035327	7.64E-32	negative
CHMP3	EBLN3P	0.438689275	1.14E-23	postive
CHMP6	EBLN3P	-0.479387224	1.50E-28	negative
CASP8	EBLN3P	0.471335262	1.56E-27	postive
GPX4	EBLN3P	-0.473385594	8.65E-28	negative
PJVK	EBLN3P	0.499935541	2.81E-31	postive

Pyroptosis-related Genes	lncRNA	cor	pvalue	Regulation
SCAF11	EBLN3P	0.711762393	2.88E-74	postive
PJVK	AC106798.1	0.481839692	7.23E-29	postive
PJVK	AC093227.1	0.546212562	3.87E-38	postive
SCAF11	AC093227.1	0.626797868	5.35E-53	postive
SCAF11	AC073529.1	0.606781495	6.60E-49	postive
CHMP2A	AC090519.2	-0.411878223	8.57E-21	negative
GSDME	AC090519.2	0.557019973	6.70E-40	postive
IL1A	AC090519.2	0.4076884	2.29E-20	postive
NLRC4	AC090519.2	0.447185821	1.24E-24	postive
SCAF11	AC090519.2	0.67365089	7.60E-64	postive
NOD1	AC008764.8	0.49117871	4.30E-30	postive
PJVK	AC008764.8	0.457962536	6.72E-26	postive
SCAF11	AC008764.8	0.415499417	3.63E-21	postive
CHMP2A	USP46-AS1	-0.405363304	3.92E-20	negative
CASP8	USP46-AS1	0.42609205	2.76E-22	postive
PJVK	USP46-AS1	0.461821455	2.31E-26	postive
SCAF11	USP46-AS1	0.53981105	3.99E-37	postive
PJVK	AC073352.1	0.473811788	7.64E-28	postive
NOD1	Z84485.1	0.436273432	2.13E-23	postive
PJVK	Z84485.1	0.476782313	3.22E-28	postive
PJVK	AL132657.1	0.481053329	9.14E-29	postive
GSDME	AC103923.1	0.429880522	1.07E-22	postive
NLRC4	AC103923.1	0.402756029	7.14E-20	postive
SCAF11	AC103923.1	0.684605529	1.12E-66	postive
CHMP2A	AC007390.1	-0.438215965	1.29E-23	negative
CHMP6	AC007390.1	-0.433503769	4.31E-23	negative
GSDME	AC007390.1	0.532615663	5.18E-36	postive
IL1A	AC007390.1	0.404714573	4.55E-20	postive
NLRC4	AC007390.1	0.460647389	3.20E-26	postive
PJVK	AC007390.1	0.433994007	3.80E-23	postive
SCAF11	AC007390.1	0.751385602	4.48E-87	postive
GSDME	ADAMTSL4-AS1	0.607670275	4.41E-49	postive
IL1A	ADAMTSL4-AS1	0.599751564	1.54E-47	postive
IL1B	ADAMTSL4-AS1	0.688619707	9.55E-68	postive
NLRC4	ADAMTSL4-AS1	0.497510542	6.03E-31	postive
NLRP3	ADAMTSL4-AS1	0.652306343	1.15E-58	postive
SCAF11	ADAMTSL4-AS1	0.606479621	7.57E-49	postive
GSDME	LINC00861	0.450258416	5.44E-25	postive

Pyroptosis-related Genes	lncRNA	cor	pvalue	Regulation
NLRC4	LINC00861	0.493979488	1.81E-30	postive
SCAF11	LINC00861	0.566403729	1.75E-41	postive
PJVK	EXTL3-AS1	0.404112163	5.23E-20	postive
GSDME	AC112721.2	0.656596609	1.13E-59	postive
IL1B	AC112721.2	0.485389294	2.50E-29	postive
NLRC4	AC112721.2	0.57860231	1.29E-43	postive
NLRP3	AC112721.2	0.639887391	7.72E-56	postive
SCAF11	AC112721.2	0.491042268	4.48E-30	postive
PJVK	AC009962.1	0.601492859	7.12E-48	postive
SCAF11	AC009962.1	0.54603255	4.13E-38	postive
CHMP2A	AC009054.2	-0.406035729	3.36E-20	negative
GSDME	AC009054.2	0.454298532	1.83E-25	postive
IL1B	AC009054.2	0.409260583	1.58E-20	postive
NLRC4	AC009054.2	0.402398479	7.75E-20	postive
SCAF11	AC009054.2	0.550883423	6.82E-39	postive
PYCARD	AC020765.2	0.427663101	1.87E-22	postive
GSDME	AC024940.6	0.432124357	6.11E-23	postive
NLRC4	AC024940.6	0.414158252	4.99E-21	postive
PJVK	AC024940.6	0.438170584	1.31E-23	postive
SCAF11	AC024940.6	0.740254184	3.06E-83	postive
GSDME	AC079915.1	0.538545526	6.29E-37	postive
NLRC4	AC079915.1	0.457023744	8.69E-26	postive
PJVK	AC079915.1	0.49073989	4.92E-30	postive
SCAF11	AC079915.1	0.693409929	4.80E-69	postive
NOD1	AZIN1-AS1	0.431435718	7.26E-23	postive
PJVK	AZIN1-AS1	0.400211085	1.27E-19	postive
CHMP3	OTUD6B-AS1	0.512876363	4.32E-33	postive
CHMP4C	OTUD6B-AS1	0.526147303	4.93E-35	postive
HMGB1	OTUD6B-AS1	0.475051832	5.33E-28	postive
PJVK	OTUD6B-AS1	0.424814281	3.78E-22	postive
SCAF11	OTUD6B-AS1	0.405935659	3.43E-20	postive
CHMP2A	AC090948.2	-0.432095592	6.15E-23	negative
GSDME	AC090948.2	0.495951805	9.82E-31	postive
IL1A	AC090948.2	0.425437967	3.24E-22	postive
NLRP3	AC090948.2	0.497749567	5.60E-31	postive
PJVK	AC090948.2	0.402853733	6.98E-20	postive
SCAF11	AC090948.2	0.632954991	2.57E-54	postive
GSDME	ARAP1-AS2	0.597471525	4.22E-47	postive

Pyroptosis-related Genes	lncRNA	cor	pvalue	Regulation
IL1A	ARAP1-AS2	0.509259625	1.41E-32	postive
IL1B	ARAP1-AS2	0.565590571	2.41E-41	postive
NLRC4	ARAP1-AS2	0.505040581	5.53E-32	postive
NLRP3	ARAP1-AS2	0.518365035	6.95E-34	postive
PJVK	ARAP1-AS2	0.415943274	3.26E-21	postive
SCAF11	ARAP1-AS2	0.658564542	3.86E-60	postive
CHMP2A	AL354989.1	-0.431996928	6.31E-23	negative
CHMP6	AL354989.1	-0.413018164	6.54E-21	negative
GSDME	AL354989.1	0.525225506	6.77E-35	postive
IL1B	AL354989.1	0.468169733	3.86E-27	postive
NLRC4	AL354989.1	0.526287325	4.70E-35	postive
PJVK	AL354989.1	0.432983598	4.91E-23	postive
SCAF11	AL354989.1	0.704663303	3.36E-72	postive
NLRP1	AC015819.1	0.447668292	1.09E-24	postive
CHMP2A	AC004943.1	-0.409193751	1.61E-20	negative
GSDME	AC004943.1	0.514922387	2.19E-33	postive
NLRC4	AC004943.1	0.428328267	1.58E-22	postive
NOD1	AC004943.1	0.419080599	1.53E-21	postive
PJVK	AC004943.1	0.455649356	1.27E-25	postive
SCAF11	AC004943.1	0.675387169	2.76E-64	postive
PJVK	AC005674.1	0.469517138	2.63E-27	postive
SCAF11	AC005674.1	0.562565783	7.89E-41	postive
CHMP2A	AC124283.3	-0.407928103	2.16E-20	negative
GSDME	AC124283.3	0.498641643	4.23E-31	postive
NLRC4	AC124283.3	0.424653884	3.94E-22	postive
PJVK	AC124283.3	0.502277584	1.34E-31	postive
SCAF11	AC124283.3	0.691541087	1.55E-68	postive
NOD1	AL353708.3	0.426323163	2.61E-22	postive
PJVK	AL353708.3	0.536670337	1.23E-36	postive
SCAF11	AL353708.3	0.506074331	3.97E-32	postive
PJVK	AC008124.1	0.505183817	5.28E-32	postive
SCAF11	AC008124.1	0.644313076	7.86E-57	postive
PJVK	AC015802.4	0.50643954	3.53E-32	postive
ELANE	ZNF667-AS1	0.44185802	5.02E-24	postive
GSDME	ZNF667-AS1	0.608503366	3.01E-49	postive
NLRC4	ZNF667-AS1	0.443749345	3.06E-24	postive
NLRP3	ZNF667-AS1	0.4526758	2.84E-25	postive
GSDME	AP000866.6	0.511118298	7.70E-33	postive

Pyroptosis-related Genes	lncRNA	cor	pvalue	Regulation
NLRC4	AP000866.6	0.446032186	1.68E-24	postive
PJVK	AP000866.6	0.482929374	5.23E-29	postive
SCAF11	AP000866.6	0.690408855	3.15E-68	postive
CHMP2A	AC134407.1	-0.428874852	1.38E-22	negative
GSDME	AC134407.1	0.547243977	2.64E-38	postive
NLRC4	AC134407.1	0.444037749	2.84E-24	postive
PJVK	AC134407.1	0.405486675	3.81E-20	postive
SCAF11	AC134407.1	0.681344064	8.04E-66	postive
GSDME	LINC00894	0.407169173	2.58E-20	postive
PJVK	LINC00894	0.420755511	1.02E-21	postive
SCAF11	LINC00894	0.598032735	3.30E-47	postive
GSDME	SENCR	0.563191807	6.18E-41	postive
NLRC4	SENCR	0.542550427	1.48E-37	postive
NLRP3	SENCR	0.488611176	9.42E-30	postive
PJVK	AC079160.1	0.562072053	9.56E-41	postive
SCAF11	AC079160.1	0.474925986	5.53E-28	postive
GSDME	AC092802.4	0.534232796	2.93E-36	postive
NLRC4	AC092802.4	0.485643496	2.32E-29	postive
PJVK	AC092802.4	0.415560236	3.57E-21	postive
SCAF11	AC092802.4	0.671857364	2.15E-63	postive
CHMP4A	AC004148.1	0.482950637	5.19E-29	postive
PJVK	AC004148.1	0.474543319	6.18E-28	postive
SCAF11	AC004148.1	0.432020158	6.27E-23	postive
CHMP2A	AC087286.2	-0.40987504	1.37E-20	negative
GSDME	AC087286.2	0.657642977	6.40E-60	postive
IL1A	AC087286.2	0.62204262	5.34E-52	postive
IL1B	AC087286.2	0.682359783	4.36E-66	postive
NLRC4	AC087286.2	0.525531676	6.09E-35	postive
NLRP3	AC087286.2	0.626624551	5.82E-53	postive
SCAF11	AC087286.2	0.676399123	1.52E-64	postive
PJVK	AC239584.1	0.42876762	1.42E-22	postive
NLRP1	LINC01679	0.473695249	7.91E-28	postive
SCAF11	SNRK-AS1	0.433603529	4.20E-23	postive
GSDME	AL355312.2	0.497624706	5.82E-31	postive
NLRC4	AL355312.2	0.40743623	2.43E-20	postive
SCAF11	AL355312.2	0.626067244	7.64E-53	postive
GSDME	NEAT1	0.592564761	3.58E-46	postive
IL1A	NEAT1	0.526564931	4.27E-35	postive

Pyroptosis-related Genes	lncRNA	cor	pvalue	Regulation
IL1B	NEAT1	0.578013596	1.64E-43	postive
NLRC4	NEAT1	0.458642869	5.57E-26	postive
NLRP3	NEAT1	0.504453018	6.68E-32	postive
SCAF11	NEAT1	0.625552279	9.81E-53	postive
GSDME	AC136604.2	0.443314399	3.43E-24	postive
PJVK	AC136604.2	0.421511771	8.50E-22	postive
SCAF11	AC136604.2	0.506208845	3.80E-32	postive
PJVK	NXT1-AS1	0.485362722	2.52E-29	postive
IRF1	LINC00996	0.442369182	4.39E-24	postive
NLRC4	LINC00996	0.415272685	3.83E-21	postive
NLRC4	AL512306.3	0.464004391	1.25E-26	postive
NLRP1	AL512306.3	0.405341486	3.94E-20	postive
SCAF11	AL512306.3	0.421581013	8.36E-22	postive
GSDME	AC007314.1	0.515737858	1.67E-33	postive
IL1B	AC007314.1	0.428508483	1.51E-22	postive
NLRC4	AC007314.1	0.484436679	3.33E-29	postive
PJVK	AC007314.1	0.404743927	4.52E-20	postive
SCAF11	AC007314.1	0.651233104	2.05E-58	postive
NOD1	C21orf62-AS1	0.432424983	5.66E-23	postive
PJVK	C21orf62-AS1	0.443682198	3.12E-24	postive
GSDMB	AC009283.1	0.411338666	9.73E-21	postive
CHMP2A	AL133342.1	-0.417830995	2.07E-21	negative
GSDME	AL133342.1	0.61035747	1.29E-49	postive
IL1A	AL133342.1	0.489505302	7.18E-30	postive
IL1B	AL133342.1	0.572267834	1.69E-42	postive
NLRC4	AL133342.1	0.588142103	2.38E-45	postive
NLRP3	AL133342.1	0.527438533	3.16E-35	postive
SCAF11	AL133342.1	0.663429668	2.60E-61	postive
CHMP2A	AC073073.2	-0.42115657	9.27E-22	negative
CHMP6	AC073073.2	-0.40671766	2.87E-20	negative
PJVK	AC073073.2	0.469380502	2.74E-27	postive
SCAF11	AC073073.2	0.676783601	1.21E-64	postive
GSDME	AC064836.1	0.436675021	1.92E-23	postive
PJVK	AC064836.1	0.418739342	1.67E-21	postive
SCAF11	AC064836.1	0.534885033	2.32E-36	postive
GSDME	HAND2-AS1	0.529931356	1.33E-35	postive
SCAF11	AC089999.4	0.476054519	3.98E-28	postive
GSDME	AC144831.1	0.55751023	5.55E-40	postive

Pyroptosis-related Genes	lncRNA	cor	pvalue	Regulation
NLRC4	AC144831.1	0.521954561	2.07E-34	postive
NLRP3	AC144831.1	0.495250434	1.22E-30	postive
SCAF11	AL139397.1	0.464433773	1.11E-26	postive
GSDME	AC012442.2	0.424768489	3.83E-22	postive
PJVK	AC012442.2	0.421319572	8.91E-22	postive
SCAF11	AC012442.2	0.43354899	4.26E-23	postive
GSDME	AL133371.2	0.541810324	1.93E-37	postive
IL1B	AL133371.2	0.413102154	6.41E-21	postive
NLRC4	AL133371.2	0.602483544	4.58E-48	postive
NLRP3	AL133371.2	0.479831845	1.31E-28	postive
SCAF11	AL133371.2	0.534250402	2.91E-36	postive
SCAF11	AC068724.2	0.430242388	9.81E-23	postive
CHMP2A	PAXIP1-AS2	-0.413913188	5.29E-21	negative
CASP8	PAXIP1-AS2	0.428831231	1.40E-22	postive
GPX4	PAXIP1-AS2	-0.416987275	2.54E-21	negative
PJVK	PAXIP1-AS2	0.43676969	1.87E-23	postive
SCAF11	PAXIP1-AS2	0.56635627	1.79E-41	postive
TIRAP	PAXIP1-AS2	0.445749782	1.81E-24	postive
GSDME	MIR3945HG	0.614860613	1.60E-50	postive
IL1A	MIR3945HG	0.655157798	2.48E-59	postive
IL1B	MIR3945HG	0.853694998	1.50E-135	postive
GSDMC	MIR3945HG	0.454217094	1.87E-25	postive
IL6	MIR3945HG	0.434887645	3.03E-23	postive
NLRC4	MIR3945HG	0.608838768	2.59E-49	postive
NLRP3	MIR3945HG	0.801364305	3.53E-107	postive
TNF	MIR3945HG	0.430113688	1.01E-22	postive
GSDME	MIR29B2CHG	0.541098304	2.50E-37	postive
NLRC4	MIR29B2CHG	0.437604375	1.51E-23	postive
NLRP3	MIR29B2CHG	0.427891932	1.76E-22	postive
NOD1	MIR29B2CHG	0.410166343	1.28E-20	postive
SCAF11	MIR29B2CHG	0.616993832	5.88E-51	postive
GSDME	AC092794.1	0.532155593	6.09E-36	postive
IL1B	AC092794.1	0.414638916	4.45E-21	postive
NLRC4	AC092794.1	0.481318238	8.45E-29	postive
PJVK	AC092794.1	0.443315689	3.43E-24	postive
SCAF11	AC092794.1	0.694069587	3.16E-69	postive
SCAF11	AL139099.3	0.442328835	4.44E-24	postive
GSDME	AL391422.4	0.406681971	2.89E-20	postive

Pyroptosis-related Genes	lncRNA	cor	pvalue	Regulation
ELANE	AC027682.6	0.432714844	5.26E-23	postive
PYCARD	PYCARD-AS1	0.471249019	1.60E-27	postive
GSDME	MACC1-AS1	0.439027358	1.05E-23	postive
PJVK	MACC1-AS1	0.409373235	1.54E-20	postive
SCAF11	MACC1-AS1	0.641945643	2.68E-56	postive
GSDME	AL162424.1	0.58749795	3.13E-45	postive
IL1A	AL162424.1	0.424226052	4.37E-22	postive
IL1B	AL162424.1	0.504109699	7.46E-32	postive
NLRC4	AL162424.1	0.507033044	2.91E-32	postive
NLRP3	AL162424.1	0.459414661	4.50E-26	postive
SCAF11	AL162424.1	0.443326401	3.42E-24	postive
PJVK	LINC01091	0.528332997	2.31E-35	postive
SCAF11	LINC01091	0.539456565	4.53E-37	postive
GSDMD	SNHG25	0.413483449	5.86E-21	postive
GSDME	AL354993.1	0.481782618	7.36E-29	postive
NOD1	AL354993.1	0.491291849	4.15E-30	postive
PJVK	AL354993.1	0.487692164	1.25E-29	postive
SCAF11	AL354993.1	0.533014031	4.50E-36	postive
GSDME	AC079385.1	0.419980653	1.23E-21	postive
IL1B	AC079385.1	0.421070687	9.46E-22	postive
SCAF11	AC079385.1	0.59503163	1.23E-46	postive
GSDME	AC108472.1	0.445728004	1.82E-24	postive
SCAF11	AC108472.1	0.515643238	1.73E-33	postive
CHMP2A	AC025165.5	-0.402610096	7.38E-20	negative
GSDME	AC025165.5	0.404025883	5.33E-20	postive
PJVK	AC025165.5	0.476764989	3.23E-28	postive
SCAF11	AC025165.5	0.694512998	2.39E-69	postive
CHMP2A	SOS1-IT1	-0.414912164	4.17E-21	negative
CHMP6	SOS1-IT1	-0.410582779	1.16E-20	negative
GSDME	SOS1-IT1	0.582661856	2.39E-44	postive
IL1A	SOS1-IT1	0.44216531	4.63E-24	postive
IL1B	SOS1-IT1	0.460083888	3.74E-26	postive
NLRC4	SOS1-IT1	0.491724384	3.64E-30	postive
PJVK	SOS1-IT1	0.413373217	6.01E-21	postive
SCAF11	SOS1-IT1	0.731995587	1.60E-80	postive
CHMP2A	AC090948.1	-0.42165335	8.21E-22	negative
GSDME	AC090948.1	0.520089593	3.89E-34	postive
NLRC4	AC090948.1	0.431314133	7.49E-23	postive

Pyroptosis-related Genes	lncRNA	cor	pvalue	Regulation
PJVK	AC090948.1	0.455098913	1.47E-25	postive
SCAF11	AC090948.1	0.700645525	4.68E-71	postive
PJVK	AC021683.2	0.477487372	2.62E-28	postive
GSDME	BX322234.1	0.663902072	2.00E-61	postive
IL1A	BX322234.1	0.543778046	9.45E-38	postive
IL1B	BX322234.1	0.73428551	2.89E-81	postive
NLRC4	BX322234.1	0.533401522	3.93E-36	postive
NLRP3	BX322234.1	0.630157296	1.03E-53	postive
SCAF11	BX322234.1	0.406176156	3.25E-20	postive
CHMP6	AC020891.3	-0.416605599	2.78E-21	negative
GSDME	AC020891.3	0.4273589	2.02E-22	postive
SCAF11	AC020891.3	0.606094164	9.02E-49	postive
GSDME	FLNB-AS1	0.437564821	1.53E-23	postive
SCAF11	FLNB-AS1	0.579015017	1.09E-43	postive
PJVK	AC007292.2	0.431108708	7.89E-23	postive
AIM2	BANCR	0.479409306	1.49E-28	postive
GSDME	LRP1-AS	0.462361091	1.99E-26	postive
IL1A	LRP1-AS	0.400790259	1.12E-19	postive
IL1B	LRP1-AS	0.448536805	8.63E-25	postive
NLRC4	LRP1-AS	0.40741523	2.44E-20	postive
SCAF11	LRP1-AS	0.602450059	4.65E-48	postive
GSDME	AC026401.2	0.550844538	6.92E-39	postive
IL1A	AC026401.2	0.425766465	2.99E-22	postive
IL1B	AC026401.2	0.46188439	2.27E-26	postive
NLRC4	AC026401.2	0.507080719	2.87E-32	postive
NLRP3	AC026401.2	0.459772188	4.08E-26	postive
SCAF11	AC026401.2	0.679855331	1.96E-65	postive
IRF1	AC022706.1	0.495497668	1.13E-30	postive
NLRC4	AC022706.1	0.46193131	2.24E-26	postive
NLRP1	AC022706.1	0.438381985	1.24E-23	postive
GZMA	AC022706.1	0.531785553	6.93E-36	postive
GSDME	AC138207.5	0.619645768	1.68E-51	postive
NLRC4	AC138207.5	0.726421505	9.64E-79	postive
NLRP3	AC138207.5	0.452508183	2.97E-25	postive
SCAF11	AC138207.5	0.458727151	5.44E-26	postive
PLCG1	LINC02538	0.461687388	2.40E-26	postive
PJVK	DGUOK-AS1	0.416321166	2.98E-21	postive
CHMP6	AC093297.2	-0.453299444	2.40E-25	negative

Pyroptosis-related Genes	lncRNA	cor	pvalue	Regulation
GSDMD	AC093297.2	-0.408380336	1.95E-20	negative
PJVK	AC093297.2	0.531033311	9.03E-36	postive
SCAF11	AC093297.2	0.653211386	7.09E-59	postive
SCAF11	AC007114.2	0.628962546	1.85E-53	postive
NOD1	AL009179.1	0.415032621	4.05E-21	postive
PJVK	AL009179.1	0.536978951	1.10E-36	postive
SCAF11	AL009179.1	0.472625181	1.08E-27	postive
SCAF11	AC079336.5	0.445052123	2.17E-24	postive
SCAF11	AL592301.1	0.441276943	5.84E-24	postive
GSDME	AC004637.1	0.547899186	2.07E-38	postive
IL1B	AC004637.1	0.415612492	3.53E-21	postive
NLRP3	AC004637.1	0.461295642	2.67E-26	postive
SCAF11	AC004637.1	0.553620703	2.44E-39	postive
CHMP2A	AC078846.1	-0.403643933	5.82E-20	negative
GSDME	AC078846.1	0.440871068	6.49E-24	postive
NOD1	AC078846.1	0.42036284	1.12E-21	postive
PJVK	AC078846.1	0.489466858	7.26E-30	postive
SCAF11	AC078846.1	0.591128112	6.64E-46	postive
CHMP2A	KCNQ1OT1	-0.422728721	6.31E-22	negative
GSDME	KCNQ1OT1	0.56124615	1.32E-40	postive
IL1A	KCNQ1OT1	0.415270492	3.83E-21	postive
IL1B	KCNQ1OT1	0.421405268	8.72E-22	postive
NLRC4	KCNQ1OT1	0.463201707	1.57E-26	postive
SCAF11	KCNQ1OT1	0.672335592	1.63E-63	postive
NOD1	AL354993.2	0.524745784	7.98E-35	postive
PJVK	AL354993.2	0.437180297	1.69E-23	postive
SCAF11	AL354993.2	0.427024526	2.19E-22	postive
GSDME	AF230666.1	0.476153631	3.87E-28	postive
NLRC4	AF230666.1	0.418863011	1.62E-21	postive
PJVK	AF230666.1	0.547311851	2.58E-38	postive
SCAF11	AF230666.1	0.684368602	1.29E-66	postive
CHMP2A	AC083805.2	-0.419236741	1.48E-21	negative
GSDME	AC083805.2	0.515788399	1.65E-33	postive
IL1A	AC083805.2	0.416779325	2.67E-21	postive
IL1B	AC083805.2	0.403838771	5.57E-20	postive
NLRC4	AC083805.2	0.425244069	3.40E-22	postive
SCAF11	AC083805.2	0.655023636	2.66E-59	postive
GSDME	MAP3K5-AS1	0.478520781	1.93E-28	postive

Pyroptosis-related Genes	lncRNA	cor	pvalue	Regulation
IL1A	MAP3K5-AS1	0.443461551	3.30E-24	postive
IL1B	MAP3K5-AS1	0.511113669	7.71E-33	postive
NLRC4	MAP3K5-AS1	0.404152323	5.18E-20	postive
NLRP3	MAP3K5-AS1	0.479027016	1.66E-28	postive
SCAF11	MAP3K5-AS1	0.539576032	4.34E-37	postive
NLRC4	BHLHE40-AS1	0.468865881	3.17E-27	postive
GSDME	GK-IT1	0.571700786	2.13E-42	postive
IL1A	GK-IT1	0.643239537	1.37E-56	postive
IL1B	GK-IT1	0.797007986	3.32E-105	postive
NLRC4	GK-IT1	0.480880872	9.62E-29	postive
NLRP3	GK-IT1	0.606707382	6.83E-49	postive
SCAF11	GK-IT1	0.512470015	4.94E-33	postive
GSDME	ZNF790-AS1	0.501233993	1.87E-31	postive
SCAF11	ZNF790-AS1	0.480895037	9.58E-29	postive
PJVK	AC004918.1	0.527000929	3.67E-35	postive
PJVK	CBR3-AS1	0.555496764	1.20E-39	postive
SCAF11	CBR3-AS1	0.433835174	3.96E-23	postive
PJVK	FMR1-IT1	0.601385867	7.47E-48	postive
SCAF11	FMR1-IT1	0.609043055	2.36E-49	postive
GSDME	AL139383.1	0.413767341	5.48E-21	postive
NOD1	AL139383.1	0.477590937	2.54E-28	postive
SCAF11	AL139383.1	0.434957494	2.98E-23	postive
GSDME	AC145423.3	0.416695208	2.72E-21	postive
SCAF11	AC145423.3	0.51476449	2.31E-33	postive
CHMP2A	DARS-AS1	-0.441714449	5.21E-24	negative
CHMP4B	DARS-AS1	-0.407975967	2.14E-20	negative
IL1B	DARS-AS1	0.413031041	6.52E-21	postive
SCAF11	DARS-AS1	0.42585131	2.93E-22	postive
GSDME	AC009262.1	0.436971051	1.78E-23	postive
NOD1	AC009262.1	0.464086622	1.22E-26	postive
PJVK	AC009262.1	0.44228564	4.49E-24	postive
SCAF11	AC009262.1	0.500722816	2.19E-31	postive
PJVK	AP003086.1	0.503112752	1.03E-31	postive
SCAF11	AP003086.1	0.567290648	1.23E-41	postive
HMGB1	UBR5-AS1	0.423206391	5.62E-22	postive
PJVK	UBR5-AS1	0.577648603	1.90E-43	postive
SCAF11	UBR5-AS1	0.447048792	1.28E-24	postive
GSDME	AL031770.1	0.577512085	2.01E-43	postive

Pyroptosis-related Genes	lncRNA	cor	pvalue	Regulation
IL1A	AL031770.1	0.412984393	6.60E-21	postive
IL1B	AL031770.1	0.415809593	3.37E-21	postive
NLRC4	AL031770.1	0.505022789	5.56E-32	postive
PJVK	AL031770.1	0.40957551	1.47E-20	postive
SCAF11	AL031770.1	0.689321353	6.19E-68	postive
PJVK	CCAT2	0.543684894	9.78E-38	postive
SCAF11	CCAT2	0.46412429	1.21E-26	postive
GSDME	AL109923.1	0.577778768	1.80E-43	postive
NLRC4	AL109923.1	0.465765862	7.63E-27	postive
PJVK	AL109923.1	0.427893515	1.76E-22	postive
SCAF11	AL109923.1	0.674616877	4.33E-64	postive
CHMP2A	AL513327.1	-0.400860334	1.10E-19	negative
GSDME	AL513327.1	0.589932746	1.11E-45	postive
IL1A	AL513327.1	0.461419109	2.58E-26	postive
IL1B	AL513327.1	0.522405521	1.77E-34	postive
NLRC4	AL513327.1	0.522551129	1.69E-34	postive
NLRP3	AL513327.1	0.440956293	6.35E-24	postive
SCAF11	AL513327.1	0.670765666	4.04E-63	postive
CHMP4B	SNHG17	0.442667022	4.07E-24	postive
CHMP2A	AC092168.2	-0.408135393	2.06E-20	negative
GSDME	AC092168.2	0.56400147	4.50E-41	postive
IL1B	AC092168.2	0.413102307	6.41E-21	postive
NLRC4	AC092168.2	0.493411307	2.16E-30	postive
SCAF11	AC092168.2	0.660452936	1.36E-60	postive
GSDME	AC078962.2	0.467429414	4.77E-27	postive
IL1A	AC078962.2	0.401040529	1.06E-19	postive
NLRC4	AC078962.2	0.407217674	2.55E-20	postive
SCAF11	AC078962.2	0.671713391	2.34E-63	postive
CHMP2A	AC004918.5	-0.424037944	4.58E-22	negative
GSDME	AC004918.5	0.470740399	1.85E-27	postive
NLRC4	AC004918.5	0.452266331	3.17E-25	postive
NOD1	AC004918.5	0.452443712	3.02E-25	postive
PJVK	AC004918.5	0.475329047	4.92E-28	postive
SCAF11	AC004918.5	0.714947279	3.24E-75	postive
NOD1	AL391684.1	0.47600319	4.04E-28	postive
PJVK	AL391684.1	0.568384328	8.00E-42	postive
SCAF11	AL391684.1	0.48646814	1.81E-29	postive
CHMP2A	AC005096.1	-0.410677957	1.14E-20	negative

Pyroptosis-related Genes	lncRNA	cor	pvalue	Regulation
GSDME	AC005096.1	0.523021967	1.44E-34	postive
IL1A	AC005096.1	0.429859342	1.08E-22	postive
IL1B	AC005096.1	0.416727394	2.70E-21	postive
NLRC4	AC005096.1	0.415777753	3.39E-21	postive
NLRP3	AC005096.1	0.430530312	9.12E-23	postive
SCAF11	AC005096.1	0.655638275	1.91E-59	postive
PJVK	AC009812.4	0.500176761	2.61E-31	postive
SCAF11	AC009812.4	0.489244773	7.77E-30	postive
CHMP4B	AC108134.3	0.480385645	1.11E-28	postive
PLCG1	AC108134.3	0.514711979	2.35E-33	postive
GSDME	AL365295.1	0.406148003	3.27E-20	postive
PJVK	AL365295.1	0.44165903	5.29E-24	postive
SCAF11	AL365295.1	0.4924181	2.94E-30	postive
CHMP4B	MHENCR	0.476397965	3.60E-28	postive
PLCG1	MHENCR	0.441816206	5.08E-24	postive
CHMP2A	SLFNL1-AS1	-0.40019404	1.28E-19	negative
GSDME	SLFNL1-AS1	0.410619375	1.15E-20	postive
PJVK	SLFNL1-AS1	0.469058174	3.00E-27	postive
SCAF11	SLFNL1-AS1	0.57695395	2.53E-43	postive
CHMP2A	LINC-PINT	-0.407551959	2.36E-20	negative
CHMP6	LINC-PINT	-0.420615509	1.06E-21	negative
GSDME	LINC-PINT	0.507310304	2.66E-32	postive
IL1A	LINC-PINT	0.414953447	4.13E-21	postive
IL1B	LINC-PINT	0.421560418	8.40E-22	postive
NLRC4	LINC-PINT	0.44628099	1.57E-24	postive
PJVK	LINC-PINT	0.483183923	4.84E-29	postive
SCAF11	LINC-PINT	0.718830433	2.17E-76	postive
GSDME	LINC01094	0.763722389	1.44E-91	postive
IL1A	LINC01094	0.460176843	3.64E-26	postive
IL1B	LINC01094	0.590478516	8.78E-46	postive
GSDMC	LINC01094	0.458160175	6.36E-26	postive
NLRC4	LINC01094	0.828979307	6.02E-121	postive
NLRP3	LINC01094	0.701863722	2.12E-71	postive
SCAF11	LINC01094	0.459034272	5.00E-26	postive
TNF	LINC01094	0.405999194	3.38E-20	postive
CHMP6	AC015727.1	-0.400135336	1.30E-19	negative
GSDME	AC015727.1	0.405923628	3.44E-20	postive
SCAF11	AC015727.1	0.556569129	7.96E-40	postive

Pyroptosis-related Genes	lncRNA	cor	pvalue	Regulation
CHMP2A	AC107068.1	-0.493223928	2.29E-30	negative
CHMP6	AC107068.1	-0.456756233	9.35E-26	negative
GSDME	AC107068.1	0.473442657	8.51E-28	postive
CASP8	AC107068.1	0.407147078	2.59E-20	postive
NLRC4	AC107068.1	0.446590249	1.45E-24	postive
PJVK	AC107068.1	0.451817369	3.58E-25	postive
SCAF11	AC107068.1	0.76106437	1.41E-90	postive
GSDME	AC027237.5	0.446882367	1.34E-24	postive
CHMP2A	AL355916.2	-0.408842946	1.75E-20	negative
GSDME	AL355916.2	0.490046869	6.08E-30	postive
NLRC4	AL355916.2	0.481425837	8.18E-29	postive
NLRP3	AL355916.2	0.407457201	2.41E-20	postive
SCAF11	AL355916.2	0.605617183	1.12E-48	postive
GSDME	PCAT19	0.408107701	2.07E-20	postive
NLRC4	PCAT19	0.466626354	5.99E-27	postive
PJVK	NFYC-AS1	0.586972197	3.91E-45	postive
SCAF11	NFYC-AS1	0.521755451	2.21E-34	postive
CHMP2A	ZBTB20-AS4	-0.415101312	3.99E-21	negative
GSDME	ZBTB20-AS4	0.559560298	2.53E-40	postive
IL1A	ZBTB20-AS4	0.468427252	3.59E-27	postive
IL1B	ZBTB20-AS4	0.478091573	2.19E-28	postive
NLRC4	ZBTB20-AS4	0.45840308	5.95E-26	postive
NLRP3	ZBTB20-AS4	0.40470728	4.56E-20	postive
SCAF11	ZBTB20-AS4	0.660183144	1.58E-60	postive
GSDME	AC040934.1	0.475581707	4.57E-28	postive
PJVK	AC040934.1	0.419196115	1.49E-21	postive
SCAF11	AC040934.1	0.583671945	1.57E-44	postive
CHMP2A	AC009090.3	-0.437464413	1.57E-23	negative
CHMP6	AC009090.3	-0.423090627	5.78E-22	negative
GSDME	AC009090.3	0.440538911	7.08E-24	postive
PJVK	AC009090.3	0.406036633	3.36E-20	postive
SCAF11	AC009090.3	0.67896259	3.34E-65	postive
GSDME	AL360270.1	0.404790356	4.47E-20	postive
SCAF11	AL360270.1	0.595166322	1.16E-46	postive
CHMP2A	LINC00624	-0.426205673	2.68E-22	negative
CHMP6	LINC00624	-0.411296352	9.83E-21	negative
GSDME	LINC00624	0.541887346	1.88E-37	postive
IL1A	LINC00624	0.426982949	2.21E-22	postive

Pyroptosis-related Genes	lncRNA	cor	pvalue	Regulation
IL1B	LINC00624	0.463423874	1.47E-26	postive
NLRC4	LINC00624	0.481878667	7.15E-29	postive
SCAF11	LINC00624	0.719473832	1.38E-76	postive
CHMP2A	AL031775.2	-0.422159186	7.26E-22	negative
GSDME	AL031775.2	0.536162647	1.47E-36	postive
IL1A	AL031775.2	0.412346259	7.67E-21	postive
IL1B	AL031775.2	0.453166502	2.49E-25	postive
NLRC4	AL031775.2	0.482161907	6.57E-29	postive
PJVK	AL031775.2	0.440255954	7.62E-24	postive
SCAF11	AL031775.2	0.687712079	1.67E-67	postive
GPX4	MID1IP1-AS1	-0.441922943	4.94E-24	negative
SCAF11	MID1IP1-AS1	0.522555442	1.69E-34	postive
CASP8	LINC01806	0.522324767	1.82E-34	postive
PJVK	LINC01806	0.431075573	7.95E-23	postive
SCAF11	LINC01806	0.451230024	4.19E-25	postive
GSDME	AL353804.2	0.468527582	3.49E-27	postive
NLRC4	AL353804.2	0.409950938	1.35E-20	postive
PJVK	AL353804.2	0.501181301	1.90E-31	postive
SCAF11	AL353804.2	0.66705836	3.37E-62	postive
GSDME	N4BP2L2-IT2	0.564061865	4.40E-41	postive
NLRC4	N4BP2L2-IT2	0.453602553	2.21E-25	postive
NOD1	N4BP2L2-IT2	0.431163568	7.78E-23	postive
SCAF11	N4BP2L2-IT2	0.636306655	4.77E-55	postive
PJVK	AC005165.1	0.491182811	4.29E-30	postive
SCAF11	AC005165.1	0.429358525	1.22E-22	postive
GSDME	AC108727.1	0.473602703	8.12E-28	postive
IL1A	AC108727.1	0.445920632	1.73E-24	postive
IL1B	AC108727.1	0.472281553	1.19E-27	postive
SCAF11	AC108727.1	0.643858773	9.96E-57	postive
CHMP2A	AC093726.1	-0.415269967	3.83E-21	negative
SCAF11	AC093726.1	0.556368971	8.59E-40	postive
PJVK	AC091729.2	0.44777877	1.06E-24	postive
IL1B	AC090617.5	0.405607324	3.71E-20	postive
NLRP3	AC090617.5	0.427786205	1.81E-22	postive
CHMP2A	AC073651.1	-0.418285634	1.86E-21	negative
GSDME	AC073651.1	0.443524262	3.25E-24	postive
SCAF11	AC073651.1	0.685649115	5.93E-67	postive
NOD1	STK24-AS1	0.466692197	5.88E-27	postive

Pyroptosis-related Genes	lncRNA	cor	pvalue	Regulation
CASP4	MIR4435-2HG	0.403880753	5.52E-20	postive
GSDME	MIR4435-2HG	0.544089267	8.43E-38	postive
NLRC4	MIR4435-2HG	0.493494962	2.11E-30	postive
NLRP3	MIR4435-2HG	0.470893595	1.77E-27	postive
GSDME	AP006259.1	0.604982712	1.49E-48	postive
IL1A	AP006259.1	0.643354248	1.29E-56	postive
IL1B	AP006259.1	0.742191331	6.80E-84	postive
NLRC4	AP006259.1	0.482755411	5.51E-29	postive
NLRP3	AP006259.1	0.635059984	8.94E-55	postive
SCAF11	AP006259.1	0.611201737	8.74E-50	postive
NOD1	AL357033.3	0.502394169	1.29E-31	postive
TIRAP	AL357033.3	0.400193164	1.28E-19	postive
GSDME	AL357874.2	0.48383758	3.98E-29	postive
NLRC4	AL357874.2	0.407868439	2.19E-20	postive
NOD1	AL357874.2	0.400428756	1.21E-19	postive
PJVK	AL357874.2	0.49215421	3.19E-30	postive
SCAF11	AL357874.2	0.573406715	1.07E-42	postive
GSDME	AC084824.5	0.465755017	7.66E-27	postive
NLRC4	AC084824.5	0.442955157	3.77E-24	postive
PJVK	AC084824.5	0.499859728	2.88E-31	postive
SCAF11	AC084824.5	0.749822619	1.59E-86	postive
GSDME	AC020763.1	0.436015338	2.27E-23	postive
NLRC4	AC020763.1	0.407895561	2.18E-20	postive
PJVK	AC020763.1	0.415162999	3.93E-21	postive
SCAF11	AC020763.1	0.557651682	5.26E-40	postive
GSDME	AL109614.1	0.477497672	2.61E-28	postive
NLRC4	AL109614.1	0.415437617	3.68E-21	postive
NOD1	AL109614.1	0.452127693	3.29E-25	postive
PJVK	AL109614.1	0.457919328	6.80E-26	postive
SCAF11	AL109614.1	0.70816913	3.26E-73	postive
NOD1	AC008667.1	0.447410448	1.16E-24	postive
IL1B	CLDN10-AS1	0.530602464	1.05E-35	postive
NLRC4	CLDN10-AS1	0.450993662	4.47E-25	postive
SCAF11	OR2A1-AS1	0.404483614	4.80E-20	postive
CHMP2A	HCG18	-0.400092861	1.31E-19	negative
GSDME	HCG18	0.530975202	9.21E-36	postive
NLRC4	HCG18	0.439436279	9.42E-24	postive
NOD1	HCG18	0.532942695	4.62E-36	postive

Pyroptosis-related Genes	lncRNA	cor	pvalue	Regulation
PJVK	HCG18	0.426485328	2.50E-22	postive
SCAF11	HCG18	0.685914603	5.04E-67	postive
SCAF11	AC027607.1	0.456069079	1.13E-25	postive
CHMP2A	LINC01376	-0.422102991	7.36E-22	negative
GSDME	LINC01376	0.465208204	8.93E-27	postive
NOD1	LINC01376	0.409285978	1.58E-20	postive
PJVK	LINC01376	0.405277288	4.00E-20	postive
SCAF11	LINC01376	0.625030207	1.26E-52	postive
SCAF11	AC069234.4	0.454648906	1.66E-25	postive
GSDME	SRD5A3-AS1	0.438625677	1.16E-23	postive
GSDME	AC008033.3	0.418771765	1.65E-21	postive
IL1A	AC008033.3	0.482067206	6.76E-29	postive
IL1B	AC008033.3	0.514957044	2.17E-33	postive
NLRP3	AC008033.3	0.503171941	1.01E-31	postive
SCAF11	AC008033.3	0.60608551	9.05E-49	postive
GPX4	MNX1-AS1	0.417203408	2.41E-21	postive
CHMP2A	GNG12-AS1	-0.417930921	2.02E-21	negative
GSDME	GNG12-AS1	0.514593964	2.45E-33	postive
NOD1	GNG12-AS1	0.440420799	7.30E-24	postive
PJVK	GNG12-AS1	0.402787238	7.09E-20	postive
SCAF11	GNG12-AS1	0.636280911	4.83E-55	postive
CHMP2A	AL020995.1	-0.435862471	2.36E-23	negative
CHMP6	AL020995.1	-0.417590996	2.20E-21	negative
GSDME	AL020995.1	0.503972852	7.79E-32	postive
NLRC4	AL020995.1	0.432695723	5.29E-23	postive
SCAF11	AL020995.1	0.700292507	5.88E-71	postive
CHMP2A	AC016405.1	-0.41869678	1.68E-21	negative
GSDME	AC016405.1	0.571493641	2.31E-42	postive
IL1B	AC016405.1	0.408920722	1.72E-20	postive
NLRC4	AC016405.1	0.484186047	3.59E-29	postive
NOD1	AC016405.1	0.417543619	2.22E-21	postive
PJVK	AC016405.1	0.404246899	5.07E-20	postive
SCAF11	AC016405.1	0.678621108	4.09E-65	postive
CHMP6	ACBD3-AS1	-0.405159489	4.11E-20	negative
GSDME	ACBD3-AS1	0.489302792	7.63E-30	postive
IL1A	ACBD3-AS1	0.41492716	4.16E-21	postive
IL1B	ACBD3-AS1	0.438825122	1.10E-23	postive
NLRC4	ACBD3-AS1	0.476589961	3.40E-28	postive

Pyroptosis-related Genes	lncRNA	cor	pvalue	Regulation
SCAF11	ACBD3-AS1	0.695916039	9.80E-70	postive
GSDME	AC145146.1	0.407401953	2.44E-20	postive
SCAF11	AC145146.1	0.675988788	1.94E-64	postive
PLCG1	AL121832.3	0.536688533	1.22E-36	postive
GSDME	AL356417.2	0.627511612	3.78E-53	postive
NLRC4	AL356417.2	0.549284117	1.24E-38	postive
NLRP3	AL356417.2	0.468730208	3.29E-27	postive
GSDME	AP002812.3	0.470163242	2.19E-27	postive
NLRC4	AP002812.3	0.407814778	2.22E-20	postive
PJVK	AP002812.3	0.533250251	4.14E-36	postive
SCAF11	AP002812.3	0.638287	1.75E-55	postive
SCAF11	AC008035.1	0.434150659	3.65E-23	postive
GSDME	AC005021.1	0.514699567	2.36E-33	postive
NLRC4	AC005021.1	0.470256351	2.13E-27	postive
NOD1	AC005021.1	0.453258563	2.43E-25	postive
PJVK	AC005021.1	0.430145248	1.00E-22	postive
SCAF11	AC005021.1	0.631986108	4.16E-54	postive
GSDME	AP001160.4	0.57708954	2.39E-43	postive
IL1A	AP001160.4	0.526867818	3.84E-35	postive
IL1B	AP001160.4	0.601722441	6.43E-48	postive
NLRC4	AP001160.4	0.507411813	2.58E-32	postive
NLRP3	AP001160.4	0.465066896	9.30E-27	postive
PJVK	AP001160.4	0.416521004	2.84E-21	postive
SCAF11	AP001160.4	0.631669025	4.87E-54	postive
SCAF11	AC005479.1	0.407666757	2.30E-20	postive
PJVK	RNF157-AS1	0.542864612	1.32E-37	postive
CASP8	SGMS1-AS1	0.460227686	3.59E-26	postive
GPX4	SGMS1-AS1	-0.40373159	5.71E-20	negative
PJVK	SGMS1-AS1	0.583053415	2.03E-44	postive
SCAF11	SGMS1-AS1	0.590554211	8.49E-46	postive
GSDME	AL360091.1	0.596827767	5.60E-47	postive
IL1A	AL360091.1	0.437788225	1.44E-23	postive
IL1B	AL360091.1	0.493742397	1.95E-30	postive
NLRC4	AL360091.1	0.547022143	2.87E-38	postive
SCAF11	AL360091.1	0.655609307	1.94E-59	postive
PJVK	AC024060.2	0.623148188	3.14E-52	postive
SCAF11	AC024060.2	0.496492525	8.30E-31	postive
CHMP2A	AC093535.1	-0.409785142	1.40E-20	negative

Pyroptosis-related Genes	lncRNA	cor	pvalue	Regulation
GSDME	AC093535.1	0.475430676	4.78E-28	postive
NLRC4	AC093535.1	0.436205669	2.16E-23	postive
PJVK	AC093535.1	0.426081076	2.77E-22	postive
SCAF11	AC093535.1	0.682386748	4.29E-66	postive
PJVK	OSGEPL1-AS1	0.485944639	2.11E-29	postive
GSDME	AC011899.2	0.494112489	1.74E-30	postive
NLRC4	AC011899.2	0.565103419	2.92E-41	postive
NLRP3	AC011899.2	0.425491604	3.20E-22	postive
PJVK	CCDC28A-AS1	0.574606431	6.58E-43	postive
CHMP2A	AC073896.3	-0.425567151	3.14E-22	negative
SCAF11	AC073896.3	0.565661476	2.35E-41	postive
GSDME	AL031666.2	0.429981386	1.05E-22	postive
PJVK	AL031666.2	0.404336631	4.97E-20	postive
SCAF11	AL031666.2	0.643325428	1.31E-56	postive
CHMP4B	AC004233.2	0.403248933	6.38E-20	postive
GSDME	AC000123.1	0.433111718	4.76E-23	postive
PJVK	AC000123.1	0.478957283	1.70E-28	postive
SCAF11	AC000123.1	0.727703239	3.79E-79	postive
SCAF11	AL133406.2	0.441568128	5.42E-24	postive
CHMP2A	LAMC1-AS1	-0.433007294	4.88E-23	negative
CHMP6	LAMC1-AS1	-0.402312623	7.90E-20	negative
GSDME	LAMC1-AS1	0.598057487	3.26E-47	postive
IL1A	LAMC1-AS1	0.47186019	1.34E-27	postive
IL1B	LAMC1-AS1	0.532222449	5.95E-36	postive
NLRC4	LAMC1-AS1	0.529290317	1.66E-35	postive
NLRP3	LAMC1-AS1	0.487003231	1.54E-29	postive
PJVK	LAMC1-AS1	0.451424721	3.98E-25	postive
SCAF11	LAMC1-AS1	0.708832218	2.09E-73	postive
GSDME	CHRM3-AS2	0.447525106	1.13E-24	postive
NLRC4	CHRM3-AS2	0.40396722	5.41E-20	postive
PJVK	CHRM3-AS2	0.402220186	8.07E-20	postive
SCAF11	CHRM3-AS2	0.567606013	1.09E-41	postive
IL1B	PRMT5-AS1	0.403208064	6.44E-20	postive
NOD1	SP2-AS1	0.433662992	4.14E-23	postive
PJVK	SP2-AS1	0.445363052	2.00E-24	postive
SCAF11	SP2-AS1	0.58868656	1.89E-45	postive
GSDME	AC008050.1	0.472567248	1.10E-27	postive
NLRC4	AC008050.1	0.419520414	1.38E-21	postive

Pyroptosis-related Genes	lncRNA	cor	pvalue	Regulation
PJVK	AC010864.1	0.450089581	5.70E-25	postive
CHMP2A	AC015987.1	-0.40169125	9.10E-20	negative
GSDME	AC015987.1	0.470656219	1.90E-27	postive
IL1B	AC015987.1	0.407040684	2.66E-20	postive
NLRC4	AC015987.1	0.438303085	1.26E-23	postive
SCAF11	AC015987.1	0.698054982	2.49E-70	postive
GSDME	LINC01705	0.526396184	4.52E-35	postive
NLRC4	LINC01705	0.432737468	5.23E-23	postive
NLRP3	LINC01705	0.411435138	9.51E-21	postive
CHMP3	U62317.2	-0.42339999	5.36E-22	negative
GSDMD	U62317.2	0.428671918	1.45E-22	postive
IRF1	U62317.2	0.632920979	2.61E-54	postive
AIM2	U62317.2	0.424801006	3.80E-22	postive
CHMP2A	RASA2-IT1	-0.420261541	1.15E-21	negative
CHMP6	RASA2-IT1	-0.402153275	8.19E-20	negative
GSDME	RASA2-IT1	0.590514871	8.64E-46	postive
IL1A	RASA2-IT1	0.468044329	4.00E-27	postive
IL1B	RASA2-IT1	0.485812028	2.20E-29	postive
NLRC4	RASA2-IT1	0.49468536	1.46E-30	postive
NLRP3	RASA2-IT1	0.404464893	4.82E-20	postive
SCAF11	RASA2-IT1	0.70580925	1.58E-72	postive
NOD1	Z68871.1	0.432951487	4.95E-23	postive
PJVK	Z68871.1	0.428537089	1.50E-22	postive
SCAF11	Z68871.1	0.692230248	1.01E-68	postive
CHMP2A	AC004223.2	-0.421993673	7.56E-22	negative
CHMP6	AC004223.2	-0.40654131	2.99E-20	negative
GSDME	AC004223.2	0.48678869	1.64E-29	postive
IL1A	AC004223.2	0.43330113	4.53E-23	postive
IL1B	AC004223.2	0.431564836	7.03E-23	postive
NLRP3	AC004223.2	0.400762313	1.12E-19	postive
SCAF11	AC004223.2	0.6933117	5.11E-69	postive
GSDME	AP002812.5	0.452712898	2.81E-25	postive
PJVK	AP002812.5	0.464579239	1.07E-26	postive
SCAF11	AP002812.5	0.564267001	4.06E-41	postive
GSDME	AC093010.2	0.565981593	2.07E-41	postive
PJVK	AC093010.2	0.446235708	1.59E-24	postive
SCAF11	AC093010.2	0.551980133	4.52E-39	postive
PJVK	KLHL7-DT	0.43185855	6.53E-23	postive

Pyroptosis-related Genes	lncRNA	cor	pvalue	Regulation
GSDME	HLA-F-AS1	0.476847401	3.16E-28	postive
NLRC4	HLA-F-AS1	0.432077101	6.18E-23	postive
SCAF11	BX284668.6	0.4908276	4.79E-30	postive
NOD1	AL355297.3	0.486722727	1.67E-29	postive
CHMP2A	INO80-AS1	-0.428624758	1.47E-22	negative
CHMP6	INO80-AS1	-0.410200574	1.27E-20	negative
GSDME	INO80-AS1	0.520089828	3.89E-34	postive
NLRC4	INO80-AS1	0.460688332	3.16E-26	postive
SCAF11	INO80-AS1	0.701272075	3.11E-71	postive
CHMP4A	AL049840.6	0.512342049	5.15E-33	postive
HMGB1	AL353796.1	0.433435899	4.38E-23	postive
PJVK	AL353796.1	0.48817394	1.08E-29	postive
SCAF11	AL353796.1	0.527621177	2.96E-35	postive
CHMP2A	AL031670.1	-0.43955246	9.14E-24	negative
CHMP6	AL031670.1	-0.419489448	1.39E-21	negative
GSDME	AL031670.1	0.519950011	4.08E-34	postive
IL1A	AL031670.1	0.433039846	4.84E-23	postive
IL1B	AL031670.1	0.47797198	2.27E-28	postive
NLRC4	AL031670.1	0.431888488	6.48E-23	postive
SCAF11	AL031670.1	0.675546236	2.51E-64	postive
CASP1	LINC01871	0.434411348	3.42E-23	postive
IRF1	LINC01871	0.657104126	8.59E-60	postive
AIM2	LINC01871	0.477482345	2.62E-28	postive
GZMA	LINC01871	0.721554298	3.18E-77	postive
GSDME	AC068790.4	0.438806759	1.11E-23	postive
SCAF11	AC068790.4	0.533403881	3.92E-36	postive
GSDME	CHN2-AS1	0.45482489	1.59E-25	postive
NOD1	CHN2-AS1	0.48591221	2.13E-29	postive
SCAF11	CHN2-AS1	0.506655808	3.29E-32	postive
CHMP2A	AC009704.2	-0.426735728	2.35E-22	negative
GSDME	AC009704.2	0.467075192	5.27E-27	postive
NLRC4	AC009704.2	0.454806915	1.59E-25	postive
SCAF11	AC009704.2	0.706878343	7.73E-73	postive
NOD1	AC004951.4	0.506190894	3.82E-32	postive
CHMP2A	LINC01409	-0.414352197	4.77E-21	negative
GSDME	LINC01409	0.468671978	3.35E-27	postive
NLRC4	LINC01409	0.403508099	6.01E-20	postive
NOD1	LINC01409	0.43533022	2.71E-23	postive

Pyroptosis-related Genes	lncRNA	cor	pvalue	Regulation
PJVK	LINC01409	0.452046321	3.37E-25	postive
SCAF11	LINC01409	0.673873972	6.68E-64	postive
CHMP2A	AC008026.3	-0.404550221	4.73E-20	negative
GSDME	AC008026.3	0.546072483	4.07E-38	postive
IL1A	AC008026.3	0.414511432	4.59E-21	postive
IL1B	AC008026.3	0.412345925	7.67E-21	postive
NLRC4	AC008026.3	0.465499542	8.23E-27	postive
PJVK	AC008026.3	0.443480137	3.29E-24	postive
SCAF11	AC008026.3	0.701874648	2.10E-71	postive
CHMP3	MKLN1-AS	0.426745067	2.35E-22	postive
CHMP2A	ATP1B3-AS1	-0.427909616	1.76E-22	negative
GSDME	ATP1B3-AS1	0.497357543	6.33E-31	postive
IL1A	ATP1B3-AS1	0.445454695	1.95E-24	postive
IL1B	ATP1B3-AS1	0.472646395	1.07E-27	postive
NLRC4	ATP1B3-AS1	0.434450748	3.39E-23	postive
NLRP3	ATP1B3-AS1	0.402040316	8.41E-20	postive
SCAF11	ATP1B3-AS1	0.722906345	1.21E-77	postive
CHMP4A	NSMCE1-DT	0.424367269	4.22E-22	postive
GSDME	NSMCE1-DT	0.401890347	8.70E-20	postive
NOD1	NSMCE1-DT	0.402966262	6.80E-20	postive
PJVK	NSMCE1-DT	0.507202805	2.76E-32	postive
SCAF11	NSMCE1-DT	0.482928873	5.23E-29	postive
CHMP4A	AC009690.2	0.430115091	1.01E-22	postive
GSDME	AC009690.2	0.514424934	2.59E-33	postive
IL1B	AC009690.2	0.456259532	1.07E-25	postive
NLRC4	AC009690.2	0.500664695	2.23E-31	postive
NLRP3	AC009690.2	0.455971045	1.16E-25	postive
NOD1	AC009690.2	0.40713778	2.60E-20	postive
SCAF11	AC009690.2	0.546287065	3.76E-38	postive
CHMP2A	AC006160.1	-0.413868666	5.35E-21	negative
GSDME	AC006160.1	0.523049755	1.43E-34	postive
NLRC4	AC006160.1	0.459491555	4.41E-26	postive
NLRP3	AC006160.1	0.405661186	3.66E-20	postive
NOD1	AC006160.1	0.414949797	4.13E-21	postive
SCAF11	AC006160.1	0.627322649	4.14E-53	postive
NLRC4	LINC02576	0.407185304	2.57E-20	postive
PJVK	LINC02576	0.494321158	1.63E-30	postive
SCAF11	LINC02576	0.513407009	3.63E-33	postive

Pyroptosis-related Genes	lncRNA	cor	pvalue	Regulation
PJVK	AC021683.1	0.435136542	2.84E-23	postive
GSDME	LRRC8C-DT	0.530512417	1.08E-35	postive
NLRC4	LRRC8C-DT	0.490838109	4.77E-30	postive
SCAF11	LRRC8C-DT	0.616972013	5.94E-51	postive
CHMP4B	AC009065.5	0.443124174	3.61E-24	postive
PYCARD	AC009065.5	0.461671563	2.41E-26	postive
CHMP2A	AC012313.5	-0.417669152	2.16E-21	negative
GSDME	AC012313.5	0.453555449	2.24E-25	postive
NLRC4	AC012313.5	0.415715458	3.44E-21	postive
SCAF11	AC012313.5	0.60877883	2.66E-49	postive
GSDME	AC107294.2	0.419159239	1.50E-21	postive
GSDME	AC005757.1	0.560275658	1.92E-40	postive
IL1A	AC005757.1	0.402689424	7.25E-20	postive
IL1B	AC005757.1	0.416711161	2.71E-21	postive
NLRC4	AC005757.1	0.459548934	4.34E-26	postive
SCAF11	AC005757.1	0.656769097	1.03E-59	postive
CASP6	MAPKAPK5-AS1	0.442596762	4.14E-24	postive
GSDME	AC023794.3	0.428303983	1.59E-22	postive
SCAF11	AC023794.3	0.526337413	4.62E-35	postive
SCAF11	AC012186.2	0.476704731	3.29E-28	postive
CHMP2A	AC005034.4	-0.417705128	2.14E-21	negative
CHMP6	AC005034.4	-0.426081076	2.77E-22	negative
GPX4	AC005034.4	-0.411855612	8.61E-21	negative
SCAF11	AC005034.4	0.703827961	5.84E-72	postive
SCAF11	GRPEL2-AS1	0.530428514	1.12E-35	postive
SCAF11	AC092902.2	0.4538173	2.08E-25	postive
GSDME	AC087893.3	0.53764136	8.70E-37	postive
IL1A	AC087893.3	0.494275816	1.65E-30	postive
IL1B	AC087893.3	0.566046416	2.02E-41	postive
NLRC4	AC087893.3	0.452594101	2.90E-25	postive
NLRP3	AC087893.3	0.495241375	1.23E-30	postive
SCAF11	AC087893.3	0.598762082	2.39E-47	postive
CHMP2A	AC244093.4	-0.42474012	3.85E-22	negative
CHMP6	AC244093.4	-0.406115941	3.29E-20	negative
GSDME	AC244093.4	0.514455419	2.56E-33	postive
IL1A	AC244093.4	0.424815375	3.78E-22	postive
IL1B	AC244093.4	0.437140896	1.70E-23	postive
NLRC4	AC244093.4	0.443140101	3.59E-24	postive

Pyroptosis-related Genes	lncRNA	cor	pvalue	Regulation
PJVK	AC244093.4	0.405159874	4.11E-20	postive
SCAF11	AC244093.4	0.698743965	1.60E-70	postive
GSDME	AC243960.3	0.417907033	2.04E-21	postive
NLRC4	AC243960.3	0.502157926	1.39E-31	postive
GSDME	AC079209.2	0.529124307	1.76E-35	postive
IL1A	AC079209.2	0.454672198	1.65E-25	postive
IL1B	AC079209.2	0.585016497	8.92E-45	postive
NLRC4	AC079209.2	0.49367734	1.99E-30	postive
SCAF11	AC079209.2	0.50905802	1.51E-32	postive
NOD1	LINC00412	0.461061472	2.85E-26	postive
PJVK	LINC00412	0.467807904	4.28E-27	postive
SCAF11	LINC00412	0.519546764	4.67E-34	postive
GSDME	AC016597.2	0.402199642	8.11E-20	postive
SCAF11	AC016597.2	0.522223586	1.89E-34	postive
GSDME	AC092681.2	0.444061037	2.82E-24	postive
SCAF11	AC092681.2	0.646021394	3.22E-57	postive
GSDME	PEF1-AS1	0.48511324	2.72E-29	postive
IL1A	PEF1-AS1	0.446817801	1.36E-24	postive
IL1B	PEF1-AS1	0.532523997	5.35E-36	postive
NLRP3	PEF1-AS1	0.511804441	6.15E-33	postive
SCAF11	PEF1-AS1	0.525610932	5.93E-35	postive
GSDME	AC005224.3	0.512598919	4.73E-33	postive
IL1B	AC005224.3	0.51165065	6.46E-33	postive
NLRC4	AC005224.3	0.476973857	3.04E-28	postive
NLRP3	AC005224.3	0.509962099	1.12E-32	postive
SCAF11	AC005224.3	0.419777113	1.30E-21	postive
GSDME	AL133330.1	0.52418014	9.69E-35	postive
IL1A	AL133330.1	0.503869865	8.05E-32	postive
IL1B	AL133330.1	0.559467827	2.62E-40	postive
NLRC4	AL133330.1	0.438437184	1.22E-23	postive
NLRP3	AL133330.1	0.483085998	4.99E-29	postive
SCAF11	AL133330.1	0.643717436	1.07E-56	postive
CHMP4A	LINC01588	0.415607456	3.53E-21	postive
CHMP2A	AP003392.1	-0.444231889	2.70E-24	negative
GSDME	AP003392.1	0.411856444	8.61E-21	postive
IL1B	AP003392.1	0.43168596	6.82E-23	postive
NLRC4	AP003392.1	0.426190942	2.69E-22	postive
NLRP3	AP003392.1	0.42495702	3.65E-22	postive

Pyroptosis-related Genes	lncRNA	cor	pvalue	Regulation
SCAF11	AP003392.1	0.561882093	1.03E-40	postive
GSDME	AL035530.2	0.653226819	7.03E-59	postive
IL1A	AL035530.2	0.617477293	4.68E-51	postive
IL1B	AL035530.2	0.71691428	8.30E-76	postive
NLRC4	AL035530.2	0.567164162	1.30E-41	postive
NLRP3	AL035530.2	0.708358977	2.87E-73	postive
SCAF11	AL035530.2	0.635891182	5.88E-55	postive
CHMP4B	AL009178.2	0.411223368	1.00E-20	postive
CHMP2A	AC073487.1	-0.428419118	1.55E-22	negative
CHMP6	AC073487.1	-0.413983496	5.20E-21	negative
GSDME	AC073487.1	0.515686748	1.70E-33	postive
IL1A	AC073487.1	0.4694256	2.70E-27	postive
IL1B	AC073487.1	0.508449577	1.84E-32	postive
NLRC4	AC073487.1	0.449088891	7.45E-25	postive
NLRP3	AC073487.1	0.44201467	4.82E-24	postive
SCAF11	AC073487.1	0.677574793	7.60E-65	postive
CHMP2A	AL162724.2	-0.407568159	2.35E-20	negative
GSDME	AL162724.2	0.506240324	3.76E-32	postive
NLRC4	AL162724.2	0.425297709	3.36E-22	postive
SCAF11	AL162724.2	0.576221427	3.41E-43	postive
GSDME	AC115102.1	0.521529334	2.39E-34	postive
IL1A	AC115102.1	0.442320258	4.45E-24	postive
IL1B	AC115102.1	0.521941519	2.08E-34	postive
NLRC4	AC115102.1	0.438444101	1.22E-23	postive
NLRP3	AC115102.1	0.49209261	3.25E-30	postive
SCAF11	AC115102.1	0.597403027	4.35E-47	postive
NOD1	NDUFA6-DT	0.449974444	5.87E-25	postive
PJVK	NDUFA6-DT	0.407886461	2.18E-20	postive
SCAF11	NDUFA6-DT	0.406161572	3.26E-20	postive
CASP8	NCK1-DT	0.418868231	1.61E-21	postive
GSDME	AC009120.3	0.421838185	7.85E-22	postive
IL1B	AC009120.3	0.404725908	4.54E-20	postive
NLRP3	AC009120.3	0.433209976	4.64E-23	postive
SCAF11	AC009120.3	0.530912152	9.42E-36	postive
NOD1	AL353801.3	0.413499469	5.84E-21	postive
PJVK	AL353801.3	0.487294087	1.41E-29	postive
SCAF11	AL353801.3	0.460254368	3.57E-26	postive
NLRC4	AL136084.3	0.466873802	5.58E-27	postive

Pyroptosis-related Genes	lncRNA	cor	pvalue	Regulation
PJVK	AL161729.3	0.40297935	6.78E-20	postive
CHMP2A	ST7-OT4	-0.422224316	7.14E-22	negative
GSDME	ST7-OT4	0.521220801	2.65E-34	postive
NLRC4	ST7-OT4	0.458739552	5.42E-26	postive
PJVK	ST7-OT4	0.404950396	4.31E-20	postive
SCAF11	ST7-OT4	0.727213577	5.42E-79	postive
GSDME	AC068234.2	0.59544051	1.03E-46	postive
IL1A	AC068234.2	0.401769916	8.94E-20	postive
IL1B	AC068234.2	0.507848078	2.24E-32	postive
NLRC4	AC068234.2	0.502861255	1.11E-31	postive
SCAF11	AC068234.2	0.52906019	1.80E-35	postive
GSDME	LINC01473	0.41068641	1.13E-20	postive
NOD1	LINC01473	0.441886601	4.98E-24	postive
PJVK	LINC01473	0.521065195	2.80E-34	postive
SCAF11	LINC01473	0.645017697	5.45E-57	postive
NOD1	AC006042.1	0.509427842	1.34E-32	postive
PJVK	AC006042.1	0.442359241	4.41E-24	postive
CHMP2A	AC006064.2	-0.434850945	3.06E-23	negative
CHMP6	AC006064.2	-0.425718796	3.03E-22	negative
GSDME	AC006064.2	0.540565692	3.04E-37	postive
IL1A	AC006064.2	0.425865568	2.92E-22	postive
IL1B	AC006064.2	0.419633585	1.34E-21	postive
NLRC4	AC006064.2	0.483774923	4.06E-29	postive
PJVK	AC006064.2	0.445295086	2.04E-24	postive
SCAF11	AC006064.2	0.757891594	2.06E-89	postive
HMGB1	AL355312.3	-0.448053411	9.81E-25	negative
GPX4	AL355312.3	0.475451808	4.75E-28	postive
PJVK	AL132765.2	0.447048283	1.28E-24	postive
GSDME	AL137244.1	0.452173941	3.25E-25	postive
NOD1	AL137244.1	0.474524549	6.22E-28	postive
SCAF11	AL137244.1	0.522376924	1.79E-34	postive
CHMP2A	AC079684.2	-0.448672513	8.32E-25	negative
CHMP6	AC079684.2	-0.415250356	3.85E-21	negative
PJVK	AC079684.2	0.431779936	6.66E-23	postive
SCAF11	AC079684.2	0.715414047	2.35E-75	postive
NOD1	AL590729.1	0.470653154	1.90E-27	postive
SCAF11	AL590729.1	0.529305265	1.65E-35	postive
CHMP2A	ZRANB2-AS1	-0.418980038	1.57E-21	negative

Pyroptosis-related Genes	lncRNA	cor	pvalue	Regulation
GSDME	ZRANB2-AS1	0.461763037	2.35E-26	postive
NLRC4	ZRANB2-AS1	0.423730658	4.94E-22	postive
NOD1	ZRANB2-AS1	0.422270063	7.06E-22	postive
PJVK	ZRANB2-AS1	0.434431719	3.40E-23	postive
SCAF11	ZRANB2-AS1	0.714377726	4.80E-75	postive
GSDME	AL442067.1	0.592866888	3.14E-46	postive
IL1A	AL442067.1	0.456536689	9.93E-26	postive
IL1B	AL442067.1	0.510264367	1.02E-32	postive
NLRC4	AL442067.1	0.49328069	2.25E-30	postive
SCAF11	AL442067.1	0.613470129	3.06E-50	postive
GSDME	NCOA7-AS1	0.546225932	3.85E-38	postive
IL1A	NCOA7-AS1	0.571545758	2.26E-42	postive
IL1B	NCOA7-AS1	0.613814859	2.60E-50	postive
NLRC4	NCOA7-AS1	0.407537289	2.37E-20	postive
NLRP3	NCOA7-AS1	0.631464187	5.39E-54	postive
SCAF11	NCOA7-AS1	0.583245437	1.88E-44	postive
GSDME	AC005332.6	0.401340343	9.86E-20	postive
PJVK	AC005332.6	0.447425657	1.16E-24	postive
SCAF11	AC005332.6	0.458873739	5.23E-26	postive
GSDME	AC016747.1	0.681847828	5.94E-66	postive
NLRC4	AC016747.1	0.685194367	7.82E-67	postive
NLRP1	AC016747.1	0.431810536	6.61E-23	postive
NLRP3	AC016747.1	0.618372787	3.07E-51	postive
SCAF11	AC016747.1	0.408397248	1.94E-20	postive
PJVK	AP001619.1	0.550131674	9.04E-39	postive
CHMP2A	AP000487.1	-0.415648648	3.50E-21	negative
CHMP6	AP000487.1	-0.405876887	3.48E-20	negative
GSDME	AP000487.1	0.504885466	5.82E-32	postive
NLRC4	AP000487.1	0.44059505	6.98E-24	postive
NOD1	AP000487.1	0.420013603	1.22E-21	postive
PJVK	AP000487.1	0.496757522	7.64E-31	postive
SCAF11	AP000487.1	0.677398462	8.43E-65	postive
CHMP4B	LINC01558	0.442687889	4.04E-24	postive
PLCG1	LINC01558	0.44012613	7.88E-24	postive
CHMP2A	AL606834.2	-0.451624772	3.77E-25	negative
GSDME	AL606834.2	0.492083299	3.26E-30	postive
NLRC4	AL606834.2	0.446183615	1.61E-24	postive
NLRP3	AL606834.2	0.418311313	1.85E-21	postive

Pyroptosis-related Genes	lncRNA	cor	pvalue	Regulation
SCAF11	AL606834.2	0.664962771	1.10E-61	postive
CHMP4A	AC025171.4	0.43021892	9.86E-23	postive
GSDME	AC025171.4	0.495518985	1.12E-30	postive
NLRC4	AC025171.4	0.455953345	1.17E-25	postive
PJVK	AC025171.4	0.479476222	1.46E-28	postive
SCAF11	AC025171.4	0.54149996	2.17E-37	postive
GSDME	GSN-AS1	0.515385292	1.88E-33	postive
IL1A	GSN-AS1	0.411829206	8.67E-21	postive
IL1B	GSN-AS1	0.436698471	1.91E-23	postive
NLRP3	GSN-AS1	0.497053858	6.96E-31	postive
SCAF11	GSN-AS1	0.537618898	8.77E-37	postive
NLRP1	LINC01781	0.411206349	1.00E-20	postive
GSDME	AL020997.3	0.49772856	5.64E-31	postive
NLRC4	AL020997.3	0.44139408	5.67E-24	postive
PJVK	AL020997.3	0.42061936	1.06E-21	postive
SCAF11	AL020997.3	0.67374526	7.20E-64	postive
NOD1	AL158063.1	0.452570563	2.92E-25	postive
GSDME	ZBTB20-AS5	0.566799875	1.50E-41	postive
IL1A	ZBTB20-AS5	0.403617262	5.86E-20	postive
NLRC4	ZBTB20-AS5	0.451528519	3.87E-25	postive
PJVK	ZBTB20-AS5	0.420959711	9.72E-22	postive
SCAF11	ZBTB20-AS5	0.672019571	1.96E-63	postive
CASP8	SNHG16	0.415773852	3.40E-21	postive
SCAF11	SNHG16	0.531049882	8.98E-36	postive
GSDME	AF117829.1	0.521543351	2.38E-34	postive
NLRC4	AF117829.1	0.453704517	2.15E-25	postive
PJVK	AF117829.1	0.552000408	4.49E-39	postive
SCAF11	AF117829.1	0.678654415	4.01E-65	postive
PJVK	CASC19	0.453410262	2.33E-25	postive
SCAF11	CASC19	0.556707901	7.55E-40	postive
PJVK	AC083843.3	0.471053165	1.69E-27	postive
SCAF11	AC083843.3	0.581363107	4.11E-44	postive
GSDME	AL353593.2	0.555740173	1.09E-39	postive
IL1A	AL353593.2	0.645931217	3.38E-57	postive
IL1B	AL353593.2	0.699286898	1.13E-70	postive
NLRP3	AL353593.2	0.663343949	2.73E-61	postive
SCAF11	AL353593.2	0.524238462	9.49E-35	postive
NOD1	AC023908.3	0.449183468	7.26E-25	postive

Pyroptosis-related Genes	lncRNA	cor	pvalue	Regulation
PJVK	AC023908.3	0.517425946	9.53E-34	postive
SCAF11	AC023908.3	0.453988386	1.99E-25	postive
GSDME	AC011899.3	0.411083737	1.03E-20	postive
SCAF11	AC011899.3	0.40090639	1.09E-19	postive
CHMP4A	AC116913.1	0.455731032	1.24E-25	postive
PJVK	AC116913.1	0.517394999	9.63E-34	postive
SCAF11	AC116913.1	0.492161334	3.18E-30	postive
NLRC4	LINC02285	0.482774122	5.47E-29	postive
NLRP1	LINC02285	0.407267444	2.52E-20	postive
GSDME	MAGI2-AS3	0.728833181	1.66E-79	postive
IL1A	MAGI2-AS3	0.521888255	2.12E-34	postive
IL1B	MAGI2-AS3	0.585071691	8.72E-45	postive
NLRC4	MAGI2-AS3	0.610497505	1.21E-49	postive
NLRP3	MAGI2-AS3	0.627613123	3.59E-53	postive
SCAF11	MAGI2-AS3	0.606617242	7.11E-49	postive
GSDME	FAM53B-AS1	0.532877217	4.72E-36	postive
NLRC4	FAM53B-AS1	0.439131155	1.02E-23	postive
NOD1	FAM53B-AS1	0.415959149	3.25E-21	postive
SCAF11	FAM53B-AS1	0.636406247	4.54E-55	postive
GSDME	AL356124.1	0.508295616	1.93E-32	postive
NLRC4	AL356124.1	0.445350847	2.01E-24	postive
PJVK	AL356124.1	0.428127027	1.66E-22	postive
SCAF11	AL356124.1	0.683817564	1.81E-66	postive
CHMP4A	AL135999.1	0.420908481	9.84E-22	postive
GSDME	AC129510.2	0.45130026	4.12E-25	postive
IL1A	AC129510.2	0.404627558	4.64E-20	postive
IL1B	AC129510.2	0.401780524	8.92E-20	postive
PJVK	AC129510.2	0.480730454	1.01E-28	postive
SCAF11	AC129510.2	0.554614511	1.67E-39	postive
CHMP4A	AC008434.1	0.415743167	3.42E-21	postive
SCAF11	AC008434.1	0.574311955	7.42E-43	postive
PLCG1	AL359881.1	0.40478149	4.48E-20	postive
GSDME	AC073592.1	0.458129275	6.42E-26	postive
SCAF11	AC073592.1	0.533910795	3.28E-36	postive
CHMP2A	AC018410.1	-0.402747294	7.15E-20	negative
GSDME	AC018410.1	0.548718516	1.53E-38	postive
IL1A	AC018410.1	0.49397239	1.82E-30	postive
IL1B	AC018410.1	0.51913058	5.38E-34	postive

Pyroptosis-related Genes	lncRNA	cor	pvalue	Regulation
NLRC4	AC018410.1	0.423282056	5.51E-22	postive
NLRP3	AC018410.1	0.520928683	2.93E-34	postive
SCAF11	AC018410.1	0.626719327	5.56E-53	postive
CHMP2A	AC098484.1	-0.414265927	4.87E-21	negative
GPX4	AC098484.1	-0.410185997	1.28E-20	negative
PJVK	AC098484.1	0.408540932	1.87E-20	postive
SCAF11	AC098484.1	0.648551028	8.51E-58	postive
SCAF11	AC016644.1	0.636513675	4.30E-55	postive
CHMP2A	AC016831.4	-0.408360951	1.96E-20	negative
GSDME	AC016831.4	0.45173859	3.66E-25	postive
NLRC4	AC016831.4	0.404005131	5.36E-20	postive
PJVK	AC016831.4	0.454073891	1.94E-25	postive
SCAF11	AC016831.4	0.70659401	9.35E-73	postive
PLCG1	AC084125.2	0.416031685	3.19E-21	postive
SCAF11	AL355483.2	0.449706139	6.31E-25	postive
GSDME	AC073957.1	0.5058967	4.20E-32	postive
NLRC4	AC073957.1	0.423890048	4.75E-22	postive
NOD1	AC073957.1	0.448271124	9.26E-25	postive
SCAF11	AC073957.1	0.616581639	7.14E-51	postive

Table S3.

gene	conMean	treatMean	logFC	pValue	fdr
LINC00174	0.536191005	1.191296604	1.151713731	1.23E-10	5.01E-10
STAG3L5P-PVRIG2P-PILRB	0.279961602	0.708892718	1.340338344	1.20E-11	5.63E-11
LINC00513	0.656762188	2.24508834	1.773329239	1.12E-08	3.32E-08
AC079907.1	0.233733943	0.586342813	1.326877143	5.86E-10	2.07E-09
AC010542.6	1.054459962	2.677765172	1.344525132	1.86E-12	1.03E-11
Z82243.1	0.190426288	0.916761654	2.267314048	7.09E-12	3.46E-11
PAN3-AS1	0.48323048	1.079128761	1.159083656	1.56E-08	4.38E-08
LINC02747	14.1917326	5.545353878	-1.355699298	4.33E-16	4.35E-15
AC018809.1	0.226766929	0.581761354	1.359217205	6.36E-06	1.16E-05
AC007342.5	0.534850711	1.337549659	1.322384292	1.70E-08	4.76E-08
LINC01315	1.030850955	4.118504909	1.998284952	8.42E-17	9.23E-16
SCAT2	0.183829003	0.861172521	2.227937885	2.19E-14	1.65E-13
AC007038.1	0.462811098	1.193304682	1.366467082	2.43E-07	5.83E-07
AL031716.1	0.318503884	0.963844098	1.597488842	3.25E-10	1.22E-09
LINC02487	0.307977001	1.167392159	1.92239476	7.57E-12	3.63E-11
GABPB1-AS1	0.524732023	1.24338066	1.244615303	3.85E-07	8.95E-07

DLEU2	0.34230637	1.238973699	1.855785516	5.58E-13	3.33E-12
PVT1	0.387063954	3.002800624	2.955664823	3.96E-26	2.13E-23
SNHG4	0.915584962	3.578926001	1.966761042	7.46E-19	1.21E-17
PABPC4-AS1	0.167462896	0.585203386	1.805096637	0.001800457	0.002212461
AC016831.1	0.09098045	0.925984083	3.347358925	2.01E-22	1.80E-20
PTOV1-AS2	1.283228426	2.738677175	1.093701211	8.63E-11	3.59E-10
AP003352.1	0.829257388	2.41376944	1.541396013	8.48E-19	1.34E-17
AC026368.1	0.073144759	0.642134341	3.134048748	1.67E-15	1.52E-14
PLAC4	0.010983289	0.743974848	6.08187183	6.19E-19	1.07E-17
LINC02441	15.03200152	2.962197075	-2.343297587	3.49E-22	2.68E-20
AC008115.3	0.732665758	1.97891883	1.433485341	3.27E-10	1.22E-09
LINC01355	0.398741944	1.038255491	1.380634223	1.24E-07	3.12E-07
LINC00265	1.019268453	2.198457381	1.108957489	2.50E-10	9.59E-10
MAFG-DT	0.374724754	3.314834526	3.145033665	7.86E-25	1.06E-22
AC020915.2	0.243234211	0.721251699	1.568156656	4.68E-05	7.15E-05
MIR17HG	0.157807247	1.429824724	3.179602938	4.79E-19	8.91E-18
AC113143.1	0.062150962	0.596735863	3.263243858	0.030163302	0.030852749
AL139089.1	0.388858159	1.471617889	1.920087204	4.76E-15	4.06E-14
AC108058.1	0.271417448	0.773865898	1.511570116	0.011445672	0.012543522
LINC02362	1.450980157	0.49892403	-1.540135728	4.14E-13	2.55E-12
AC026356.1	0.182409804	0.822678164	2.17314488	3.05E-17	3.72E-16
AL133410.1	0.362753268	0.929294263	1.357146891	3.99E-12	2.02E-11
AC011676.1	0.087524561	0.655641553	2.905147468	3.54E-16	3.66E-15
FENDRR	5.782781292	1.690043033	-1.774703558	2.38E-20	7.52E-19
AL080317.1	0.149475165	0.571773701	1.935538462	4.80E-10	1.73E-09
AC009065.3	7.809713714	16.98904161	1.1212629	3.49E-14	2.53E-13
AC093788.1	0.250890556	0.723105225	1.527147437	6.60E-08	1.74E-07
LENG8-AS1	0.99799771	2.369775945	1.247642253	1.54E-14	1.20E-13
AL138689.1	0.304048915	1.221805149	2.006638881	2.54E-06	5.04E-06
AC010834.3	0.467155979	1.177721677	1.334022399	4.99E-06	9.23E-06
AC009032.1	0.06994346	1.448730662	4.372456424	0.031231661	0.031884795
AC007128.1	0.017840389	0.623272803	5.126644757	4.12E-22	2.77E-20
AL354696.1	0.571796785	1.316014764	1.202601262	1.54E-10	6.17E-10
AL049539.1	0.048230772	0.695946392	3.850950376	3.99E-14	2.86E-13
AL928654.2	1.154732154	2.58688538	1.163657882	2.48E-07	5.91E-07
LINC01614	0.015179692	0.985324678	6.020384786	1.77E-17	2.21E-16
AP005899.1	0.223091029	0.65669907	1.557599911	0.000110621	0.000160985
AC007608.2	0.001369514	0.627810201	8.840520749	1.39E-07	3.47E-07
AC058791.1	0.172828095	1.299616999	2.910676854	8.58E-05	0.000126996

LINC00941	0.023853111	0.631167165	4.725772811	2.59E-15	2.24E-14
LINC02163	0.002926396	1.325400867	8.823088129	3.09E-21	1.19E-19
AC084117.1	0.237641226	0.735883392	1.630692038	4.55E-05	6.99E-05
AC009269.5	0.09428621	0.570440787	2.596958452	0.000927913	0.00119208
AL138963.1	0.093276623	0.995169661	3.415355034	0.000223796	0.000314603
AC100814.2	0.763822494	1.547545969	1.018672952	4.58E-06	8.64E-06
AC010761.1	0.92049821	2.47814479	1.428773661	2.36E-16	2.53E-15
AC103591.3	0.349667057	1.64368039	2.232876011	5.80E-07	1.29E-06
FIRRE	0.010701573	0.683801866	5.997683616	2.96E-20	8.83E-19
AP001429.1	0.056636393	1.107091803	4.28890166	2.70E-07	6.36E-07
AP000692.1	0.20811195	0.553746328	1.411865421	1.07E-05	1.87E-05
AC145207.8	0.26104736	0.540269493	1.049367654	0.003237375	0.003846173
ZNF433-AS1	0.369007868	0.789245931	1.096823339	7.95E-15	6.47E-14
SNHG20	0.966465657	2.333083854	1.271447789	2.15E-18	3.29E-17
LMO7-AS1	0.078464887	0.758371015	3.27278472	1.24E-17	1.59E-16
AC048341.2	0.565233377	1.445494262	1.354644318	6.06E-10	2.13E-09
TMEM147-AS1	0.626373708	2.220382677	1.825712781	1.45E-20	5.20E-19
AC015922.3	6.034200749	2.2584217	-1.417847797	7.25E-19	1.21E-17
LINC01811	0.005606982	0.828703433	7.207487775	2.40E-19	5.15E-18
AC132192.2	0.417676268	1.032964453	1.306333531	6.80E-12	3.35E-11
AC012360.3	0.282228519	0.628925454	1.156025246	1.64E-10	6.53E-10
AL049840.2	0.920708751	1.879774698	1.029742992	0.001817696	0.002228545
AC004908.2	0.401542813	0.881544899	1.134480235	5.22E-07	1.18E-06
AL034550.1	0.38183191	0.800712627	1.068346883	0.001731677	0.002137726
AC004837.2	0.129295776	0.735377802	2.507810485	3.42E-08	9.32E-08
AP002387.2	2.933200791	6.450162045	1.136859572	3.28E-08	9.00E-08
MCF2L-AS1	2.470693054	7.291302733	1.561260814	2.09E-13	1.33E-12
ZKSCAN2-DT	0.421689296	1.02649044	1.283467884	2.24E-09	7.24E-09
AC130371.2	1.812508642	0.486109378	-1.898635003	2.03E-21	8.40E-20
MIR100HG	1.459197931	0.713986255	-1.031207382	1.45E-06	2.98E-06
AL158837.1	1.164556707	0.502141042	-1.21361634	9.28E-16	8.74E-15
CAPN10-DT	0.290575998	0.745308372	1.358921925	4.87E-16	4.75E-15
AC115522.1	0.27823632	0.56316256	1.017240665	6.28E-06	1.15E-05
AC018653.3	0.438541007	1.164931079	1.409460948	3.25E-10	1.22E-09
AC063948.1	0.286343694	0.628703242	1.134631369	5.96E-06	1.10E-05
INE1	0.863148258	1.732633271	1.005286036	1.11E-05	1.91E-05
LINC02195	0.054761623	0.573376971	3.388246838	9.97E-12	4.74E-11
AC010536.2	0.256512039	0.861017	1.747015183	0.012622087	0.013748602
AL136115.2	0.055318642	0.777498055	3.813001421	2.70E-05	4.32E-05

AC005261.1	3.001028482	6.402482204	1.093174327	2.75E-14	2.03E-13
RUSC1-AS1	0.815245726	2.304706089	1.499275902	7.43E-14	5.18E-13
ALG13-AS1	0.337670619	1.092732693	1.694251968	0.038184073	0.038615531
AL442125.2	0.192763646	0.555285758	1.5263974	0.002704508	0.003263642
AC087222.1	0.124540952	0.580315873	2.220218175	3.70E-07	8.68E-07
AC055717.2	0.002987511	1.504354541	8.975984653	5.92E-17	6.63E-16
AC020978.3	0.139708919	0.570501275	2.029805982	0.000892272	0.001154579
SMIM25	0.658907623	1.782329776	1.435616175	1.18E-12	6.69E-12
AC099850.4	3.225716427	9.05167861	1.488565744	5.72E-19	1.02E-17
AL590723.1	0.206210915	0.682281096	1.726245549	0.001230506	0.001554781
AC019080.5	0.369386416	0.870964151	1.237482525	1.58E-07	3.91E-07
BCL2L1-AS1	0.08787788	1.025271	3.544361419	0.02855952	0.029436588
AL139384.1	0.221771521	0.650941518	1.553453824	4.39E-09	1.34E-08
AP006621.2	0.651923633	1.538627444	1.238869067	5.05E-05	7.68E-05
AC233728.1	0.258078945	0.68939948	1.417527766	1.45E-08	4.15E-08
AC138932.6	0.225476097	0.563903701	1.322474314	0.007024128	0.007858244
AC091057.1	0.485327239	1.034769919	1.092280282	1.55E-11	7.22E-11
AC121761.1	0.268384398	1.003255559	1.902316442	4.20E-14	2.97E-13
AC141002.1	0.253238957	0.5576527	1.138867551	0.000321253	0.000440084
AL356299.3	0.105481175	0.815907801	2.951420587	3.22E-19	6.65E-18
AC245884.8	0.37343235	1.187237426	1.668689659	6.34E-12	3.15E-11
AC008649.2	0.051843226	1.102149513	4.41002064	0.001014189	0.001296713
SERTAD4-AS1	2.838923882	1.034099481	-1.456969186	1.18E-08	3.43E-08
ARHGEF38-IT1	0.094761582	0.737797871	2.960851433	2.71E-09	8.70E-09
SCARNA9	0.469788009	7.090874825	3.915881834	2.97E-12	1.55E-11
AL161729.4	0.164393511	0.780221151	2.246729758	5.32E-12	2.67E-11
AL161891.1	0.139418692	0.568677479	2.028186674	4.35E-17	5.02E-16
AC007637.1	1.117732965	0.496414299	-1.17095898	2.74E-18	3.87E-17
DTNB-AS1	1.953052755	0.503826872	-1.954730942	1.97E-15	1.77E-14
AL355488.1	0.740985277	1.656777595	1.160863166	4.99E-06	9.23E-06
HIF1A-AS3	0.183174759	1.922161229	3.391436732	0.004285218	0.004959401
NCBP2-AS1	0.228808111	0.576239777	1.332531057	5.81E-06	1.07E-05
AL031985.3	0.455716383	1.220688373	1.421486802	9.53E-18	1.25E-16
AL133520.1	0.779760324	1.569913801	1.009582694	5.16E-07	1.17E-06
AC000061.1	0.16934003	0.958237616	2.500460402	9.67E-05	0.000142237
AC084125.4	0.397897521	1.03709072	1.382073286	1.09E-06	2.27E-06
AC025857.2	1.269421687	4.86081392	1.937026511	4.74E-19	8.91E-18
AC104695.4	0.254618026	1.916977647	2.912427047	3.90E-05	6.07E-05
AL035071.2	0.079781595	0.573804884	2.846432377	1.03E-05	1.80E-05

AC020907.4	0.28083252	1.222442188	2.121984325	2.34E-14	1.75E-13
AL354836.1	0.917089207	2.844912497	1.6332503	4.37E-16	4.35E-15
LINC02381	1.984890369	0.777902695	-1.351397716	2.71E-18	3.87E-17
LINC01235	0.236040955	0.6358771	1.429710753	1.79E-06	3.60E-06
AL121832.2	1.137982268	3.291242907	1.53215443	1.28E-15	1.19E-14
AP002336.2	0.165196921	0.570147153	1.787147527	1.18E-10	4.83E-10
Z83843.1	0.615517282	3.476779366	2.49788025	4.36E-05	6.73E-05
AC243967.2	0.093709092	0.638051091	2.767411008	6.26E-07	1.38E-06
AC004253.1	0.343672319	0.841821974	1.292481514	1.04E-06	2.16E-06
HM13-IT1	0.531299635	1.574333965	1.567143988	1.57E-11	7.26E-11
AC109322.1	0.627479299	1.285052206	1.034187202	5.32E-13	3.21E-12
LINC01138	0.406821763	0.87673436	1.107742931	6.14E-13	3.63E-12
Z94721.1	0.234559284	0.584531225	1.317327491	2.66E-07	6.30E-07
MIR222HG	0.637629335	1.648395526	1.370272544	3.49E-08	9.48E-08
AC015922.2	8.158355787	2.566953496	-1.668221265	1.66E-21	7.43E-20
AC016394.3	0.473160275	1.638365453	1.791856341	4.39E-17	5.02E-16
LINC02604	0.817551368	2.316278871	1.502427672	6.66E-13	3.89E-12
AC090739.1	0.357680261	1.215732848	1.765083827	0.000152343	0.000217575
MIR181A2HG	0.1362878	0.670286078	2.29812055	6.95E-09	2.10E-08
AC007938.3	0.276721708	0.799011882	1.529781135	4.25E-10	1.55E-09
MALAT1	7.994962623	131.4480175	4.039257285	2.45E-08	6.77E-08
AC006333.1	5.475964487	2.580458438	-1.085485697	1.73E-19	4.00E-18
AC092338.1	0.134231158	0.628867949	2.22803752	0.024898926	0.025812207
AC093732.1	0.029839412	0.583537373	4.289534023	1.54E-13	9.98E-13
AL096828.3	0.142975176	0.531379766	1.893978615	2.07E-12	1.12E-11
AC022211.2	0.413967639	0.902391935	1.124236182	0.000476906	0.00063865
ANKRD10-IT1	4.238488964	11.51423396	1.441796496	4.93E-06	9.20E-06
AC092910.3	0.265696075	0.574516789	1.112572139	1.62E-09	5.33E-09
AL021578.1	0.141787586	0.881351551	2.63598637	3.68E-12	1.88E-11
AC137630.3	0.237785037	0.608554326	1.355728125	0.003604709	0.00423573
PSPC1-AS2	0.392760577	0.956839234	1.28462642	1.74E-05	2.93E-05
TFAP2A-AS1	0.04018322	0.599963385	3.900209382	2.26E-15	1.99E-14
AL078587.1	0.106765309	0.731052652	2.775532363	5.55E-11	2.44E-10
AC074117.1	0.9638567	2.011721913	1.061540314	8.92E-14	6.07E-13
AC022150.4	0.206637717	0.674872786	1.707511968	0.043363504	0.043607119
AL139349.1	0.324389461	1.049890097	1.69443946	1.56E-08	4.38E-08
GK-AS1	0.247653697	1.103527809	2.155726926	0.000182234	0.000258205
AC015813.1	0.944762336	2.887714762	1.61190489	1.54E-10	6.17E-10
AC245100.7	0.188106902	0.632980234	1.750607662	8.07E-07	1.70E-06

AC004241.3	0.435791321	0.963319242	1.144376519	4.47E-08	1.20E-07
AL022322.1	0.724793121	1.689926055	1.221318953	9.91E-08	2.52E-07
AL031600.1	0.264144735	0.884868982	1.744135208	2.50E-11	1.12E-10
TSPOAP1-AS1	0.330672671	1.009245476	1.609801399	2.88E-10	1.10E-09
MED8-AS1	0.247046878	0.608525597	1.300533125	3.82E-09	1.19E-08
CD44-AS1	0.170170973	1.195230689	2.812232223	1.37E-13	8.96E-13
AC068790.5	0.154574628	0.533805669	1.788011095	0.000453761	0.000612235
AC092119.2	0.467779931	1.064957375	1.186893816	8.89E-08	2.28E-07
AC093620.1	0.421372576	1.43625925	1.769147855	2.75E-12	1.45E-11
AL117379.1	0.477144292	1.629264699	1.771723492	3.41E-10	1.26E-09
AL021707.6	1.606824188	3.218186787	1.00203598	5.56E-07	1.24E-06
MCM3AP-AS1	0.326483338	0.673430551	1.044519804	2.11E-13	1.33E-12
AL596223.2	0.121088696	0.564424898	2.220717444	5.97E-11	2.59E-10
C2orf27A	0.281043884	1.129542619	2.006871383	2.48E-18	3.70E-17
AL355075.2	0.397000858	0.886025004	1.158205286	2.66E-05	4.29E-05
LINC02595	0.047313632	1.040667505	4.459109466	1.01E-19	2.46E-18
AC005519.1	0.235718147	0.528554114	1.164988353	1.35E-06	2.78E-06
AC002128.2	0.283037806	0.632220074	1.159432073	0.002735385	0.003293501
PRR7-AS1	0.094701228	0.619933341	2.710658053	1.11E-21	5.43E-20
AP000786.1	0.206335638	0.649539694	1.65442467	0.019631315	0.020589876
AC092535.5	0.763944306	2.277368374	1.575828302	7.23E-12	3.50E-11
AC015849.3	0.962138796	2.216627778	1.204049595	4.33E-06	8.25E-06
WNT5A-AS1	0.341277505	0.700806948	1.038071756	0.00581611	0.006645215
LINC00114	0.24664557	0.776874458	1.655242104	1.13E-05	1.96E-05
THUMPD3-AS1	1.211711872	2.593217839	1.097696715	6.45E-11	2.77E-10
FTX	0.193368065	1.238558217	2.679240229	3.08E-06	5.98E-06
USP30-AS1	5.382795228	2.197533862	-1.292470147	7.97E-11	3.34E-10
EIF1AX-AS1	0.030596914	0.560459525	4.195152134	0.006401031	0.007236533
AL137782.1	0.24037268	0.818514011	1.76773418	1.05E-13	7.07E-13
AC048344.4	0.074043106	0.54511353	2.880119404	2.42E-16	2.55E-15
AC008870.2	0.281877962	0.673659957	1.256949858	3.72E-09	1.17E-08
AC018695.4	0.222748892	0.551842835	1.308839189	6.34E-07	1.39E-06
AL035071.1	1.866222217	3.869145447	1.05189418	1.38E-08	3.99E-08
SNHG11	2.004840765	5.111233877	1.350183952	5.07E-18	6.81E-17
UBE2R2-AS1	0.17636802	0.629722995	1.836128358	2.73E-06	5.38E-06
AC073957.3	0.719799901	1.489485505	1.049146276	1.88E-05	3.16E-05
AL162595.1	0.352866328	0.847344638	1.263827103	3.68E-11	1.63E-10
AC131971.1	0.053365264	0.75754462	3.827357967	2.68E-05	4.31E-05
AC254629.1	8.388990296	3.97250727	-1.078447318	3.29E-12	1.70E-11

AC009404.1	0.177873478	0.567851232	1.674661603	3.29E-18	4.52E-17
LINC02418	0.006824177	4.285042821	9.294438937	9.20E-20	2.35E-18
AC112496.1	0.205385709	1.01812079	2.309501026	0.000234573	0.000328892
AP000873.2	0.269880459	0.570139665	1.078994856	3.34E-06	6.45E-06
AC011468.1	0.642827485	1.317307206	1.035088311	4.93E-06	9.20E-06
AL117382.2	2.649522214	10.89373586	2.039694663	1.00E-08	2.97E-08
AL606834.1	0.370179901	0.817185796	1.142437563	2.29E-05	3.80E-05
DLEU1	0.532370825	1.099434982	1.046258873	1.46E-08	4.15E-08
AL031673.1	0.403463275	1.256573527	1.638985826	2.81E-09	8.99E-09
SNHG22	0.175241637	0.824491647	2.234159281	6.89E-07	1.47E-06
AC087277.2	1.810730518	0.782437465	-1.210524496	1.11E-13	7.37E-13
MBNL1-AS1	2.815611305	0.530347682	-2.408437816	1.79E-19	4.00E-18
AL133243.2	0.404950486	1.318029722	1.702565483	1.42E-05	2.42E-05
SNHG6	22.30177364	61.43249111	1.461843436	6.96E-22	3.74E-20
AP002907.1	0.200089712	0.689137779	1.784145458	8.89E-08	2.28E-07
AL357079.1	0.477407305	0.962593485	1.011706018	6.40E-07	1.40E-06
AL157838.1	0.268042513	0.603444807	1.170759985	0.000206021	0.000290376
AC062037.3	0.397532225	0.802916889	1.014178847	0.019169446	0.020184299
AC087588.2	0.482507399	1.043755584	1.113160943	9.83E-05	0.000144291
AC004492.1	0.418726402	0.948309551	1.17935018	0.039532168	0.039903711
AC020765.2	0.423678099	0.885194814	1.063026443	4.76E-07	1.09E-06
AP000866.6	0.142414479	0.554145035	1.960167786	1.55E-06	3.15E-06
NEAT1	10.80814616	37.89709548	1.809968191	0.005955787	0.00677597
MACC1-AS1	0.014264832	0.608254034	5.414139324	0.002038153	0.002487473
SNHG25	0.967888169	29.11773566	4.910913992	5.61E-20	1.51E-18
AL354993.2	0.18434656	0.640689733	1.797205375	0.00140499	0.001750533
MAP3K5-AS1	0.163080843	0.555027502	1.766971942	0.000693559	0.000917343
GK-IT1	0.062842341	0.57121952	3.18423644	1.14E-05	1.97E-05
FMR1-IT1	0.358469435	0.808231983	1.17291933	0.006676951	0.007516819
AL513327.1	0.272124028	0.634476813	1.221303091	0.000107659	0.0001571
SNHG17	2.753759536	12.94545672	2.232971374	6.43E-23	6.90E-21
AC092168.2	0.038407786	0.821549943	4.418877568	5.24E-07	1.18E-06
AC108134.3	0.40331326	2.341315425	2.537346564	6.85E-07	1.47E-06
MHENCR	2.258880094	8.461957917	1.905383819	4.81E-19	8.91E-18
LINC-PINT	0.484134958	1.639641385	1.759899133	1.93E-12	1.06E-11
AL353804.2	0.061110326	0.578397496	3.242573221	7.18E-06	1.30E-05
N4BP2L2-IT2	0.258799521	0.91708898	1.825226773	5.21E-05	7.91E-05
AC108727.1	0.10050287	0.610136916	2.601896325	0.018561674	0.019582748
MIR4435-2HG	0.635678655	1.978756252	1.638224357	5.17E-22	3.08E-20

AL109614.1	0.294118274	1.384125423	2.23450635	4.25E-08	1.14E-07
MNX1-AS1	1.072486207	4.273495223	1.994457416	5.33E-20	1.51E-18
ACBD3-AS1	0.330695498	0.883811031	1.418234534	2.46E-05	4.02E-05
AL121832.3	0.227277316	0.799286986	1.8142599	4.93E-15	4.14E-14
AC024060.2	1.764880465	3.638910703	1.043936176	4.78E-10	1.73E-09
AC000123.1	0.921666084	1.949252652	1.08060503	9.02E-07	1.89E-06
SP2-AS1	0.360194329	0.72391901	1.007052835	6.41E-10	2.24E-09
LINC01705	0.005419936	1.312204041	7.919500447	1.57E-20	5.26E-19
Z68871.1	0.223311367	0.648389502	1.53780404	1.43E-08	4.12E-08
AL353796.1	0.267909662	0.703111422	1.39200672	1.09E-14	8.57E-14
AL031670.1	0.355364825	0.839768118	1.240690132	0.000821405	0.001070618
LINC01871	5.778569727	2.631195391	-1.134994065	6.73E-11	2.85E-10
CHN2-AS1	0.032150256	0.767204444	4.576708953	2.54E-12	1.35E-11
AL133330.1	0.125802169	0.733103099	2.542859302	5.09E-13	3.11E-12
AC073487.1	0.216864905	0.645973246	1.574677813	1.90E-05	3.18E-05
AL162724.2	0.205176458	0.732718607	1.836394045	2.65E-05	4.29E-05
LINC01473	0.197177621	0.810155175	2.03870244	7.31E-10	2.53E-09
AC006042.1	1.647802156	3.766569157	1.192707983	6.02E-07	1.33E-06
AL355312.3	0.098529924	0.562457263	2.513109628	6.48E-07	1.41E-06
AC079684.2	0.28336659	1.000589089	1.820108053	2.46E-11	1.11E-10
AL442067.1	0.115027234	0.793537787	2.786323454	5.17E-08	1.38E-07
LINC01558	0.576888282	1.560622011	1.435757289	1.24E-05	2.12E-05
SNHG16	2.101056157	6.051035479	1.526067321	7.34E-25	1.06E-22
AF117829.1	0.208630722	0.663883357	1.669978169	1.05E-12	6.06E-12
CASC19	0.126995689	4.318680894	5.087739289	5.91E-25	1.06E-22
AL359881.1	0.041211281	0.870477458	4.400695716	3.86E-13	2.41E-12
AC016831.4	0.050568129	0.993387231	4.296055907	6.18E-06	1.13E-05
